# Copper-Catalyzed Ring-Opening Reactions of Alkyl Aziridines with B_2_pin_2_: Experimental and Computational Studies

**DOI:** 10.3390/molecules26237399

**Published:** 2021-12-06

**Authors:** Lucilla Favero, Andrea Menichetti, Cosimo Boldrini, Lucrezia Margherita Comparini, Valeria Di Bussolo, Sebastiano Di Pietro, Mauro Pineschi

**Affiliations:** Department of Pharmacy, University of Pisa, Via Bonanno 33, 56126 Pisa, Italy; a.menichetti90@gmail.com (A.M.); cosimo@boldrini.org (C.B.); lucrezia.comparini@phd.unipi.it (L.M.C.); valeria.dibussolo@unipi.it (V.D.B.); sebastiano.dipietro@unipi.it (S.D.P.)

**Keywords:** copper catalysis, diboron reagents, aziridines, aminoboronates, DFT study

## Abstract

The possibility to form new C–B bonds with aziridines using diboron derivatives continues to be a particularly challenging field in view of the direct preparation of functionalized β-aminoboronates, which are important compounds in drug discovery, being a bioisostere of β-aminoacids. We now report experimental and computational data that allows the individuation of the structural requisites and of reaction conditions necessary to open alkyl aziridines using bis(pinacolate)diboron (B_2_pin_2_) in a regioselective nucleophilic addition reaction under copper catalysis.

## 1. Introduction

**Scheme 1:**
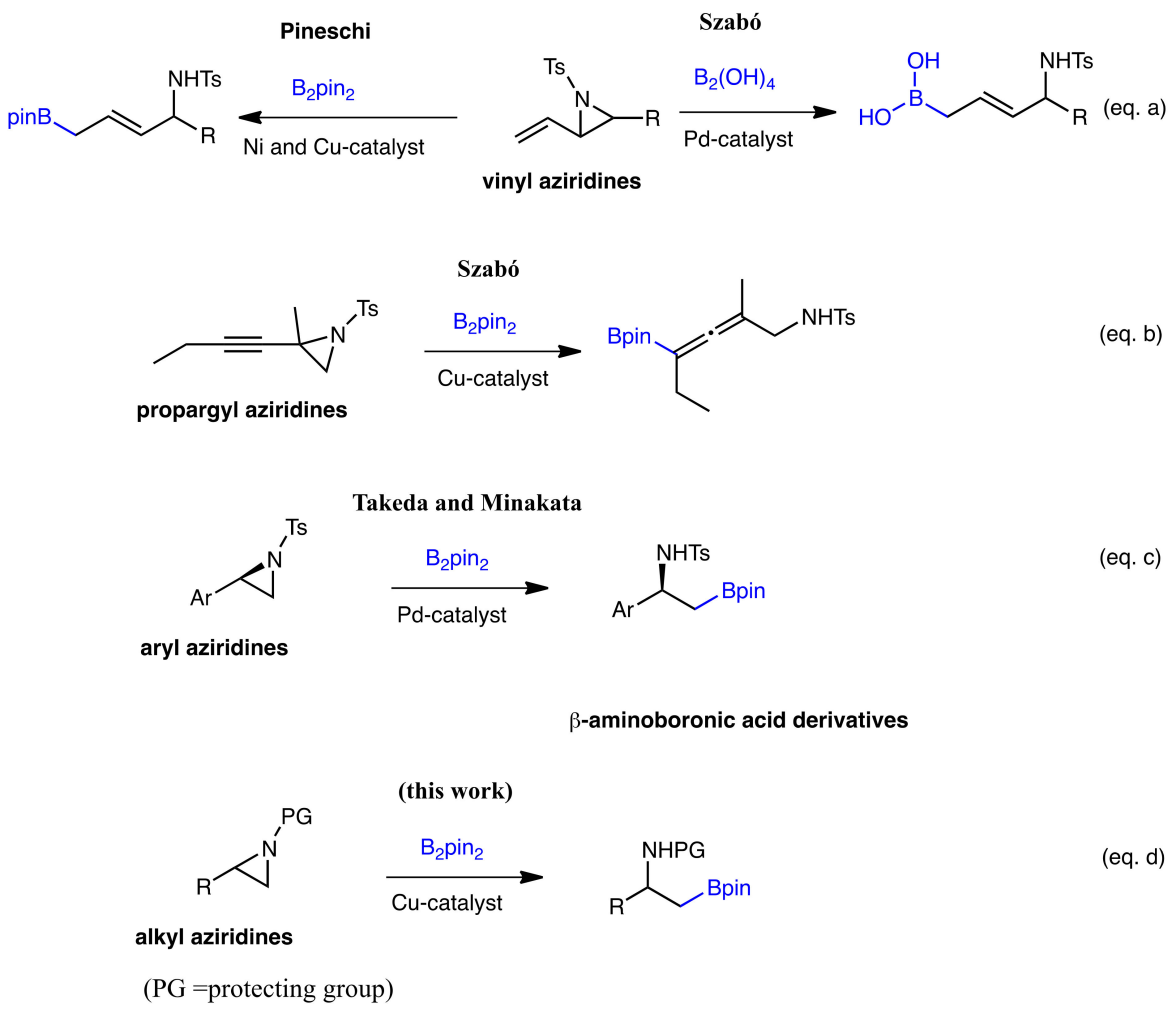
State of the art (eq. (**a**–**c**)) and aim of the work (eq. (**d**)).

The ring-opening (RO) reactions of aziridines with nucleophilic reagents are important operations in synthetic organic chemistry to obtain β-substituted amines [1,2,3,4]. Beside the consolidated use of heteroatom- and carbon-based nucleophiles, the use of nucleophilic boron reagents in the RO of strained three-membered heterocycles has come to the fore more recently [5,6,7,8]. The regio- and stereoselective introduction of a boron atom in sp^3^-rich functionalized molecules is of particular importance in synthetic organic chemistry, considering that the boron atom is easily transformable into hydroxy, amino or halo groups or it can serve as cross-coupling partner [9]. Moreover, there is a consolidated importance of incorporating the boron atom into new and existing drugs [10]. Hence, it is surprising that the ring opening of aziridines comprising the formation of carbon-boron bonds has been described so far only for aziridines bearing an adjacent double or triple bond. For example, allylic aziridines have been regioselectively borylated by using nickel and/or copper catalysts [11], or palladium catalysts as shown by Szabó (Scheme 1, eq. a) [12]. More recently, copper-catalyzed borylative ring openings of three- and four-membered rings bearing an adjacent triple bond including one example of a propargylic aziridines have also been reported (Scheme 1, eq. b) [13]. Aryl aziridines have been recently engaged by Takeda and Minakata in a regioselective borylative ring opening at the less substituted position using Pd/P(*t*-Bu)_2_Me as the catalyst and proceeding in neutral reaction conditions (Scheme 1, eq. c) [14]. In any case, these reaction conditions work only for tosyl-protected aryl aziridines, while they are ineffective for alkyl aziridines [15]. Indeed, a borylative ring opening of differently substituted alkyl aziridines has not yet been reported despite its potentiality to access β-aminoboronates, a scaffold of considerable interest in medicinal chemistry (Scheme 1, eq. d) [16,17,18].

We now report our findings about the individuation of the suitable substrates and the necessary reaction conditions to achieve the borylative ring-opening reactions of alkyl aziridines under copper-catalysis. We also report a detailed computational study which attempts to shed some light on the possible mechanistic intricacies of such a transformation.

## 2. Results and Discussion

### 2.1. Experimental Data

In studies made during the last several years in our laboratory, we had many confirmations of the difficulties associated with a direct borylation of alkyl aziridines. After an extensive screening of differently protected alkyl aziridines and reaction conditions, we noticed in the literature a direct borylative ring opening of alkyl epoxides with B_2_pin_2_ as reported by Xiao and Fu. The reaction conditions allowed the attainment β-hydroxyboronates in good yields by the use of CuI (15 mol%), *t*-BuOLi (3.0 equiv) in THF at 60 °C for 20 h [19]. Curiously, this protocol was particularly effective for terminal β-glycidyl epoxides generally containing an aryl moiety at the β-position, considerably reducing the generality of the borylative ring opening of alkyl epoxides. Importantly for this work, non-mechanistic rationale or tentative explanation was proposed by the authors to support the peculiar reactivity of phenylglycidol derivatives under copper catalysis.

Thus, having in mind to find alkyl aziridines amenable to copper borylative ring-opening reactions, phenylglycidol-derived aziridines **1a**–**c** containing electron-withdrawing protecting groups were prepared and subjected to reaction conditions as described in Table 1. Taking inspiration from the previous copper-catalyzed borylation of terminal epoxides [19], the reactions were carried out in THF at 60 °C for several hours. However, no appreciable conversion of protected aziridines **1a**,**b** into borylated products was observed (entries 1–5). Promisingly, we found that *N*-(2-picolinoyl) protected aziridine **1c** in combination with the use of freshly sublimed *t*-BuOK afforded the desired borylated product **2c**, albeit in a low isolated yield (entry 6). In order to try to increase the yields and to assess the ability of copper-phosphine complexes to influence the reaction outcome, a catalytic amount of racemic Binap was introduced into the reaction flask. In this way it was possible to increase the conversion of aziridine **1c** and to observe some borylated product **2c** also using *t*-BuONa (entry 7) or *t*-BuOLi as base (entry 9). However, in these reaction conditions, the major reaction product was oxazoline **3c** deriving from a Heine rearrangement of the starting aziridine [20,21]. Even in this scenario, one that clearly modifies the solubility of the reactive species involved in the reaction, the use of *t*-BuOK afforded the best result in term of borylative ring opening reaction (entry 8). The use of other phosphines of a different nature in the presence of *t*-BuOK afforded unsatisfactory amounts of borylated compound **2c** (entries 10–12). A decisive further improvement of the borylative ring opening of aziridine **1c** came from the use of catalytic amounts (20 mol%) of CuCl instead of CuI. To our delight, with this copper salt it was possible to perform the borylative ring-opening process to deliver desired compound **2c** with a satisfactory isolated yield at room temperature in 2 h (entry 13). It was also possible to reduce the amount of *t*-BuOK to 2.0 equivalents just increasing the reaction time to 5 h (entry 14). The reaction is possible also with CuI, albeit the quality of the crude reaction mixture and the isolated yields were lower (entry 15). This amount of base was found to be critical as a further diminishing to 1.5 equivalents gave no appreciable borylation product after 72 h (entry 16).

**Scheme 2:**
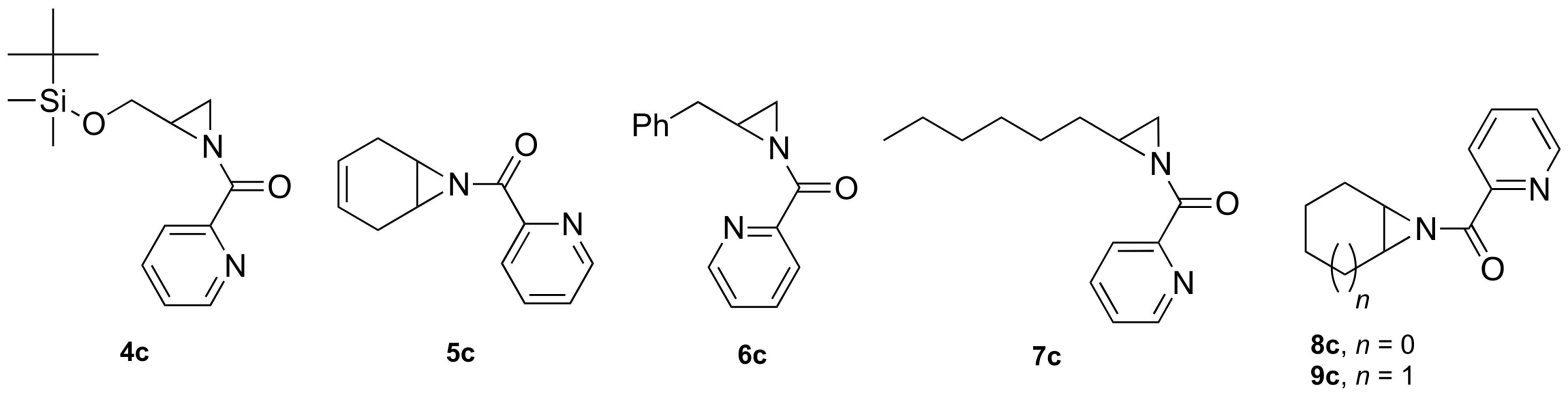
Picolinoyl-protected aziridines **4**–**9c** screened under optimized reaction conditions.

Once the optimal reaction conditions (entry 14, Table 1) to perform the borylative ring opening of aziridine **1c** had been found, other *N*-(2-picolinoyl)-protected aliphatic aziridines, such as compounds **4c**–**9c**, were prepared and tested (Scheme 2). Disappointingly, we soon realized the very narrow scope of the borylative ring opening as substituted aziridines **4c**–**9c** gave low conversion, decomposition or both to unidentified by-products without the attainment of the corresponding desired β-aminoboronates.

**Scheme 3:**
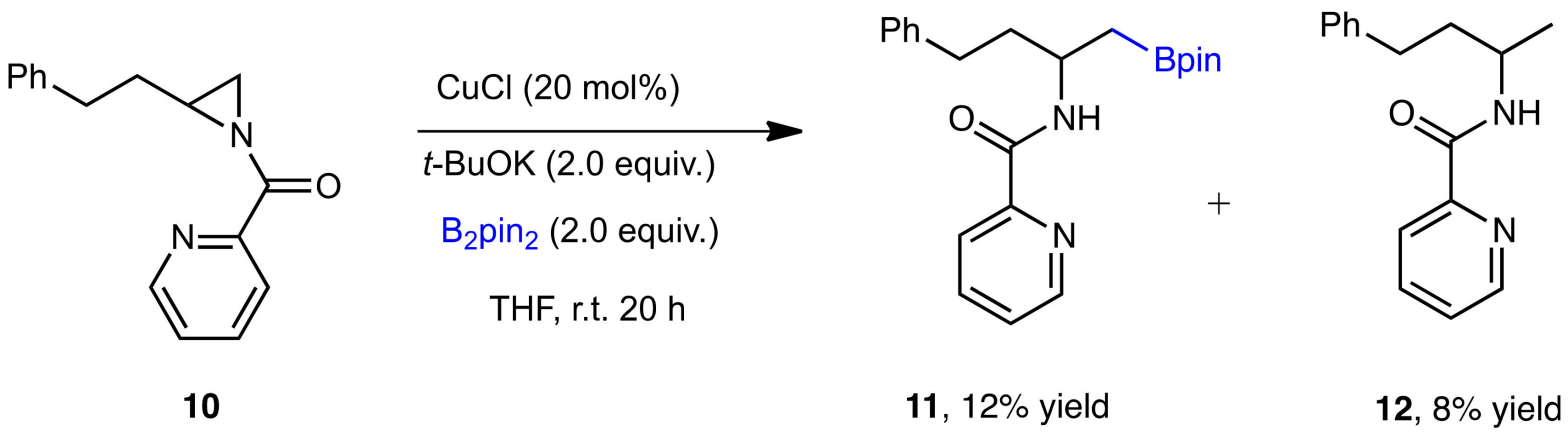
Ring-opening reaction of aziridine **10** under copper-catalyzed borylative conditions.

In order to gain some other insights on the requirements necessary to achieve the borylation and on the importance of a phenyl moiety in a β-position with respect to the three-membered ring, aziridine **10** was subjected to the optimized reaction conditions (Scheme 3). The borylative ring-opening reaction now occurred again to produce β-aminoboronate **11**, albeit with a low isolated yield. Curiously, also *N*-(2-picolinoyl)-aziridine **12** deriving from an unexpected reduction pathway was isolated from the reaction mixture.

Relying on this experimental data, the fundamental role of the 2-picolinoyl protective group and the structural requirements of the aziridine substrate for a successful borylative ring-opening process have been rationalized by DFT calculations in the following computational section.

### 2.2. Computational Studies

Experimental data clearly showed that aziridine substrates analyzed can be divided into three main categories: inert aziridines (**1a**,**b**), reactive aziridines devoid of a borylative outcome (**4c**–**9c**), and reactive aziridines forming borylation products (**1c** and **10**). Interestingly, the 2-picolinoyl protective group appeared to be essential for the occurrence of a ring-opening process, while the structure of the lateral chain, in particular the presence of an aromatic ring (**1c** and **10**), determined a borylative ring-opening reaction which occurred exclusively with an *anti*-Markovnikov regioselectivity. All these factors have been considered for the construction of a mechanistic model as detailed below.

**Scheme 4:**
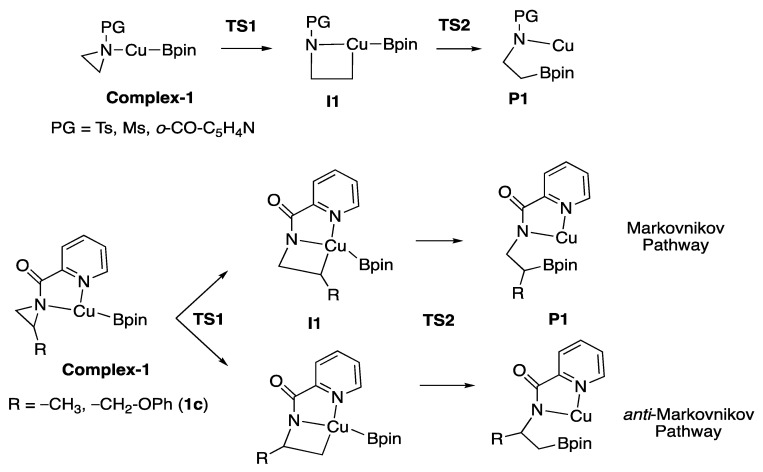
Preliminary mechanistic model applied to differently protected non-substituted aziridine (above) and to *N*-(2-picolinoyl)-methyl aziridine and aziridine **1c** (below).

Usually, more the protecting group of the aziridine is electron-withdrawing, more the substrate is reactive, in particular, towards nucleophilic ring-opening processes [22,23,24]. In our case, only the *N*-(2-picolinoyl)-aziridines were reactive (**1c**, **4c**–**9c** and **10**), while, surprisingly, the benzensulfonated ones (**1a**), even if fluorine substituted on the protective group (**1b**), were completely inert. These pivotal observations represented a deep divergence from the experimental and mechanistic data reported by Takeda et al. for the Pd-catalyzed borylation of styrene-derived tosyl-aziridine [14,15] by means of a zwitterionic intermediate [25]. It is of note that the intervention of protic solvents promoted a stabilization of the charged intermediate, while in our case, even a slight presence of water or other protic solvents was detrimental. In addition, copper with respect to other transition metals can be efficiently coordinated by nitrogen-based molecules such as a pyridine ring. An interesting alternative to the mechanism proposed by Takeda et al. focused on azametallacyclobutane intermediates, which derive from oxidative addition of aziridines to low-valent late transition metal complexes [26]. Lin et al. in 2002 isolated a series of nickel(II) azametallacyclobutanes from the reaction of several alkyl *N*-tosyl aziridines (with also a XRD structure) with Ni(bpy)(cod) or NiEt_2_(bpy), which could be consistent with the particular adduct needed in order to rationalize our borylation process [27]. On this basis, we initially hypothesized a simple model (Scheme 4) in which the borylation is preceded by the opening of the protected aziridine to give rise to a four-membered azacopper intermediate **I1**, which promoted the successive borylation [28].

**Figure 1:**
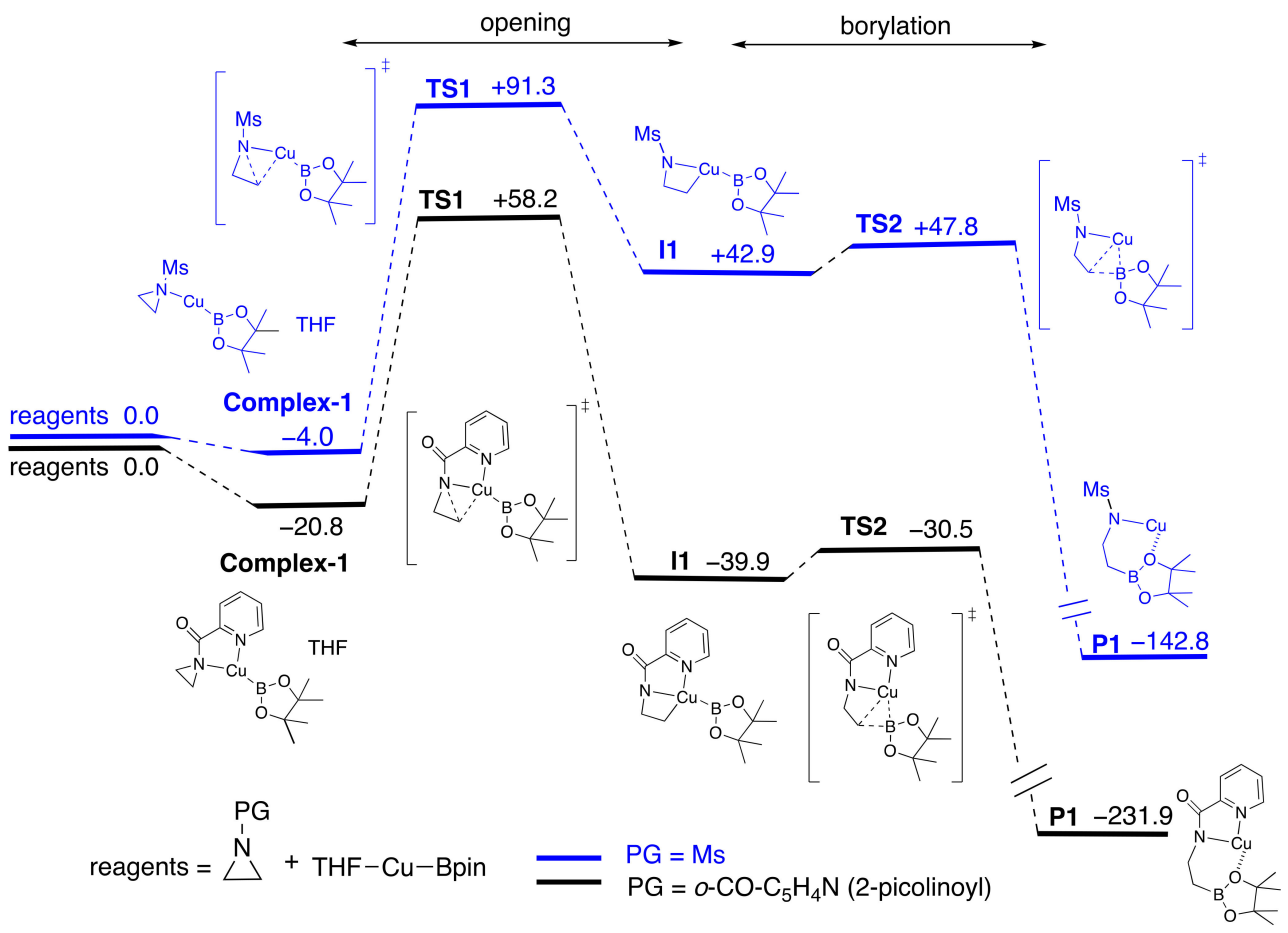
Comparative calculated reaction energy profile of *N*-mesyl-azirdine (blue) and *N*-(2-picolinoyl)-aziridine (black) using the mono-copper-boron borylation adduct (the ΔG values are in kJ/mol). See also Table 2 below.

We performed a first calculation using this model (see Supplementary Materials for details) to rationalize the inertness of the sulfonyl-protected aziridines to the reaction conditions, evaluating the activation energy of the ring-opening process for differently protected non-substituted aziridines (Table 2, Figure 1). This simple model showed that the ring-opening process (**TS1**) of a tosyl/mesyl aziridine possessed a higher activation energy of ca. 15 kJ/mol (+16.3 kJ/mol for the *N*-mesyl and +14.9 kJ/mol for the *N*-tosyl) with respect to the *N*-(2-picolinoyl)-aziridine. Particularly noteworthy is that the first four-membered azacopper intermediate **I1** was also thermodynamically not favoured, with a ΔG = +46.9 kJ/mol and +37.9 kJ/mol, respectively for *N*-mesyl and *N*-tosyl, with respect to a ΔG = −19.1 kJ/mol for the *N*-(2-picolinoyl)-methyl aziridine.

The next step consisted of the tentative rationalization of the role of the lateral chain on the energy reaction profile and on the regiochemistry for *N*-(2-picolinoyl)-aziridines: we chose the *N*-protected methyl aziridine as a model for simple alkyl substrates and compound **1c** as representative of aromatic substituted substrates, actually the only compound capable of undergoing a borylative ring opening (Table 3).

**Figure 2:**
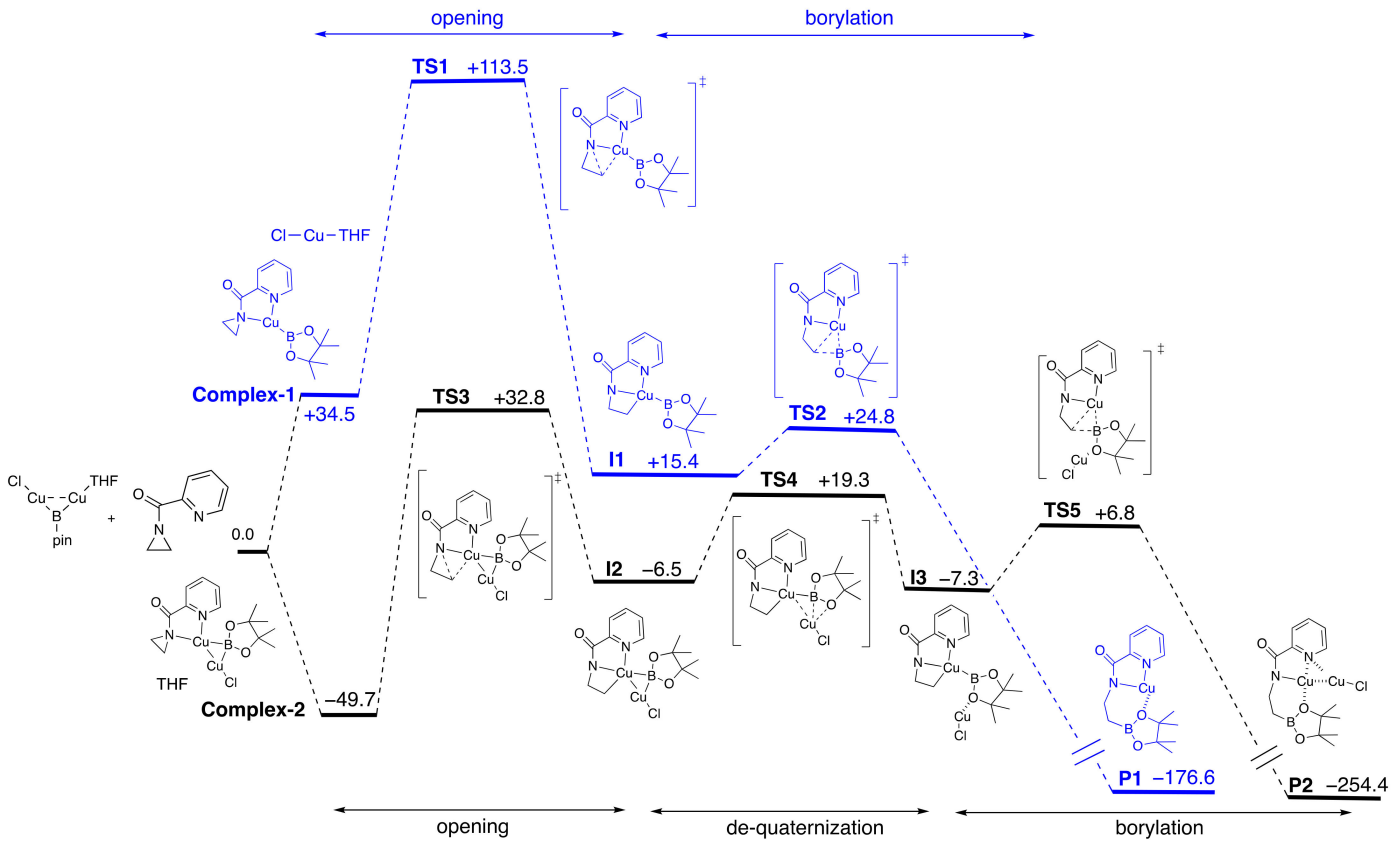
Comparative calculated reaction energy profile between the first (blue) and the second model (black) using a dicopper-boron borylation adduct on the non-substituted aziridine (the ΔG values are in kJ/mol).

With this model, the energy profiles of the reaction towards both the Markovnikov and *anti*-Markovnikov products for methyl aziridine and aziridine **1c** were similar to that of the non-substituted aziridine of the first calculation (Figure 1, Table 2). Unfortunately, this model failed to reproduce the effect of the lateral chain on the reaction outcome (Table 3): for both the aziridines considered, the kinetically determinant step was the ring opening process (**TS1**), while the boron transfer step (**TS2**) was very fast with an almost negligible activation energy. Following this model, alkylaziridines like **7c** should also give borylation products, which was actually not the case. Moreover, the regiochemistry for the borylation of **1c** was not in agreement with the experimental data, which shown the sole *anti*-Markovnikov product: in fact, the ring opening step (**TS1**) was poorly regioselective for both substituted *N*-(2-picolinoyl) aziridines considered with a ΔΔG^‡^ = 3.5 kJ/mol for methyl aziridine and ΔΔG^‡^ = 1.5 kJ/mol for **1c** in favor of the Markovnikov pathway. At this point, considering that the model so far adopted was unsatisfactory, we moved towards a new mechanistic model in which the boron quaternization was realized with the intervention of a second copper atom in the form of a molecule of CuCl. A first calculation (Figure 2, Table 4) was realized as a comparison between the first model with this new borylation agent through a monocopper complex on the non-substituted aziridine (**Complex-1**), and a second new model in which the di-copper/boron reagent formed a new complex (**Complex-2**) with the substrate in which it retained this second copper atom; the dicopper boron agent served to assess a better comparison between the two pathways. These kinds of dicopper-boron compounds such as **Complex-2** have been reported in the literature [29,30,31,32], and they are commonly obtained by reaction of copper(I) *tert*-butoxide with various diboron reagents (B_2_pin_2_, B_2_cat_2_), with the additional intervention of phosphine or carbene ligands. Similarly, in our case, the borylating agent can be obtained by reaction of in situ formed copper(I) *tert*-butoxide with B_2_pin_2_. Importantly, in our model the choice of the chloride counterion on the second copper ion was mainly for the sake of computational simplicity (neutral adduct), but it could possibly be replaced by a *tert*-butoxide anion [33] or even a solvent molecule.

Interestingly, the energy pathway originated from **Complex-2** for the second model is strongly favored with respect to **Complex-1** in the first model, with the presence of a new extra step also consisting in the boron de-quaternization (**TS4**), followed by the actual borylation step (**TS5**). Even with the second model, the rate determinant moment was again the aziridine ring opening process (**TS3**), but the energy difference with respect to all the other transition states (**TS4** and **TS5**) appeared to be drastically reduced in comparison with the mono-copper model. In addition, the azacoppercyclobutane intermediate **I2** (see Figure 2 and Table 4) possessed +43.2 kJ/mol with respect to the starting **Complex-2** and the de-quaternization process resulted more energy demanding (**TS4**, ΔG^‡^ of 12.5 kJ/mol) with respect to the borylation step.

**Figure 3:**
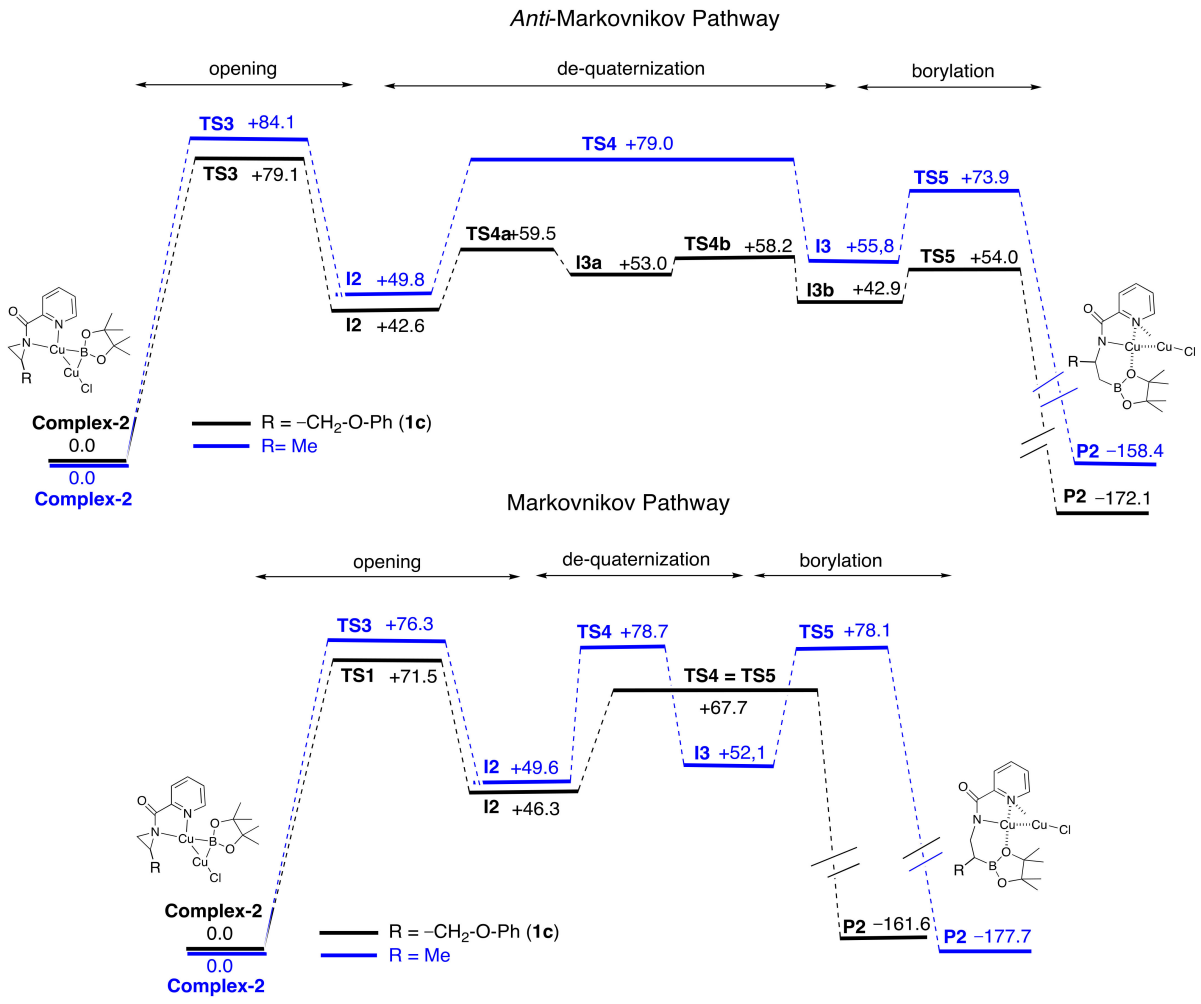
Reaction energy profile with the second model for the reaction of *N*-(2-picolinoyl)-methyl aziridine and **1c** (the ΔG values are in kJ/mol).

**Figure 4:**
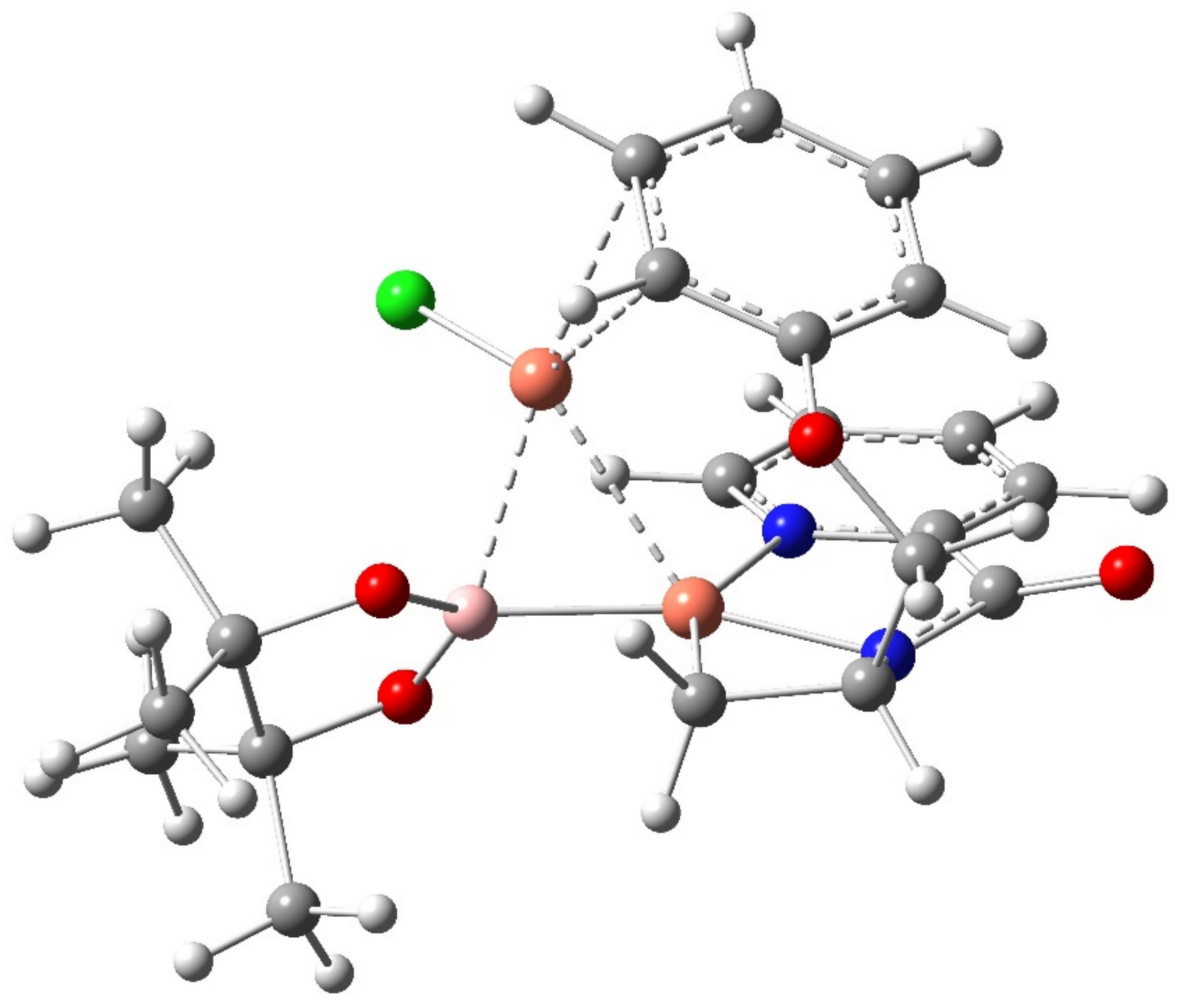
Structure of intermediate **I3a** for compound **1c** with highlighted the η^6^- coordination of the phenyl ring with the copper atom (orange).

Then, using the second model we evaluated the effect of the nature of the lateral chain on the energy profile of the reaction for the two model compounds i.e., *N*-(2-picolinoyl)-methyl aziridine and aziridine **1c** (Figure 3). Once again, the rate-limiting step of the process was the aziridine ring opening (**TS3**), and the regiochemistry was unaltered by the presence of a second copper atom: the opening process was in favor of the Markovnikov product both for the methyl aziridine (ΔΔG^‡^ = 7.8 kJ/mol) and for **1c** (ΔΔG^‡^ = 7.6 kJ/mol). Interestingly, a significative difference was found on the boron de-quaternization step: The phenyl ring in **1c** helped the process through a η^6^-type coordination to the second copper atom (Figure 4), which was more efficient for the *anti*-Markovnikov product (TS4: ΔG = +59.5 kJ/mol for **1c** vs. ΔG = +79.0 kJ/mol for methyl aziridine) than for the Markovnikov one (**TS4**: ΔG = +67.7 kJ/mol for **1c** vs. +78.7 kJ/mol for methyl aziridine). Moreover, for **1c**, the Markovnikov product pathway was characterized by a single de-quaternization/borylation step, while for the *anti*-Markovnikov one the boron de-quaternization process split in two distinct steps. The last aspect to consider for the rationalization of lateral chain effect was the possible role of the phenoxy oxygen atom in **1c** in the coordination of the metal ions. The only **TS3** geometry involved in the opening process, in which the phenoxy oxygen atom is able to coordinate the metal (found for the Markovnikov path), possessed a higher energy (9.2 kJ/mol) with respect to the one for the η^6^-interaction, which then represented the most stable binding mode [34,35].

At this point of the mechanism rationalization, the second dual copper model succeeded in explaining the role of the phenyl ring in substrate **1c**, but we still observed that the aziridine ring-opening step exceeded in energy all the rest of the pathway. This aspect represented a strong divergence from the experimental observation that the aliphatic aziridines (**4c**–**9c**) were reactive, but without borylation outcome. Interestingly, considering the remarkable change in relative energies observed between opening and borylation steps once the CuCl molecule was introduced to the aziridine-copper complex, we decided to modify the initial borylation agent by introducing a solvent molecule (diethyl ether) in place of the chloride counterion (**Complex-3**). The presence in the model of the diethyl ether molecule is required in order to make the de-quaternization process possible, thanks to the copper-oxygen interaction.

**Figure 5:**
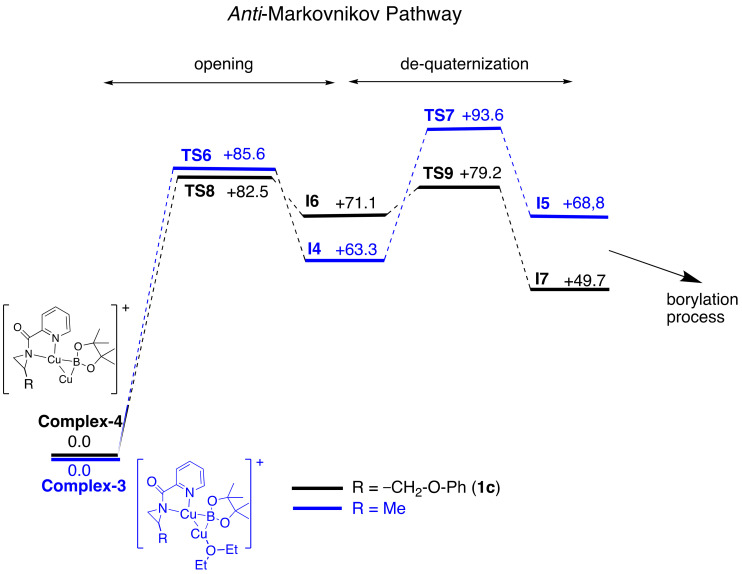
Particular of the reaction energy profile comparison of the *anti*-Markovnikov pathway between **1c** (without the Et_2_O molecule; black) and *N*-2-picolinoyl-methyl aziridine (with the Et_2_O molecule; blue). The ΔG values are in kJ/mol.

The results obtained for the less computing-demanding *N*-(2-picolinoyl)-methyl aziridine showed a remarkable change in relative energy between the two key moments of the process as shown in Figure 5 (blue) only for the *anti*-Markovnikov product (for the Markovnikov path see Figure S1, Page S6 of Supporting Information). The coordination network changes around the second copper atom (diethyl ether instead of chloride anion) did not alter significantly the regioselectivity of both the aziridine opening step (**TS6**, in favor of the Markovnikov product ΔΔG^‡^ = 8.4 kJ/mol) and the boron de-quaternization process (**TS7**, in favor of the *anti*-Markovnikov product ΔΔG^‡^ = 8 kJ/mol), with also a similar activation energy for the ring opening step with respect to the chloride anion (Figure 3, compare **TS3** and **TS6**: +1.5 kJ/mol for *anti*-Markovnikov opening and +0.9 kJ/mol for the Markovnikov one). Interestingly, a remarkable change was observed on the boron de-quaternization **TS7**, which was higher in energy with respect to the TS of the ring opening step (**TS6**) of about +8.0 kJ/mol for the *anti*-Markovnikov pathway and +20.8 kJ/mol for the Markovnikov one, in line with the observed non-borylative reactivity of the fully aliphatic aziridines (**4c**–**9c**).

This last result highlighted the remarkable influence of the copper counterion on the relative energies of the two main TSs of opening and de-quaternization, without changing the absolute activation energy of the ring-opening step.

Lastly, a final comparison between the *anti*-Markovnikov pathway of aziridine **1c** (without Et_2_O and chloride counterion, **Complex-4**, (Figure 5, black) and the *N*-(2-picolinoyl)-methyl aziridine (with the Et_2_O molecule, **Complex-3**) gave us some final remarks. For compound **1c**, the ring-opening process was almost unaltered with respect to the calculation performed with the chloride counterion (compare **TS3** and **TS8**, +3.4 kJ/mol), while the boron de-quaternization step was lower in energy (compare **TS8** and **TS9**, ΔΔG^‡^ = 3.3 kJ/mol) with respect to the former one, despite the absence of the stabilizing effect of a counterion and of an oxygenated solvent molecule (Et_2_O). In comparison with the *anti*-Markovnikov pathway of the 2-methyl aziridine, this last aspect was of primary importance and perfectly reflected the experimental behavior observed. For aziridine **1c** the borylation step is energetically favored, while for aliphatic aziridines, the borylation is the rate determinant step; in fact, these aziridines opened but without a successful borylation. Moreover, the phenyl ring coordination to the copper atom stabilized the boron de-quaternization process (interaction absent in the case of the aliphatic aziridine) in a way that the borylation outcome, in particular the *anti*-Markovnikov one, resulted the favored pathway with respect to any other possible ones.

In addition the corresponding profile calculated for the Markovnikov product (not shown) in the case of aziridine **1c**, without solvent and counterion, revealed a ring-opening process higher in energy +85.2 kJ/mol (+2.7 kJ/mol with respect the *anti*-Markovnikov opening) and with a TS9 around +120 kJ/mol, because of the lower stabilizing effect of the phenyl ring observed in the Markovnikov pathway, especially for the de-quaternization step.

This final model fitted well with the strong dependency of this reaction from the solvent and the copper counterion, and it also depicted particularly well the role of the phenyl ring coordinative stabilization on the borylation process even in terms of regioselectivity. This model, involving the quaternization of the boron atom by two copper atoms, can be reasonably extended for the rationalization of the alkyl epoxides borylation catalyzed by CuI [19].

## 3. Materials and Methods

All reagents were purchased from commercially available sources. THF and toluene were distilled on sodium/benzophenone ketyl. Solvents for extraction and chromatography were distilled before use. Analytical thin layer chromatography (TLC) was performed on silica gel on TLC Al foils (Sigma-Aldrich, St. Louis, MO, USA) with detection by exposure to ultraviolet light (254 nm) and/or by immersion in an acidic staining solution of *p*-anisaldehyde in EtOH. Merck (Kenilworth, NJ, USA) silica gel 60 (230–400 mesh) was used for flash chromatography. Semipreparative TLC was performed on Merck preparative-layer chromatography (PLC) silica gel 60. The ^1^H NMR spectra were recorded on Bruker Avance II at 250 or 400 MHz spectrometer. Chemical shifts are reported in ppm downfield from tetramethylsilane, with the solvent resonance as the internal standard (deuterochloroform: δ 7.26). Signal patterns are indicated as follows: br s, broad singlet; s, singlet; d, doublet; t, triplet; q, quartet; m, multiplet. Coupling constants (*J*) are given in hertz (Hz). Chemical shifts are reported in ppm downfield from tetramethylsilane with the solvent resonance as the internal standard (deuterochloroform: δ 77.16). High-resolution electron spray ionization mass spectrometry (HRESIMS) was acquired in positive ion mode with Orbitrap high-resolution mass spectrometer (Thermo, San Jose, CA, USA), equipped with H-ESI source.

### 3.1. Experimental Part

#### 3.1.1. General Procedure for Borylative Ring-Opening (Table 1)

A dried 10 mL Schlenk tube was charged with B_2_pin_2_ (2.0 equiv), CuCl (0.2 equiv), freshly sublimed t-BuOK (2.0 equiv), corresponding aziridine (1.0 equiv) and, in this case, phosphine (0.2 equiv.). The vessel was further dried under vacuum and backfilled with argon (three cycles), then freshly distilled THF (0.2 M) was added, and reaction mixture was left to react until no aziridine was detected by TLC. After consumption of the starting material, NH_4_Cl sat. was added and an aqueous layer was extracted three times with AcOEt. The collected organic layers were dried with Na_2_SO_4_, solvent was evaporated and the crude was purified by flash chromatography.

#### 3.1.2. *N*-(1-Phenoxy-3-(4,4,5,5-tetramethyl-1,3,2-dioxaborolan-2-yl)propan-2-yl)picolinamide (**2c**)

According to general procedure, a dried 10 mL Schlenk tube was charged with B_2_pin_2_ (76.2 mg, 0.3 mmol), CuCl (3.0 mg, 0.03 mmol), freshly sublimed t-BuOK (33.6 mg, 0.3 mmol), corresponding aziridine (38.1 mg, 0.15 mmol) and THF (0.75 mL). After standard workup, flash chromatography (hexane/AcOEt 7:3 + 3% Et_3_N) afforded the title compound (29.5 mg, 60%) as colorless oil. ^1^H NMR (250 MHz, CDCl_3_) δ 8.21 (d, *J* = 7.8 Hz, 1H), 7.85 (td, *J* = 1.5 Hz, 7.5 Hz, 1H), 7.48–7.39 (m, 2H), 7.33–7.24 (m, 2H), 7.00–6.91 (m, 3H), 4.65–4.51 (m, 1H), 4.15–4.00 (m, 2H), 1.45 (d, *J* = 6.8 Hz, 2H), 1.26 (s, 9H). ^13^C NMR (63 MHz, CDCl_3_) δ 163.9, 158.8, 148.1, 137.5, 129.6, 126.3, 122.4, 121.1, 114.7, 83.6, 70.6, 44.8, 25.1, 25.0, 24.9, 24.7, 24.6, 17.8. ^11^B NMR (128 MHz, CDCl_3_) δ 30.74. [M + Na]^+^ found = 405.1968, C_21_H_27_BN_2_O_4_Na + requires 405.1956.

#### 3.1.3. 4-(Phenoxymethyl)-2-(pyridin-2-yl)-4,5-dihydrooxazole (**3c**)

According to procedure D, a dried 10 mL Schlenk tube was charged with B_2_pin_2_ (76.2 mg, 0.3 mmol), CuCl (3.0 mg, 0.03 mmol), freshly sublimed *t*-BuOK (33.6 mg, 0.3 mmol), BINAP (37.4 mg, 0.06 mmol), corresponding aziridine (38.1 mg, 0.15 mmol) and THF (0.75 mL). After standard workup, flash chromatography (hexane/AcOEt 7:3 + 3% Et_3_N) afforded the title compound as colorless oil. ^1^H NMR (250 MHz, CDCl_3_) δ 8.73 (d, *J* = 4.2 Hz, 1H), 8.06 (d, *J* = 7.8 Hz, 1H), 7.80 (td, *J* = 7.8, 1.7 Hz, 1H), 7.47–7.39 (m, 1H), 7.34–7.21 (m, 5H), 7.01–6.86 (m, 5H), 4.86–4.65 (m, 2H), 4.54 (t, *J* = 8.2, 7.3 Hz, 1H), 4.33 (dd, *J* = 9.3, 4.0 Hz, 1H), 4.03 (dd, *J* = 9.3, 7.1 Hz, 1H). ^13^C NMR (63 MHz, CDCl_3_) δ 164.6, 158.7, 150.0, 146.6, 136.8, 129.6, 126.0, 124.2, 121.3, 114.7, 71.6, 69.7, 66.3. [M + Na]^+^ found = 277.0958, C_15_H_14_N_2_O_2_Na^+^ requires 277.0947.

#### 3.1.4. *N*-(4-Phenyl-1-(4,4,5,5-tetramethyl-1,3,2-dioxaborolan-2-yl)butan-2-yl)picolinamide (**11**)

According to procedure D, a dried 10 mL Schlenk tube was charged with B_2_pin_2_ (114.3 mg, 0.45 mmol), CuCl (3.0 mg, 0.03 mmol), freshly sublimed t-BuOK (33.6 mg, 0.3 mmol), corresponding aziridine (38.0 mg, 0.15 mmol) and THF (0.75 mL). After standard workup, preparative TLC (hexane/AcOEt 9:1 + 2% Et_3_N, 4 runs; Rf = 0.28) afforded the title compound (6.8 mg, 12%) as colorless oil. ^1^H NMR (400 MHz, CDCl_3_) δ 8.55 (dt, *J* = 4.8, 1.8, 0.9 Hz, 1H), 8.33 (d, *J* = 9.5 Hz, 1H), 8.21 (dt, *J* = 7.8, 1.1 Hz, 1H), 7.84 (td, *J* = 7.7, 1.7 Hz, 1H), 7.40 (ddd, *J* = 7.6, 4.8, 1.3 Hz, 1H), 7.30–7.12 (m, 5H), 4.49–4.37 (m, 1H), 2.70 (t, *J* = 9.6, 7.0 Hz, 2H), 1.99–1.90 (m, 2H), 1.27 (d, *J* = 8.0 Hz, 1H), 1.23 (s, 12H). [M + Na]^+^ found = 405.1968, C_21_H_27_BN_2_O_4_Na^+^ requires 405.1956.

#### 3.1.5. *N*-(4-Phenylbutan-2-yl)picolinamide (**12**)

From previously described preparative TLC, the title compound was also separated (Rf = 0.45) as a colorless oil (2.9 mg, 8%). ^1^H NMR (400 MHz, CDCl_3_) δ 8.54 (dt, *J* = 4.8, 1.8, 0.9 Hz, 1H), 7.92 (d, *J* = 9.0 Hz, 1H), 7.85 (td, *J* = 7.7, 1.7 Hz, 1H), 7.43 (ddd, *J* = 7.6, 4.8, 1.3 Hz, 1H), 7.32–7.13 (m, 5H), 4.32–4.20 (m, 1H), 2.71 (t, *J* = 9.0, 7.2 Hz, 2H), 1.97–1.86 (m, 2H), 1.31 (d, *J* = 6.7 Hz, 3H). [M + Na]^+^ found = 277.1318, C_16_H_18_N_2_ONa^+^ requires 277.1311.

The synthesis of protected aziridines is reported in the Supporting Information.

### 3.2. Computational Section

Optimization of all structures was carried out with the Gaussian 16 package [36] at the DFT level [37], employing the functional B3LYP-D3 (damping function: S6 = 1.000; SR6 = 1.2610; S8 = 1.039). [38] and 6 − 31 + G(d) basis set for C, H, B, N, O, S and Cl atoms and SDD basis set (Stuttgart RSC 1997 ECP) for Cu [39]. Berny analytical gradient optimization routines were applied for optimization of the minima on the PES [40]. Analytical frequency calculations (T = 298.15 K and 1 atm pressure) were performed in order to verify that Transition States or minima had one or no imaginary frequency, respectively. Several conformers were investigated, and only the less energetic ones were considered here. IRC calculation was also performed in order to verify whether we had indeed found the desired TS structure. Free Energies, obtained by frequency calculation, were corrected using a single point energy obtained with the larger basis set 6-311+G(2d,p) on C, H, B, N, O and Cl atoms and with solvent (THF) simulation by PCM model [41]. No appreciable improvement were found by optimization and frequency calculation with the most CPU expensive basis set 6-311+G(2d,p). See Table S2, Page S7, Supporting Information.

## 4. Conclusions

To sum up, we have shown the difficulties associated with the development of a general protocol to open the strained ring of protected alkyl aziridines with a nucleophilic boron reagent to give β-aminoboronates. We have now individuated precise reaction parameters (copper salts, protecting group, base and substrates) that can promote the direct borylative ring-opening pathway. The experimental data have also been rationalized theoretically by computational calculation, laying for the first time the foundation for further discoveries in the nucleophilic borylative ring-opening of strained heterocycles.

## Figures and Tables

**Table 1:**
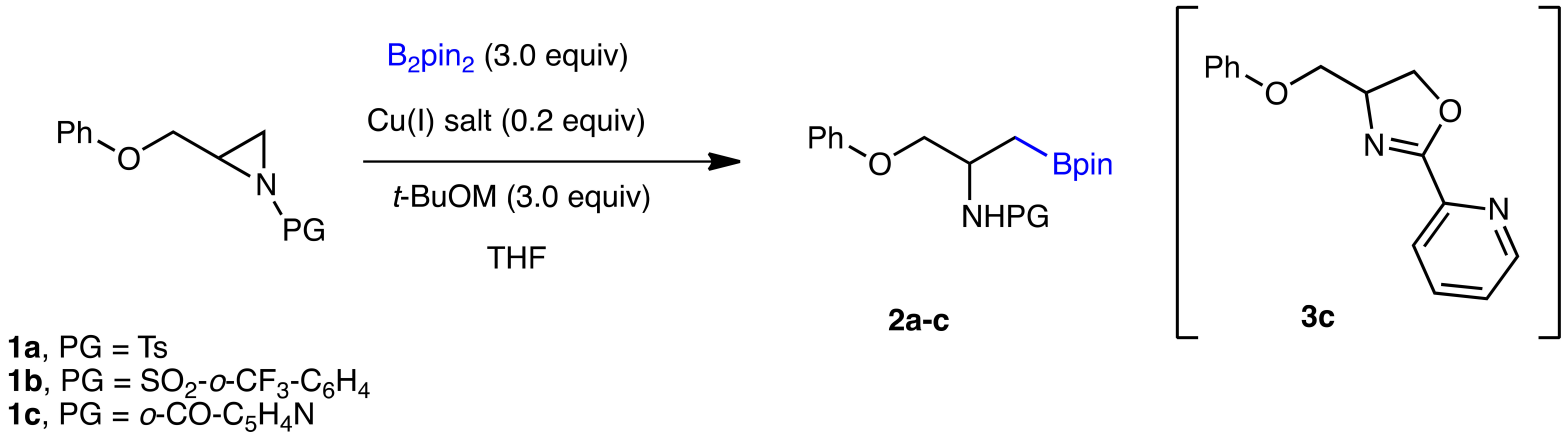
Results of the copper-catalyzed borylative ring opening of differently protected aziridines 1**a**–**c** ^a^.

Entry ^a^	1	Cu Salt	M	L	T (h)	Conversion (Yield) ^b^
1	1a	CuI	Li	-	48	<5
2	1b	CuI	Li	-	48	<5
3	1c	CuI	Li	-	48	<5
4	1a	CuI	K	-	48	<5
5	1b	CuI	K	-	48	<5
6	1c	CuI	K	-	20	>99 (25)
7	1c	CuI	Na	Binap	20	82 **2c/3c** = 0.1
8	1c	CuI	K	Binap	20	77 **2c/3c** = 1.3
9	1c	CuI	Li	Binap	20	73 **2c/3c** = 0.3
10	1c	CuI	K	PPh_3_	20	66 **2c/3c** = 0.8
11	1c	CuI	K	Xantphos	20	28 **2c/3c** = 0.3
12	1c	CuI	K	dppb	20	37 **2c/3c** = 0.6
13^c^	1c	CuCl	K	-	2	71 (60)
14 ^c,d^	1c	CuCl	K	-	5	75(65)
15 ^c,d^	1c	CuI	K	-	5	65 (48)
16 ^c,e^	1c	CuCl	K	-	72	<5

^a^ Unless stated otherwise, all reactions were carried out in anhydrous THF at 60 °C using copper salt (0.2 equiv.), 3.0 equiv. of base and 3.0 equiv. of B_2_pin_2_. ^b^ NMR conversion using α-methylnaphthalene as internal standard. Isolated yields of **2c** after chromatographic purification on SiO_2_ in parentheses. ^c^ Reaction carried out at room temperature. ^d^ 2.0 equivalents of *t*-BuOK were used. ^e^ 1.5 equivalents of *t*-BuOK were used.

**Table 2:** Free energy for the variously N-protected non-substituted aziridine using the model in Figure 1 (see below).

	*N*-Picolinoyl ΔG (kJ/mol)	*N*-Ms ΔG (kJ/mol)	*N*-Ts ΔG (kJ/mol)
Complex-1 ^a^	0.0	0.0	0.0
TS1	+79.0	+95.3	+93.9
I1	−19.1	+46.9	+37.9
TS2	−9.7	+51.8	/
P1 ^b^	−211.1	−138.8	/

^a^ The zero value is represented by the aziridine *N*-complex with Cu and not by the isolated reagents. ^b^ the final product is the one obtained by IRC, so we cannot exclude lower energy conformers.

**Table 3:** Free energy for the regioselectivity of *N*-(2-picolinoyl)-methyl aziridine and **1c** using the model in Scheme 4.

	*N*-(2-Picolinoyl)-methyl Aziridine		Aziridine 1c	
	ΔG (kJ/mol)	ΔG (kJ/mol)	ΔG (kJ/mol)	ΔG (kJ/mol)
	*Anti*-Markovnikov	Markovnikov	*Anti*-Markovnikov	Markovnikov
**Complex-1**	0.0	0.0	0.0	0.0
**TS1**	+78.7	+75.2	+78.7	+77.2
**I1**	−10.2	−7.2	−17.6	−20.3
**TS2**	+4.8	+8.3	−1.4	+8.4
**P1**	−202.0	−189.3	−221.4	−211.4

**Table 4:** Free energies of the second model calculation on the non-substituted *N*-(2-piconiloyl) aziridine, Figure 2 (black).

	ΔG (kJ/mol)
**Complex-2**	0.0 *
**TS3**	+82.6
**I2**	+43.2
**TS4**	+69.0
**I3**	+42.6
**TS5**	+56.5
**P2**	−204.7

* The zero value is represented by the aziridine *N*-complex with Cu and not by the isolated reagents.

## Supplementary Materials

The following are available online. Synthesis of aziridines **1a–c**, **4c–9c**, **10** [42,43,44,45,46,47,48,49,50,51,52,53,54,55,56], Scheme S1: Synthetic pathway for the synthesis of aziridines, Table S1: Two-Copper model, substitution of chloride with alkoxide anion: comparison of activation Free Energies corresponding to TS3 and TS4 from *N*-(2-picolinoyl)-methyl aziridine. Figure S1: Particular of the reaction energy profile comparison between the anti-Markovnikov and Markovnikov pathways of *N*-2-picolinoyl-methyl aziridine (model with the Et2O molecule), Table S2: One-Copper model, *N*-(2-picolinoyl)-methyl aziridine: comparison between Free Energies obtained at different theory level. Cartesian Coordinates and Thermochemical data of the optimized structures. Copies of 1H and 13C NMR of all new products and further computational details are provided as Supplementary Materials.

## Sample Availability

No samples are available.

## References

[1] Sabir S., Kumar G., Verma V.P., Jat J.L. (2018). Aziridine Ring Opening: An Overview of Sustainable Methods. ChemistrySelect.

[2] Nonn M., Remete A.M., Fülöp F., Kiss L. (2017). Recent advances in the transformations of cycloalkane-fused oxiranes and aziridines. Tetrahedron.

[3] Pineschi M. (2006). Asymmetric Ring-Opening of Epoxides and Aziridines with Carbon Nucleophiles. Eur. J. Org. Chem..

[4] Huang C.-Y., Doyle A.G. (2014). The Chemistry of Transition Metals with Three-Membered Ring Heterocycles. Chem. Rev..

[5] Pineschi M. (2014). Advances in the Ring Opening of Small-Ring Heterocycles with Organoboron Derivatives. Synlett.

[6] Pineschi M., Boldrini C., Fernández E. (2020). Advances in Organoboron Chemistry towards Organic Synthesis.

[7] Pineschi M. (2020). The Binomial Copper-Catalysis and Asymmetric Ring Opening of Strained Heterocycles: Past and Future Challenges. Eur. J. Org. Chem..

[8] Pineschi M. (2021). Boron Reagents and Catalysts for the Functionalization of Strained Heterocycles. Adv. Synth. Cat..

[9] Fyfe J.W.B., Watson A.J.B. (2017). Recent Developments in Organoboron Chemistry: Old Dogs, New Tricks. Chem.

[10] Silva M.P., Saraiva L., Pinoto M., Sousa M.E. (2020). Boronic Acids and Their Derivatives in Medicinal Chemistry: Synthesis and Biological Applications. Molecules.

[11] Crotti S., Bertolini F., Macchia F., Pineschi M. (2009). Nickel-Catalyzed Borylative Ring-Opening of Vinyl Epoxides and Aziridines. Org. Lett..

[12] Sebelius S., Olsson V.J., Szabó K.J. (2005). Palladium Pincer Complex Catalyzed Substitution of Vinyl Cyclopropanes, Vinyl Aziridines, and Allyl Acetates with Tetrahydroxydiboron. An Efficient Route to Functionalized Allylboronic Acids and Potassium Trifluoro(allyl)borates. J. Am. Chem. Soc..

[13] Zhao J., Szabó K.J. (2016). Catalytic Borylative Opening of Propargyl Cyclopropane, Epoxide, Aziridine, and Oxetane Substrates: Ligand Controlled Synthesis of Allenyl Boronates and Alkenyl Diboronates. Angew. Chem. Int. Ed..

[14] Takeda Y., Kuroda A., Sameera W.M.C., Morokuma K., Minakata S. (2016). Palladium-catalyzed regioselective and stereo-invertive ring-opening borylation of 2-arylaziridines with bis(pinacolato)diboron: Experimental and computational studies. Chem. Sci..

[15] Takeda Y., Sameera W.M.C., Minakata S. (2020). Palladium-Catalyzed Regioselective and Stereospecific Ring-Opening Cross-Coupling of Aziridines: Experimental and Computational Studies. Acc. Chem. Res..

[16] Šterman A., Sosic I., Gobec S., Casar Z. (2019). Synthesis of aminoboronic acid derivatives: An update on recent advances. Org. Chem. Front..

[17] DeGorovoy A.S., Gozhina O.V., Svendsen J.S., Domorad A.A., Tetz G.V., Tetz V.V., Lejon T. (2013). Boron-Containing Peptidomimetics — A Novel Class of Selective Anti-tubercular Drugs. Chem. Biol. Drug. Des..

[18] Li X., Hall D.G. (2021). Synthesis and Applications of β-aminoalkylboronic Acid Derivatives. Adv. Synth. Cat..

[19] Ahmed E.-A.M.A., Lu X., Gong T.-J., Zhang Z.-Q., Xiao B., Fu Y. (2017). Copper-catalyzed/mediated borylation reactions of epoxides with diboron reagents: Access to β-hydroxyl boronic esters. Chem. Commun..

[20] Martin A., Casto K., Morris W., Morgan J.B. (2011). Phosphine-Catalyzed Heine Reaction. Org. Lett..

[21] Heine H.W., King D.C., Portland L.A. (1966). Aziridines XII. The isomerization of Some *cis*- and *trans*-1-p-nitrobenzoyl-2,3-Substituted Aziridines. J. Org. Chem..

[22] Sweeney J.B. (2002). Aziridines: Epoxides’ ugly cousin?. Chem. Soc. Rev..

[23] Stankovic S., D’hooghe M., Catak S., Eum H., Waroquier M., Van Speybroeck V., De Kimpe N., Ha J.-H. (2012). Regioselectivity in the ring opening of non-activated aziridines. Chem. Soc. Rev..

[24] Di Bussolo V., Checchia L., Romano M.R., Favero L., Pineschi M., Crotti P. (2010). Aminolysis of glycal-derived allylic epoxides and activated aziridines. Effects of the absence of coordination processes on the regio- and stereoselectivity. Tetrahedron.

[25] The anti-periplanar ring opening (in line with the Takeda model) of the not substituted mesyl-aziridine and picolinoyl-aziridine with the THF-Cu-Bpin adduct were investigated. The result shown a ΔΔG‡ of 3 kJ/mol in favour of the sulfonyl aziridine with respect to the N-2(picolinoyl) one in contradiction with the experimental evidence in which the formers are inert while the latter are reactive. See also Supporting Information, pp. S7–S10.

[26] Ney J.E., Wolfe J.P. (2006). Synthesis and Reactivity of Azapalladacyclobutanes. J. Am. Chem. Soc..

[27] Lin B.L., Clough C.R., Hillhouse G.L. (2002). Interactions of Aziridines with Nickel Complexes: Oxidative-Addition and Reductive-Elimination Reactions that Break and Make C–N Bonds. J. Am. Chem. Soc..

[28] Han L., Xu B., Liu T. (2018). Mechanisms of the synthesis of trialkylsubstituted alkenylboronates from unactivated internal alkynes catalyzed by copper: A theoretical study. J. Org. Chem..

[29] Kajiwara T., Terabayashi T., Yamashita M., Nozaki K. (2008). Syntheses, Structures, and Reactivities of Borylcopper and -zinc Compounds: 1,4-Silaboration of an α,β-Unsaturated Ketone to Form a γ-Siloxyallylborane. Angew. Chem. Int. Ed..

[30] Wyss C.M., Bitting J., Bacsa J., Gray T.G., Sadighi J.P. (2016). Bonding and Reactivity of a Dicopper(I) μ-Boryl Cation. Organometallics..

[31] Borner C., Anders L., Brandhorst K., Kleeberg C. (2017). Elusive Phosphine Copper(I) Boryl Complexes: Synthesis, Structures, and Reactivity. Organometallics..

[32] Drescher W., Kleeberg C. (2019). Terminal versus Bridging Boryl Coordination in N-Heterocyclic Carbene Copper(I) Boryl Complexes: Syntheses, Structures, and Dynamic Behavior. Inorg. Chem..

[33] The substitution of the chloride anion with methoxide or tert-butoxide did not determined a significative difference in activation free energy with respect to the chloride one. In particular, we observed a modest decrease of both activation free energies of **TS3** and **TS4** from chloride towards methoxide/tert-butoxide, and a slight increase of the relative energy between the two steps (slightly bigger for tert-butoxide). This small increment of the relative energy between the **TS3** and **TS4** is most likely due to the contribution of the dispersion force between the methyl groups of the alkoxide and the pyridine ring in **TS4**. For more information see Table S1, pp. S6, Supporting Information.

[34] The TS with the shortest PhO-Cu distance (2.87 Å, **TS3-B** see supplementary material, Figure S2) can be found in the pathway towards the Markovnikov product: Also for the relative intermediates the energy remained particularly high (7.9 kJ/mol) even with a shorter O-Cu distance (2.3 Å). See pp. S133–S136, Supporting Information.

[35] We have also evaluated the possibility that CuCl can stabilize an anti-ring opening for the anti-Markovnikov product; the complex relative to the anti-transition state in which the aziridine nitrogen atom was coordinated to CuCl and the Cu-Bpin was coordinated to the phenoxy oxygen atom possessed a higher energy with respect to **Complex-2** (ΔΔG = +74.9 kJ/mol). Although the anti-opening possessed a lower ΔG‡ (+41.7 kJ/mol) with respect to the one of the syn opening (+84.1 kJ/mol), the difference between the TS of anti-opening with respect to the syn one was remarkably in favor of the latter (37.4 kJ/mol). Please see pp. S136–S142, Supporting Information.

[36] Frisch M.J., Trucks G.W., Schlegel H.B., Scuseria G.E., Robb M.A., Cheeseman J.R., Scalmani G., Barone V., Petersson G.A., Nakatsuji H. (2016). Gaussian 16, Revision A.03.

[37] Ziegler T. (1991). Approximate density functional theory as a practical tool in molecular energetics and dynamics. Chem. Rev..

[38] Grimme S., Antony J., Ehrlich S., Krieg H.J. (2010). A Consistent and accurate ab initio parametrization of density functional dispersion correction (DFT-D) for 94 elements H-Pu. Chem. Phys..

[39] Schuchardt K.L., Didier B.T., Elsethagen T., Sun L., Gurumoorthi V., Chase J., Li J., Windus T.L. (2007). Basis Set Exchange: A Community Database for Computational Sciences. J. Chem. Inf. Model..

[40] Schlegel H.B.J. (1982). Optimization of equilibrium geometries and transition structures. Comp. Chem..

[41] Tomasi J., Mennucci B., Cammi R. (2005). Quantum mechanical continuum solvation models. Chem. Rev..

[42] Fukuta Y., Mita T., Fukuda N., Kanai M., Shibasaki M. (2006). De Novo Synthesis of Tamiflu via a Catalytic Asymmetric Ring-Opening of *meso*-Aziridines with TMSN_3_. J. Am. Chem. Soc..

[43] Sommerdijk N.A.J.M., Buynsters P.J.J.A., Akdemir H., Geurts D.G., Nolte R.J.M., Zwanenburg B. (1997). Aziridines as Precursors for Chiral Amide-Containing Surfactants. J. Org. Chem..

[44] Christoffers J., Schulze Y., Pickardt J. (2001). Synthesis, resolution, and absolute configuration of *trans*-1-amino-2-dimethylaminocyclohexane. Tetrahedron.

[45] Namutebi M., McGarrigle E.M., Aggarwal V.K. (2010). Ring-Opening of NH-Aziridines with Thiols in Ionic Liquids: Application to the Synthesis of Aminosulfide Catalysts for Asymmetric Epoxidation of Aldehydes. Phosphorus Sulfur Silicon Relat. Elem..

[46] McLeod D.C., Tsarevsky N.V. (2016). Reversible Deactivation Radical Polymerization of Monomers Containing Activated Aziridine Groups. Macromol. Rapid Commun..

[47] Schrittwieser J.H., Lavandera I., Seisser B., Mautner B., Kroutil W. (2009). Biocatalytic Cascade for the Synthesis of Enantiopure β-Azidoalcohols and β-Hydroxynitriles. Eur. J. Org. Chem..

[48] Namba K., Mera A., Osawa A., Sakuda E., Kitamura N., Tanino K. (2012). One-Pot Synthesis of Highly Fluorescent 2,5-Disubstituted-1,3a,6a-triazapentalene. Org. Lett..

[49] Hanessian S., Del Valle J.R., Xue Y., Blomberg N. (2006). Total Synthesis and Structural Confirmation of Chlorodysinosin A. J. Am. Chem. Soc..

[50] Jun Y.C., Borch R.F. (2007). Highly Efficient Synthesis of Enantiomerically Enriched 2-Hydroxymethylaziridines by Enzymatic Desymmetrization. Org. Lett..

[51] Lebel H., Lectard S., Parmentier M. (2007). Copper-Catalyzed Alkene Aziridination with N-Tosyloxycarbamates. Org. Lett..

[52] Buckley B.R., Patel A.P., Wijayantha K.G.U. (2013). Observations on the Modified Wenker Synthesis of Aziridines and the Development of a Biphasic System. J. Org. Chem..

[53] Ankner T., Hilmersson G. (2009). Instantaneous Deprotection of Tosylamides and Esters with SmI_2_/Amine/Water. Org. Lett..

[54] Hayashi M., Shiomi N., Funahashi Y., Nakamura S. (2012). Cinchona Alkaloid Amides/Dialkylzinc Catalyzed Enantioselective Desymmetrization of Aziridines with Phosphites. J. Am. Chem. Soc..

[55] Li J., Liao Y., Zhang Y., Liu X., Lin L., Feng X. (2014). Chiral magnesium(II)-catalyzed asymmetric ring-opening of *meso*-aziridines with primary alcohols. Chem. Commun..

[56] Wang L., Yang D., Han F., Li D., Zhao D., Wang R. (2015). Catalytic Asymmetric Construction of Pyrroloindolines via an in Situ Generated Magnesium Catalyst. Org. Lett..

