# Supplementary material for: Copper-Catalyzed Ring-Opening Reactions of Alkyl Aziridines with B2pin2: Experimental and Computational Studies
Source: Molecules. 2021 Dec 6;26(23):7399. doi: 10.3390/molecules26237399 (PMC8659106; doi:10.3390/molecules26237399)

# SUPPORTING INFORMATION

# Copper-Catalyzed Ring-Opening Reactions of Alkyl Aziridines with $B_2pin_2$: Experimental and Computational Studies

**Lucilla Favero*, Andrea Menichetti, Cosimo Boldrini, Lucrezia Margherita Comparini, Valeria Di Bussolo, Sebastiano Di Pietro and Mauro Pineschi ***

Department of Pharmacy, University of Pisa, Via Bonanno 33, 56126 Pisa, Italy.
*Correspondence: lucilla.favero@unipi.it; Tel.: +39-050-221-9685
* Correspondence: mauro.pineschi@unipi.it; Tel.: +39-050-221-9668

## Contents

General synthetic pathway for substrates formation . . . . . . . . . . S1

Aziridines protection. . . . . . . . . . S1

Picolin-2-yl protection. . . . . . . . . . S2

References. . . . . . . . . . S5

Computational Section. . . . . . . . . . S6

$^{1}$H and $^{13}$C NMR spectra. . . . . . . . . . S176

## General synthetic pathway for substrates formation

Aziridines were synthesized following the synthetic pathway shown in **Scheme S1**. Only aziridine **5c** was formed starting from the corresponding alkene following a literature procedure **A'**.[1]

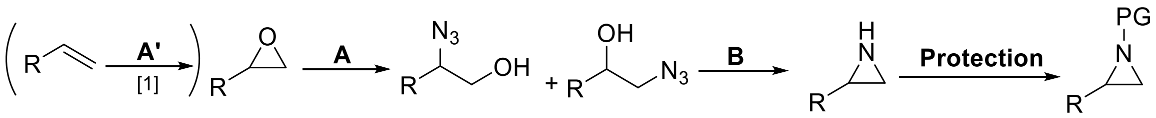


***Scheme S1**. Synthetic pathway for the synthesis of aziridines.*

**A.** A round-bottomed flask was charged with the corresponding epoxide, $NaN_3$, $NH_4Cl$ and solvent, and the mixture was left to stir overnight at reflux. After workup, the crude containing a regioisomeric mixture was used without any further purification. Complete procedures and spectra are described in the literature for each compound.[2–7]

**B.** A round-bottomed flask was charged with the corresponding azido alcohol regioisomeric mixture (1.0 equiv.) in $CH_3CN$ or MTBE, and $PPh_3$ (1.0 equiv.) was portionwise added. When no evolution of nitrogen was noticed, the mixture was warmed up to reflux overnight. Then, the solvent was evaporated, and the solid residue was suspended in a 1:1 mixture of hexane/AcOEt. The residue solid (triphenylphosphine oxide) was filtered away. Complete procedures and spectra are described in the literature for each compounds.[1,3–5,8–11]

## Aziridines protection

**2-(phenoxymethyl)-1-tosylaziridine (1a)**

According to literature procedure,[12] a three-neck round-bottomed flask was charged with corresponding unprotected aziridine (300 mg, 2.0 mmol), dry $Et_2O$ (7 mL), $Et_3N$ (0.38 mL) and 2-(trifluoromethyl)benzenesolfonyl chloride (434 mg, 2.3 mmol, portionwise) under argon. The reaction was left to react until no aziridine was detected by TLC, and cooled at 0°C. Then, 10 mL of water and 10 mL of saturated solution of $NaHCO_3$ were slowly added. The organic layer was separated and the aqueous layer was extracted three times with $Et_2O$ (20 mL). Collected organic layers were washed with brine (15 mg) and dried with $MgSO_4$. The crude was purified by flash chromatography (hexane/AcOEt 8:2) affording the title compound (321.6 mg, 60%) as white solid. $^1H$ NMR (250 MHz, $CDCl_3$) δ 7.92 – 7.76 (m, 2H), 7.38 – 7.27 (m, 2H), 7.26 – 7.16 (m, 2H), 7.01 – 6.87 (m, 1H), 6.80 – 6.68 (m, 2H), 4.05 (dd, J = 10.8, 4.4 Hz, 1H), 3.92 (dd, J = 10.8, 6.0 Hz, 1H), 3.21 – 3.10 (m, 1H), 2.80 (d, J = 7.1 Hz, 1H), 2.45 (s, 3H), 2.35 (d, J = 4.4 Hz, 1H). $^{13}C$ NMR (63 MHz, $CDCl_3$) δ ppm: 158.1, 144.8, 129.8, 129.6, 128.2, 121.4, 114.6, 67.1, 38.3, 31.2, 21.8. $[M + Na]^+$ found = 326.0825, $C_{16}H_{17}NO_3SNa^+$ requires 326.0821.

**2-(phenoxymethyl)-1-((2-(trifluoromethyl)phenyl)sulfonyl)aziridine (1b)**

According to literature procedure,[12] a three-neck round-bottomed flask was charged with corresponding unprotected aziridine (300 mg, 2.0 mmol), dry $Et_2O$ (7 mL), $Et_3N$ (0.38 mL) and 2-(trifluoromethyl)benzenesolfonyl chloride (580 mg, 2.3 mmol, portionwise) under argon. The reaction was left to react until no aziridine was detected by TLC, and cooled at 0°C. Then, 10 mL of water and 10 mL of saturated solution of $NaHCO_3$ were slowly added. The organic layer was separated and the aqueous layer was extracted three times with $Et_2O$ (20 mL). Collected organic layers were washed with brine (15 mg) and dried with $MgSO_4$. The crude was purified by flash chromatography (hexane/AcOEt 8:2) affording the title compound (321.6 mg, 45%) as white solid. $^1H$ NMR (250 MHz, $CDCl_3$) δ 8.42 – 8.30 (m, 1H), 8.01 – 7.93 (m, 1H), 7.87 – 7.75 (m, 2H), 7.35 – 7.25 (m, 2H), 7.05 – 6.96 (m, 1H), 6.87 – 6.77 (m, 2H), 4.26 – 4.01 (m, 2H), 3.47 – 3.36 (m, 1H), 3.05 (d, *J* = 7.2 Hz, 1H), 2.55 (d, *J* = 4.6 Hz, 1H).$^{13}C$ NMR (63 MHz, $CDCl_3$) δ 158.1, 133.7, 132.4, 132.2, 129.6, 128.44 (q, *J* = 6.3 Hz), 124.7,121.5, 120.3, 114.6, 66.9, 39.4, 32.4. $[M + Na]^+$ found = 380.0542, $C_{16}H_{14}F_3NO_3SNa^+$ requires 380.0539.

## Picolin-2-yl protection

**General procedure C.** A round-bottomed flask was charged with 2-picolinic acid (1.05 equiv.), DCC (1.1 equiv.), DMAP (0.2 equiv.) and freshly distilled DCM (8 mL per equivalent of aziridine). Then, a solution of corresponding aziridine (1.0 equiv., DCM 1 M) was slowly added. The reaction mixture was left to vigorously stir until no aziridine was detected by TLC, and the reaction mixture was filtered on celite. After evaporation of the solvent, the crude was purified by flash chromatography.[13]

**(2-(phenoxymethyl)aziridin-1-yl)(pyridin-2-yl)methanone (1c)**

According to general procedure C, a round-bottomed flask was charged with 2-picolinic acid (264 mg, 2.1 mmol), DCC (454 mg, 2.2 mmol), DMAP (24.4 mg, 0.2 mmol), DCM (8 mL), and corresponding unprotected aziridine (300 mg, 2.0 mmol in 2 mL of DCM). After standard workup, the crude was purified by flash chromatography (hexanes/AcOEt 7:3) affording the title compound (280 mg, 55%) as waxy yellow solid. m.p. = 55-56°C. $^1H$ NMR (250 MHz, $CDCl_3$) δ 8.67 (d, J = 4.7 Hz, 1H), 8.11 (d, J = 7.9 Hz, 1H), 7.77 (dd, J = 7.7, 1.8 Hz, 1H), 7.41 (ddd, J = 7.6, 4.7, 1.3 Hz, 1H), 7.28 – 7.18 (m, 2H), 6.96 – 6.84 (m, 3H), 4.52 (dd, J = 10.4, 3.8 Hz, 1H), 4.09 (dd, J = 10.5, 5.7 Hz, 1H), 3.17 – 3.09 (m, 1H), 2.66 (d, J = 5.6 Hz, 1H), 2.57 (d, J = 3.6 Hz, 1H). $^{13}C$ NMR (101 MHz, $CDCl_3$) δ 176.5, 158.4, 150.4, 149.3, 136.9, 129.5, 126.8, 124.2, 121.2, 114.6, 7.6, 36.8, 29.6. $[M + Na]^+$ found = 277.0954, $C_{15}H_{14}N_2O_2Na^+$ requires 277.0947.

**(2-(((tert-butyldimethylsilyl)oxy)methyl)aziridin-1-yl)(pyridin-2-yl)methanone (4c)**

According to procedure C, a round-bottomed flask was charged with 2-picolinic acid (643 mg, 5.2 mmol), DCC (1.07 g, 5.2 mmol), DMAP (39.5 mg, 0.32 mmol), DCM (15 mL), and corresponding unprotected aziridine (605 mg, 3.2 mmol in 3.2 mL of DCM). After standard workup, the crude was purified by flash chromatography (petroleum ether/AcOEt 9:1 + 3% $Et_3N$) affording the title compound (191 mg, 20%) as white solid. $^1H$ NMR (250 MHz, $CDCl_3$) δ 8.75 – 8.64 (m, 1H), 8.19 – 8.12 (m, 1H), 7.81 (td, J = 7.7, 1.8 Hz, 1H), 7.43 (ddd, J = 7.6, 4.7,

1.2 Hz, 1H), 4.08 (dd, J = 11.2, 3.0 Hz, 1H), 3.84 (dd, J = 11.2, 4.2 Hz, 1H), 3.02 – 2.93 (m, 1H), 2.54 – 2.46 (m, 2H), 0.82 (s, 9H), -0.01 (s, 3H), -0.10 (s, 3H). $^{13}$C NMR (101 MHz, $CDCl_3$) δ 176.7, 149.3, 148.4, 136.9, 126.5, 124.2, 61.8, 39.9, 27.9, 26.0, 25.9, -5.4, -5.4. $[M + Na]^+$ found = 315.1501, $C_{15}H_{24}N_2O_2SiNa^+$ requires 315.1499.

**(7-azabicyclo[4.1.0]hept-3-en-7-yl)(pyridin-2-yl)methanone (5c)**

A modified procedure was used because of volatility of NH-free aziridine. In a round-bottomed flask, to a solution of corresponding azido alcohol 6-azidocyclohex-3-en-1-ol (409 mg, 2.294 mmol) in MTBE (4 mL, 0.88 M), $PPh_3$ was portionwise added. When no formation of $N_2$ was observed, the reaction mixture was heated up to reflux overnight. Then, the solution was filtered in order to separate triphenylphosphine oxide. Meanwhile, a round-bottomed flask was charged with 2-picolinic acid (380 mg, 3.09 mmol), DCC (667 mg, 2.32 mmol), DMAP (36 mg, 0.29 mmol), DCM (14 mL) and the previously filtered solution was dropwise added. The reaction mixture was left to react overnight, and after standard workup, the crude was purified by flash chromatography (hexanes/$Et_2O$ 5:5 + 2% $Et_3N$) affording the title compound (325 mg, 55%) as white solid. The spectral data are consistent with literature.[14]

**(2-benzylaziridin-1-yl)(pyridin-2-yl)methanone (6c)**

According to procedure C, a round-bottomed flask was charged with 2-picolinic acid (606 mg, 5.52 mmol), DCC (1.2 g, 5.79 mmol), DMAP (63.5 mg, 0.53 mmol), DCM (16 mL), and corresponding unprotected aziridine (700 mg, 5.26 mmol in 4 mL of DCM). After standard workup, the crude was purified by flash chromatography (hexanes/AcOEt 7:3; $R_f$ = 0.45) affording the title compound (400 mg, 35%) as yellow oil. $^1$H NMR (400 MHz, $CDCl_3$) δ 8.74 (d, J = 4.8 Hz, 0H), 8.14 (d, J = 7.9 Hz, 1H), 7.84 (td, J = 7.7, 1.8 Hz, 1H), 7.48 (ddd, J = 7.6, 4.7, 1.3 Hz, 1H), 7.40 – 7.20 (m, 4H), 3.42 (dd, J = 14.3, 4.5 Hz, 1H), 3.06 – 2.93 (m, 1H), 2.80 (dd, J = 14.4, 7.6 Hz, 1H), 2.62 (d, J = 5.6 Hz, 1H), 2.42 (d, J = 3.6 Hz, 1H). $^{13}$C NMR (101 MHz, $CDCl_3$) δ 176.2, 158.2, 150.2, 149.0, 136.7, 129.3, 126.5, 123.9, 121.0, 114.4, 67.3, 36.6, 29.3. $[M + Na]^+$ found = 261.0989, $C_{15}H_{14}N_2ONa^+$ requires 261.0998

**(2-hexylaziridin-1-yl)(pyridin-2-yl)methanone (7c)**

According to procedure C, a round-bottomed flask was charged with 2-picolinic acid (264 mg, 2.1 mmol), DCC (454 mg, 2.2 mmol), DMAP (24.4 mg, 0.2 mmol), DCM (8 mL), and corresponding unprotected aziridine (254.5 mg, 2.0 mmol in 2 mL of DCM). After standard workup, the crude was purified by flash chromatography (hexanes/AcOEt 7:3) affording the title compound (209 mg, 45%) as yellow oil. $^1$H NMR (250 MHz, $CDCl_3$) δ 8.69 (dt, *J* = 4.8, 1.4 Hz, 1H), 8.09 (d, *J* = 7.8 Hz, 1H), 7.81 (td, *J* = 7.8, 1.8 Hz, 1H), 7.42 (ddd, *J* = 7.6, 4.8, 1.2 Hz, 1H), 2.74 – 2.63 (m, 1H), 2.53 (d, *J* = 5.7 Hz, 1H), 2.29 (d, *J* = 3.7 Hz, 1H), 1.95 – 1.81 (m, 1H), 1.54 – 1.23 (m, 9H), 0.86 (t, *J* = 6.5 Hz, 3H). $^{13}$C NMR (63 MHz, $CDCl_3$) δ 177.4, 150.9, 149.4, 136.9, 126.6, 124.1, 38.9, 32.3, 32.0, 31.8, 29.1, 26.8, 22.7, 14.2. $[M + Na]^+$ found = 255.1471, $C_{14}H_{20}N_2ONa^+$ requires 255.1468

**(6-azabicyclo[3.1.0]hexan-6-yl)(pyridin-2-yl)methanone (8c)**

According to procedure C, a round-bottomed flask was charged with 2-picolinic acid (946 mg, 7.6 mmol), DCC (1.79 g, 8.69 mmol), DMAP (88.4 mg, 0.724 mmol), DCM (20 mL), and unprotected aziridine (603 mg, 7.24

mmol in 7.2 mL of DCM). After standard workup, the crude was purified by flash chromatography (Petroleum ether/AcOEt 8:2 + 3% $Et_3N$, R*f* = 0.26) affording the title compound (1.08 g, 79%) as yellowish solid. The spectral data are consistent with literature.[15]

**(7-azabicyclo[4.1.0]heptan-7-yl)(pyridin-2-yl)methanone (9c)**

According to procedure C, a round-bottomed flask was charged with 2-picolinic acid (1.3 g, 10.5 mmol), DCC (2.27 g, 11.0 mmol), DMAP (122.2 mg, 1.0 mmol), DCM (40 mL), and unprotected aziridine (972 mg, 10.0 mmol in 10 mL of DCM). After standard workup, the crude was purified by flash chromatography (Hexane/AcOEt 7:3, R*f* = 0.25) affording the title compound (1.02 g, 50%) as yellowish solid. The spectral data are consistent with literature.[15]

**(2-phenethylaziridin-1-yl)(pyridin-2-yl)methanone (10)**

According to procedure C, a round-bottomed flask was charged with 2-picolinic acid (388 mg, 3.15 mmol), DCC (618 mg, 3.3 mmol), DMAP (36.7 mg, 0.3 mmol), DCM (15 mL), and unprotected aziridine (441 mg, 3.0 mmol in 3 mL of DCM). After standard workup, the crude was purified by flash chromatography (hexane/AcOEt 7:3 + 1% $Et_3N$, R*f* = 0.33) affording the title compound (400 mg, 53%) as yellowish solid. $^1H$ NMR (250 MHz, $CDCl_3$) δ 8.70 (ddd, J = 4.7, 1.8, 0.9 Hz, 1H), 8.10 (dt, J = 7.9, 1.1 Hz, 1H), 7.81 (td, J = 7.7, 1.8 Hz, 1H), 7.43 (ddd, J = 7.6, 4.8, 1.3 Hz, 1H), 7.33 – 7.13 (m, 5H), 2.93 – 2.70 (m, 3H), 2.53 (d, J = 5.8 Hz, 1H), 2.29 (d, J = 3.7 Hz, 1H), 2.27 – 2.13 (m, 1H), 1.90 – 1.73 (m, 1H). $^{13}C$ NMR (63 MHz, $CDCl_3$) δ 177.0, 150.5, 149.1, 141.2, 136.7, 128.3, 126.4, 125.8, 123.8, 38.0, 33.9, 32.9, 31.8. $[M + Na]^+$ found = 275.1159, $C_{16}H_{16}N_2ONa^+$ requires 275.1155

References


1. Fukuta, Y.; Mita, T.; Fukuda, N.; Kanai, M.; Shibasaki, M. De Novo Synthesis of Tamiflu via a Catalytic Asymmetric Ring-Opening of *meso*-Aziridines with $TMSN_3$. *J. Am. Chem. Soc.* 2006, *128*, 6312–6313
2. Sommerdijk, N. A. J. M.; Buynsters, P. J. J. A.; Akdemir, H.; Geurts, D. G.; Nolte, R. J. M.; Zwanenburg, B. Aziridines as Precursors for Chiral Amide-Containing Surfactants. *J. Org. Chem.* 1997, *62*, 4955–4960.
3. Christoffers, J.; Schulze, Y.; Pickardt, J. Synthesis, resolution, and absolute configuration of *trans*-1-amino-2-dimethylaminocyclohexane. *Tetrahedron* 2001, *57*, 1765–1769.
4. Namutebi, M.; McGarrigle, E. M.; Aggarwal, V. K. Ring-Opening of NH-Aziridines with Thiols in Ionic Liquids: Application to the Synthesis of Aminosulfide Catalysts for Asymmetric Epoxidation of Aldehydes. *Phosphorus, Sulfur Silicon Relat. Elem.* 2010, *185*, 1250–1272.
5. McLeod, D. C.; Tsarevsky, N. V. Reversible Deactivation Radical Polymerization of Monomers Containing Activated Aziridine Groups. *Macromol. Rapid Commun.* 2016, *37*, 1694–1700.
6. Schrittwieser, J. H.; Lavandera, I.; Seisser, B.; Mautner, B.; Kroutil, W. Biocatalytic Cascade for the Synthesis of Enantiopure β-Azidoalcohols and β-Hydroxynitriles. *European J. Org. Chem.* 2009, No. 14, 2293–2298.
7. Namba, K.; Mera, A.; Osawa, A.; Sakuda, E.; Kitamura, N.; Tanino, K. One-Pot Synthesis of Highly Fluorescent 2,5-Disubstituted-1,3a,6a-triazapentalene. *Org. Lett.* 2012, *14*, 5554–5557.
8. Hanessian, S.; Del Valle, J. R.; Xue, Y.; Blomberg, N. Total Synthesis and Structural Confirmation of Chlorodysinosin A. *J. Am. Chem. Soc.* 2006, *128*, 10491–10495.
9. Jun, Y. C.; Borch, R. F. Highly Efficient Synthesis of Enantiomerically Enriched 2-Hydroxymethylaziridines by Enzymatic Desymmetrization. *Org. Lett.* 2007, *9*, 215–218.
10. Lebel, H.; Lectard, S.; Parmentier, M. Copper-Catalyzed Alkene Aziridination with *N*-Tosyloxycarbamates. *Org. Lett.* 2007, *9*, 4797–4800.
11. Buckley, B. R.; Patel, A. P.; Wijayantha, K. G. U. Observations on the Modified Wenker Synthesis of Aziridines and the Development of a Biphasic System. *J. Org. Chem.* 2013, *78*, 1289–1292.
12. Ankner, T.; Hilmersson, G. Instantaneous Deprotection of Tosylamides and Esters with $SmI_2$/Amine/Water. *Org. Lett.* 2009, *11*, 503–506.
13. Hayashi, M.; Shiomi, N.; Funahashi, Y.; Nakamura, S. Cinchona Alkaloid Amides/Dialkylzinc Catalyzed Enantioselective Desymmetrization of Aziridines with Phosphites *J. Am. Chem. Soc.* 2012, *134*, 19366–19369.
14. Li, J.; Liao, Y.; Zhang, Y.; Liu, X.; Lin, L.; Feng, X. Chiral magnesium(II)-catalyzed asymmetric ring-opening of *meso*-aziridines with primary alcohols. *Chem. Commun.* 2014, *50*, 6672–6674.
15. Wang, L.; Yang, D.; Han, F.; Li, D.; Zhao, D.; Wang, R. Catalytic Asymmetric Construction of Pyrroloindolines via an in Situ Generated Magnesium Catalyst *Org. Lett.* 2015, *17*, 176–179.

COMPUTATIONAL SECTION

Table S1. Two-Copper model, substitution of chloride with alkoxide anion: comparison of activation Free Energies (kJ/mol) corresponding to **TS3** and **TS4** from *N*-(2-picolinoyl)-methyl aziridine.

| Anion | ΔG‡ (**TS3**) | ΔG‡ (**TS4**) | ΔΔG* (**TS3-TS4**) |
|---|---|---|---|
| $Cl^-$ | +82.6 | +69.0 | 13.6 |
| $MeO^-$ | +74.4 | +58.5 | 15.9 |
| *t*-$BuO^-$ | +76.7 | +58.4 | 18.3 |

[a] (A) see ref. 29, main text, and Thermochemical Data , pag S77-S86.

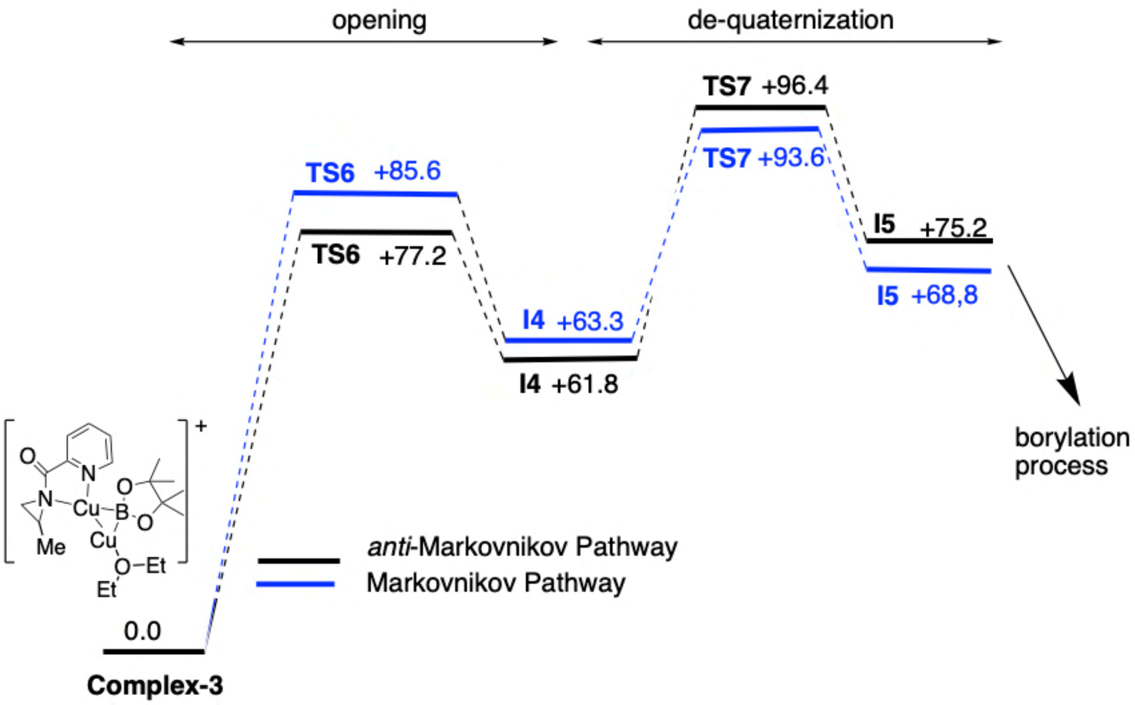


Figure S1. Particular of the reaction energy profile comparison between the *anti*-Markovnikov (blue) and Markovnikov (black) pathways of *N*-(2-picolinoyl)-methyl aziridine (model with the $Et_2O$ molecule). The ΔG values are in kJ/mol.

Table S2. One-Copper model, *N*-(2-picolinoyl)-methyl aziridine: comparison between Free Energies obtained at different theory level.

| Structure | ΔG (kJ/mol)[a] | G (Hartree) [a] | ΔG (kJ/mol) [b] | G (Hartree) [b] |
|---|---|---|---|---|
| | *Anti*-Markovnikov (A) | | *Anti*-Markovnikov (B) | |
| Complex-1 | 0,0 | -1142,35944836 | 0,0 | -1142.36293795 |
| TS1 | +78.7 | -1142.32948224 | +78.8 | -1142.33293874 |
| I1 | -10.2 | -1142.36334256 | -9.0 | -1142.36637520 |
| TS2 | +4.8 | -1142.35763571 | +5,8 | -1142.36073507 |
| P1 | -202.0 | -1142.43640126 | -201.5 | -1142.43967762 |
| | Markovnikov (A) | | Markovnikov (B) | |
| TS1 | +75.2 | -1142.33080044 | +75.4 | -1142.33420243 |
| I1 | -7.2 | -1142.36219417 | -5.6 | -1142.36508446 |
| TS2 | +8.3 | -1142.35630147 | +9.2 | -1142.35942931 |

[a] (A) see Table 3 and Computational Section, main text, and Thermochemical Data, SI.

[b] (B) B3LYP-D3/6-311+G(2D,P)//B3LYP-D3/6-311+G(2D,P) corrected by a solvent-simulation SP at same theory level (PCM calculation)

---------------------------------------------------------------------------------------------------------------------------
---------------------------------------------------------------------------------------------------------------------------

CARTESIAN COORDINATES and THEMOCHEMICAL DATA

PRELIMINARY RESULTS (ANTIPERIPLANAR RING OPENING, REF 25, MAIN TEXT)

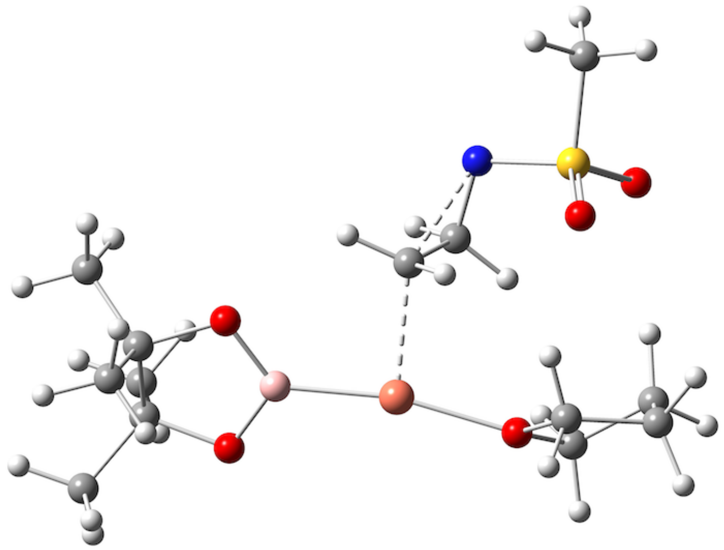

TS (Ref 25)                    Substrate: mesylaziridine

CARTESIAN COORDINATES

| | | | |
|---|---|---|---|
| C | -1.214511 | 1.044693 | 1.111519 |
| N | -1.974511 | 2.155272 | 0.564555 |
| O | -4.251355 | 1.057294 | 1.167402 |
| C | -0.515316 | 0.947208 | -0.202602 |
| Cu | 0.309145 | -1.056888 | -0.155875 |
| B | 2.134057 | -0.331278 | -0.106477 |
| O | 3.183049 | -1.088144 | 0.376530 |
| O | 2.551876 | 0.922031 | -0.509785 |
| C | 4.350244 | -0.215069 | 0.507539 |
| C | 4.012935 | 0.939724 | -0.501981 |
| O | -1.379057 | -2.156960 | -0.195956 |
| C | -2.053010 | -2.645184 | 1.000157 |
| C | -2.323451 | -2.113699 | -1.322463 |
| C | -3.528990 | -2.361437 | 0.748275 |
| H | -1.839691 | -3.717683 | 1.096574 |
| H | -1.623950 | -2.115552 | 1.854617 |
| C | -3.654358 | -2.626467 | -0.763526 |
| H | -2.400098 | -1.074283 | -1.643231 |
| H | -1.905675 | -2.734477 | -2.120138 |
| H | -3.769098 | -1.317655 | 0.974502 |
| H | -4.179005 | -3.007328 | 1.347199 |
| H | -4.497546 | -2.090616 | -1.207281 |
| H | -3.774398 | -3.698618 | -0.960870 |
| H | -1.103806 | 0.643324 | -1.065341 |
| H | 0.273111 | 1.663075 | -0.400617 |
| H | -1.790659 | 0.166537 | 1.418377 |
| C | 4.480293 | 2.328638 | -0.076980 |
| H | 5.572093 | 2.353498 | 0.024163 |
| H | 4.190370 | 3.063124 | -0.835700 |
| H | 4.033267 | 2.631527 | 0.872730 |
| C | 4.463562 | 0.644438 | -1.938293 |
| H | 4.019606 | 1.387591 | -2.608342 |
| H | 5.553707 | 0.695762 | -2.035348 |
| H | 4.128404 | -0.346237 | -2.261641 |
| C | 5.601844 | -1.021460 | 0.174760 |
| H | 6.488508 | -0.375751 | 0.179968 |
| H | 5.745160 | -1.805530 | 0.925617 |
| H | 5.523633 | -1.502006 | -0.803170 |
| C | 4.389310 | 0.246729 | 1.969841 |
| H | 4.424291 | -0.634430 | 2.618410 |
| H | 5.271936 | 0.863530 | 2.172648 |
| H | 3.493572 | 0.821214 | 2.226856 |
| S | -3.490197 | 1.691519 | 0.059727 |
| C | -4.205734 | 3.306491 | -0.282501 |
| H | -3.595750 | 3.797393 | -1.042869 |
| H | -5.218608 | 3.126753 | -0.650358 |
| H | -4.220826 | 3.884116 | 0.642982 |
| O | -3.482421 | 0.924046 | -1.218888 |
| H | -0.562954 | 1.363892 | 1.928597 |

Electronic Energy = -1562.96129548 (Hartree/Particle)
Dipole Moment (Debye): 3.6448
index 0
Harmonic frequencies
Number of imaginary frequencies= 1
Negatives Eigenvalues: -182.255

THERMOCHEMICAL DATA

```
 Temperature   298.150 Kelvin.  Pressure   1.00000 Atm.
 Zero-point correction=                           0.409514
(Hartree/Particle)
 Thermal correction to Energy=                    0.435819
 Thermal correction to Enthalpy=                  0.436763
 Thermal correction to Gibbs Free Energy=         0.349837
 Sum of electronic and zero-point Energies=          -1562.551781
 Sum of electronic and thermal Energies=             -1562.525477
 Sum of electronic and thermal Enthalpies=           -1562.524533
 Sum of electronic and thermal Free Energies=        -1562.611458
 PCM-SP (solvent=THF;6-311+g(2d,p)) = -1563.31552956
 Corrected Free Energy =  -1562.96569208
 Corrected Free Energy =  -1533.52220177
```

------------------------------------------------------

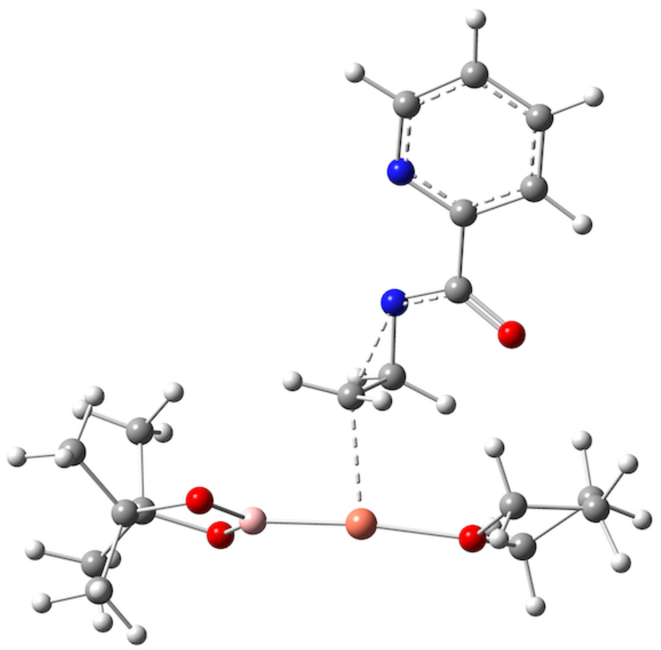

TS (Ref 25)          Substrate:  N-(2picolinoyl)-aziridine

CARTESIAN COORDINATES

| | | | |
|---|---|---|---|
| C | 0.679183 | -0.020737 | 1.429902 |
| N | 1.673513 | -1.015347 | 1.084563 |
| C | 2.814244 | -0.481574 | 0.541481 |
| O | 3.039292 | 0.733514 | 0.352355 |
| C | 0.168996 | -0.312002 | 0.069698 |
| C | 3.885059 | -1.483858 | 0.172043 |
| C | 5.189901 | -1.012577 | -0.044352 |
| C | 6.187407 | -1.924605 | -0.385231 |
| C | 5.846317 | -3.272460 | -0.514334 |
| C | 4.514373 | -3.640683 | -0.291999 |
| N | 3.551416 | -2.779291 | 0.049630 |
| Cu | -1.152326 | 1.511397 | -0.301088 |
| H | 0.018884 | -0.356390 | 2.230585 |
| H | 5.391249 | 0.048171 | 0.057719 |
| H | 7.209061 | -1.590847 | -0.549363 |
| H | 4.207475 | -4.680967 | -0.391907 |
| H | 6.586457 | -4.022036 | -0.781035 |
| B | -2.614208 | 0.214806 | -0.214424 |

| | | | |
|---|---|---|---|
| O | -3.455072 | 0.099943 | 0.877218 |
| O | -2.894594 | -0.739084 | -1.175497 |
| C | -4.204268 | -1.147901 | 0.742295 |
| C | -4.139800 | -1.406850 | -0.805104 |
| O | 0.203027 | 2.996973 | -0.370751 |
| C | 0.539217 | 3.761412 | 0.827362 |
| C | 1.345859 | 2.968876 | -1.288296 |
| C | 2.055714 | 3.925880 | 0.777606 |
| H | 0.008324 | 4.719116 | 0.767259 |
| H | 0.173534 | 3.200759 | 1.691568 |
| C | 2.322138 | 4.004354 | -0.735674 |
| H | 1.781201 | 1.967374 | -1.250718 |
| H | 0.960696 | 3.190260 | -2.286837 |
| H | 2.548176 | 3.039830 | 1.189318 |
| H | 2.390075 | 4.815368 | 1.320823 |
| H | 3.355349 | 3.755143 | -0.992652 |
| H | 2.093485 | 5.005193 | -1.122606 |
| H | 0.717052 | 0.059287 | -0.790464 |
| H | -0.416710 | -1.206072 | -0.093020 |
| H | 1.043791 | 0.996471 | 1.590392 |
| C | -4.043615 | -2.874649 | -1.211470 |
| H | -4.926354 | -3.428478 | -0.869034 |
| H | -3.992835 | -2.952188 | -2.302652 |
| H | -3.150403 | -3.349712 | -0.798942 |
| C | -5.263178 | -0.712656 | -1.585895 |
| H | -5.036545 | -0.768176 | -2.655494 |
| H | -6.232006 | -1.193509 | -1.409877 |
| H | -5.339883 | 0.344084 | -1.310577 |
| C | -5.606646 | -0.940774 | 1.306366 |
| H | -6.223332 | -1.833247 | 1.143707 |
| H | -5.547030 | -0.758060 | 2.384518 |
| H | -6.102841 | -0.082913 | 0.846549 |
| C | -3.451208 | -2.200060 | 1.567117 |
| H | -3.367931 | -1.846867 | 2.599930 |
| H | -3.975884 | -3.161925 | 1.569398 |
| H | -2.438452 | -2.357355 | 1.182041 |

Electronic Energy = -1335.50039192 (Hartree/Particle)
Dipole Moment (Debye): 3.1329
index 1
Number of imaginary frequencies= 1
Negatives Eigenvalues: -322.373

THERMOCHEMICAL DATA

Temperature 298.150 Kelvin. Pressure 1.00000 Atm.
Zero-point correction= 0.450274
(Hartree/Particle)
Thermal correction to Energy= 0.478160
Thermal correction to Enthalpy= 0.479105
Thermal correction to Gibbs Free Energy= 0.386814
Sum of electronic and zero-point Energies= -1335.050118
Sum of electronic and thermal Energies= -1335.022231
Sum of electronic and thermal Enthalpies= -1335.021287
Sum of electronic and thermal Free Energies= -1335.113582
PCM-SP (solvent=THF;6-311+g(2d,p)) = -1335.83942948
Corrected Free Energy = -1335.45261956

-----------------------------------------------------------------

MODEL: Az-Cu-Bpin

SUBSTRATE: N-MESYL-AZIRIDINE

N-mesyl-aziridine

CARTESIAN COORDINATES

| | | | |
|---|---|---|---|
| C | 2.019232 | -0.608194 | -0.197541 |
| C | 1.912543 | 0.834411 | 0.143586 |
| H | 1.841746 | -1.321369 | 0.605557 |
| H | 2.490274 | 1.576843 | -0.400705 |
| N | 0.867954 | 0.183199 | -0.679551 |
| H | 2.675838 | -0.950295 | -0.992513 |
| H | 1.656183 | 1.079936 | 1.172243 |
| S | -0.632935 | -0.175458 | 0.087113 |
| O | -0.516997 | -0.056953 | 1.552431 |
| O | -1.124224 | -1.421630 | -0.507619 |
| C | -1.611208 | 1.205657 | -0.525850 |
| H | -2.613207 | 1.085760 | -0.106944 |
| H | -1.159879 | 2.137448 | -0.178901 |
| H | -1.633305 | 1.154043 | -1.615359 |

Electronic Energy = -721.839183223 (Hartree/Particle)
Dipole Moment (Debye): 4.0306
index 0
Harmonic frequencies
Number of imaginary frequencies= 0

THERMOCHEMICAL DATA

Temperature 298.150 Kelvin. Pressure 1.00000 Atm.
Zero-point correction= 0.108343
(Hartree/Particle)
Thermal correction to Energy= 0.115923
Thermal correction to Enthalpy= 0.116867
Thermal correction to Gibbs Free Energy= 0.076193
Sum of electronic and zero-point Energies= -721.730840
Sum of electronic and thermal Energies= -721.723260
Sum of electronic and thermal Enthalpies= -721.722316
Sum of electronic and thermal Free Energies= -721.762990
PCM-SP (solvent=THF;6-311+g(2d,p)) = -721.994189343
Corrected Free Energy = -721.917996120

-----------------------------------------------------

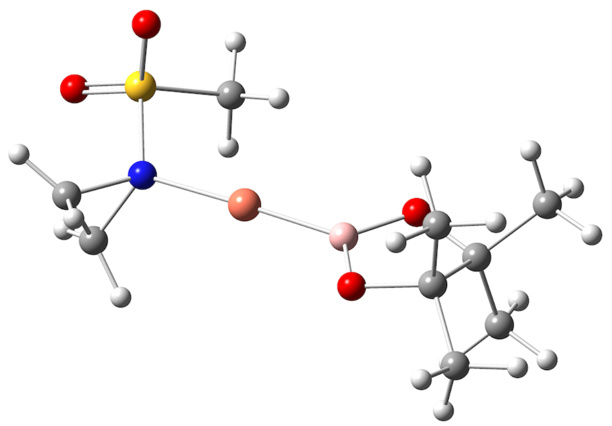

Complex-1    Substrate: N-mesyl-aziridine

CARTESIAN COORDINATES

| | | | |
|---|---|---|---|
| C | -3.100354 | -1.568728 | -1 .265188 |
| C | -3.178320 | -2.019169 | 0.144267 |
| H | -4.025511 | -1.253842 | -1.740983 |
| H | -2.452342 | -2.718786 | 0.546056 |
| N | -2.591149 | -0.686416 | -0.175152 |
| Cu | -0.556083 | -0.450515 | -0.032506 |
| B | 1.385038 | -0.194298 | -0.014723 |
| O | 2.332615 | -1.200985 | 0.123822 |
| O | 2.005551 | 1.045201 | -0.138632 |
| C | 3.637505 | -0.587854 | 0.328979 |
| C | 3.437797 | 0.835758 | -0.300255 |
| C | 4.698647 | -1.452060 | -0.348108 |
| H | 5.684615 | -0.974700 | -0.286373 |
| H | 4.757010 | -2.423809 | 0.153952 |
| H | 4.460869 | -1.631219 | -1.399420 |
| C | 3.877354 | -0.552881 | 1.845055 |
| H | 3.803662 | -1.572437 | 2.237310 |
| H | 4.869997 | -0.156758 | 2.088478 |
| H | 3.120972 | 0.057707 | 2.348328 |
| C | 3.724557 | 0.878773 | -1.808060 |
| H | 3.372439 | 1.835821 | -2.207054 |
| H | 4.796255 | 0.787138 | -2.019052 |
| H | 3.192837 | 0.077670 | -2.331431 |
| C | 4.182961 | 1.966174 | 0.405550 |
| H | 5.266108 | 1.793554 | 0.377232 |
| H | 3.974860 | 2.916050 | -0.098864 |
| H | 3.870605 | 2.064133 | 1.447910 |
| H | -2.311039 | -1.932680 | -1.916104 |
| H | -4.155348 | -2.014541 | 0.621821 |
| S | -3.609941 | 0.679444 | 0.291328 |
| O | -3.440866 | 0.834826 | 1.734529 |
| O | -4.945908 | 0.483484 | -0.290023 |
| C | -2.762778 | 2.006827 | -0.579610 |
| H | -3.330463 | 2.916954 | -0.368970 |
| H | -1.741757 | 2.076447 | -0.197031 |
| H | -2.770813 | 1.782280 | -1.648044 |

Electronic Energy =  -1330.51255415 (Hartree/Particle)
Dipole Moment (Debye):      3.2042
index       0
Harmonic frequencies
Number of imaginary frequencies=        0

THERMOCHEMICAL DATA

Temperature 298.150 Kelvin. Pressure 1.00000 Atm.
Zero-point correction= 0.291959
(Hartree/Particle)
Thermal correction to Energy= 0.312198
Thermal correction to Enthalpy= 0.313143
Thermal correction to Gibbs Free Energy= 0.240581
Sum of electronic and zero-point Energies= -1330.220595
Sum of electronic and thermal Energies= -1330.200356
Sum of electronic and thermal Enthalpies= -1330.199412
Sum of electronic and thermal Free Energies= -1330.271973
PCM-SP (solvent=THF;6-311+g(2d,p)) = -1330.79867657
Corrected Free Energy = -1330.55809542

-------------------------------------------------------

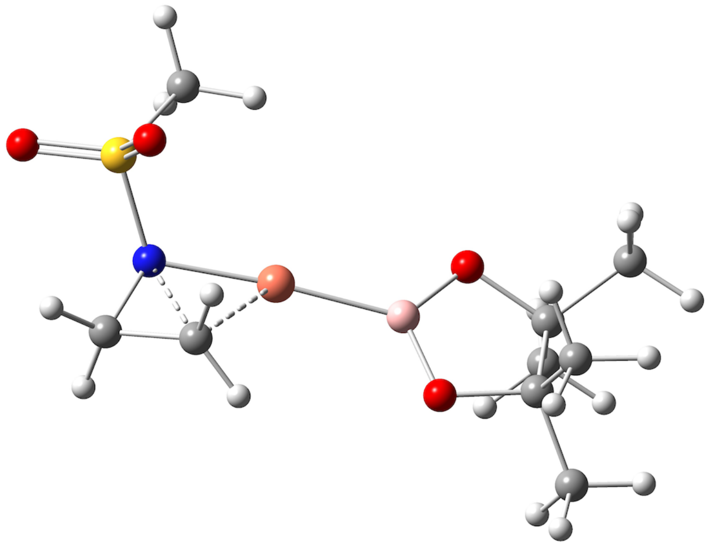

TS1 Substrate: N-mesyl-aziridine

CARTESIAN COORDINATES

| | | | |
|---|---|---|---|
| Cu | -0.660995 | -0.149623 | -0.420382 |
| B | 1.299961 | 0.010456 | -0.191299 |
| O | 2.033328 | 1.184527 | -0.116695 |
| C | 3.452580 | 0.849631 | -0.155559 |
| C | 4.211526 | 1.827197 | 0.738111 |
| N | -2.563877 | -0.442353 | -0.687457 |
| C | -1.787653 | -1.953704 | 0.260251 |
| C | -2.932729 | -1.881900 | -0.663351 |
| O | 2.127825 | -1.097967 | -0.060054 |
| C | 3.455758 | -0.641393 | 0.335780 |
| C | 3.531971 | -0.778077 | 1.862646 |
| C | 4.500518 | -1.538058 | -0.323416 |
| C | 3.903688 | 1.010113 | -1.613899 |
| H | -3.922623 | -2.094645 | -0.254542 |
| H | -1.976254 | -1.775610 | 1.314097 |
| H | -0.855332 | -2.439626 | -0.020197 |
| H | 5.512803 | -1.170383 | -0.114819 |
| H | 4.417767 | -2.554953 | 0.075146 |
| H | 4.362388 | -1.588856 | -1.406018 |
| H | 3.328405 | -1.818874 | 2.135154 |

| | | | |
|---|---|---|---|
| H | 4.523301 | -0.507101 | 2.243222 |
| H | 2.783073 | -0.147298 | 2.352129 |
| H | 3.683298 | 2.031338 | -1.941247 |
| H | 4.979548 | 0.833509 | -1.725502 |
| H | 3.363911 | 0.320829 | -2.271069 |
| H | 5.271849 | 1.552443 | 0.799171 |
| H | 4.143645 | 2.837088 | 0.319736 |
| H | 3.797433 | 1.853742 | 1.748816 |
| H | -2.801217 | -2.334724 | -1.646042 |
| S | -3.666953 | 0.492911 | 0.271291 |
| O | -3.604330 | 0.093053 | 1.688146 |
| O | -4.960063 | 0.449174 | -0.425343 |
| C | -2.943080 | 2.131224 | 0.084598 |
| H | -1.951841 | 2.132795 | 0.545568 |
| H | -2.887920 | 2.367472 | -0.979050 |
| H | -3.614362 | 2.819040 | 0.605163 |

Electronic Energy = -1330.47537013 (Hartree/Particle)
Dipole Moment (Debye): 2.6389
index 1
Number of imaginary frequencies= 1
Negatives Eigenvalues: -276.427

THERMOCHEMICAL DATA

Temperature 298.150 Kelvin. Pressure 1.00000 Atm.
Zero-point correction= 0.289830
(Hartree/Particle)
Thermal correction to Energy= 0.309650
Thermal correction to Enthalpy= 0.310595
Thermal correction to Gibbs Free Energy= 0.239583
Sum of electronic and zero-point Energies= -1330.185540
Sum of electronic and thermal Energies= -1330.165720
Sum of electronic and thermal Enthalpies= -1330.164775
Sum of electronic and thermal Free Energies= -1330.235787
PCM-SP (solvent=THF;6-311+g(2d,p)) = -1330.761392330
Corrected Free Energy = -1330.521809200

---

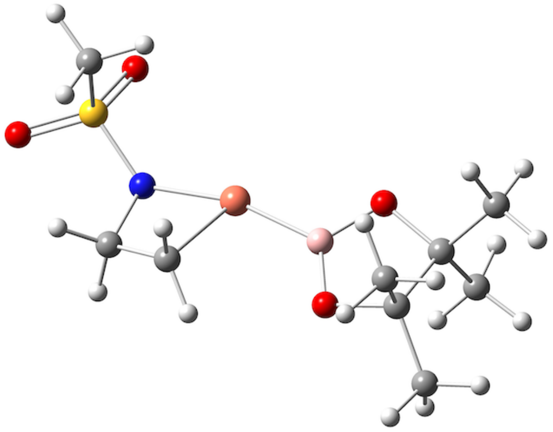

I1 Substrate: N-mesyl-aziridine

CARTESIAN COORDINATES

| | | | |
|---|---|---|---|
| Cu | 0.642652 | -0.143437 | -0.284199 |
| B | -1.318963 | -0.028205 | -0.098457 |

| | | | |
|---|---|---|---|
| O | -2.123989 | 1.071973 | -0.216555 |
| C | -3.479553 | 0.665147 | 0.178271 |
| C | -4.481727 | 1.407347 | -0.698874 |
| N | 2.479654 | 0.228452 | -0.604136 |
| C | 2.260502 | 1.700584 | -0.512522 |
| C | 0.863760 | 1.780693 | 0.078729 |
| O | -2.004528 | -1.187310 | 0.154002 |
| C | -3.435599 | -0.893374 | -0.034850 |
| C | -3.780877 | -1.326350 | -1.463385 |
| C | -4.231299 | -1.708325 | 0.978177 |
| C | -3.643476 | 1.076525 | 1.645567 |
| H | 2.997709 | 2.202185 | 0.130747 |
| H | -5.501667 | 1.064341 | -0.488664 |
| H | -4.432183 | 2.480641 | -0.488478 |
| H | -4.274878 | 1.261397 | -1.761381 |
| H | -3.467192 | 2.153160 | 1.734384 |
| H | -4.653196 | 0.859487 | 2.010040 |
| H | -2.922141 | 0.559254 | 2.286405 |
| H | -3.531360 | -2.385054 | -1.583535 |
| H | -4.848189 | -1.195102 | -1.671485 |
| H | -3.207821 | -0.756894 | -2.202082 |
| H | -5.295452 | -1.449111 | 0.924706 |
| H | -4.127488 | -2.775078 | 0.755831 |
| H | -3.880232 | -1.540237 | 1.998739 |
| H | 0.801645 | 1.838354 | 1.166847 |
| H | 0.086395 | 2.354877 | -0.422539 |
| H | 2.320795 | 2.153528 | -1.507103 |
| S | 3.882980 | -0.385196 | -0.002809 |
| C | 3.741918 | -0.322673 | 1.810577 |
| H | 2.906476 | -0.957016 | 2.115934 |
| H | 4.680153 | -0.698202 | 2.227139 |
| H | 3.583609 | 0.713549 | 2.121326 |
| O | 3.920429 | -1.810572 | -0.367094 |
| O | 5.002559 | 0.513802 | -0.348982 |

Electronic Energy = -1330.48570273 (Hartree/Particle)
Dipole Moment (Debye): 10.8658
index 0
Harmonic frequencies
Number of imaginary frequencies= 0

THERMOCHEMICAL DATA

Temperature 298.150 Kelvin. Pressure 1.00000 Atm.
Zero-point correction= 0.290784 (Hartree/Particle)
Thermal correction to Energy= 0.310947
Thermal correction to Enthalpy= 0.311891
Thermal correction to Gibbs Free Energy= 0.240029
Sum of electronic and zero-point Energies= -1330.194918
Sum of electronic and thermal Energies= -1330.174756
Sum of electronic and thermal Enthalpies= -1330.173812
Sum of electronic and thermal Free Energies= -1330.245674
PCM-SP (solvent=THF;6-311+g(2d,p)) = -1330.78025244
Corrected Free Energy = -1330.54022371

-------------------------------------------------------

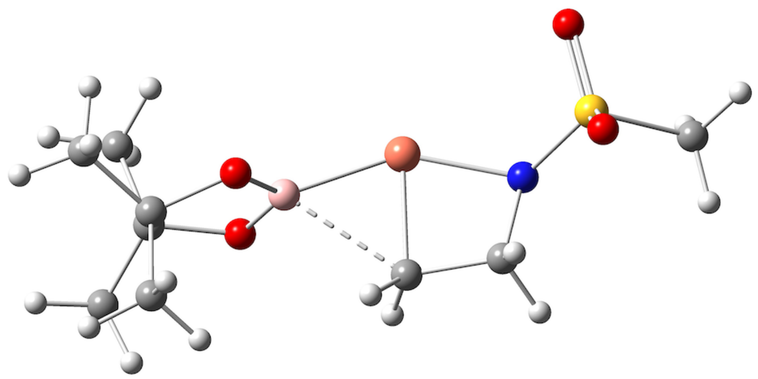

TS2 Substrate: N-mesyl-aziridine

CARTESIAN COORDINATES

| | | | |
|---|---|---|---|
| Cu | -0.648959 | -0.378699 | 0.061505 |
| B | 1.311276 | -0.149153 | -0.008806 |
| O | 1.968780 | 0.981073 | -0.414033 |
| C | 3.384473 | 0.621264 | -0.579362 |
| C | 4.236664 | 1.814397 | -0.161214 |
| N | -2.467881 | -0.226409 | 0.601905 |
| C | -2.165880 | 0.959099 | 1.453407 |
| C | -0.754910 | 1.336549 | 1.028064 |
| O | 2.142278 | -1.180890 | 0.329633 |
| C | 3.514514 | -0.646238 | 0.344294 |
| C | 3.826337 | -0.309163 | 1.806092 |
| C | 4.459173 | -1.726827 | -0.168451 |
| C | 3.583757 | 0.315881 | -2.067892 |
| H | -2.861526 | 1.794319 | 1.284704 |
| H | 5.301006 | 1.552846 | -0.190205 |
| H | 4.070415 | 2.646673 | -0.852734 |
| H | 3.987913 | 2.156606 | 0.845907 |
| H | 3.283850 | 1.190726 | -2.653167 |
| H | 4.632098 | 0.091357 | -2.291418 |
| H | 2.969395 | -0.532495 | -2.386048 |
| H | 3.699572 | -1.210549 | 2.413424 |
| H | 4.855864 | 0.046441 | 1.921473 |
| H | 3.147123 | 0.458178 | 2.191697 |
| H | 5.479782 | -1.334308 | -0.250050 |
| H | 4.471006 | -2.568322 | 0.531493 |
| H | 4.149670 | -2.104738 | -1.145331 |
| H | -0.659489 | 2.066483 | 0.222988 |
| H | 0.011396 | 1.457678 | 1.792705 |
| H | -2.221700 | 0.687234 | 2.512417 |
| S | -3.901763 | -0.259643 | -0.197900 |
| C | -3.766099 | 0.937889 | -1.562391 |
| H | -2.967244 | 0.611812 | -2.232578 |
| H | -4.724875 | 0.950453 | -2.087157 |
| H | -3.553130 | 1.927994 | -1.150385 |
| O | -4.016529 | -1.587210 | -0.823634 |
| O | -4.971093 | 0.257242 | 0.680590 |

Electronic Energy = -1330.48452246 (Hartree/Particle)
Dipole Moment (Debye): 11.2461
index 0
Harmonic frequencies
Number of imaginary frequencies= 1

Negatives Eigenvalues: -16.336

THERMOCHEMICAL DATA

Temperature 298.150 Kelvin. Pressure 1.00000 Atm.
Zero-point correction= 0.290658
(Hartree/Particle)
Thermal correction to Energy= 0.309967
Thermal correction to Enthalpy= 0.310911
Thermal correction to Gibbs Free Energy= 0.242113
Sum of electronic and zero-point Energies= -1330.193864
Sum of electronic and thermal Energies= -1330.174555
Sum of electronic and thermal Enthalpies= -1330.173611
Sum of electronic and thermal Free Energies= -1330.242409
PCM-SP (solvent=THF;6-311+g(2d,p)) = -1330.78049149
Corrected Free Energy = -1330.53837803

------------------------------------------------------

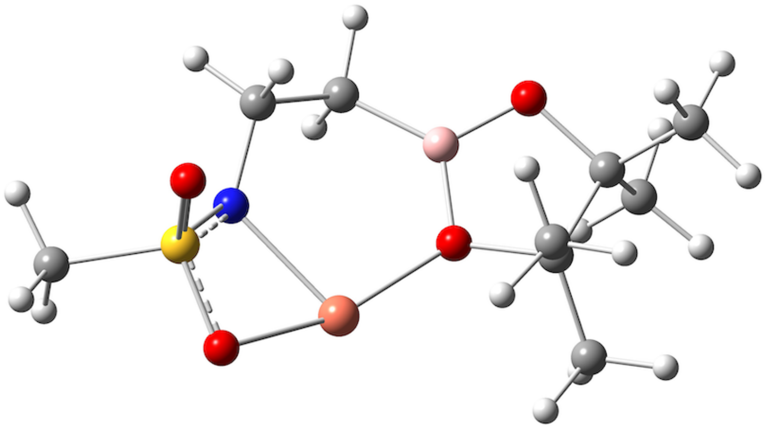

P1 Substrate: N-mesyl-aziridine

CARTESIAN COORDINATES

| | | | |
|---|---|---|---|
| C | -1.353679 | 2.049226 | 0.012723 |
| Cu | -0.739178 | -0.987884 | -0.562338 |
| B | 1.168639 | 1.227236 | -0.290043 |
| O | 1.082040 | -0.171239 | -0.459895 |
| C | 2.255803 | -0.784976 | 0.204661 |
| C | 2.634011 | -2.035222 | -0.576005 |
| C | 0.039774 | 2.268621 | -0.627333 |
| N | -1.903204 | 0.794614 | -0.505440 |
| O | 2.407379 | 1.569987 | 0.172353 |
| C | 3.278929 | 0.399432 | 0.140476 |
| C | 4.052670 | 0.461760 | -1.181483 |
| C | 4.236591 | 0.474666 | 1.325270 |
| C | 1.811399 | -1.141164 | 1.625236 |
| H | -1.991735 | 2.907352 | -0.251024 |
| H | -0.097478 | 2.271688 | -1.720312 |
| H | 0.409859 | 3.266655 | -0.358244 |
| H | 4.846206 | -0.434516 | 1.384539 |
| H | 4.907927 | 1.329916 | 1.199169 |
| H | 3.701094 | 0.601908 | 2.268663 |
| H | 4.590671 | 1.413308 | -1.231154 |
| H | 4.780070 | -0.353497 | -1.256333 |
| H | 3.376737 | 0.408229 | -2.041205 |
| H | 0.945891 | -1.811251 | 1.574082 |

```
 H              2.611248   -1.651308    2.171292
 H              1.513226   -0.250583    2.186633
 H              3.543009   -2.483596   -0.159816
 H              1.825147   -2.770827   -0.500054
 H              2.799057   -1.819522   -1.633817
 H             -1.268499    2.037713    1.108990
 S             -3.005568   -0.008979    0.330010
 C             -4.639454    0.218086   -0.413979
 H             -4.566249   -0.034446   -1.473236
 H             -5.341973   -0.442124    0.100173
 H             -4.927480    1.263587   -0.278981
 O             -2.670392   -1.472169    0.009188
 O             -3.137591    0.346241    1.761999
```

Electronic Energy = -1330.56983965 (Hartree/Particle)
Dipole Moment (Debye): 6.4223
index 0
Harmonic frequencies
Number of imaginary frequencies= 0

THERMOCHEMICAL DATA

Temperature 298.150 Kelvin. Pressure 1.00000 Atm.
Zero-point correction= 0.292852 (Hartree/Particle)
Thermal correction to Energy= 0.312324
Thermal correction to Enthalpy= 0.313268
Thermal correction to Gibbs Free Energy= 0.245148
Sum of electronic and zero-point Energies= -1330.276988
Sum of electronic and thermal Energies= -1330.257516
Sum of electronic and thermal Enthalpies= -1330.256572
Sum of electronic and thermal Free Energies= -1330.324692
PCM-SP (solvent=THF;6-311+g(2d,p)) = -1330.85610185
Corrected Free Energy = -1330.61095420

------------------------------------------------------

SUBSTRATE: N-TOSYL-AZIRIDINE

N-tosyl-aziridine

CARTESIAN COORDINATES

```
 C             -3.081264   -0.437755   -0.877590
 C             -2.067213   -1.522640   -0.958585
 H             -3.581488   -0.301747    0.079509
 H             -1.867585   -2.031399   -1.897930
 N             -1.641945   -0.107525   -0.954165
 H             -3.644443   -0.129452   -1.753980
 H             -1.896401   -2.100761   -0.052647
 S             -0.947445    0.498482    0.502044
 O             -1.372423   -0.315244    1.656589
 O             -1.183403    1.944701    0.487712
 C              0.783745    0.168579    0.178296
 C              1.490678    1.044498   -0.649247
 C              1.384016   -0.953505    0.752218
 C              2.834768    0.778364   -0.913606
 H              0.994720    1.915176   -1.066026
 C              2.731073   -1.206576    0.480574
```

| | | | |
|---|---|---|---|
| H | 0.808925 | -1.598341 | 1.408821 |
| C | 3.452242 | -0.345059 | -0.351653 |
| H | 3.400998 | 1.449837 | -1.553076 |
| H | 3.216134 | -2.072025 | 0.923554 |
| H | 4.500211 | -0.545415 | -0.558632 |

Electronic Energy = -913.586590503 (Hartree/Particle)
Dipole Moment (Debye): 4.6379
index 0
Harmonic frequencies
Number of imaginary frequencies= 0

THERMOCHEMICAL DATA

Temperature 298.150 Kelvin. Pressure 1.00000 Atm.
Zero-point correction= 0.161572 (Hartree/Particle)
Thermal correction to Energy= 0.172181
Thermal correction to Enthalpy= 0.173125
Thermal correction to Gibbs Free Energy= 0.123997
Sum of electronic and zero-point Energies= -913.425019
Sum of electronic and thermal Energies= -913.414409
Sum of electronic and thermal Enthalpies= -913.413465
Sum of electronic and thermal Free Energies= -913.462594
PCM-SP (solvent=THF;6-311+g(2d,p)) = -913.784535565
Corrected Free Energy = -913.660539062

-------------------------------------------------------

Complex-1 Substrate: N-tosyl-aziridine

CARTESIAN COORDINATES

| | | | |
|---|---|---|---|
| C | -2.852053 | -1.808241 | -1.619934 |
| C | -2.826559 | -2.803543 | -0.520288 |
| H | -3.801741 | -1.322090 | -1.827468 |
| H | -2.102167 | -3.611929 | -0.517046 |
| N | -2.208606 | -1.463488 | -0.323723 |
| Cu | -0.172070 | -1.225181 | -0.267445 |
| B | 1.708918 | -0.697944 | -0.149375 |
| O | 2.840373 | -1.348321 | -0.621995 |
| O | 2.068563 | 0.502132 | 0.473050 |
| C | 4.022534 | -0.675170 | -0.105207 |
| C | 3.476038 | 0.761399 | 0.210114 |
| C | 5.122282 | -0.731153 | -1.163029 |
| H | 6.000665 | -0.158159 | -0.840451 |
| H | 5.430190 | -1.770688 | -1.318491 |
| H | 4.778187 | -0.336543 | -2.122113 |
| C | 4.459759 | -1.445136 | 1.149143 |
| H | 4.631846 | -2.491728 | 0.877702 |
| H | 5.385531 | -1.038057 | 1.572083 |
| H | 3.679881 | -1.419868 | 1.916834 |
| C | 3.533714 | 1.710715 | -0.995651 |
| H | 2.959181 | 2.613835 | -0.762788 |
| H | 4.562697 | 2.006133 | -1.230870 |
| H | 3.088236 | 1.246757 | -1.881276 |
| C | 4.090855 | 1.434491 | 1.434991 |
| H | 5.172419 | 1.564849 | 1.304979 |
| H | 3.643564 | 2.424817 | 1.576388 |
| H | 3.914362 | 0.853837 | 2.343464 |

| | | | |
|---|---|---|---|
| H | -2.145809 | -1.872460 | -2.442165 |
| H | -3.758110 | -2.988981 | 0.009102 |
| S | -3.113249 | -0.382635 | 0.757483 |
| O | -2.673871 | -0.746714 | 2.103055 |
| O | -4.531426 | -0.449363 | 0.373658 |
| C | -2.428935 | 1.192434 | 0.272627 |
| C | -3.141032 | 1.991903 | -0.626132 |
| C | -1.168817 | 1.553033 | 0.761082 |
| C | -2.563987 | 3.191451 | -1.047850 |
| H | -4.122413 | 1.683450 | -0.971340 |
| C | -0.600466 | 2.746997 | 0.315251 |
| H | -0.633789 | 0.914358 | 1.454892 |
| C | -1.295957 | 3.561909 | -0.583246 |
| H | -3.102297 | 3.832796 | -1.739970 |
| H | 0.391833 | 3.017193 | 0.661107 |
| H | -0.847979 | 4.490613 | -0.926837 |

Electronic Energy = -1522.26036101 (Hartree/Particle)
Dipole Moment (Debye): 4.0242
index 0
Harmonic frequencies
Number of imaginary frequencies= 0

THERMOCHEMICAL DATA

Temperature 298.150 Kelvin. Pressure 1.00000 Atm.
Zero-point correction= 0.345112 (Hartree/Particle)
Thermal correction to Energy= 0.368409
Thermal correction to Enthalpy= 0.369353
Thermal correction to Gibbs Free Energy= 0.289816
Sum of electronic and zero-point Energies= -1521.915249
Sum of electronic and thermal Energies= -1521.891952
Sum of electronic and thermal Enthalpies= -1521.891008
Sum of electronic and thermal Free Energies= -1521.970545
PCM-SP (solvent=THF;6-311+g(2d,p)) = -1522.590872550
Corrected Free Energy = -1522.301056540

---

TS1 Substrate: N-tosyl-aziridine

CARTESIAN COORDINATES

| | | | |
|---|---|---|---|
| Cu | -0.332187 | -0.913672 | -0.480565 |
| B | 1.590291 | -0.479914 | -0.263171 |
| O | 2.045575 | 0.728972 | 0.256381 |
| C | 3.492711 | 0.788054 | 0.098553 |
| C | 4.086825 | 1.528040 | 1.294529 |
| N | -2.215322 | -1.373741 | -0.655081 |
| C | -1.271629 | -2.769831 | 0.316371 |
| C | -2.491312 | -2.825039 | -0.509216 |
| O | 2.659761 | -1.319073 | -0.547398 |
| C | 3.876498 | -0.730221 | -0.003191 |
| C | 4.114009 | -1.388816 | 1.363062 |
| C | 5.037713 | -1.042713 | -0.943675 |
| C | 3.765304 | 1.571559 | -1.193129 |
| H | -3.425512 | -3.069313 | 0.000327 |
| H | -1.392651 | -2.558949 | 1.373861 |
| H | -0.340272 | -3.221803 | -0.016503 |

| | | | |
|---|---|---|---|
| H | 5.953868 | -0.540971 | -0.608161 |
| H | 5.223284 | -2.122113 | -0.953989 |
| H | 4.821103 | -0.729727 | -1.967756 |
| H | 4.174743 | -2.473507 | 1.226446 |
| H | 5.047707 | -1.043810 | 1.821760 |
| H | 3.287006 | -1.179709 | 2.049338 |
| H | 3.291124 | 2.555558 | -1.116088 |
| H | 4.838946 | 1.714771 | -1.360511 |
| H | 3.338819 | 1.058751 | -2.061000 |
| H | 5.182977 | 1.516698 | 1.252677 |
| H | 3.756586 | 2.572573 | 1.282570 |
| H | 3.768235 | 1.084919 | 2.241186 |
| H | -2.413365 | -3.333595 | -1.470329 |
| S | -3.286090 | -0.425381 | 0.337747 |
| O | -3.225145 | -0.891615 | 1.734652 |
| O | -4.575537 | -0.407860 | -0.365845 |
| C | -2.501524 | 1.177580 | 0.201512 |
| C | -2.925215 | 2.054418 | -0.798743 |
| C | -1.434356 | 1.477460 | 1.053542 |
| C | -2.250382 | 3.268210 | -0.947621 |
| H | -3.758893 | 1.784722 | -1.438700 |
| C | -0.760412 | 2.687151 | 0.881711 |
| H | -1.131499 | 0.774477 | 1.822032 |
| C | -1.168646 | 3.578322 | -0.115458 |
| H | -2.564474 | 3.966409 | -1.718421 |
| H | 0.092252 | 2.917216 | 1.512113 |
| H | -0.638069 | 4.517298 | -0.248814 |

Electronic Energy = -1522.22116686 (Hartree/Particle)
Dipole Moment (Debye): 3.2788
index 1
Number of imaginary frequencies= 1
Negatives Eigenvalues: -277.345

THERMOCHEMICAL DATA

Temperature 298.150 Kelvin. Pressure 1.00000 Atm.
Zero-point correction= 0.342780 (Hartree/Particle)
Thermal correction to Energy= 0.365792
Thermal correction to Enthalpy= 0.366736
Thermal correction to Gibbs Free Energy= 0.287514
Sum of electronic and zero-point Energies= -1521.878387
Sum of electronic and thermal Energies= -1521.855375
Sum of electronic and thermal Enthalpies= -1521.854431
Sum of electronic and thermal Free Energies= -1521.933653
PCM-SP (solvent=THF;6-311+g(2d,p)) = -1522.55292705
Corrected Free Energy = -1522.26541319

---

I1 Substrate: N-tosyl-aziridine

CARTESIAN COORDINATES

| | | | |
|---|---|---|---|
| Cu | -0.146098 | -1.138052 | -0.149097 |
| B | 1.729532 | -0.540415 | -0.013173 |
| O | 2.523558 | -0.491431 | 1.101594 |
| C | 3.739188 | 0.247920 | 0.739655 |

| | | | |
|---|---|---|---|
| C | 4.919588 | -0.364760 | 1.485274 |
| N | -1.948906 | -1.722963 | 0.167552 |
| C | -1.757361 | -1.604615 | 1.650124 |
| C | -0.499336 | -0.768556 | 1.756013 |
| O | 2.354084 | -0.092061 | -1.148970 |
| C | 3.779221 | 0.078279 | -0.823831 |
| C | 4.485209 | -1.201821 | -1.282825 |
| C | 4.306729 | 1.284466 | -1.592591 |
| C | 3.512551 | 1.695524 | 1.190476 |
| H | -2.614919 | -1.133541 | 2.148363 |
| H | 5.857480 | 0.110598 | 1.174372 |
| H | 4.797457 | -0.207459 | 2.561877 |
| H | 4.995783 | -1.439812 | 1.307493 |
| H | 3.300054 | 1.703521 | 2.264217 |
| H | 4.397722 | 2.314115 | 1.007489 |
| H | 2.658685 | 2.143468 | 0.671908 |
| H | 4.294105 | -1.349447 | -2.350243 |
| H | 5.567787 | -1.136083 | -1.129488 |
| H | 4.108368 | -2.078407 | -0.746238 |
| H | 5.344417 | 1.495341 | -1.307523 |
| H | 4.282827 | 1.075903 | -2.666979 |
| H | 3.704176 | 2.176675 | -1.408066 |
| H | -0.643031 | 0.313260 | 1.755771 |
| H | 0.339531 | -1.099805 | 2.365551 |
| H | -1.629207 | -2.606899 | 2.068298 |
| S | -3.254256 | -0.931266 | -0.468436 |
| O | -3.251974 | -1.218746 | -1.910731 |
| O | -4.450196 | -1.179695 | 0.359206 |
| C | -2.860913 | 0.828764 | -0.279550 |
| C | -3.341227 | 1.535719 | 0.824854 |
| C | -1.984315 | 1.424356 | -1.194172 |
| C | -2.918984 | 2.854529 | 1.026168 |
| H | -4.044066 | 1.058293 | 1.500572 |
| C | -1.566380 | 2.739755 | -0.983828 |
| H | -1.651734 | 0.862194 | -2.062510 |
| C | -2.028323 | 3.453394 | 0.129241 |
| H | -3.291079 | 3.414418 | 1.880352 |
| H | -0.888893 | 3.209798 | -1.691969 |
| H | -1.703505 | 4.478183 | 0.289697 |

Electronic Energy = -1522.23482096 (Hartree/Particle)
Dipole Moment (Debye): 10.8657
index 0
Harmonic frequencies
Number of imaginary frequencies= 0

THERMOCHEMICAL DATA

Temperature 298.150 Kelvin. Pressure 1.00000 Atm.
Zero-point correction= 0.344144 (Hartree/Particle)
Thermal correction to Energy= 0.367282
Thermal correction to Enthalpy= 0.368227
Thermal correction to Gibbs Free Energy= 0.288094
Sum of electronic and zero-point Energies= -1521.890677
Sum of electronic and thermal Energies= -1521.867539
Sum of electronic and thermal Enthalpies= -1521.866594
Sum of electronic and thermal Free Energies= -1521.946726
PCM-SP (solvent=THF;6-311+g(2d,p)) = -1522.57473379
Corrected Free Energy = -1522.28663883

-------------------------------------------------------

SUBSTRATE: N-(2-PICOLINOYL)-AZIRIDINE

N-(2-picolinoyl)-aziridine

CARTESIAN COORDINATES

| | | | |
|---|---|---|---|
| C | -3.256706 | -0.213857 | 0.114189 |
| C | -2.299273 | -1.355547 | 0.337341 |
| H | -3.453074 | 0.470213 | 0.938665 |
| H | -1.850832 | -1.460163 | 1.323339 |
| H | -2.405086 | -2.282572 | -0.220964 |
| N | -1.922587 | -0.193835 | -0.443387 |
| C | -0.969823 | 0.757536 | -0.137435 |
| C | 0.459104 | 0.271375 | -0.075166 |
| C | 1.476976 | 1.222972 | 0.071304 |
| C | 2.797611 | 0.780803 | 0.131453 |
| H | 1.214297 | 2.273506 | 0.131456 |
| C | 1.963234 | -1.459467 | -0.088757 |
| C | 3.050316 | -0.589668 | 0.049367 |
| H | 3.613065 | 1.491068 | 0.239930 |
| H | 2.120886 | -2.534648 | -0.152690 |
| H | 4.062585 | -0.981700 | 0.091817 |
| O | -1.244142 | 1.945106 | 0.001024 |
| N | 0.692381 | -1.049127 | -0.152119 |
| H | -4.065895 | -0.320708 | -0.604978 |

Electronic Energy = -494.386797421 (Hartree/Particle)
Dipole Moment (Debye): 3.1534
index 0
Harmonic frequencies
Number of imaginary frequencies= 0

THERMOCHEMICAL DATA

Temperature 298.150 Kelvin. Pressure 1.00000 Atm.
Zero-point correction= 0.149540
(Hartree/Particle)
Thermal correction to Energy= 0.158597
Thermal correction to Enthalpy= 0.159541
Thermal correction to Gibbs Free Energy= 0.114419
Sum of electronic and zero-point Energies= -494.237257
Sum of electronic and thermal Energies= -494.228201
Sum of electronic and thermal Enthalpies= -494.227257
Sum of electronic and thermal Free Energies= -494.272378
PCM-SP (solvent=THF;6-311+g(2d,p)) = -494.520574019
Corrected Free Energy = -494.406154598

-------------------------------------------------------

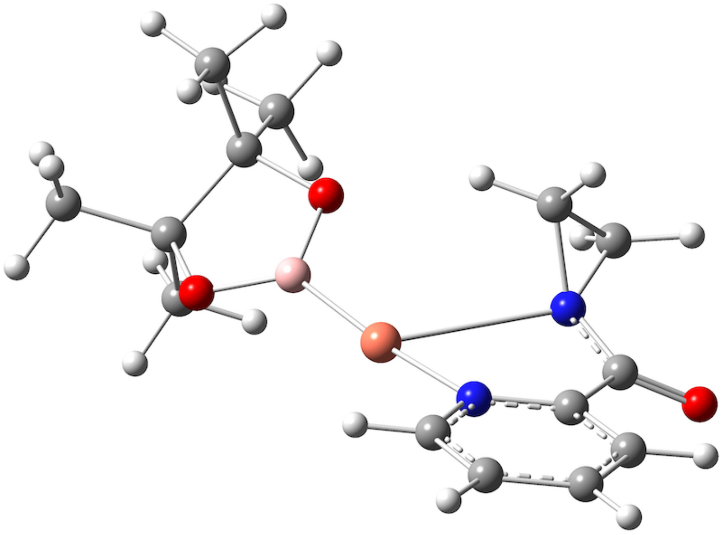

Complex-1    Substrate: N-(2-picolinoyl)-aziridine

CARTESIAN COORDINATES

| | | | |
|---|---|---|---|
| C | 1.073592 | 3.121071 | 0.030476 |
| C | 0.967944 | 2.077211 | 1.102678 |
| H | 1.829370 | 3.899325 | 0.122055 |
| H | 1.661810 | 2.137183 | 1.939928 |
| H | 0.010477 | 1.599264 | 1.294885 |
| N | 1.554787 | 1.777761 | -0.212130 |
| C | 2.881065 | 1.498872 | -0.469347 |
| C | 3.342295 | 0.097202 | -0.134153 |
| C | 4.718755 | -0.127709 | -0.034222 |
| C | 5.181525 | -1.408430 | 0.261987 |
| H | 5.393238 | 0.705256 | -0.197985 |
| C | 2.895256 | -2.132689 | 0.310473 |
| C | 4.250197 | -2.432713 | 0.438812 |
| H | 6.246723 | -1.603245 | 0.349268 |
| H | 2.134557 | -2.898672 | 0.426923 |
| H | 4.558342 | -3.448364 | 0.666356 |
| O | 3.652619 | 2.287585 | -1.003560 |
| N | 2.438565 | -0.896008 | 0.032862 |
| Cu | 0.426010 | -0.740512 | -0.126889 |
| B | -1.525247 | -0.541938 | -0.091173 |
| O | -2.101551 | 0.650941 | 0.372091 |
| O | -2.526913 | -1.425604 | -0.476901 |
| C | -3.538711 | 0.464444 | 0.497597 |
| C | -3.801388 | -0.726596 | -0.488856 |
| C | -4.239570 | 1.772412 | 0.136910 |
| H | -5.329148 | 1.642933 | 0.137015 |
| H | -3.987760 | 2.541770 | 0.875437 |
| H | -3.930534 | 2.135752 | -0.846195 |
| C | -3.815293 | 0.109003 | 1.965746 |
| H | -3.421544 | 0.908090 | 2.603066 |
| H | -4.888589 | 0.005237 | 2.163271 |
| H | -3.313825 | -0.823455 | 2.243546 |
| C | -4.046492 | -0.271783 | -1.935179 |
| H | -4.017364 | -1.149100 | -2.589566 |
| H | -5.022358 | 0.215026 | -2.047620 |
| H | -3.265901 | 0.420759 | -2.265844 |

| | | | |
|---|---|---|---|
| C | -4.898876 | -1.695907 | -0.054680 |
| H | -5.863384 | -1.179249 | 0.028889 |
| H | -5.000870 | -2.492741 | -0.799299 |
| H | -4.665995 | -2.162585 | 0.905510 |
| H | 0.176202 | 3.389377 | -0.521609 |

Electronic Energy = -1103.06390231 (Hartree/Particle)
Dipole Moment (Debye): 4.5991
index 0
Harmonic frequencies
Number of imaginary frequencies= 0

THERMOCHEMICAL DATA

Temperature 298.150 Kelvin. Pressure 1.00000 Atm.
Zero-point correction= 0.333410
(Hartree/Particle)
Thermal correction to Energy= 0.355091
Thermal correction to Enthalpy= 0.356035
Thermal correction to Gibbs Free Energy= 0.280581
Sum of electronic and zero-point Energies= -1102.730493
Sum of electronic and thermal Energies= -1102.708811
Sum of electronic and thermal Enthalpies= -1102.707867
Sum of electronic and thermal Free Energies= -1102.783322
PCM-SP (solvent=THF;6-311+g(2d,p)) = -1103.33107889
Corrected Free Energy = -1103.050498580

-------------------------------------------------------

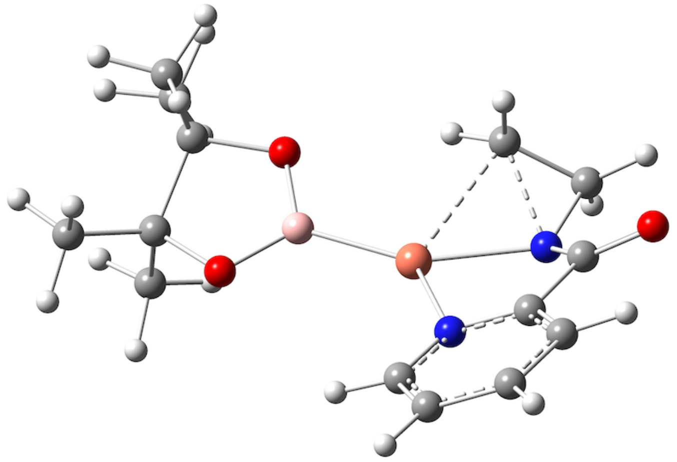

TS1 Substrate: N-(2-picolinoyl)-aziridine

CARTESIAN COORDINATES

| | | | |
|---|---|---|---|
| N | -2.157884 | 1.000066 | -0.271207 |
| C | -3.282602 | 0.341837 | 0.061414 |
| C | -4.442247 | 1.000531 | 0.478829 |
| C | -4.432869 | 2.394943 | 0.519740 |
| C | -3.266031 | 3.078186 | 0.163773 |
| C | -2.143022 | 2.337054 | -0.211243 |
| C | -3.233571 | -1.160401 | -0.000298 |
| O | -4.166339 | -1.846490 | 0.413908 |
| Cu | -0.480548 | -0.345464 | -0.446033 |
| B | 1.450238 | 0.003426 | -0.197391 |
| O | 2.159526 | 1.179032 | -0.428992 |
| C | 3.583943 | 0.903599 | -0.315392 |

| | | | |
|---|---|---|---|
| C | 4.276348 | 2.126322 | 0.282442 |
| N | -2.069422 | -1.661372 | -0.590599 |
| C | -0.856338 | -2.519069 | 0.461252 |
| C | -1.861239 | -3.098972 | -0.451267 |
| O | 2.315644 | -0.984333 | 0.284941 |
| C | 3.603993 | -0.379215 | 0.588232 |
| C | 3.603978 | -0.062970 | 2.090852 |
| C | 4.709767 | -1.381527 | 0.265845 |
| C | 4.103321 | 0.650330 | -1.738112 |
| H | -2.698245 | -3.642021 | -0.012823 |
| H | -1.143856 | -2.341834 | 1.492116 |
| H | 0.205212 | -2.533565 | 0.239113 |
| H | -5.310271 | 0.414869 | 0.761745 |
| H | -5.319565 | 2.941489 | 0.829176 |
| H | -1.199610 | 2.809268 | -0.471470 |
| H | -3.220386 | 4.162887 | 0.179675 |
| H | 5.698638 | -0.925452 | 0.400264 |
| H | 4.636289 | -2.241709 | 0.940425 |
| H | 4.630923 | -1.750805 | -0.759493 |
| H | 3.413284 | -0.987348 | 2.646325 |
| H | 4.566177 | 0.347031 | 2.419107 |
| H | 2.812910 | 0.651217 | 2.340744 |
| H | 3.871665 | 1.522393 | -2.358364 |
| H | 5.187921 | 0.491397 | -1.751616 |
| H | 3.613454 | -0.220675 | -2.184886 |
| H | 5.339399 | 1.921968 | 0.461889 |
| H | 4.203250 | 2.968190 | -0.414632 |
| H | 3.813704 | 2.429522 | 1.224826 |
| H | -1.476275 | -3.555922 | -1.362811 |

Electronic Energy = -1103.03395879 (Hartree/Particle)
Dipole Moment (Debye): 2.2937
index 0
Harmonic frequencies
Number of imaginary frequencies= 1
Negatives Eigenvalues: -360.238

THERMOCHEMICAL DATA

Temperature 298.150 Kelvin. Pressure 1.00000 Atm.
Zero-point correction= 0.331157
(Hartree/Particle)
Thermal correction to Energy= 0.352398
Thermal correction to Enthalpy= 0.353342
Thermal correction to Gibbs Free Energy= 0.278812
Sum of electronic and zero-point Energies= -1102.702801
Sum of electronic and thermal Energies= -1102.681561
Sum of electronic and thermal Enthalpies= -1102.680617
Sum of electronic and thermal Free Energies= -1102.755147
PCM-SP (solvent=THF;6-311+g(2d,p)) = -1103.29923250
Corrected Free Energy = -1103.020420710

---

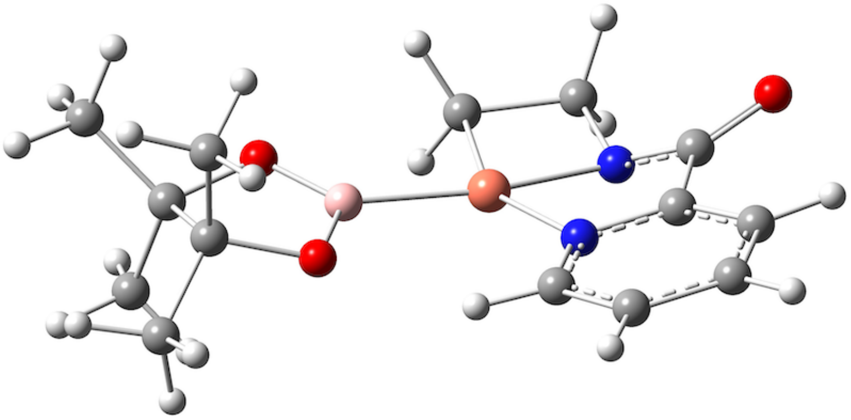

I1    Substrate: N-(2-picolinoyl)-aziridine

CARTESIAN COORDINATES

| | | | |
|---|---|---|---|
| C | 1.909592 | -3.004087 | 0.020938 |
| C | 0.386511 | -2.758070 | 0.011979 |
| H | 2.233571 | -3.753591 | -0.713782 |
| H | -0.165147 | -3.152630 | -0.842693 |
| H | -0.151938 | -2.922717 | 0.945758 |
| N | 2.328969 | -1.650163 | -0.341823 |
| C | 3.349825 | -0.939388 | 0.157533 |
| C | 3.046163 | 0.556562 | 0.008636 |
| C | 4.028876 | 1.532833 | 0.149306 |
| C | 3.663801 | 2.875918 | 0.037545 |
| H | 5.046787 | 1.214574 | 0.349414 |
| C | 1.388163 | 2.175133 | -0.292605 |
| C | 2.324066 | 3.204833 | -0.189247 |
| H | 4.412508 | 3.658083 | 0.132132 |
| H | 0.327480 | 2.353424 | -0.440114 |
| H | 2.002539 | 4.238067 | -0.275959 |
| O | 4.399111 | -1.318212 | 0.692253 |
| N | 1.749757 | 0.887021 | -0.202915 |
| Cu | 0.640164 | -0.823538 | -0.244799 |
| B | -1.267967 | -0.342678 | -0.078820 |
| O | -2.323634 | -1.167325 | 0.225614 |
| O | -1.668631 | 0.969134 | -0.261215 |
| C | -3.560325 | -0.413073 | 0.030472 |
| C | -3.067488 | 1.073846 | 0.161875 |
| C | -4.571263 | -0.853267 | 1.084827 |
| H | -5.482805 | -0.246656 | 1.021760 |
| H | -4.844824 | -1.900149 | 0.917338 |
| H | -4.162992 | -0.769703 | 2.094673 |
| C | -4.069217 | -0.763217 | -1.373460 |
| H | -4.200696 | -1.847745 | -1.441333 |
| H | -5.031044 | -0.282456 | -1.583535 |
| H | -3.350131 | -0.461003 | -2.141598 |
| C | -3.048448 | 1.584130 | 1.607828 |
| H | -2.512997 | 2.538573 | 1.641240 |
| H | -4.062754 | 1.742659 | 1.990324 |
| H | -2.530268 | 0.881695 | 2.268301 |
| C | -3.777644 | 2.074056 | -0.744592 |
| H | -4.849050 | 2.109268 | -0.513147 |
| H | -3.362666 | 3.075429 | -0.587505 |
| H | -3.655424 | 1.819348 | -1.799858 |
| H | 2.293408 | -3.302519 | 1.005583 |

Electronic Energy = -1103.07323400 (Hartree/Particle)
Dipole Moment (Debye): 7.2003
index 0
Harmonic frequencies
Number of imaginary frequencies= 0

THERMOCHEMICAL DATA

Temperature 298.150 Kelvin. Pressure 1.00000 Atm.
Zero-point correction= 0.333209
(Hartree/Particle)
Thermal correction to Energy= 0.354210
Thermal correction to Enthalpy= 0.355154
Thermal correction to Gibbs Free Energy= 0.282281
Sum of electronic and zero-point Energies= -1102.740025
Sum of electronic and thermal Energies= -1102.719024
Sum of electronic and thermal Enthalpies= -1102.718080
Sum of electronic and thermal Free Energies= -1102.790953
PCM-SP (solvent=THF;6-311+g(2d,p)) = -1103.340038680
Corrected Free Energy = -1103.057757680

-------------------------------------------------------

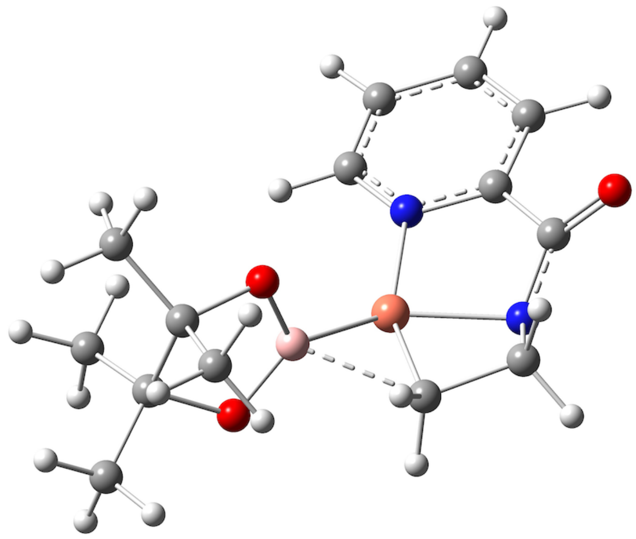

TS2 Substrate: N-(2-picolinoyl)-aziridine

CARTESIAN COORDINATES

| | | | |
|---|---|---|---|
| C | 0.962623 | -2.788739 | -0.216965 |
| Cu | 0.594820 | -0.375960 | -0.588670 |
| B | -1.323467 | -0.250497 | -0.211515 |
| O | -2.284309 | -0.032510 | -1.173965 |
| C | -3.401320 | 0.644253 | -0.515072 |
| C | -4.699844 | 0.189254 | -1.173154 |
| C | -0.391142 | -2.103047 | -0.589507 |
| N | 1.958578 | -1.772640 | -0.577250 |
| C | 2.925178 | -1.397048 | 0.266941 |
| O | 3.527766 | -2.025945 | 1.152619 |
| C | 3.199275 | 0.108823 | 0.121152 |
| C | 4.427952 | 0.679749 | 0.451481 |
| C | 4.582234 | 2.062188 | 0.361629 |
| C | 3.490379 | 2.847039 | -0.025081 |
| C | 2.281458 | 2.214791 | -0.304201 |
| N | 2.139804 | 0.880091 | -0.239955 |

| | | | |
|---|---|---|---|
| O | -1.773573 | -0.012249 | 1.066433 |
| C | -3.220565 | 0.197219 | 0.982384 |
| C | -3.878460 | -1.151757 | 1.295503 |
| C | -3.621766 | 1.238534 | 2.021296 |
| C | -3.193434 | 2.148891 | -0.727391 |
| H | 1.074002 | -3.727796 | -0.779275 |
| H | -0.666301 | -2.268078 | -1.635351 |
| H | -1.214738 | -2.419579 | 0.055693 |
| H | 5.227081 | 0.022723 | 0.778178 |
| H | 5.536497 | 2.526619 | 0.596065 |
| H | 1.394117 | 2.774855 | -0.582911 |
| H | 3.566156 | 3.927445 | -0.097953 |
| H | -4.685142 | 1.488307 | 1.924099 |
| H | -3.453875 | 0.839532 | 3.026935 |
| H | -3.034862 | 2.154320 | 1.920198 |
| H | -3.535957 | -1.493973 | 2.277128 |
| H | -4.970482 | -1.068497 | 1.317318 |
| H | -3.605301 | -1.910864 | 0.555258 |
| H | -3.127550 | 2.348883 | -1.801573 |
| H | -4.025060 | 2.730352 | -0.314995 |
| H | -2.263263 | 2.488171 | -0.259971 |
| H | -5.564788 | 0.606948 | -0.644065 |
| H | -4.730501 | 0.539784 | -2.209985 |
| H | -4.786730 | -0.899634 | -1.182883 |
| H | 1.011118 | -3.031597 | 0.852738 |

Electronic Energy = -1103.06636388 (Hartree/Particle)
Dipole Moment (Debye): 7.3715
index 0
Harmonic frequencies
Number of imaginary frequencies= 1
Negatives Eigenvalues: -116.042

THERMOCHEMICAL DATA

Temperature 298.150 Kelvin. Pressure 1.00000 Atm.
Zero-point correction= 0.332836 (Hartree/Particle)
Thermal correction to Energy= 0.353061
Thermal correction to Enthalpy= 0.354005
Thermal correction to Gibbs Free Energy= 0.284125
Sum of electronic and zero-point Energies= -1102.733528
Sum of electronic and thermal Energies= -1102.713303
Sum of electronic and thermal Enthalpies= -1102.712359
Sum of electronic and thermal Free Energies= -1102.782239
PCM-SP (solvent=THF;6-311+g(2d,p)) = -1103.338300330
Corrected Free Energy = -1103.054175450

---

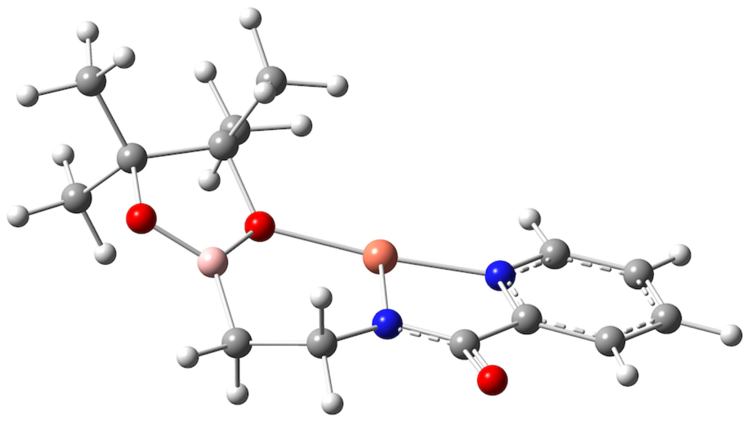

P1    Substrate: N-(2-picolinoyl)-aziridine

CARTESIAN COORDINATES

| | | | |
|---|---|---|---|
| C | -0.233591 | 2.661788 | 0.058186 |
| Cu | -0.503801 | -0.444046 | -0.302499 |
| B | 1.945344 | 1.187104 | -0.328031 |
| O | 1.483527 | -0.146111 | -0.348564 |
| C | 2.508505 | -0.999375 | 0.286224 |
| C | 2.487553 | -2.354599 | -0.406261 |
| C | 1.086935 | 2.440110 | -0.722403 |
| N | -1.070188 | 1.482986 | -0.030576 |
| C | -2.369706 | 1.595940 | 0.220838 |
| O | -3.038189 | 2.607135 | 0.527906 |
| C | -3.116820 | 0.258172 | 0.100111 |
| C | -4.505312 | 0.229460 | 0.253871 |
| C | -5.185741 | -0.981407 | 0.140357 |
| C | -4.459348 | -2.146821 | -0.120693 |
| C | -3.076534 | -2.048854 | -0.254973 |
| N | -2.417599 | -0.880634 | -0.147709 |
| O | 3.264356 | 1.223203 | 0.031669 |
| C | 3.796114 | -0.134402 | 0.060383 |
| C | 4.457745 | -0.376640 | -1.301519 |
| C | 4.828725 | -0.229093 | 1.179374 |
| C | 2.105773 | -1.134897 | 1.756994 |
| H | -0.754038 | 3.545227 | -0.338039 |
| H | 0.824792 | 2.330819 | -1.787373 |
| H | 1.717888 | 3.335213 | -0.646035 |
| H | -5.005901 | 1.169488 | 0.458662 |
| H | -6.266147 | -1.019043 | 0.253938 |
| H | -2.463378 | -2.922346 | -0.453227 |
| H | -4.946130 | -3.112541 | -0.216483 |
| H | 5.186149 | -1.259949 | 1.286770 |
| H | 5.686176 | 0.407955 | 0.940458 |
| H | 4.418175 | 0.103034 | 2.135536 |
| H | 5.218991 | 0.392156 | -1.465429 |
| H | 4.940408 | -1.358965 | -1.342313 |
| H | 3.728028 | -0.310248 | -2.115134 |
| H | 1.094895 | -1.554914 | 1.809956 |
| H | 2.787862 | -1.801131 | 2.294913 |
| H | 2.092665 | -0.162230 | 2.258013 |
| H | 3.276726 | -3.000824 | -0.005821 |
| H | 1.520888 | -2.838056 | -0.225238 |
| H | 2.621137 | -2.260820 | -1.486118 |
| H | -0.004512 | 2.892409 | 1.112568 |

Electronic Energy = -1103.14609975 (Hartree/Particle)
Dipole Moment (Debye): 8.8804
index 0
Harmonic frequencies
Number of imaginary frequencies= 0

THERMOCHEMICAL DATA

Temperature 298.150 Kelvin. Pressure 1.00000 Atm.
Zero-point correction= 0.334744
(Hartree/Particle)
Thermal correction to Energy= 0.355453
Thermal correction to Enthalpy= 0.356397
Thermal correction to Gibbs Free Energy= 0.285303
Sum of electronic and zero-point Energies= -1102.811355
Sum of electronic and thermal Energies= -1102.790647
Sum of electronic and thermal Enthalpies= -1102.789702
Sum of electronic and thermal Free Energies= -1102.860796
PCM-SP (solvent=THF;6-311+g(2d,p)) = -1103.41620638
Corrected Free Energy = -1103.13090263

-------------------------------------------------------

SUBSTRATE: N-(2-PICOLINOYL)-METHYL AZIRIDINE

N-(2-picolinoyl)-methyl aziridine

CARTESIAN COORDINATES

| | | | |
|---|---|---|---|
| N | 0.739112 | 0.955005 | -0.079436 |
| C | 0.776532 | -0.387910 | -0.072773 |
| C | 1.967630 | -1.120861 | 0.008367 |
| C | 3.174191 | -0.425834 | 0.075142 |
| C | 3.145540 | 0.969825 | 0.065820 |
| C | 1.903300 | 1.608771 | -0.009241 |
| C | -0.528476 | -1.147080 | -0.140626 |
| O | -0.556011 | -2.370787 | -0.055804 |
| N | -1.655332 | -0.388689 | -0.382427 |
| C | -2.238070 | 0.636589 | 0.469511 |
| C | -2.633731 | 1.941152 | -0.174604 |
| C | -2.954307 | -0.662022 | 0.196876 |
| H | -1.786307 | 0.724153 | 1.458422 |
| H | 1.921745 | -2.204427 | 0.015446 |
| H | 4.117646 | -0.962213 | 0.133503 |
| H | 1.841136 | 2.695632 | -0.014609 |
| H | 4.059521 | 1.554850 | 0.116273 |
| H | -2.993625 | -1.420789 | 0.977345 |
| H | -3.351531 | 2.479435 | 0.456363 |
| H | -1.745589 | 2.566318 | -0.314562 |
| H | -3.090604 | 1.765469 | -1.154598 |
| H | -3.786413 | -0.672113 | -0.504951 |

Electronic Energy = -533.711901220 (Hartree/Particle)
Dipole Moment (Debye): 3.3143
index 0
Harmonic frequencies
Number of imaginary frequencies= 0

THERMOCHEMICAL DATA

```
 Temperature   298.150 Kelvin.  Pressure   1.00000 Atm.
 Zero-point correction=                           0.177547
(Hartree/Particle)
 Thermal correction to Energy=                    0.188078
 Thermal correction to Enthalpy=                  0.189022
 Thermal correction to Gibbs Free Energy=         0.140571
 Sum of electronic and zero-point Energies=           -533.534355
 Sum of electronic and thermal Energies=              -533.523824
 Sum of electronic and thermal Enthalpies=            -533.522879
 Sum of electronic and thermal Free Energies=         -533.571331
 PCM-SP (solvent=THF;6-311+g(2d,p)) =    -533.855563883
 Corrected Free Energy =  -533.714993663
```

------------------------------------------------------

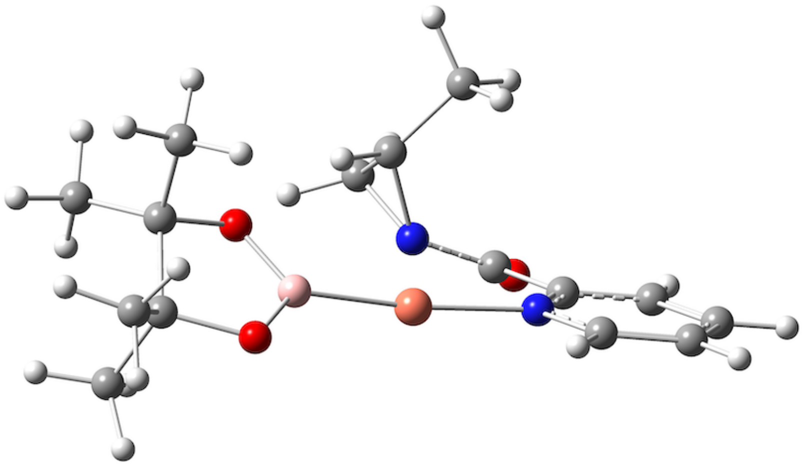

Complex-1    Substrate: N-(2-picolinoyl)-methyl aziridine

CARTESIAN COORDINATES

| | | | |
|---|---|---|---|
| N | 2.310494 | -1.036268 | 0.010075 |
| C | 3.262244 | -0.127749 | -0.305403 |
| C | 4.626386 | -0.418974 | -0.198799 |
| C | 5.025672 | -1.673261 | 0.257416 |
| C | 4.044335 | -2.609506 | 0.586416 |
| C | 2.706040 | -2.250223 | 0.443192 |
| C | 2.883953 | 1.251159 | -0.804598 |
| O | 3.709505 | 1.920604 | -1.419434 |
| Cu | 0.304735 | -0.832843 | -0.135828 |
| B | -1.640870 | -0.585303 | -0.090424 |
| O | -2.662583 | -1.496993 | -0.331330 |
| C | -3.927240 | -0.784768 | -0.414428 |
| C | -5.028670 | -1.666306 | 0.170436 |
| N | 1.586293 | 1.651535 | -0.591680 |
| C | 0.960401 | 2.082498 | 0.674070 |
| C | 1.788387 | 2.110464 | 1.936381 |
| C | 1.211784 | 3.044612 | -0.450602 |
| O | -2.189882 | 0.668535 | 0.216275 |
| C | -3.626402 | 0.525714 | 0.393716 |
| C | -3.876200 | 0.379592 | 1.901905 |
| C | -4.316164 | 1.783470 | -0.129922 |
| C | -4.196769 | -0.528514 | -1.904510 |
| H | -0.057390 | 1.711631 | 0.783576 |
| H | 5.340987 | 0.345318 | -0.482345 |
| H | 6.080548 | -1.915537 | 0.351402 |

```
 H                1.908241   -2.949205    0.675767
 H                4.301056   -3.602262    0.942516
 H               -5.407198    1.673193   -0.092095
 H               -4.038637    2.642411    0.491287
 H               -4.021996    2.003621   -1.158963
 H               -3.457981    1.251935    2.415550
 H               -4.946310    0.321467    2.132494
 H               -3.381556   -0.514088    2.295391
 H               -4.193722   -1.487620   -2.432588
 H               -5.168024   -0.045659   -2.064037
 H               -3.413939    0.099174   -2.342030
 H               -5.983245   -1.125996    0.202097
 H               -5.159265   -2.555385   -0.455893
 H               -4.781123   -2.001528    1.180556
 H                0.377495    3.364651   -1.070886
 H                1.309549    2.758553    2.679661
 H                1.866738    1.105151    2.366697
 H                2.799788    2.490447    1.748244
 H                2.032654    3.755389   -0.359733

 Electronic Energy =  -1142.38831055 (Hartree/Particle)
 Dipole Moment (Debye):      4.6135
 index          0
 Harmonic frequencies
 Number of imaginary frequencies=           0

 THERMOCHEMICAL DATA

 Temperature   298.150 Kelvin.  Pressure   1.00000 Atm.
 Zero-point correction=                          0.361536
(Hartree/Particle)
 Thermal correction to Energy=                   0.384669
 Thermal correction to Enthalpy=                 0.385614
 Thermal correction to Gibbs Free Energy=        0.306949
 Sum of electronic and zero-point Energies=         -1142.026774
 Sum of electronic and thermal Energies=            -1142.003641
 Sum of electronic and thermal Enthalpies=          -1142.002697
 Sum of electronic and thermal Free Energies=       -1142.081362
 PCM-SP (solvent=THF;6-311+g(2d,p)) =   -1142.66700664
 Corrected Free Energy =   -1142.36293795

------------------------------------------------------

PATH: ANTI_MARKOVNIKOV
```

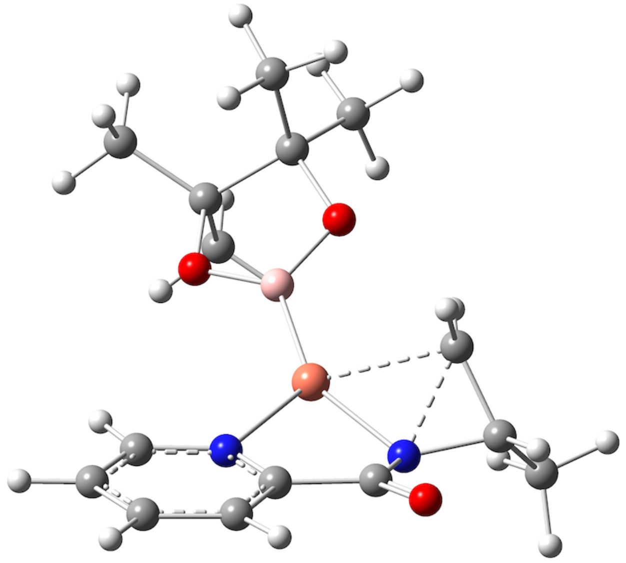

TS1 Substrate: N-(2-picolinoyl)-methyl aziridine
Path: anti-Markovnikov

CARTESIAN COORDINATES

| | | | |
|---|---|---|---|
| N | 2.057018 | -1.213949 | -0.293452 |
| C | 3.209598 | -0.643956 | 0.100543 |
| C | 4.343246 | -1.392129 | 0.428787 |
| C | 4.276012 | -2.780849 | 0.312625 |
| C | 3.079822 | -3.371362 | -0.105903 |
| C | 1.986722 | -2.547088 | -0.384306 |
| C | 3.221174 | 0.857104 | 0.211014 |
| O | 4.188470 | 1.453991 | 0.681304 |
| Cu | 0.435591 | 0.228720 | -0.323142 |
| B | -1.508174 | -0.091876 | -0.147358 |
| O | -2.244819 | -1.213992 | -0.518856 |
| C | -3.663120 | -0.913037 | -0.395126 |
| C | -4.397529 | -2.174613 | 0.053320 |
| N | 2.069033 | 1.464836 | -0.292666 |
| C | 0.895790 | 2.238601 | 0.870009 |
| C | 1.901326 | 2.887832 | 0.001663 |
| O | -2.355983 | 0.860152 | 0.429432 |
| C | -3.664612 | 0.261728 | 0.645159 |
| C | -3.698938 | -0.219037 | 2.103140 |
| C | -4.737825 | 1.324505 | 0.420944 |
| C | -4.151586 | -0.489503 | -1.787999 |
| H | 2.764029 | 3.320390 | 0.510918 |
| H | -0.168345 | 2.315457 | 0.667226 |
| H | 5.236467 | -0.877943 | 0.766940 |
| H | 5.140930 | -3.394545 | 0.549349 |
| H | 1.023343 | -2.947967 | -0.687820 |
| H | 2.989082 | -4.448192 | -0.211605 |
| H | -5.740765 | 0.884820 | 0.490109 |
| H | -4.652681 | 2.102194 | 1.187967 |
| H | -4.631799 | 1.802836 | -0.555729 |
| H | -3.493398 | 0.632388 | 2.760586 |
| H | -4.677151 | -0.635567 | 2.370185 |
| H | -2.931390 | -0.978476 | 2.283217 |
| H | -3.930772 | -1.293068 | -2.498224 |
| H | -5.231568 | -0.301619 | -1.799482 |
| H | -3.632813 | 0.412551 | -2.127718 |

| | | | |
|---|---|---|---|
| H | -5.457655 | -1.961603 | 0.239912 |
| H | -4.335125 | -2.935687 | -0.732114 |
| H | -3.958890 | -2.593440 | 0.962307 |
| H | 1.189671 | 1.931727 | 1.868194 |
| C | 1.399719 | 3.709539 | -1.169951 |
| H | 1.070017 | 4.697261 | -0.826731 |
| H | 2.197783 | 3.845043 | -1.908044 |
| H | 0.557262 | 3.206102 | -1.657275 |

Electronic Energy = -1142.35837407 (Hartree/Particle)
Dipole Moment (Debye): 2.0990
index 0
Harmonic frequencies
Number of imaginary frequencies= 1
Negatives Eigenvalues: -354.351

THERMOCHEMICAL DATA

Temperature 298.150 Kelvin. Pressure 1.00000 Atm.
Zero-point correction= 0.359037
(Hartree/Particle)
Thermal correction to Energy= 0.381795
Thermal correction to Enthalpy= 0.382739
Thermal correction to Gibbs Free Energy= 0.305011
Sum of electronic and zero-point Energies= -1141.999337
Sum of electronic and thermal Energies= -1141.976579
Sum of electronic and thermal Enthalpies= -1141.975635
Sum of electronic and thermal Free Energies= -1142.053363
PCM-SP (solvent=THF;6-311+g(2d,p)) = -1142.63449331
Corrected Free Energy = -1142.32948224

------------------------------------------------------

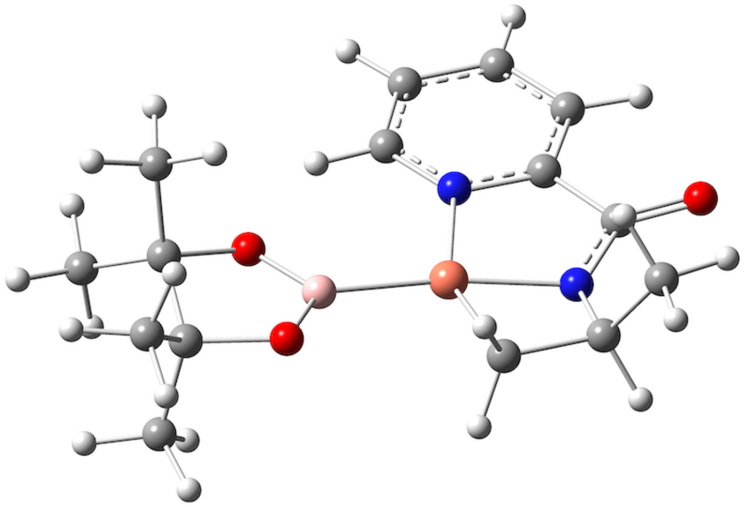

I1 Substrate: N-(2-picolinoyl)-methyl aziridine
Path: anti-Markovnikov

CARTESIAN COORDINATES

| | | | |
|---|---|---|---|
| N | 1.558320 | 1.183179 | -0.137824 |
| C | 2.892995 | 0.955679 | -0.070341 |
| C | 3.800082 | 2.004675 | 0.049651 |
| C | 3.316521 | 3.314496 | 0.084419 |
| C | 1.938985 | 3.537163 | 0.009527 |

| | | | |
|---|---|---|---|
| C | 1.086008 | 2.437365 | -0.095070 |
| C | 3.350771 | -0.510453 | -0.091974 |
| O | 4.528727 | -0.806044 | 0.150037 |
| Cu | 0.571913 | -0.597708 | -0.258717 |
| N | 2.304409 | -1.280140 | -0.399751 |
| C | 2.014522 | -2.704292 | -0.247622 |
| C | 0.471312 | -2.563756 | -0.245167 |
| C | 2.586320 | -3.325828 | 1.026405 |
| B | -1.371635 | -0.280073 | -0.099900 |
| O | -2.373595 | -1.215635 | 0.002134 |
| C | -3.655752 | -0.526247 | -0.122240 |
| C | -4.670108 | -1.214962 | 0.785734 |
| O | -1.867142 | 1.012749 | -0.071536 |
| C | -3.284350 | 0.944106 | 0.290936 |
| C | -3.360874 | 1.190095 | 1.802701 |
| C | -4.034243 | 2.037849 | -0.462851 |
| C | -4.082116 | -0.655937 | -1.590002 |
| H | 2.367570 | -3.271856 | -1.122082 |
| H | -0.054371 | -2.890488 | -1.143845 |
| H | -0.043749 | -2.887974 | 0.660911 |
| H | 4.856061 | 1.763882 | 0.117117 |
| H | 4.004299 | 4.151525 | 0.172076 |
| H | 0.005414 | 2.530999 | -0.141131 |
| H | 1.525581 | 4.540569 | 0.036755 |
| H | 2.282759 | -4.377172 | 1.107892 |
| H | 3.678731 | -3.265251 | 1.025037 |
| H | -5.622435 | -0.670917 | 0.782363 |
| H | -4.855772 | -2.231861 | 0.424701 |
| H | -4.308788 | -1.283996 | 1.814404 |
| H | -4.127681 | -1.717681 | -1.852232 |
| H | -5.069008 | -0.212381 | -1.762420 |
| H | -3.358991 | -0.173973 | -2.255788 |
| H | -2.901324 | 2.157690 | 2.029270 |
| H | -4.398478 | 1.208523 | 2.153929 |
| H | -2.816594 | 0.418707 | 2.356656 |
| H | -5.113366 | 1.957349 | -0.284642 |
| H | -3.704163 | 3.021685 | -0.112399 |
| H | -3.851982 | 1.983601 | -1.538639 |
| H | 2.215522 | -2.786634 | 1.907685 |

Electronic Energy = -1142.39531716 (Hartree/Particle)
Dipole Moment (Debye): 6.9198
index 0
Harmonic frequencies
Number of imaginary frequencies= 0

THERMOCHEMICAL DATA

Temperature 298.150 Kelvin. Pressure 1.00000 Atm.
Zero-point correction= 0.361253 (Hartree/Particle)
Thermal correction to Energy= 0.383694
Thermal correction to Enthalpy= 0.384638
Thermal correction to Gibbs Free Energy= 0.308618
Sum of electronic and zero-point Energies= -1142.034064
Sum of electronic and thermal Energies= -1142.011624
Sum of electronic and thermal Enthalpies= -1142.010679
Sum of electronic and thermal Free Energies= -1142.086699
PCM-SP (solvent=THF;6-311+g(2d,p)) = -1142.67196072
Corrected Free Energy = -1142.36334256

-------------------------------------------------------

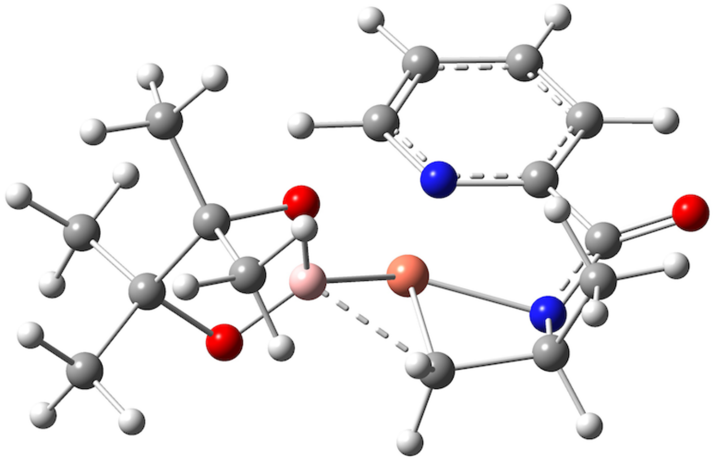

TS2 Substrate: N-(2-picolinoyl)-methyl aziridine
Path: anti-Markovnikov

CARTESIAN COORDINATES

| | | | |
|---|---|---|---|
| N | -2.099117 | -1.086394 | -0.150624 |
| C | -3.189198 | -0.299227 | 0.056007 |
| C | -4.424153 | -0.854772 | 0.389819 |
| C | -4.550240 | -2.240614 | 0.474401 |
| C | -3.426625 | -3.042189 | 0.248390 |
| C | -2.216450 | -2.421327 | -0.051646 |
| C | -2.977765 | 1.228833 | 0.010769 |
| O | -3.753701 | 1.949791 | 0.661220 |
| Cu | -0.549437 | 0.123497 | -0.618419 |
| N | -1.904558 | 1.506996 | -0.729222 |
| C | -0.917532 | 2.577166 | -0.519454 |
| C | 0.413771 | 1.841702 | -0.907374 |
| C | -0.873951 | 3.122477 | 0.911475 |
| B | 1.384831 | 0.111068 | -0.281556 |
| O | 1.854710 | 0.070601 | 1.012327 |
| C | 3.303334 | -0.128048 | 0.940417 |
| C | 3.733496 | -0.998493 | 2.116039 |
| O | 2.339708 | -0.233359 | -1.213665 |
| C | 3.474624 | -0.788506 | -0.477284 |
| C | 3.289744 | -2.311008 | -0.462182 |
| C | 4.758210 | -0.416567 | -1.212433 |
| C | 3.942779 | 1.261798 | 1.042289 |
| H | -1.069028 | 3.415334 | -1.219265 |
| H | 0.592981 | 1.849057 | -1.987057 |
| H | 1.284047 | 2.264509 | -0.397959 |
| H | -5.250292 | -0.178248 | 0.581942 |
| H | -5.507843 | -2.693081 | 0.718626 |
| H | -1.307315 | -2.991774 | -0.215253 |
| H | -3.478964 | -4.124555 | 0.312549 |
| H | -0.092945 | 3.887571 | 1.005567 |
| H | -1.839403 | 3.554115 | 1.186436 |
| H | 4.799680 | -1.244375 | 2.042528 |
| H | 3.570372 | -0.457800 | 3.054020 |
| H | 3.160582 | -1.927620 | 2.159061 |
| H | 3.606788 | 1.739863 | 1.967776 |
| H | 5.036226 | 1.198345 | 1.059677 |

| | | | |
|---|---|---|---|
| H | 3.648100 | 1.899754 | 0.202682 |
| H | 3.215377 | -2.668647 | -1.494072 |
| H | 4.135435 | -2.812675 | 0.020473 |
| H | 2.370420 | -2.591023 | 0.062392 |
| H | 5.635892 | -0.739280 | -0.639623 |
| H | 4.782848 | -0.915206 | -2.186902 |
| H | 4.827191 | 0.660194 | -1.383218 |
| H | -0.650888 | 2.310963 | 1.616027 |

Electronic Energy = -1142.38693333 (Hartree/Particle)
Dipole Moment (Debye): 7.2355
index 0
Harmonic frequencies
Number of imaginary frequencies= 1
Negatives Eigenvalues: -111.628

THERMOCHEMICAL DATA

Temperature 298.150 Kelvin. Pressure 1.00000 Atm.
Zero-point correction= 0.360854
(Hartree/Particle)
Thermal correction to Energy= 0.382440
Thermal correction to Enthalpy= 0.383384
Thermal correction to Gibbs Free Energy= 0.310726
Sum of electronic and zero-point Energies= -1142.026079
Sum of electronic and thermal Energies= -1142.004493
Sum of electronic and thermal Enthalpies= -1142.003549
Sum of electronic and thermal Free Energies= -1142.076207
PCM-SP (solvent=THF;6-311+g(2d,p)) = -1142.66836204
Corrected Free Energy = -1142.36073507

------------------------------------------------------

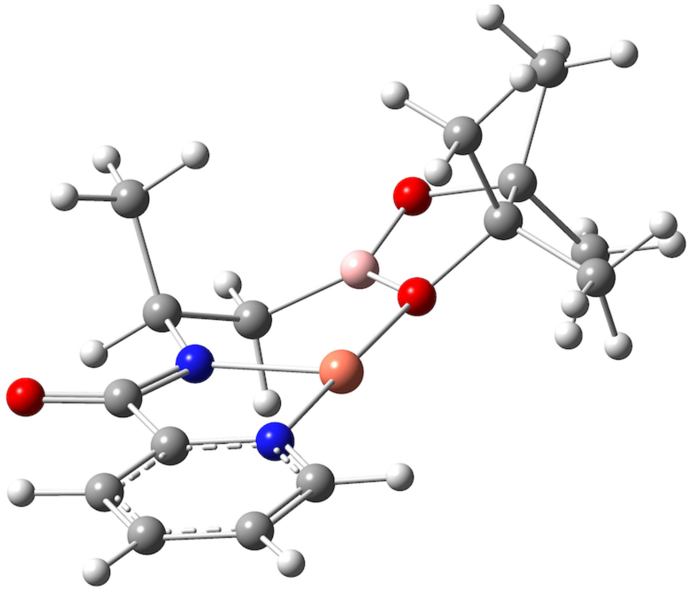

P1 Substrate: N-(2-picolinoyl)-methyl aziridine
Path: anti-Markovnikov

CARTESIAN COORDINATES

| | | | |
|---|---|---|---|
| N | -2.430702 | -1.023142 | -0.088177 |
| C | -3.131893 | 0.138097 | -0.005961 |

| | | | |
|---|---|---|---|
| C | -4.523968 | 0.130434 | 0.112516 |
| C | -5.206048 | -1.084646 | 0.136912 |
| C | -4.477552 | -2.274195 | 0.046959 |
| C | -3.091251 | -2.195014 | -0.062312 |
| C | -2.381755 | 1.479420 | -0.032352 |
| O | -3.052990 | 2.521286 | 0.137838 |
| Cu | -0.511750 | -0.610163 | -0.260342 |
| N | -1.077708 | 1.334187 | -0.240736 |
| C | -0.225451 | 2.511176 | -0.246389 |
| C | 1.063838 | 2.192484 | -1.052907 |
| B | 1.938104 | 0.995376 | -0.536323 |
| O | 3.268806 | 1.068751 | -0.230507 |
| C | 3.796821 | -0.276206 | -0.034955 |
| C | 4.862674 | -0.226162 | 1.055373 |
| C | 0.104355 | 2.954692 | 1.189603 |
| O | 1.472996 | -0.325976 | -0.366695 |
| C | 2.514240 | -1.093757 | 0.344782 |
| C | 2.155532 | -1.026673 | 1.831667 |
| C | 2.467758 | -2.529635 | -0.157792 |
| C | 4.415470 | -0.705233 | -1.370664 |
| H | -0.746393 | 3.343164 | -0.742667 |
| H | 0.758121 | 1.957141 | -2.084568 |
| H | 1.696093 | 3.088095 | -1.108554 |
| H | -5.025992 | 1.089198 | 0.182787 |
| H | -6.289211 | -1.106870 | 0.225514 |
| H | -2.476660 | -3.087101 | -0.130992 |
| H | -4.965426 | -3.243972 | 0.063215 |
| H | 0.768055 | 3.829355 | 1.195369 |
| H | -0.819060 | 3.210379 | 1.718555 |
| H | 5.220498 | -1.235474 | 1.290428 |
| H | 5.714138 | 0.367179 | 0.707376 |
| H | 4.481916 | 0.234725 | 1.969535 |
| H | 5.174238 | 0.028396 | -1.659600 |
| H | 4.892882 | -1.687891 | -1.292769 |
| H | 3.661404 | -0.743330 | -2.163621 |
| H | 1.144210 | -1.425890 | 1.969325 |
| H | 2.849635 | -1.620878 | 2.434656 |
| H | 2.162118 | 0.004583 | 2.197190 |
| H | 3.267255 | -3.121558 | 0.301759 |
| H | 1.505799 | -2.977505 | 0.116094 |
| H | 2.567744 | -2.582921 | -1.244035 |
| H | 0.600620 | 2.140521 | 1.736950 |

Electronic Energy = -1142.46838562 (Hartree/Particle)
Dipole Moment (Debye): 8.6974
index 0
Harmonic frequencies
Number of imaginary frequencies= 0

THERMOCHEMICAL DATA

Temperature 298.150 Kelvin. Pressure 1.00000 Atm.
Zero-point correction= 0.362904 (Hartree/Particle)
Thermal correction to Energy= 0.384972
Thermal correction to Enthalpy= 0.385916
Thermal correction to Gibbs Free Energy= 0.312272
Sum of electronic and zero-point Energies= -1142.105482
Sum of electronic and thermal Energies= -1142.083414
Sum of electronic and thermal Enthalpies= -1142.082470

Sum of electronic and thermal Free Energies= -1142.156113
PCM-SP (solvent=THF;6-311+g(2d,p)) = -1142.74867388
Corrected Free Energy = -1142.43640126

---

PATH: MARKOVNIKOV

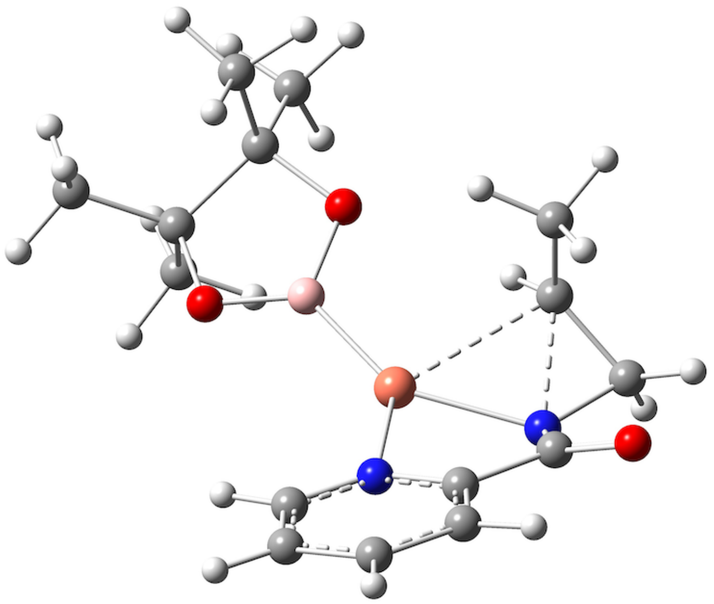

TS1 Substrate: N-(2-picolinoyl)-methyl aziridine
Path: Markovnikov

CARTESIAN COORDINATES

| | | | |
|---|---|---|---|
| N | -2.155983 | 1.164611 | -0.261196 |
| C | -3.255037 | 0.437938 | 0.010750 |
| C | -4.431410 | 1.013409 | 0.498589 |
| C | -4.469085 | 2.396173 | 0.678069 |
| C | -3.329482 | 3.151195 | 0.384191 |
| C | -2.186756 | 2.489596 | -0.069926 |
| C | -3.158712 | -1.050446 | -0.200301 |
| O | -4.062352 | -1.799143 | 0.179462 |
| Cu | -0.443061 | -0.074003 | -0.605668 |
| B | 1.491177 | 0.187544 | -0.292179 |
| O | 2.274575 | 1.328513 | -0.442721 |
| C | 3.675534 | 0.970522 | -0.285141 |
| C | 4.406916 | 2.127327 | 0.392339 |
| N | -2.007052 | -1.444339 | -0.865368 |
| C | -0.690391 | -2.442545 | 0.018735 |
| C | -1.757598 | -2.883471 | -0.901216 |
| O | 2.285781 | -0.867668 | 0.180181 |
| C | 3.589403 | -0.347455 | 0.561679 |
| C | 3.540891 | -0.093772 | 2.075484 |
| C | 4.653685 | -1.394646 | 0.242090 |
| C | 4.240456 | 0.747627 | -1.695597 |
| H | -2.559515 | -3.491203 | -0.478258 |
| H | 0.320958 | -2.342076 | -0.361461 |
| H | -5.276327 | 0.372593 | 0.726269 |

| | | | |
|---|---|---|---|
| H | -5.370782 | 2.878399 | 1.045772 |
| H | -1.262994 | 3.018037 | -0.288909 |
| H | -3.319561 | 4.229887 | 0.507728 |
| H | 5.658695 | -0.999972 | 0.437346 |
| H | 4.505219 | -2.276966 | 0.874601 |
| H | 4.600584 | -1.715858 | -0.800973 |
| H | 3.278119 | -1.028643 | 2.582139 |
| H | 4.507909 | 0.249334 | 2.461226 |
| H | 2.778525 | 0.651827 | 2.322975 |
| H | 4.080776 | 1.655441 | -2.286493 |
| H | 5.314914 | 0.531068 | -1.672799 |
| H | 3.724733 | -0.076581 | -2.198568 |
| H | 5.449052 | 1.857292 | 0.604780 |
| H | 4.408148 | 2.999951 | -0.269800 |
| H | 3.921886 | 2.416824 | 1.327773 |
| H | -1.436499 | -3.227891 | -1.885476 |
| C | -0.851407 | -2.493092 | 1.503824 |
| H | -0.523145 | -3.489000 | 1.842080 |
| H | -0.219737 | -1.745465 | 1.990818 |
| H | -1.892912 | -2.364325 | 1.813650 |

Electronic Energy = -1142.35990393 (Hartree/Particle)
Dipole Moment (Debye): 2.0494
index 0
Harmonic frequencies
Number of imaginary frequencies= 1
Negatives Eigenvalues: -305.921

THERMOCHEMICAL DATA

Temperature 298.150 Kelvin. Pressure 1.00000 Atm.
Zero-point correction= 0.359211
(Hartree/Particle)
Thermal correction to Energy= 0.381992
Thermal correction to Enthalpy= 0.382936
Thermal correction to Gibbs Free Energy= 0.305466
Sum of electronic and zero-point Energies= -1142.000693
Sum of electronic and thermal Energies= -1141.977912
Sum of electronic and thermal Enthalpies= -1141.976968
Sum of electronic and thermal Free Energies= -1142.054438
PCM-SP (solvent=THF;6-311+g(2d,p)) = -1142.63626637
Corrected Free Energy = -1142.33080044

-------------------------------------------------------

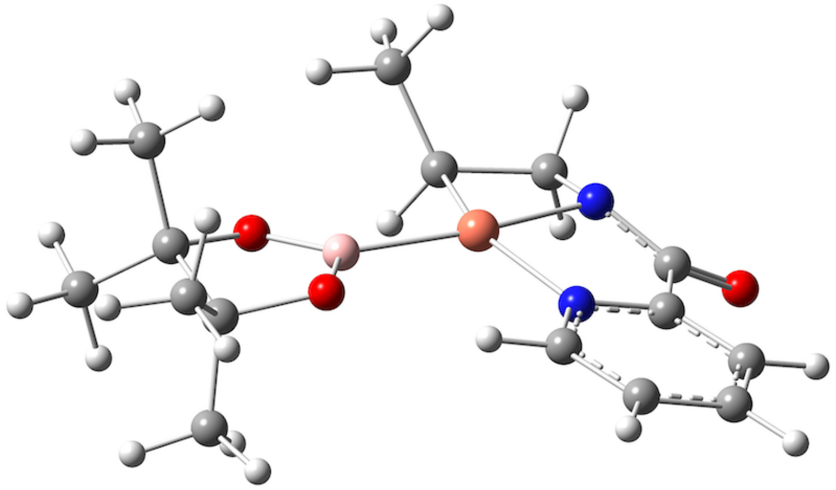

I1 Substrate: N-(2-picolinoyl)-methyl aziridine
Path: Markovnikov

CARTESIAN COORDINATES

| | | | |
|---|---|---|---|
| N | 1.781262 | 1.010488 | -0.223980 |
| C | 3.074491 | 0.684617 | 0.007474 |
| C | 4.067995 | 1.658077 | 0.074952 |
| C | 3.715615 | 2.993253 | -0.131877 |
| C | 2.378209 | 3.318020 | -0.377542 |
| C | 1.432343 | 2.292247 | -0.404530 |
| C | 3.363220 | -0.797960 | 0.264021 |
| O | 4.412765 | -1.150064 | 0.816567 |
| Cu | 0.644335 | -0.702321 | -0.143585 |
| N | 2.331275 | -1.535701 | -0.169947 |
| C | 1.922238 | -2.856130 | 0.296693 |
| C | 0.392902 | -2.642335 | 0.228956 |
| B | -1.256414 | -0.176246 | -0.004991 |
| O | -1.638556 | 1.134630 | -0.237266 |
| C | -3.037486 | 1.277722 | 0.169624 |
| C | -3.724191 | 2.255943 | -0.778259 |
| O | -2.332561 | -0.969884 | 0.322414 |
| C | -3.554197 | -0.204072 | 0.088043 |
| C | -4.055762 | -0.597311 | -1.307324 |
| C | -4.582386 | -0.588821 | 1.147437 |
| C | -3.022051 | 1.839956 | 1.596427 |
| H | 2.265220 | -3.664990 | -0.365371 |
| H | -0.123451 | -2.699374 | 1.189599 |
| H | 5.083474 | 1.344933 | 0.294580 |
| H | 4.472152 | 3.772793 | -0.095722 |
| H | 0.372725 | 2.470379 | -0.560366 |
| H | 2.066250 | 4.345593 | -0.536809 |
| H | -4.796823 | 2.316801 | -0.557901 |
| H | -3.294189 | 3.255545 | -0.653551 |
| H | -3.597246 | 1.961565 | -1.822634 |
| H | -2.469392 | 2.785103 | 1.599884 |
| H | -4.036459 | 2.030516 | 1.963909 |
| H | -2.521972 | 1.152962 | 2.286418 |
| H | -4.206349 | -1.681214 | -1.336671 |
| H | -5.006949 | -0.108577 | -1.545598 |
| H | -3.323806 | -0.336337 | -2.078435 |
| H | -5.483231 | 0.029652 | 1.053148 |

| | | | |
|---|---|---|---|
| H | -4.871792 | -1.636651 | 1.015852 |
| H | -4.182286 | -0.474716 | 2.157564 |
| C | -0.338336 | -3.355341 | -0.887260 |
| H | -0.309807 | -4.443298 | -0.708704 |
| H | -1.383670 | -3.041012 | -0.941695 |
| H | 0.139304 | -3.172187 | -1.856684 |
| H | 2.288072 | -3.067114 | 1.310852 |

Electronic Energy = -1142.39413538 (Hartree/Particle)
Dipole Moment (Debye): 6.9373
index 0
Harmonic frequencies
Number of imaginary frequencies= 0

THERMOCHEMICAL DATA

Temperature 298.150 Kelvin. Pressure 1.00000 Atm.
Zero-point correction= 0.361533
(Hartree/Particle)
Thermal correction to Energy= 0.383998
Thermal correction to Enthalpy= 0.384942
Thermal correction to Gibbs Free Energy= 0.309154
Sum of electronic and zero-point Energies= -1142.032603
Sum of electronic and thermal Energies= -1142.010138
Sum of electronic and thermal Enthalpies= -1142.009193
Sum of electronic and thermal Free Energies= -1142.084981
PCM-SP (solvent=THF;6-311+g(2d,p)) = -1142.67134855
Corrected Free Energy = -1142.36219417

-------------------------------------------------------

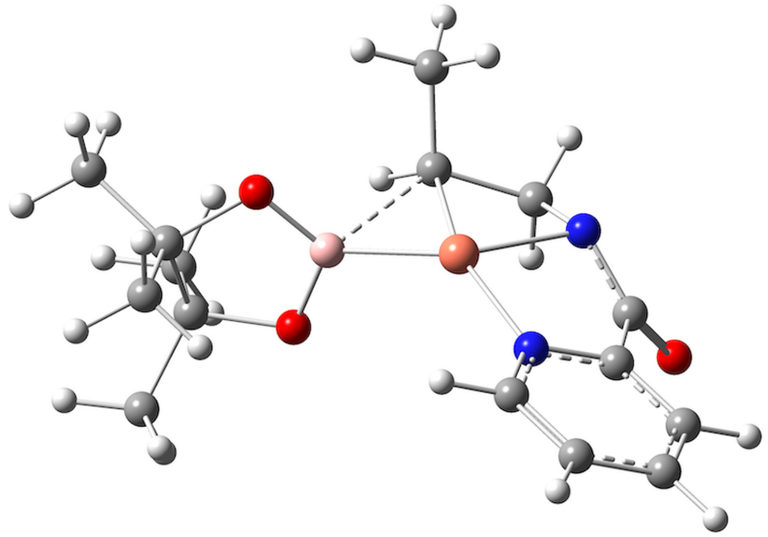

TS2 Substrate: N-(2-picolinoyl)-methyl aziridine
Path: Markovnikov

CARTESIAN COORDINATES

| | | | |
|---|---|---|---|
| N | 2.197798 | 0.892005 | -0.362836 |
| C | 3.198238 | 0.175305 | 0.214581 |
| C | 4.433076 | 0.758932 | 0.497797 |
| C | 4.658491 | 2.085599 | 0.135169 |
| C | 3.627854 | 2.811491 | -0.472361 |
| C | 2.407034 | 2.178577 | -0.690584 |

| | | | |
|---|---|---|---|
| C | 2.842468 | -1.254275 | 0.652846 |
| O | 3.347361 | -1.691370 | 1.701168 |
| Cu | 0.618250 | -0.356492 | -0.554583 |
| N | 1.921203 | -1.774863 | -0.163616 |
| C | 0.850871 | -2.639019 | 0.346213 |
| C | -0.434056 | -2.056761 | -0.327910 |
| B | -1.295602 | -0.162580 | -0.151490 |
| O | -1.667236 | 0.237534 | 1.112623 |
| C | -3.106181 | 0.502939 | 1.078066 |
| C | -3.408221 | 1.667716 | 2.014421 |
| O | -2.299057 | -0.002935 | -1.082742 |
| C | -3.349354 | 0.793173 | -0.448676 |
| C | -3.090904 | 2.254397 | -0.835925 |
| C | -4.699138 | 0.330852 | -0.987729 |
| C | -3.800493 | -0.772542 | 1.569988 |
| H | 0.963405 | -3.697714 | 0.062203 |
| H | -1.310565 | -2.235578 | 0.303881 |
| H | 5.180750 | 0.156677 | 1.003055 |
| H | 5.620031 | 2.554086 | 0.328265 |
| H | 1.563835 | 2.700184 | -1.133124 |
| H | 3.759211 | 3.849744 | -0.760751 |
| H | -4.464339 | 1.954474 | 1.943393 |
| H | -3.201834 | 1.372307 | 3.048245 |
| H | -2.790870 | 2.538956 | 1.783920 |
| H | -3.419221 | -1.022602 | 2.564968 |
| H | -4.885481 | -0.637026 | 1.636313 |
| H | -3.599374 | -1.619112 | 0.905280 |
| H | -3.076260 | 2.334172 | -1.927581 |
| H | -3.873570 | 2.915688 | -0.448770 |
| H | -2.122979 | 2.597224 | -0.456071 |
| H | -5.516437 | 0.844327 | -0.467287 |
| H | -4.770880 | 0.566686 | -2.054572 |
| H | -4.831798 | -0.747216 | -0.871076 |
| C | -0.662941 | -2.600597 | -1.738514 |
| H | -0.907737 | -3.671065 | -1.676595 |
| H | -1.482815 | -2.085735 | -2.246187 |
| H | 0.243127 | -2.505330 | -2.346411 |
| H | 0.790558 | -2.592550 | 1.441737 |

Electronic Energy = -1142.38486250 (Hartree/Particle)
Dipole Moment (Debye): 7.2577
index 0
Harmonic frequencies
Number of imaginary frequencies= 1
Negatives Eigenvalues: -112.199

THERMOCHEMICAL DATA

Temperature 298.150 Kelvin. Pressure 1.00000 Atm.
Zero-point correction= 0.361217
(Hartree/Particle)
Thermal correction to Energy= 0.382847
Thermal correction to Enthalpy= 0.383792
Thermal correction to Gibbs Free Energy= 0.311286
Sum of electronic and zero-point Energies= -1142.023646
Sum of electronic and thermal Energies= -1142.002015
Sum of electronic and thermal Enthalpies= -1142.001071
Sum of electronic and thermal Free Energies= -1142.073576
PCM-SP (solvent=THF;6-311+g(2d,p)) = -1142.66758797
Corrected Free Energy = -1142.35630147

---

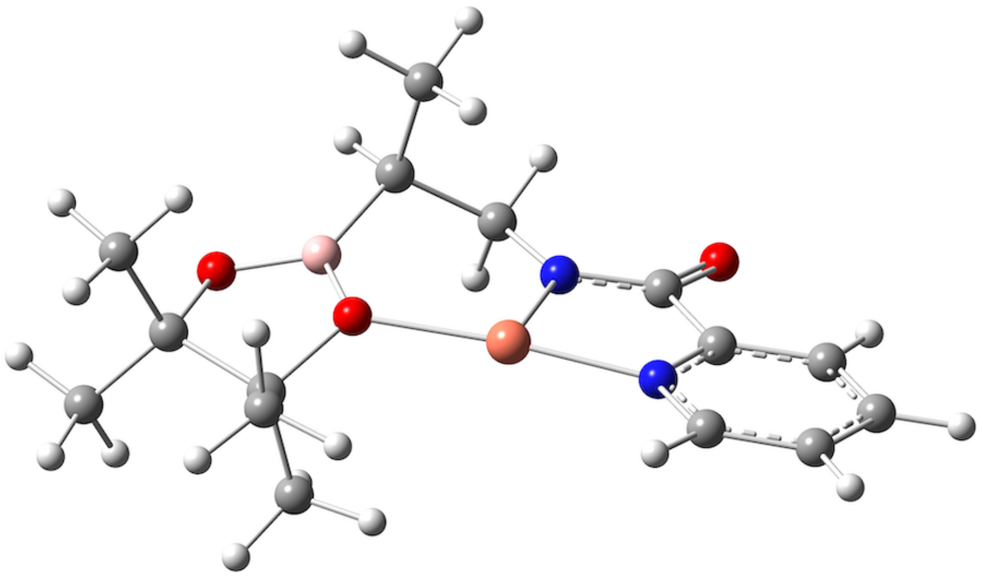

P1 Substrate: N-(2-picolinoyl)-methyl aziridine
Path: Markovnikov

CARTESIAN COORDINATES

| | | | |
|---|---|---|---|
| N | -2.462138 | -0.933162 | -0.196503 |
| C | -3.147293 | 0.170975 | 0.202296 |
| C | -4.538010 | 0.143763 | 0.335079 |
| C | -5.234626 | -1.028245 | 0.046757 |
| C | -4.522368 | -2.157349 | -0.367803 |
| C | -3.136845 | -2.063903 | -0.473685 |
| C | -2.383088 | 1.468221 | 0.513537 |
| O | -3.046923 | 2.447482 | 0.918982 |
| Cu | -0.540834 | -0.512883 | -0.265586 |
| N | -1.078407 | 1.359916 | 0.293391 |
| C | -0.221475 | 2.501601 | 0.534061 |
| C | 1.087881 | 2.388316 | -0.291952 |
| B | 1.921755 | 1.078373 | -0.042335 |
| O | 3.235444 | 1.049060 | 0.336795 |
| C | 3.757429 | -0.301797 | 0.164849 |
| C | 4.778845 | -0.573257 | 1.264823 |
| O | 1.449923 | -0.232407 | -0.264015 |
| C | 2.461582 | -1.180086 | 0.245879 |
| C | 2.042322 | -1.530670 | 1.675855 |
| C | 2.438598 | -2.415554 | -0.642611 |
| C | 4.429007 | -0.340404 | -1.213073 |
| H | -0.734548 | 3.440300 | 0.273603 |
| H | 1.732240 | 3.232266 | -0.007167 |
| H | -5.027223 | 1.054586 | 0.662571 |
| H | -6.316780 | -1.063835 | 0.143078 |
| H | -2.534248 | -2.910350 | -0.787750 |
| H | -5.022140 | -3.092008 | -0.603130 |
| H | 5.128124 | -1.611418 | 1.217970 |
| H | 5.642863 | 0.085515 | 1.132087 |
| H | 4.361621 | -0.386186 | 2.256823 |
| H | 5.198038 | 0.437073 | -1.252523 |
| H | 4.903597 | -1.309731 | -1.399416 |

```
  H              3.706742   -0.143908   -2.012232
  H              1.028291   -1.946010    1.656138
  H              2.714420   -2.275183    2.114572
  H              2.030168   -0.643613    2.316331
  H              3.219173   -3.121225   -0.336810
  H              1.466793   -2.912657   -0.545439
  H              2.583985   -2.161273   -1.694706
  C              0.781780    2.504452   -1.806533
  H              0.275973    3.454969   -2.019160
  H              1.692756    2.463956   -2.418099
  H              0.114164    1.696643   -2.127165
  H              0.025221    2.577079    1.606895

 Electronic Energy =  -1142.46291962 (Hartree/Particle)
 Dipole Moment (Debye):      8.8624
 index          0
 Harmonic frequencies
 Number of imaginary frequencies=           0

 THERMOCHEMICAL DATA

 Temperature   298.150 Kelvin.  Pressure   1.00000 Atm.
 Zero-point correction=                           0.363179
(Hartree/Particle)
 Thermal correction to Energy=                    0.385327
 Thermal correction to Enthalpy=                  0.386271
 Thermal correction to Gibbs Free Energy=         0.312250
 Sum of electronic and zero-point Energies=          -1142.099741
 Sum of electronic and thermal Energies=             -1142.077593
 Sum of electronic and thermal Enthalpies=           -1142.076649
 Sum of electronic and thermal Free Energies=        -1142.150669
 PCM-SP (solvent=THF;6-311+g(2d,p)) =  -1142.74378919
 Corrected Free Energy =   -1142.43153857

------------------------------------------------------

SUBSTRATE: 1c

1c

 CARTESIAN COORDINATES

  C             -2.972280   -1.942506    0.233467
  C             -2.302083   -1.440191   -1.017106
  H             -2.352174   -2.428390    0.984127
  N             -2.730748   -0.529033    0.026683
  C             -1.957973    0.237868    0.883219
  C             -1.100412    1.301607    0.244185
  C             -0.292353    2.086294    1.076206
  C              0.531914    3.046001    0.492930
  H             -0.322380    1.922697    2.147550
  C             -0.327695    2.358978   -1.639032
  C              0.516080    3.189400   -0.895436
  H              1.178301    3.666001    1.108385
  H             -0.367072    2.440565   -2.723955
  H              1.143062    3.921775   -1.395971
  O             -2.018738    0.119198    2.101652
  N             -1.120283    1.430622   -1.092205
  H             -4.012749   -2.259073    0.197053
  C             -0.830943   -1.676609   -1.284332
```

| | | | |
|---|---|---|---|
| H | -0.456285 | -0.868044 | -1.921453 |
| H | -0.695841 | -2.638422 | -1.800997 |
| O | -0.146524 | -1.683352 | -0.037266 |
| C | 1.177907 | -1.335288 | 0.017545 |
| C | 1.653409 | -0.974033 | 1.285618 |
| C | 2.036614 | -1.337186 | -1.087131 |
| C | 2.988695 | -0.610995 | 1.443111 |
| H | 0.957478 | -0.972752 | 2.119244 |
| C | 3.375229 | -0.964405 | -0.913156 |
| H | 1.680722 | -1.632430 | -2.068607 |
| C | 3.858135 | -0.599642 | 0.344279 |
| H | 3.351367 | -0.327660 | 2.428085 |
| H | 4.040561 | -0.967506 | -1.773069 |
| H | 4.898500 | -0.312901 | 0.470340 |
| H | -2.889643 | -1.377512 | -1.933366 |

Electronic Energy = -839.994211918 (Hartree/Particle)
Dipole Moment (Debye): 4.1141
index 0
Harmonic frequencies
Number of imaginary frequencies= 0

THERMOCHEMICAL DATA

Temperature 298.150 Kelvin. Pressure 1.00000 Atm.
Zero-point correction= 0.263916
(Hartree/Particle)
Thermal correction to Energy= 0.279548
Thermal correction to Enthalpy= 0.280492
Thermal correction to Gibbs Free Energy= 0.218680
Sum of electronic and zero-point Energies= -839.730296
Sum of electronic and thermal Energies= -839.714664
Sum of electronic and thermal Enthalpies= -839.713720
Sum of electronic and thermal Free Energies= -839.775531
PCM-SP (solvent=THF;6-311+g(2d,p)) = -840.217420194
Corrected Free Energy = -839.998739276

-------------------------------------------------------

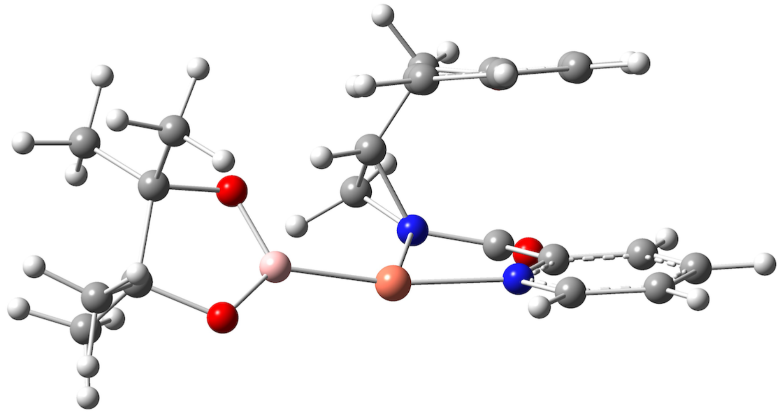

Complex-1 Substrate: 1c

CARTESIAN COORDINATES

| | | | |
|---|---|---|---|
| C | -0.672592 | -3.165588 | 1.478305 |
| N | -1.143900 | -2.297956 | 0.424495 |
| C | -2.469124 | -2.226866 | 0.029465 |

| | | | |
|---|---|---|---|
| O | -3.269567 | -3.138254 | 0.203813 |
| C | -0.501938 | -1.674776 | 1.589485 |
| C | -2.870600 | -1.001196 | -0.757603 |
| C | -4.237654 | -0.740706 | -0.888412 |
| C | -4.654967 | 0.366248 | -1.623295 |
| C | -3.688323 | 1.181386 | -2.212985 |
| C | -2.346179 | 0.854060 | -2.041343 |
| N | -1.932588 | -0.214787 | -1.332239 |
| Cu | 0.078993 | -0.345234 | -1.180681 |
| B | 1.997858 | -0.207229 | -0.800263 |
| O | 3.093561 | -0.042219 | -1.638881 |
| C | 4.317688 | -0.187355 | -0.869200 |
| C | 5.373945 | 0.756605 | -1.439837 |
| O | 2.438802 | -0.234464 | 0.538472 |
| C | 3.839771 | 0.152993 | 0.584550 |
| C | 3.874958 | 1.656292 | 0.896989 |
| C | 4.533661 | -0.628195 | 1.697645 |
| C | 4.775527 | -1.644573 | -1.028569 |
| H | -1.432200 | -3.649361 | 2.091544 |
| H | 0.478120 | -1.261896 | 1.366426 |
| H | -4.941067 | -1.414037 | -0.412120 |
| H | -5.712935 | 0.588340 | -1.732078 |
| H | -1.560012 | 1.463154 | -2.476261 |
| H | -3.958805 | 2.058558 | -2.792145 |
| H | 5.612044 | -0.426248 | 1.701054 |
| H | 4.128020 | -0.326443 | 2.669960 |
| H | 4.380211 | -1.704519 | 1.587702 |
| H | 3.335525 | 1.836776 | 1.833283 |
| H | 4.900913 | 2.024113 | 1.012651 |
| H | 3.384045 | 2.230029 | 0.104455 |
| H | 4.893566 | -1.861508 | -2.095105 |
| H | 5.733454 | -1.826362 | -0.527283 |
| H | 4.028677 | -2.336006 | -0.625412 |
| H | 6.284673 | 0.737078 | -0.827989 |
| H | 5.637170 | 0.442335 | -2.455471 |
| H | 5.007786 | 1.784802 | -1.492324 |
| C | -1.362998 | -0.908948 | 2.583265 |
| H | -0.716531 | -0.462692 | 3.351429 |
| H | -2.070561 | -1.583829 | 3.071975 |
| O | -2.202121 | 0.084734 | 1.996002 |
| C | -1.689944 | 1.286384 | 1.569541 |
| C | -2.653474 | 2.233413 | 1.193897 |
| C | -0.327697 | 1.584194 | 1.458116 |
| C | -2.254429 | 3.472932 | 0.700389 |
| H | -3.702935 | 1.970745 | 1.287391 |
| C | 0.057345 | 2.836218 | 0.962580 |
| H | 0.442715 | 0.861533 | 1.694704 |
| C | -0.892599 | 3.783515 | 0.581019 |
| H | -3.008632 | 4.200043 | 0.409169 |
| H | 1.117487 | 3.047703 | 0.857533 |
| H | -0.580822 | 4.749153 | 0.192674 |
| H | 0.187766 | -3.778956 | 1.220263 |

Electronic Energy = -1448.67011541 (Hartree/Particle)
Dipole Moment (Debye): 3.2139
index 0
Harmonic frequencies
Number of imaginary frequencies= 0

THERMOCHEMICAL DATA

```
 Temperature   298.150 Kelvin.  Pressure   1.00000 Atm.
 Zero-point correction=                           0.448049
(Hartree/Particle)
 Thermal correction to Energy=                    0.476249
 Thermal correction to Enthalpy=                  0.477193
 Thermal correction to Gibbs Free Energy=         0.387698
 Sum of electronic and zero-point Energies=          -1448.222067
 Sum of electronic and thermal Energies=             -1448.193867
 Sum of electronic and thermal Enthalpies=           -1448.192923
 Sum of electronic and thermal Free Energies=        -1448.282417
 PCM-SP (solvent=THF;6-311+g(2d,p)) =  -1449.030321190
 Corrected Free Energy =   -1448.64262276
```

------------------------------------------------------

PATH: ANTI_MARKOVNIKOV

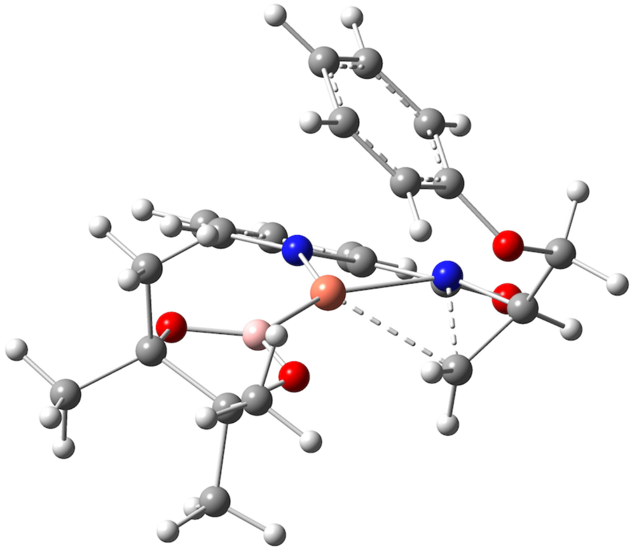

TS1    Substrate: 1c
       Path: anti-Markovnikov

CARTESIAN COORDINATES

| | | | |
|---|---|---|---|
| N | -2.291319 | -1.371703 | 0.591008 |
| C | -3.420292 | -0.994961 | -0.038151 |
| C | -4.620853 | -1.699748 | 0.089542 |
| C | -4.648641 | -2.814577 | 0.926908 |
| C | -3.477999 | -3.196635 | 1.590303 |
| C | -2.314632 | -2.454045 | 1.379031 |
| C | -3.334711 | 0.218586 | -0.919769 |
| O | -4.291466 | 0.583987 | -1.603280 |
| Cu | -0.573845 | -0.386797 | -0.214258 |
| B | 1.332467 | -0.899835 | -0.298911 |
| O | 2.013409 | -1.871554 | 0.428860 |
| C | 3.444536 | -1.679443 | 0.251446 |
| C | 4.128218 | -3.044964 | 0.244074 |
| N | -2.108786 | 0.881323 | -0.839832 |
| C | -0.950318 | 0.848894 | -2.226872 |
| C | -1.880145 | 1.925827 | -1.828150 |
| O | 2.221136 | -0.227586 | -1.141621 |

| | | | |
|---|---|---|---|
| C | 3.512239 | -0.894752 | -1.107781 |
| C | 3.571957 | -1.811323 | -2.337729 |
| C | 4.610527 | 0.163400 | -1.189244 |
| C | 3.930595 | -0.855233 | 1.452162 |
| H | -2.741282 | 2.100018 | -2.477413 |
| H | -1.292925 | 0.106626 | -2.938957 |
| H | 0.118269 | 0.957076 | -2.074576 |
| H | -5.490972 | -1.365310 | -0.465090 |
| H | -5.566674 | -3.380995 | 1.057614 |
| H | -1.367452 | -2.719468 | 1.840383 |
| H | -3.460451 | -4.056049 | 2.253601 |
| H | 5.602310 | -0.293138 | -1.080508 |
| H | 4.568870 | 0.663041 | -2.163402 |
| H | 4.488593 | 0.925424 | -0.415606 |
| H | 3.418420 | -1.205663 | -3.237277 |
| H | 4.540852 | -2.317378 | -2.421220 |
| H | 2.781388 | -2.567587 | -2.300239 |
| H | 3.676417 | -1.390926 | 2.372847 |
| H | 5.015868 | -0.701245 | 1.426935 |
| H | 3.432096 | 0.117568 | 1.483327 |
| H | 5.200081 | -2.942027 | 0.032770 |
| H | 4.017613 | -3.516365 | 1.226701 |
| H | 3.686694 | -3.712460 | -0.499954 |
| C | -1.295320 | 3.236941 | -1.311053 |
| H | -1.219290 | 3.929981 | -2.155482 |
| H | -1.964965 | 3.682646 | -0.564707 |
| O | 0.035029 | 3.122091 | -0.822346 |
| C | 0.307225 | 2.813165 | 0.489757 |
| C | 1.670169 | 2.802602 | 0.816973 |
| C | -0.651002 | 2.517411 | 1.465828 |
| C | 2.070053 | 2.503358 | 2.116379 |
| H | 2.392205 | 3.011631 | 0.034066 |
| C | -0.232871 | 2.217298 | 2.768154 |
| H | -1.703489 | 2.472201 | 1.218121 |
| C | 1.121186 | 2.207811 | 3.103691 |
| H | 3.129637 | 2.495391 | 2.359385 |
| H | -0.982751 | 1.976708 | 3.517718 |
| H | 1.436442 | 1.966051 | 4.114850 |

Electronic Energy = -1448.63550863 (Hartree/Particle)
Dipole Moment (Debye): 2.7296
index 1
Number of imaginary frequencies= 1
Negatives Eigenvalues: -354.111

THERMOCHEMICAL DATA

Temperature 298.150 Kelvin. Pressure 1.00000 Atm.
Zero-point correction= 0.445180
(Hartree/Particle)
Thermal correction to Energy= 0.473169
Thermal correction to Enthalpy= 0.474114
Thermal correction to Gibbs Free Energy= 0.383444
Sum of electronic and zero-point Energies= -1448.190329
Sum of electronic and thermal Energies= -1448.162339
Sum of electronic and thermal Enthalpies= -1448.161395
Sum of electronic and thermal Free Energies= -1448.252065
PCM-SP (solvent=THF;6-311+g(2d,p)) = -1448.996090760
Corrected Free Energy = -1448.612647130

------------------------------------------------------

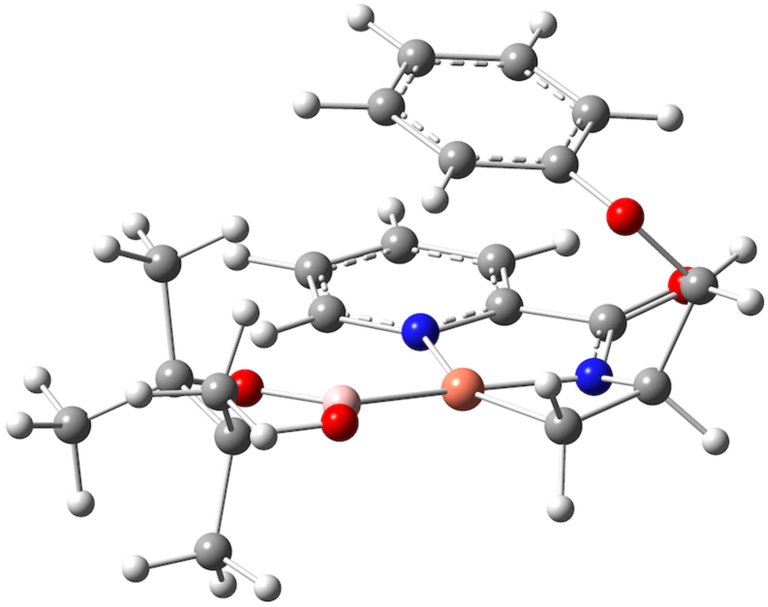

I1 Substrate: 1c
Path: anti-Markovnikov

CARTESIAN COORDINATES

| | | | |
|---|---|---|---|
| C | 1.553884 | -2.534828 | -1.172968 |
| C | 0.009315 | -2.423661 | -1.166077 |
| H | 1.942091 | -3.164843 | -1.989855 |
| H | -0.476346 | -2.669574 | -2.111688 |
| H | -0.515222 | -2.841373 | -0.309064 |
| N | 1.820199 | -1.117939 | -1.396185 |
| C | 2.885483 | -0.346167 | -1.179434 |
| C | 2.425700 | 1.108616 | -1.038206 |
| C | 3.333243 | 2.160164 | -0.951939 |
| C | 2.849432 | 3.453459 | -0.748682 |
| H | 4.391312 | 1.932614 | -1.030752 |
| C | 0.621358 | 2.553164 | -0.712092 |
| C | 1.472479 | 3.654996 | -0.621783 |
| H | 3.536488 | 4.292909 | -0.680066 |
| H | -0.456204 | 2.630446 | -0.607374 |
| H | 1.058692 | 4.644113 | -0.451594 |
| O | 4.080480 | -0.650435 | -1.050325 |
| N | 1.091870 | 1.316676 | -0.925094 |
| Cu | 0.103567 | -0.459240 | -1.039093 |
| B | -1.790402 | -0.193976 | -0.569722 |
| O | -2.730077 | -1.160691 | -0.300658 |
| O | -2.294007 | 1.082088 | -0.385624 |
| C | -4.021524 | -0.502314 | -0.118761 |
| C | -3.595257 | 0.957893 | 0.274234 |
| C | 2.137712 | -3.095697 | 0.136568 |
| H | 2.018348 | -4.183030 | 0.148252 |
| H | 3.204507 | -2.856105 | 0.195210 |
| O | 1.430815 | -2.658768 | 1.309841 |
| C | 1.575510 | -1.368479 | 1.762204 |
| C | 0.402041 | -0.708271 | 2.155124 |
| C | 2.814301 | -0.729339 | 1.889635 |
| C | 0.466386 | 0.596486 | 2.647368 |
| H | -0.545266 | -1.226760 | 2.043493 |

| | | | |
|---|---|---|---|
| C | 2.863859 | 0.581376 | 2.371156 |
| H | 3.725546 | -1.224749 | 1.575551 |
| C | 1.696940 | 1.252772 | 2.750359 |
| H | -0.450290 | 1.106664 | 2.931011 |
| H | 3.827445 | 1.079326 | 2.443138 |
| H | 1.746518 | 2.273733 | 3.119082 |
| C | -4.805560 | -1.256292 | 0.951013 |
| H | -5.745220 | -0.738155 | 1.177851 |
| H | -5.047484 | -2.261160 | 0.589715 |
| H | -4.230651 | -1.360768 | 1.874118 |
| C | -4.749014 | -0.580476 | -1.466669 |
| H | -4.829902 | -1.630683 | -1.764314 |
| H | -5.757983 | -0.158038 | -1.403340 |
| H | -4.194081 | -0.049438 | -2.246731 |
| C | -3.341633 | 1.131511 | 1.776703 |
| H | -2.850282 | 2.095294 | 1.945370 |
| H | -4.277160 | 1.113926 | 2.346325 |
| H | -2.684681 | 0.344679 | 2.159585 |
| C | -4.511559 | 2.062710 | -0.240908 |
| H | -5.523819 | 1.943140 | 0.163753 |
| H | -4.130492 | 3.038667 | 0.078529 |
| H | -4.568039 | 2.062566 | -1.331882 |

Electronic Energy = -1448.67962787 (Hartree/Particle)
Dipole Moment (Debye): 6.3387
index 0
Harmonic frequencies
Number of imaginary frequencies= 0

THERMOCHEMICAL DATA

Temperature 298.150 Kelvin. Pressure 1.00000 Atm.
Zero-point correction= 0.447803 (Hartree/Particle)
Thermal correction to Energy= 0.475187
Thermal correction to Enthalpy= 0.476132
Thermal correction to Gibbs Free Energy= 0.390052
Sum of electronic and zero-point Energies= -1448.231825
Sum of electronic and thermal Energies= -1448.204440
Sum of electronic and thermal Enthalpies= -1448.203496
Sum of electronic and thermal Free Energies= -1448.289576
PCM-SP (solvent=THF;6-311+g(2d,p)) = -1449.039370430
Corrected Free Energy = -1448.649318560

-------------------------------------------------------

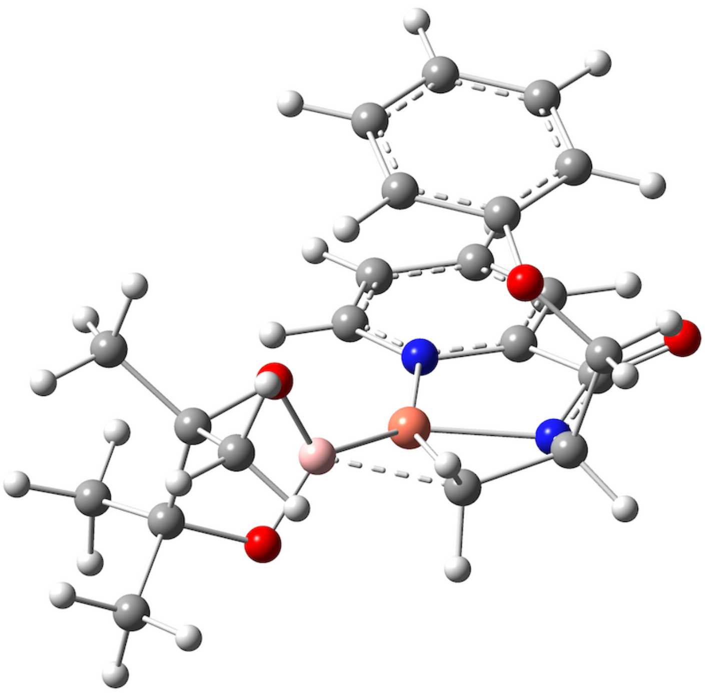

TS2 Substrate: 1c
Path: anti-Markovnikov

CARTESIAN COORDINATES

| | | | |
|---|---|---|---|
| C | 2.762474 | 0.203021 | 2.914445 |
| C | 1.364116 | 0.254095 | 2.913464 |
| C | 0.622846 | -0.659388 | 2.162962 |
| C | 1.281865 | -1.638297 | 1.404807 |
| C | 2.678892 | -1.718394 | 1.423078 |
| C | 3.408043 | -0.786970 | 2.167868 |
| O | 0.468845 | -2.511869 | 0.716488 |
| C | 0.837770 | -3.004873 | -0.578263 |
| C | 0.453823 | -2.041783 | -1.718578 |
| Cu | -0.023885 | 0.279332 | -0.962088 |
| B | -1.923049 | -0.003698 | -0.479176 |
| O | -2.930854 | 0.539543 | -1.250978 |
| C | -4.016420 | 0.905911 | -0.344373 |
| C | -5.342697 | 0.717489 | -1.073390 |
| C | -0.983825 | -1.419310 | -1.520474 |
| N | 1.283116 | -0.851868 | -1.831821 |
| C | 2.554956 | -0.650012 | -1.516776 |
| O | 3.539030 | -1.411866 | -1.511544 |
| C | 2.723823 | 0.796778 | -1.007630 |
| C | 3.989987 | 1.362848 | -0.867131 |
| C | 4.113496 | 2.640591 | -0.324022 |
| C | 2.962263 | 3.316377 | 0.088845 |
| C | 1.729823 | 2.685299 | -0.065759 |
| N | 1.607188 | 1.462488 | -0.605382 |
| O | -2.327149 | -0.265396 | 0.811448 |
| C | -3.775658 | -0.071425 | 0.863268 |
| C | -4.408556 | -1.453188 | 0.661278 |
| C | -4.141013 | 0.488751 | 2.233333 |
| C | -3.810708 | 2.381383 | 0.020685 |
| H | 0.450650 | -2.647390 | -2.642119 |
| H | -1.444425 | -1.223976 | -2.491923 |
| H | -1.625993 | -2.080136 | -0.931644 |

| | | | |
|---|---|---|---|
| H | 4.846072 | 0.772291 | -1.175911 |
| H | 5.092002 | 3.100684 | -0.213011 |
| H | 0.806110 | 3.157531 | 0.254090 |
| H | 3.011707 | 4.307024 | 0.530068 |
| H | 0.267845 | -3.933384 | -0.685454 |
| H | 1.904621 | -3.236182 | -0.645005 |
| H | -0.459858 | -0.619245 | 2.126356 |
| H | 3.203342 | -2.456619 | 0.829675 |
| H | 0.844078 | 1.016349 | 3.489522 |
| H | 4.493757 | -0.839567 | 2.153626 |
| H | 3.340443 | 0.923017 | 3.487891 |
| H | -5.208791 | 0.734679 | 2.275695 |
| H | -3.930744 | -0.258458 | 3.005525 |
| H | -3.564265 | 1.386774 | 2.466776 |
| H | -4.023251 | -2.134442 | 1.426315 |
| H | -5.499671 | -1.410592 | 0.748839 |
| H | -4.156166 | -1.866304 | -0.320903 |
| H | -3.785988 | 2.973436 | -0.899643 |
| H | -4.623118 | 2.754442 | 0.653634 |
| H | -2.861142 | 2.528309 | 0.545576 |
| H | -6.183236 | 0.894694 | -0.391753 |
| H | -5.414834 | 1.432494 | -1.899442 |
| H | -5.434155 | -0.288610 | -1.489069 |

Electronic Energy = -1448.67004685 (Hartree/Particle)
Dipole Moment (Debye): 6.6134
index 1
Number of imaginary frequencies= 1
Negatives Eigenvalues: -152.728

THERMOCHEMICAL DATA

Temperature 298.150 Kelvin. Pressure 1.00000 Atm.
Zero-point correction= 0.446976
(Hartree/Particle)
Thermal correction to Energy= 0.473918
Thermal correction to Enthalpy= 0.474862
Thermal correction to Gibbs Free Energy= 0.389903
Sum of electronic and zero-point Energies= -1448.223071
Sum of electronic and thermal Energies= -1448.196129
Sum of electronic and thermal Enthalpies= -1448.195185
Sum of electronic and thermal Free Energies= -1448.280147
PCM-SP (solvent=THF;6-311+g(2d,p)) = -1449.033067670
Corrected Free Energy = -1448.643167820

-------------------------------------------------------

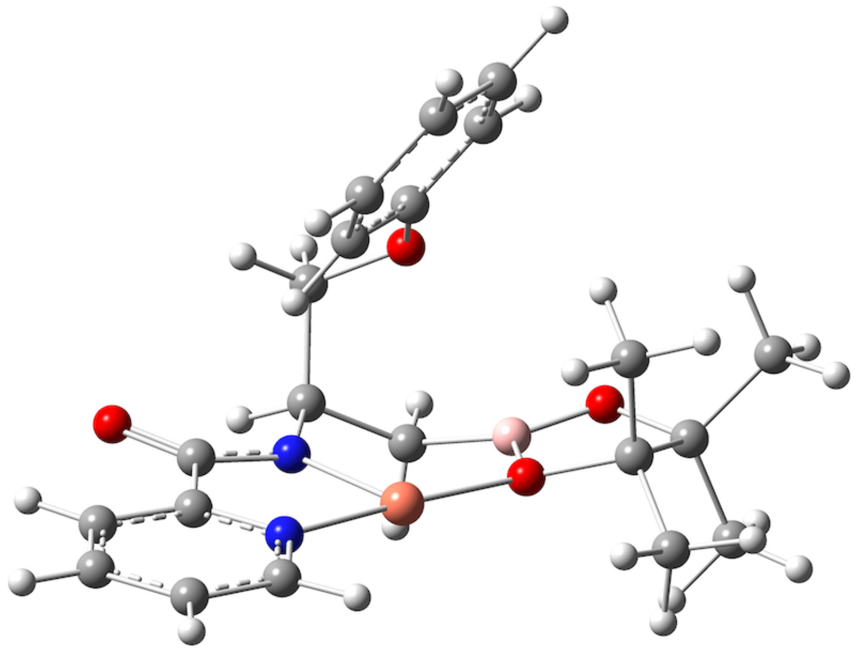

P1 Substrate: 1c
Path: anti-Markovnikov

CARTESIAN COORDINATES

| | | | |
|---|---|---|---|
| C | -0.048315 | 3.941478 | -1.977978 |
| C | -1.275774 | 4.092846 | -1.321442 |
| C | -1.535260 | 3.397449 | -0.139798 |
| C | -0.570479 | 2.528255 | 0.390055 |
| C | 0.662516 | 2.375247 | -0.257551 |
| C | 0.915054 | 3.086564 | -1.434558 |
| O | -0.943003 | 1.821903 | 1.501215 |
| C | 0.022460 | 1.487811 | 2.523713 |
| C | 0.322704 | -0.019112 | 2.564250 |
| Cu | 0.622526 | -1.103700 | -0.374093 |
| B | -1.837331 | -0.854811 | 1.217260 |
| O | -1.335445 | -1.202334 | -0.054352 |
| C | -2.426712 | -1.107145 | -1.045006 |
| C | -2.243319 | -2.243596 | -2.042816 |
| C | -0.990346 | -0.838031 | 2.543007 |
| N | 1.176959 | -0.440140 | 1.471535 |
| C | 2.481753 | -0.204201 | 1.556445 |
| O | 3.135468 | 0.325407 | 2.483359 |
| C | 3.245182 | -0.630804 | 0.295081 |
| C | 4.637935 | -0.531845 | 0.264226 |
| C | 5.327654 | -0.902012 | -0.889204 |
| C | 4.604579 | -1.357061 | -1.995236 |
| C | 3.216422 | -1.423544 | -1.898727 |
| N | 2.548489 | -1.071690 | -0.785220 |
| O | -3.197341 | -0.728103 | 1.167590 |
| C | -3.689053 | -1.213739 | -0.113796 |
| C | -4.153407 | -2.656864 | 0.119098 |
| C | -4.864634 | -0.340729 | -0.543835 |
| C | -2.273144 | 0.245195 | -1.737629 |
| H | 0.834678 | -0.184640 | 3.525315 |
| H | -0.722412 | -1.885854 | 2.751504 |
| H | -1.643854 | -0.517584 | 3.364903 |
| H | 5.135295 | -0.161025 | 1.153812 |

```
 H               6.411774   -0.834764   -0.928559
 H               2.606261   -1.764431   -2.729041
 H               5.098091   -1.652746   -2.915926
 H              -0.445647    1.795508    3.466458
 H               0.946269    2.057290    2.387077
 H              -2.479400    3.505866    0.386670
 H               1.397055    1.687458    0.139889
 H              -2.031745    4.762008   -1.725436
 H               1.873654    2.958351   -1.931773
 H               0.155188    4.487105   -2.895435
 H              -5.217393   -0.631612   -1.540290
 H              -5.690388   -0.467346    0.163582
 H              -4.593193    0.716975   -0.560936
 H              -4.914014   -2.659474    0.905880
 H              -4.588373   -3.090555   -0.787788
 H              -3.324729   -3.292029    0.449040
 H              -1.265199    0.317021   -2.161415
 H              -3.001359    0.357275   -2.547624
 H              -2.392356    1.072699   -1.037673
 H              -3.065923   -2.253556   -2.766597
 H              -1.305624   -2.092739   -2.589840
 H              -2.193716   -3.216986   -1.549916

 Electronic Energy =  -1448.75568260 (Hartree/Particle)
 Dipole Moment (Debye):      7.6320
 index           0
 Harmonic frequencies
 Number of imaginary frequencies=            0

 THERMOCHEMICAL DATA

 Temperature   298.150 Kelvin.  Pressure   1.00000 Atm.
 Zero-point correction=                           0.449187
(Hartree/Particle)
 Thermal correction to Energy=                    0.476571
 Thermal correction to Enthalpy=                  0.477515
 Thermal correction to Gibbs Free Energy=         0.390519
 Sum of electronic and zero-point Energies=          -1448.306495
 Sum of electronic and thermal Energies=             -1448.279111
 Sum of electronic and thermal Enthalpies=           -1448.278167
 Sum of electronic and thermal Free Energies=        -1448.365164
 PCM-SP (solvent=THF;6-311+g(2d,p)) =  -1449.11747741
 Corrected Free Energy =  -1448.72695881
```

------------------------------------------------------

PATH: MARKOVNIKOV

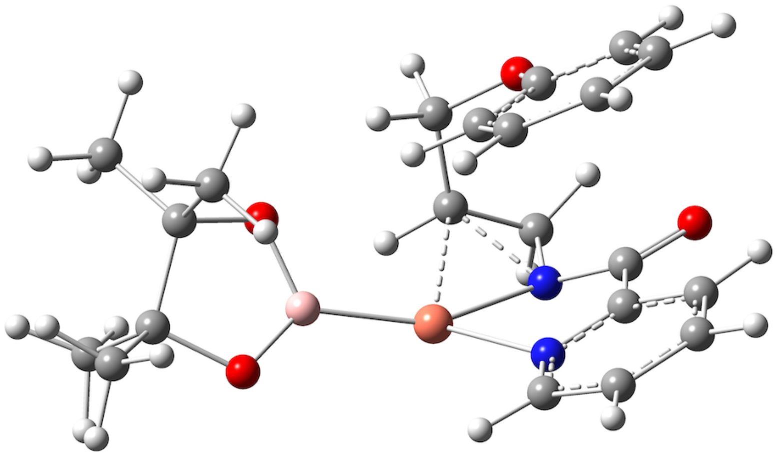

TS1 Substrate: 1c
Path: Markovnikov

CARTESIAN COORDINATES

| | | | |
|---|---|---|---|
| C | -1.539071 | -3.219791 | -0.940023 |
| N | -1.497699 | -1.820718 | -1.343657 |
| C | -2.643205 | -1.070986 | -1.052892 |
| O | -3.728369 | -1.557097 | -0.748347 |
| C | -0.358641 | -2.671925 | -0.247513 |
| C | -2.482551 | 0.408716 | -1.251461 |
| C | -3.551502 | 1.273344 | -1.006355 |
| C | -3.361162 | 2.638075 | -1.213195 |
| C | -2.112495 | 3.092770 | -1.647103 |
| C | -1.091884 | 2.161698 | -1.842005 |
| N | -1.277590 | 0.848614 | -1.655983 |
| Cu | 0.219586 | -0.594883 | -1.334917 |
| B | 2.044159 | -0.193478 | -0.688605 |
| O | 3.056997 | 0.571332 | -1.257198 |
| C | 4.232022 | 0.530664 | -0.399565 |
| C | 4.936621 | 1.883685 | -0.466210 |
| O | 2.425076 | -0.575314 | 0.611436 |
| C | 3.612040 | 0.174370 | 0.995135 |
| C | 3.123362 | 1.404304 | 1.774884 |
| C | 4.484804 | -0.700501 | 1.890914 |
| C | 5.151497 | -0.568230 | -0.951006 |
| H | -2.401069 | -3.475072 | -0.323064 |
| H | 0.618844 | -2.874188 | -0.676276 |
| H | -4.488927 | 0.862177 | -0.650254 |
| H | -4.170449 | 3.339208 | -1.028985 |
| H | -0.092138 | 2.455976 | -2.149126 |
| H | -1.925890 | 4.147756 | -1.823884 |
| H | 5.431023 | -0.196129 | 2.123239 |
| H | 3.964521 | -0.899333 | 2.834474 |
| H | 4.707370 | -1.660972 | 1.419706 |
| H | 2.519943 | 1.069481 | 2.625353 |
| H | 3.958706 | 2.002199 | 2.156759 |
| H | 2.494888 | 2.042049 | 1.144456 |
| H | 5.375429 | -0.346917 | -1.999551 |
| H | 6.095795 | -0.622856 | -0.396842 |
| H | 4.661550 | -1.546332 | -0.910108 |
| H | 5.778350 | 1.918542 | 0.236852 |
| H | 5.327772 | 2.045694 | -1.476361 |

| | | | |
|---|---|---|---|
| H | 4.253136 | 2.704572 | -0.235553 |
| C | -0.328779 | -2.388779 | 1.232940 |
| H | 0.512281 | -1.737232 | 1.476755 |
| H | -0.153527 | -3.360476 | 1.720537 |
| O | -1.566105 | -1.927303 | 1.750422 |
| C | -1.787810 | -0.580650 | 1.934959 |
| C | -3.089133 | -0.252092 | 2.339597 |
| C | -0.832024 | 0.424850 | 1.747645 |
| C | -3.430656 | 1.079487 | 2.562689 |
| H | -3.814496 | -1.051577 | 2.451452 |
| C | -1.193824 | 1.758589 | 1.972720 |
| H | 0.172714 | 0.199970 | 1.411299 |
| C | -2.484160 | 2.096708 | 2.380634 |
| H | -4.443381 | 1.324569 | 2.873234 |
| H | -0.448283 | 2.534027 | 1.814762 |
| H | -2.753684 | 3.135854 | 2.548710 |
| H | -1.363748 | -3.926068 | -1.753743 |

Electronic Energy = -1448.64018835 (Hartree/Particle)
Dipole Moment (Debye): 2.2655
index 1
Number of imaginary frequencies= 1
Negatives Eigenvalues: -319.822

THERMOCHEMICAL DATA

Temperature 298.150 Kelvin. Pressure 1.00000 Atm.
Zero-point correction= 0.445787
(Hartree/Particle)
Thermal correction to Energy= 0.473495
Thermal correction to Enthalpy= 0.474439
Thermal correction to Gibbs Free Energy= 0.386458
Sum of electronic and zero-point Energies= -1448.194401
Sum of electronic and thermal Energies= -1448.166693
Sum of electronic and thermal Enthalpies= -1448.165749
Sum of electronic and thermal Free Energies= -1448.253731
PCM-SP (solvent=THF;6-311+g(2d,p)) = -1448.999723900
Corrected Free Energy = -1448.613266550

---

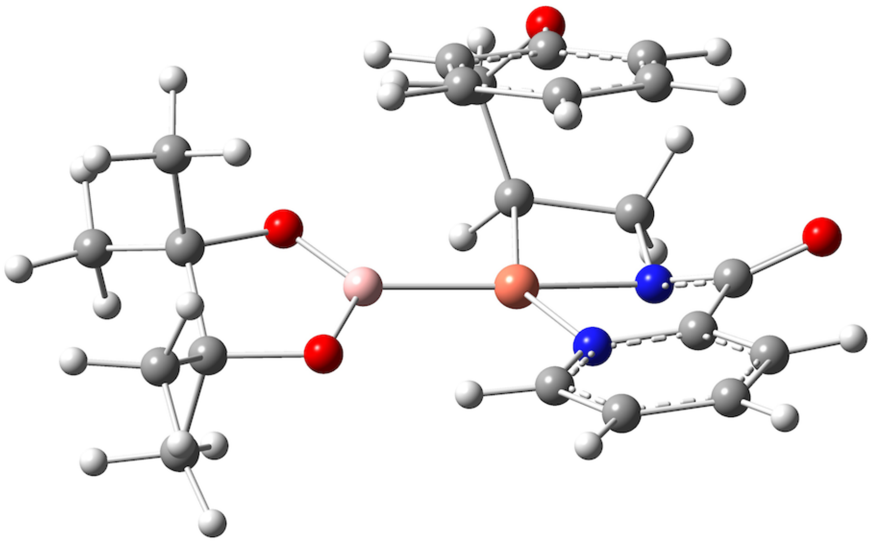

I1 Substrate: 1c
Path: Markovnikov

CARTESIAN COORDINATES

| | | | |
|---|---|---|---|
| C | -1.428552 | 0.783966 | 2.961970 |
| C | -2.522516 | 0.070286 | 2.460296 |
| C | -2.336254 | -1.158196 | 1.824283 |
| C | -1.045633 | -1.681231 | 1.686717 |
| C | 0.054373 | -0.984665 | 2.206116 |
| C | -0.143821 | 0.247562 | 2.834591 |
| O | -0.929703 | -2.906657 | 1.072728 |
| C | 0.074298 | -3.079084 | 0.057429 |
| C | -0.237905 | -2.350303 | -1.237568 |
| Cu | -0.316172 | -0.373191 | -1.043184 |
| B | 1.567790 | -0.056663 | -0.549372 |
| O | 2.550517 | -0.974385 | -0.250103 |
| C | 3.735295 | -0.248111 | 0.205227 |
| C | 4.974873 | -0.983010 | -0.295277 |
| C | -1.705885 | -2.402463 | -1.719998 |
| N | -1.975892 | -0.967064 | -1.700351 |
| C | -3.050389 | -0.340789 | -1.203542 |
| C | -2.671726 | 1.090734 | -0.813390 |
| C | -3.630083 | 2.054738 | -0.514170 |
| C | -3.206076 | 3.319273 | -0.101847 |
| C | -1.837552 | 3.575858 | 0.017611 |
| C | -0.934395 | 2.555484 | -0.282527 |
| N | -1.348703 | 1.351188 | -0.697627 |
| O | -4.198363 | -0.763311 | -1.013179 |
| O | 2.050144 | 1.239434 | -0.513102 |
| C | 3.508851 | 1.177622 | -0.416674 |
| C | 4.048082 | 1.311782 | -1.845954 |
| C | 3.988762 | 2.343470 | 0.442310 |
| C | 3.700053 | -0.252360 | 1.738027 |
| H | -2.357260 | -2.958892 | -1.035282 |
| H | 0.514108 | -2.580888 | -1.996299 |
| H | -4.677430 | 1.783524 | -0.597787 |
| H | -3.932613 | 4.093188 | 0.132008 |
| H | 0.140648 | 2.675944 | -0.196265 |
| H | -1.469832 | 4.543855 | 0.343695 |
| H | 5.882989 | -0.419477 | -0.049385 |
| H | 5.040938 | -1.964134 | 0.186605 |
| H | 4.941137 | -1.140068 | -1.375788 |
| H | 3.654899 | -1.287681 | 2.091323 |
| H | 4.595587 | 0.216878 | 2.159573 |
| H | 2.819750 | 0.275648 | 2.115282 |
| H | 3.678548 | 2.247169 | -2.278117 |
| H | 5.143424 | 1.330253 | -1.861730 |
| H | 3.701853 | 0.487831 | -2.477920 |
| H | 5.070531 | 2.279381 | 0.610530 |
| H | 3.779495 | 3.288761 | -0.069580 |
| H | 3.484675 | 2.364933 | 1.411449 |
| H | 1.070257 | -2.811523 | 0.421846 |
| H | 0.057554 | -4.161394 | -0.130454 |
| H | -3.172334 | -1.699819 | 1.394246 |
| H | 1.051241 | -1.398076 | 2.108580 |
| H | -3.526622 | 0.477785 | 2.541706 |
| H | 0.713230 | 0.790321 | 3.226593 |
| H | -1.575649 | 1.746117 | 3.445054 |
| H | -1.810454 | -2.826653 | -2.727383 |

Electronic Energy = -1448.68016741 (Hartree/Particle)

Dipole Moment (Debye): 7.7856
index 0
Harmonic frequencies
Number of imaginary frequencies= 0

THERMOCHEMICAL DATA

Temperature 298.150 Kelvin. Pressure 1.00000 Atm.
Zero-point correction= 0.448298
(Hartree/Particle)
Thermal correction to Energy= 0.475662
Thermal correction to Enthalpy= 0.476606
Thermal correction to Gibbs Free Energy= 0.390666
Sum of electronic and zero-point Energies= -1448.231870
Sum of electronic and thermal Energies= -1448.204505
Sum of electronic and thermal Enthalpies= -1448.203561
Sum of electronic and thermal Free Energies= -1448.289502
PCM-SP (solvent=THF;6-311+g(2d,p)) = -1449.04101926
Corrected Free Energy = -1448.650353850

---

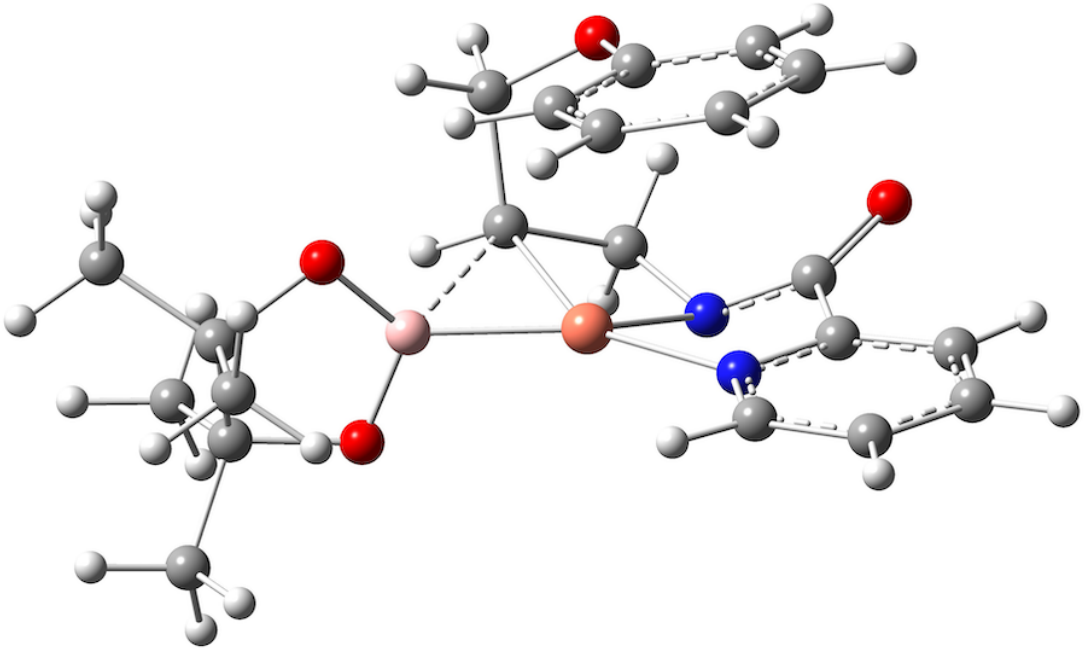

TS2 Substrate: 1c
Path: Markovnikov

CARTESIAN COORDINATES

| | | | |
|---|---|---|---|
| C | -2.608680 | 1.650478 | 2.384837 |
| C | -3.340835 | 0.479817 | 2.145806 |
| C | -2.692444 | -0.740353 | 1.969959 |
| C | -1.293223 | -0.799684 | 2.031609 |
| C | -0.547674 | 0.364487 | 2.269157 |
| C | -1.216409 | 1.582334 | 2.444999 |
| O | -0.748238 | -2.049051 | 1.867634 |
| C | 0.600296 | -2.189937 | 1.424443 |
| C | 0.795546 | -1.920119 | -0.075970 |
| Cu | -0.090813 | -0.177955 | -0.674358 |
| B | 1.836120 | -0.155208 | -0.271237 |

| | | | |
|---|---|---|---|
| O | 2.480294 | 0.145262 | 0.910408 |
| C | 3.723072 | 0.832320 | 0.560205 |
| C | 4.784582 | 0.469676 | 1.593118 |
| C | -0.279437 | -2.664462 | -0.963874 |
| N | -1.142497 | -1.613390 | -1.476431 |
| C | -2.437747 | -1.456807 | -1.218338 |
| C | -2.789568 | 0.042555 | -1.301000 |
| C | -4.097329 | 0.470648 | -1.525369 |
| C | -4.377127 | 1.836461 | -1.530372 |
| C | -3.343972 | 2.742735 | -1.276370 |
| C | -2.067171 | 2.241976 | -1.029920 |
| N | -1.790428 | 0.930019 | -1.055350 |
| O | -3.326132 | -2.264353 | -0.893941 |
| O | 2.639468 | 0.017869 | -1.375105 |
| C | 3.994843 | 0.295515 | -0.892990 |
| C | 4.745399 | -1.040909 | -0.914418 |
| C | 4.653519 | 1.293702 | -1.838100 |
| C | 3.420797 | 2.335051 | 0.606063 |
| H | -0.813081 | -3.407453 | -0.354496 |
| H | 1.801424 | -2.267939 | -0.339942 |
| H | -4.862166 | -0.283695 | -1.677350 |
| H | -5.387065 | 2.191859 | -1.718705 |
| H | -1.234857 | 2.899121 | -0.797390 |
| H | -3.519747 | 3.813845 | -1.255112 |
| H | 5.754996 | 0.894716 | 1.310590 |
| H | 4.504262 | 0.877290 | 2.569916 |
| H | 4.893147 | -0.612377 | 1.697183 |
| H | 3.043905 | 2.590207 | 1.601602 |
| H | 4.319326 | 2.930198 | 0.411362 |
| H | 2.654213 | 2.605794 | -0.127059 |
| H | 4.713155 | -1.449816 | -1.929033 |
| H | 5.793617 | -0.915436 | -0.622279 |
| H | 4.283890 | -1.768805 | -0.238769 |
| H | 5.637159 | 1.592489 | -1.456365 |
| H | 4.792667 | 0.833486 | -2.821613 |
| H | 4.040382 | 2.188271 | -1.968638 |
| H | 1.288870 | -1.602456 | 2.038225 |
| H | 0.816471 | -3.250586 | 1.597924 |
| H | -3.240057 | -1.646104 | 1.732211 |
| H | 0.534094 | 0.338444 | 2.306448 |
| H | -4.424698 | 0.518702 | 2.075704 |
| H | -0.631747 | 2.481865 | 2.624151 |
| H | -3.119351 | 2.601004 | 2.513161 |
| H | 0.222888 | -3.183021 | -1.789506 |

Electronic Energy = -1448.66478263 (Hartree/Particle)
Dipole Moment (Debye): 8.3356
index 1
Number of imaginary frequencies= 1
Negatives Eigenvalues: -140.390

THERMOCHEMICAL DATA

Temperature 298.150 Kelvin. Pressure 1.00000 Atm.
Zero-point correction= 0.447377
(Hartree/Particle)
Thermal correction to Energy= 0.474279
Thermal correction to Enthalpy= 0.475223
Thermal correction to Gibbs Free Energy= 0.390899
Sum of electronic and zero-point Energies= -1448.217405

Sum of electronic and thermal Energies= -1448.190504
Sum of electronic and thermal Enthalpies= -1448.189560
Sum of electronic and thermal Free Energies= -1448.273884
PCM-SP (solvent=THF;6-311+g(2d,p)) = -1449.030306880
Corrected Free Energy = -1448.639408250

-------------------------------------------------------

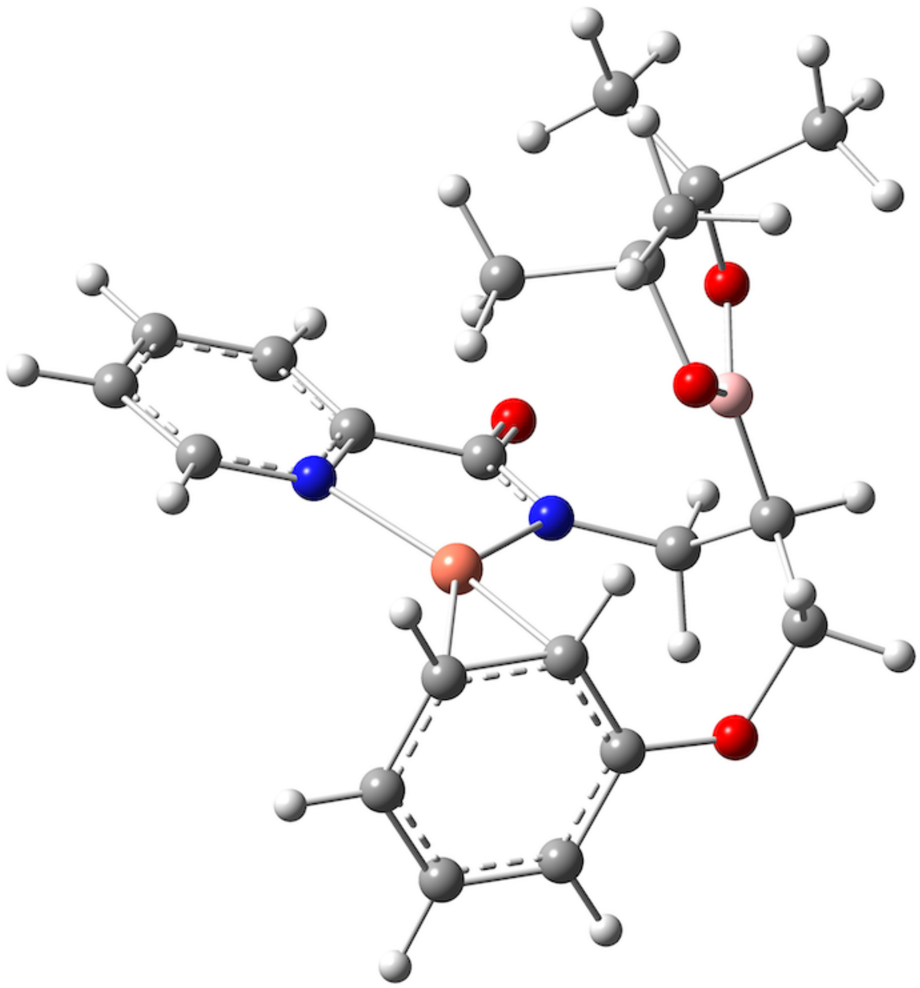

P1 Substrate: 1c
Path: Markovnikov

CARTESIAN COORDINATES

| | | | |
|---|---|---|---|
| C | -3.720677 | -1.064927 | 1.660248 |
| C | -4.284119 | -1.700216 | 0.560295 |
| C | -3.488324 | -2.399862 | -0.365100 |
| C | -2.107940 | -2.476668 | -0.190124 |
| C | -1.501859 | -1.863195 | 0.947081 |
| C | -2.319055 | -1.136806 | 1.858339 |
| O | -1.405655 | -3.124084 | -1.149974 |
| C | 0.032711 | -3.210205 | -1.095218 |
| C | 0.819114 | -1.982362 | -1.658552 |
| Cu | -1.308521 | 0.082452 | 0.332527 |
| B | 1.787618 | -1.296943 | -0.618496 |
| O | 1.757550 | -1.514107 | 0.745628 |
| C | 2.631471 | -0.523752 | 1.365677 |
| C | 3.309506 | -1.161446 | 2.574277 |
| C | -0.018159 | -0.857133 | -2.340479 |
| N | -0.391429 | 0.211415 | -1.414576 |

```
 C              -0.218931    1.470287   -1.838132
 C              -0.650742    2.522177   -0.813862
 C              -0.426116    3.877891   -1.066747
 C              -0.830351    4.821917   -0.123856
 C              -1.458318    4.389248    1.048440
 C              -1.652634    3.021304    1.228367
 N              -1.256477    2.112115    0.323621
 O               0.232672    1.883079   -2.924705
 O               2.785216   -0.434841   -0.991642
 C               3.596475   -0.141586    0.178001
 C               4.836422   -1.040188    0.091122
 C               4.002208    1.329282    0.128757
 C               1.734994    0.638155    1.810102
 H              -0.921983   -1.291901   -2.787570
 H               1.458117   -2.385122   -2.457609
 H               0.056522    4.146310   -2.000064
 H              -0.662531    5.881443   -0.299261
 H              -2.139129    2.626925    2.116223
 H              -1.794176    5.090724    1.806028
 H               4.038755   -0.471161    3.014539
 H               2.559013   -1.396509    3.336826
 H               3.821755   -2.088822    2.307420
 H               0.956136    0.252570    2.477304
 H               2.305422    1.403029    2.347768
 H               1.240333    1.102460    0.953912
 H               5.349439   -0.842064   -0.855210
 H               5.533980   -0.845477    0.913199
 H               4.559288   -2.099459    0.110048
 H               4.541329    1.613663    1.040578
 H               4.662181    1.497647   -0.728241
 H               3.132873    1.980056    0.011561
 H               0.342457   -3.455145   -0.073602
 H               0.235958   -4.088247   -1.712121
 H              -3.926800   -2.872796   -1.238224
 H              -0.467345   -2.074201    1.200710
 H              -5.358218   -1.654111    0.400151
 H              -1.887740   -0.795549    2.797124
 H              -4.341485   -0.527748    2.370935
 H               0.577893   -0.429498   -3.152805

 Electronic Energy =  -1448.75207130 (Hartree/Particle)
 Dipole Moment (Debye):      6.8666
 index          0
 Harmonic frequencies
 Number of imaginary frequencies=           0

 THERMOCHEMICAL DATA

 Temperature   298.150 Kelvin.  Pressure   1.00000 Atm.
 Zero-point correction=                           0.449885
(Hartree/Particle)
 Thermal correction to Energy=                    0.476964
 Thermal correction to Enthalpy=                  0.477908
 Thermal correction to Gibbs Free Energy=         0.392388
 Sum of electronic and zero-point Energies=          -1448.302186
 Sum of electronic and thermal Energies=             -1448.275108
 Sum of electronic and thermal Enthalpies=           -1448.274163
 Sum of electronic and thermal Free Energies=        -1448.359683
 PCM-SP (solvent=THF;6-311+g(2d,p)) =   -1449.11552297
 Corrected Free Energy =   -1448.72313467
```

----------------------------------------------------
----------------------------------------------------

MODEL: Az-(Cu2Cl)-Bpin

THF-(Cu2Cl)-Bpin complex

CARTESIAN COORDINATES

| | | | |
|---|---|---|---|
| Cu | -0.833728 | 1.102582 | 0.105023 |
| Cu | -0.540900 | -1.170849 | -0.063251 |
| B | 1.036585 | 0.290464 | 0.036155 |
| O | 1.818024 | 0.099455 | 1.160279 |
| O | 1.801216 | 0.468626 | -1.100956 |
| C | 3.188111 | -0.142964 | 0.708316 |
| C | 3.202355 | 0.566217 | -0.692600 |
| C | 3.530706 | 2.062475 | -0.615138 |
| H | 4.586187 | 2.228223 | -0.372808 |
| H | 3.320842 | 2.522278 | -1.586165 |
| H | 2.914847 | 2.563176 | 0.138972 |
| C | 4.064802 | -0.113883 | -1.751291 |
| H | 4.005551 | 0.445233 | -2.690900 |
| H | 5.113885 | -0.139841 | -1.432666 |
| H | 3.731101 | -1.135648 | -1.945322 |
| C | 3.348567 | -1.666244 | 0.621561 |
| H | 4.369566 | -1.946238 | 0.340268 |
| H | 3.123927 | -2.102024 | 1.599941 |
| H | 2.651255 | -2.096990 | -0.104297 |
| C | 4.152848 | 0.437275 | 1.737312 |
| H | 4.062131 | -0.113976 | 2.678896 |
| H | 5.187665 | 0.347092 | 1.385561 |
| H | 3.942047 | 1.489886 | 1.940494 |
| Cl | -1.653706 | -2.988961 | -0.225159 |
| O | -2.689389 | 1.853829 | 0.145814 |
| C | -3.487459 | 1.316948 | 1.246945 |
| C | -3.451626 | 1.528327 | -1.058133 |
| C | -4.029126 | -0.025217 | 0.733120 |
| H | -2.828643 | 1.244069 | 2.114528 |
| H | -4.283184 | 2.043923 | 1.453239 |
| C | -3.989603 | 0.111485 | -0.816752 |
| H | -4.250477 | 2.275568 | -1.147483 |
| H | -2.769346 | 1.622981 | -1.905289 |
| H | -5.036689 | -0.215267 | 1.114808 |
| H | -3.382203 | -0.852155 | 1.040718 |
| H | -4.971966 | -0.023291 | -1.279068 |
| H | -3.308470 | -0.639028 | -1.229035 |

Electronic Energy = -1498.79415092 (Hartree/Particle)
Dipole Moment (Debye): 6.7869
index 0
Harmonic frequencies
Number of imaginary frequencies= 0

THERMOCHEMICAL DATA

Temperature 298.150 Kelvin. Pressure 1.00000 Atm.
Zero-point correction= 0.303731 (Hartree/Particle)
Thermal correction to Energy= 0.325086

Thermal correction to Enthalpy= 0.326030
Thermal correction to Gibbs Free Energy= 0.249514
Sum of electronic and zero-point Energies= -1498.490420
Sum of electronic and thermal Energies= -1498.469065
Sum of electronic and thermal Enthalpies= -1498.468121
Sum of electronic and thermal Free Energies= -1498.544637
PCM-SP (solvent=THF;6-311+g(2d,p)) = -1499.03284364
Corrected Free Energy = -1498.78332972

-------------------------------------------------------

Cl-Cu-THF

CARTESIAN COORDINATES

| | | | |
|---|---|---|---|
| Cu | 1.062744 | 0.000000 | 0.000000 |
| Cl | 3.157013 | 0.000000 | 0.000000 |
| O | -0.862046 | 0.000000 | 0.000000 |
| C | -1.657972 | 1.213414 | 0.000000 |
| C | -1.657973 | -1.213414 | 0.000000 |
| C | -3.132607 | 0.776735 | 0.000000 |
| H | -1.377069 | 1.783257 | 0.890423 |
| H | -1.377069 | 1.783257 | -0.890423 |
| C | -3.132606 | -0.776734 | 0.000000 |
| H | -1.377070 | -1.783257 | -0.890423 |
| H | -1.377069 | -1.783258 | 0.890422 |
| H | -3.649301 | 1.167712 | -0.880917 |
| H | -3.649301 | 1.167713 | 0.880917 |
| H | -3.649302 | -1.167712 | -0.880916 |
| H | -3.649301 | -1.167712 | 0.880917 |

Electronic Energy = -890.093411832 (Hartree/Particle)
Dipole Moment (Debye): 10.0129
index 0
Harmonic frequencies
Number of imaginary frequencies= 2
Negatives Eigenvalues: -196.601
-193.939

THERMOCHEMICAL DATA

Temperature 298.150 Kelvin. Pressure 1.00000 Atm.
Zero-point correction= 0.120025
(Hartree/Particle)
Thermal correction to Energy= 0.127414
Thermal correction to Enthalpy= 0.128359
Thermal correction to Gibbs Free Energy= 0.084918
Sum of electronic and zero-point Energies= -889.973386
Sum of electronic and thermal Energies= -889.965997
Sum of electronic and thermal Enthalpies= -889.965053
Sum of electronic and thermal Free Energies= -890.008494
PCM-SP (solvent=THF;6-311+g(2d,p)) = -890.210751525
Corrected Free Energy = -890.125833693

-------------------------------------------------------

THF

CARTESIAN COORDINATES

| | | | |
|---|---|---|---|
| C | 1.110010 | -0.503453 | 0.199533 |
| O | -0.032909 | -1.209485 | -0.279624 |
| C | -1.166880 | -0.427611 | 0.103690 |
| C | -0.725575 | 1.057410 | 0.015153 |
| C | 0.819692 | 0.972490 | -0.115508 |
| H | 1.224249 | -0.661468 | 1.286536 |
| H | 1.989161 | -0.909054 | -0.308634 |
| H | -1.987023 | -0.688794 | -0.570743 |
| H | -1.462949 | -0.688033 | 1.133022 |
| H | -1.170085 | 1.558784 | -0.850285 |
| H | -1.030421 | 1.611974 | 0.909101 |
| H | 1.133082 | 1.202613 | -1.139318 |
| H | 1.343769 | 1.656845 | 0.560099 |

Electronic Energy = -232.466403941 (Hartree/Particle)
Dipole Moment (Debye): 1.8228
index 0
Harmonic frequencies
Number of imaginary frequencies= 0

THERMOCHEMICAL DATA

Temperature 298.150 Kelvin. Pressure 1.00000 Atm.
Zero-point correction= 0.117173
(Hartree/Particle)
Thermal correction to Energy= 0.122138
Thermal correction to Enthalpy= 0.123083
Thermal correction to Gibbs Free Energy= 0.087703
Sum of electronic and zero-point Energies= -232.349231
Sum of electronic and thermal Energies= -232.344266
Sum of electronic and thermal Enthalpies= -232.343321
Sum of electronic and thermal Free Energies= -232.378701
PCM-SP (solvent=THF;6-311+g(2d,p)) = -232.534621381
Corrected Free Energy = -232.446918440

-----------------------------------------------------

SUBSTRATE: N-PICOLINOYL-AZIRIDINE

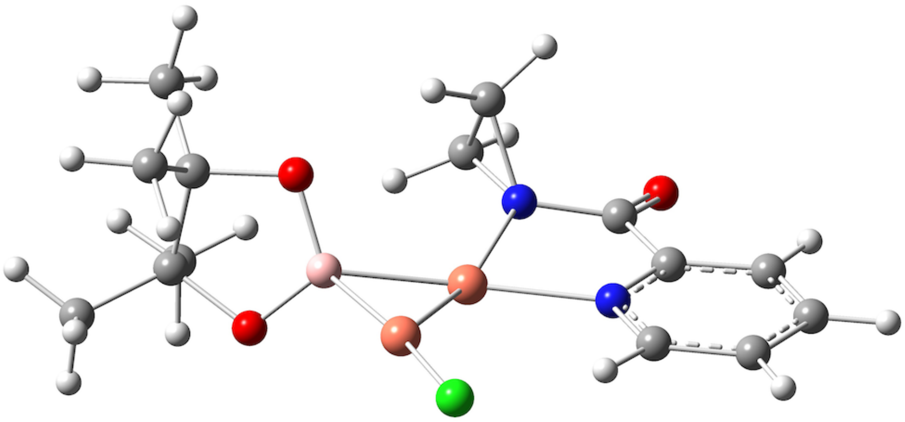

Complex-2 Substrate: N-(2-picolinoyl)-aziridine

CARTESIAN COORDINATES

| | | | |
|---|---|---|---|
| C | -0.542709 | -2.662926 | 1.258566 |

| | | | |
|---|---|---|---|
| N | -1.155239 | -2.128528 | -0.015373 |
| C | -2.575430 | -2.280262 | -0.141559 |
| O | -3.130360 | -3.349775 | -0.304831 |
| C | -0.349094 | -3.362143 | -0.030492 |
| C | -3.321888 | -0.980578 | -0.080847 |
| C | -4.713650 | -0.970393 | 0.008373 |
| C | -5.362897 | 0.265170 | 0.073534 |
| C | -4.600361 | 1.433490 | 0.047512 |
| C | -3.207985 | 1.337602 | -0.050543 |
| N | -2.587351 | 0.150731 | -0.118283 |
| Cu | 0.332869 | 2.014387 | -0.026419 |
| H | -5.253469 | -1.911019 | 0.027108 |
| H | -6.445753 | 0.312126 | 0.145970 |
| H | -2.563608 | 2.214700 | -0.071679 |
| H | -5.064816 | 2.413153 | 0.099423 |
| H | -0.897284 | -4.283501 | -0.206653 |
| H | 0.592498 | -3.246119 | -0.557266 |
| H | -1.247745 | -3.091259 | 1.967234 |
| Cu | -0.542970 | -0.090629 | -0.279139 |
| B | 1.482328 | 0.229692 | -0.143114 |
| O | 2.030928 | -0.410763 | 0.973650 |
| O | 2.413466 | 0.296315 | -1.169298 |
| C | 3.472608 | -0.511966 | 0.777380 |
| C | 3.597613 | -0.459105 | -0.787202 |
| Cl | -0.489750 | 3.994594 | 0.216554 |
| H | 0.242055 | -2.027072 | 1.659324 |
| C | 4.094086 | 0.703814 | 1.477615 |
| H | 3.794457 | 0.697460 | 2.530408 |
| H | 5.188332 | 0.682867 | 1.425846 |
| H | 3.737165 | 1.638169 | 1.033345 |
| C | 4.832163 | 0.269557 | -1.312034 |
| H | 5.748881 | -0.225236 | -0.968435 |
| H | 4.826900 | 0.260972 | -2.406991 |
| H | 4.850897 | 1.311521 | -0.985191 |
| C | 3.966212 | -1.803367 | 1.423900 |
| H | 5.034360 | -1.951822 | 1.224752 |
| H | 3.826246 | -1.749257 | 2.508856 |
| H | 3.419678 | -2.675431 | 1.055369 |
| C | 3.475748 | -1.835066 | -1.455612 |
| H | 3.361727 | -1.696040 | -2.535219 |
| H | 4.361610 | -2.453682 | -1.273513 |
| H | 2.594265 | -2.370681 | -1.089471 |

Electronic Energy = -1760.73090756 (Hartree/Particle)
Dipole Moment (Debye): 8.8294
index 0
Harmonic frequencies
Number of imaginary frequencies= 0

THERMOCHEMICAL DATA

Temperature 298.150 Kelvin. Pressure 1.00000 Atm.
Zero-point correction= 0.335852 (Hartree/Particle)
Thermal correction to Energy= 0.361316
Thermal correction to Enthalpy= 0.362260
Thermal correction to Gibbs Free Energy= 0.277426
Sum of electronic and zero-point Energies= -1760.395055
Sum of electronic and thermal Energies= -1760.369592
Sum of electronic and thermal Enthalpies= -1760.368648

Sum of electronic and thermal Free Energies=        -1760.453482
PCM-SP (solvent=THF;6-311+g(2d,p)) =  -1761.03892194
Corrected Free Energy =  -1760.76149638

---

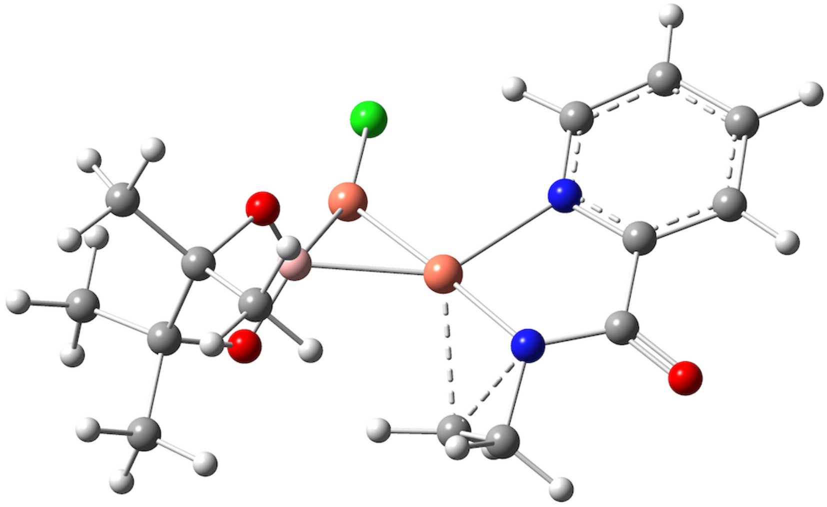

TS3     Substrate: N-(2-picolinoyl)-aziridine

CARTESIAN COORDINATES

| | | | |
|---|---|---|---|
| C | 0.158449 | -1.875530 | -1.799364 |
| N | 1.177813 | -2.151507 | -0.192739 |
| C | 2.575439 | -2.219641 | -0.165330 |
| O | 3.210548 | -3.226336 | -0.456653 |
| C | 0.486481 | -3.108989 | -1.039983 |
| C | 3.251533 | -0.963670 | 0.316683 |
| C | 4.618944 | -0.944954 | 0.582719 |
| C | 5.196648 | 0.263138 | 0.983094 |
| C | 4.394646 | 1.401031 | 1.078492 |
| C | 3.032573 | 1.298848 | 0.777161 |
| N | 2.478500 | 0.133325 | 0.419603 |
| Cu | -0.232301 | 1.879015 | -0.435811 |
| H | 5.195621 | -1.855789 | 0.462987 |
| H | 6.258819 | 0.315522 | 1.205347 |
| H | 2.369342 | 2.159556 | 0.793564 |
| H | 4.806890 | 2.361023 | 1.372606 |
| H | 1.140416 | -3.838518 | -1.520667 |
| H | -0.355437 | -3.583639 | -0.533154 |
| H | 0.865850 | -1.550291 | -2.556450 |
| Cu | 0.489332 | -0.325566 | -0.164282 |
| B | -1.537726 | 0.365377 | 0.000118 |
| O | -2.441902 | -0.108101 | -0.946355 |
| O | -2.058080 | 0.258686 | 1.279466 |
| C | -3.726169 | -0.314924 | -0.282235 |
| C | -3.287616 | -0.523107 | 1.209731 |
| Cl | 0.708874 | 3.794117 | -0.712525 |
| H | -0.850785 | -1.473877 | -1.837696 |
| C | -4.266972 | 0.007119 | 2.251963 |
| H | -5.234585 | -0.501994 | 2.165358 |
| H | -3.871715 | -0.175707 | 3.256556 |
| H | -4.424391 | 1.082460 | 2.142961 |
| C | -2.897082 | -1.972135 | 1.531387 |
| H | -2.409302 | -1.998504 | 2.510720 |

| | | | |
|---|---|---|---|
| H | -3.772108 | -2.630715 | 1.557991 |
| H | -2.186966 | -2.359593 | 0.792893 |
| C | -4.546394 | 0.961964 | -0.502516 |
| H | -4.633373 | 1.146457 | -1.577875 |
| H | -5.553660 | 0.868552 | -0.081905 |
| H | -4.054565 | 1.828869 | -0.050469 |
| C | -4.420516 | -1.511245 | -0.925846 |
| H | -5.348122 | -1.750712 | -0.392315 |
| H | -4.675023 | -1.275935 | -1.964612 |
| H | -3.781577 | -2.398236 | -0.926259 |

Electronic Energy = -1760.69923010 (Hartree/Particle)
Dipole Moment (Debye): 6.6275
index 0
Harmonic frequencies
Number of imaginary frequencies= 1
Negatives Eigenvalues: -250.419

THERMOCHEMICAL DATA

Temperature 298.150 Kelvin. Pressure 1.00000 Atm.
Zero-point correction= 0.334219
(Hartree/Particle)
Thermal correction to Energy= 0.359120
Thermal correction to Enthalpy= 0.360064
Thermal correction to Gibbs Free Energy= 0.277475
Sum of electronic and zero-point Energies= -1760.365011
Sum of electronic and thermal Energies= -1760.340110
Sum of electronic and thermal Enthalpies= -1760.339166
Sum of electronic and thermal Free Energies= -1760.421755
PCM-SP (solvent=THF;6-311+g(2d,p)) = -1761.00756245
Corrected Free Energy = -1760.73008735

-------------------------------------------------------

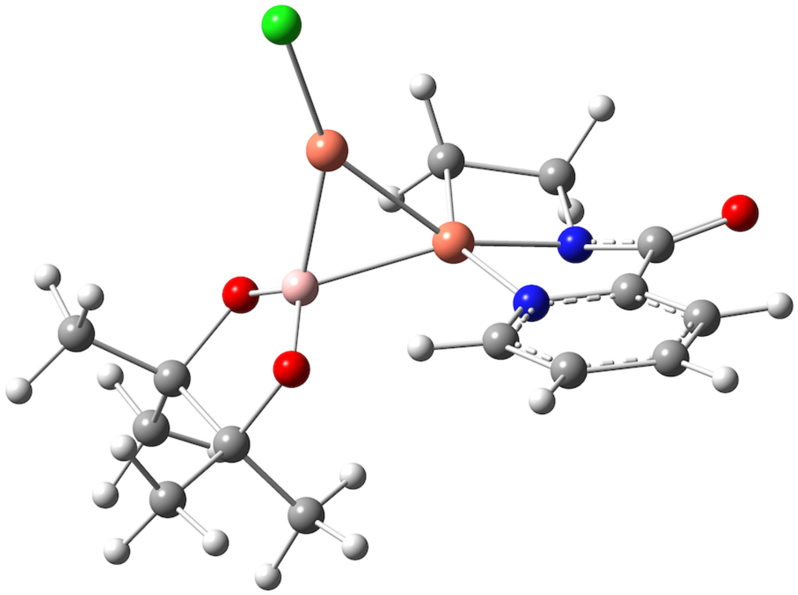

I2 Substrate: N-(2-picolinoyl)-aziridine

CARTESIAN COORDINATES

| | | | |
|---|---|---|---|
| C | 0.543509 | 1.455816 | -2.310895 |
| N | 2.104637 | -0.048611 | -1.870806 |

| | | | |
|---|---|---|---|
| C | 3.287260 | -0.323275 | -1.266037 |
| O | 4.417673 | -0.139136 | -1.715004 |
| C | 1.864794 | 0.955508 | -2.902785 |
| C | 3.055005 | -0.938667 | 0.108640 |
| C | 4.074158 | -1.539606 | 0.839542 |
| C | 3.775369 | -2.044123 | 2.107926 |
| C | 2.474954 | -1.924015 | 2.605367 |
| C | 1.508043 | -1.294462 | 1.820276 |
| N | 1.802992 | -0.820415 | 0.602439 |
| Cu | -0.458564 | 1.784597 | 0.688350 |
| H | 5.068024 | -1.587504 | 0.406843 |
| H | 4.548755 | -2.520715 | 2.703950 |
| H | 0.479943 | -1.162525 | 2.140318 |
| H | 2.208916 | -2.301270 | 3.587548 |
| H | 2.652474 | 1.717742 | -2.932501 |
| H | 1.765370 | 0.513248 | -3.901323 |
| H | 0.521328 | 2.477400 | -1.931132 |
| Cu | 0.699510 | 0.280272 | -0.699454 |
| B | -1.352322 | 0.114069 | -0.054363 |
| O | -2.340004 | 0.250280 | -1.001195 |
| O | -1.554022 | -0.974534 | 0.770287 |
| C | -3.429572 | -0.657036 | -0.628467 |
| C | -2.672002 | -1.745028 | 0.214403 |
| Cl | -0.467064 | 3.492789 | 1.951594 |
| H | -0.385623 | 1.152621 | -2.792270 |
| C | -3.471396 | -2.341264 | 1.367137 |
| H | -4.369158 | -2.844911 | 0.989530 |
| H | -2.862571 | -3.081605 | 1.896689 |
| H | -3.774293 | -1.575285 | 2.084423 |
| C | -2.043988 | -2.851813 | -0.640328 |
| H | -1.369330 | -3.444885 | -0.014724 |
| H | -2.807126 | -3.518226 | -1.056270 |
| H | -1.458743 | -2.430806 | -1.464494 |
| C | -4.422248 | 0.169898 | 0.196150 |
| H | -4.751044 | 1.026453 | -0.400151 |
| H | -5.302051 | -0.422024 | 0.470564 |
| H | -3.956674 | 0.554897 | 1.108865 |
| C | -4.086587 | -1.172658 | -1.903955 |
| H | -4.847731 | -1.924876 | -1.664828 |
| H | -4.576554 | -0.344794 | -2.426400 |
| H | -3.355987 | -1.617257 | -2.583879 |

Electronic Energy = -1760.71306275 (Hartree/Particle)
Dipole Moment (Debye): 8.7512
index 0
Harmonic frequencies
Number of imaginary frequencies= 0

THERMOCHEMICAL DATA

Temperature 298.150 Kelvin. Pressure 1.00000 Atm.
Zero-point correction= 0.335220 (Hartree/Particle)
Thermal correction to Energy= 0.360420
Thermal correction to Enthalpy= 0.361364
Thermal correction to Gibbs Free Energy= 0.277322
Sum of electronic and zero-point Energies= -1760.377842
Sum of electronic and thermal Energies= -1760.352643
Sum of electronic and thermal Enthalpies= -1760.351698
Sum of electronic and thermal Free Energies= -1760.435740

PCM-SP (solvent=THF;6-311+g(2d,p)) = -1761.02236686
Corrected Free Energy = -1760.74504404

-------------------------------------------------------

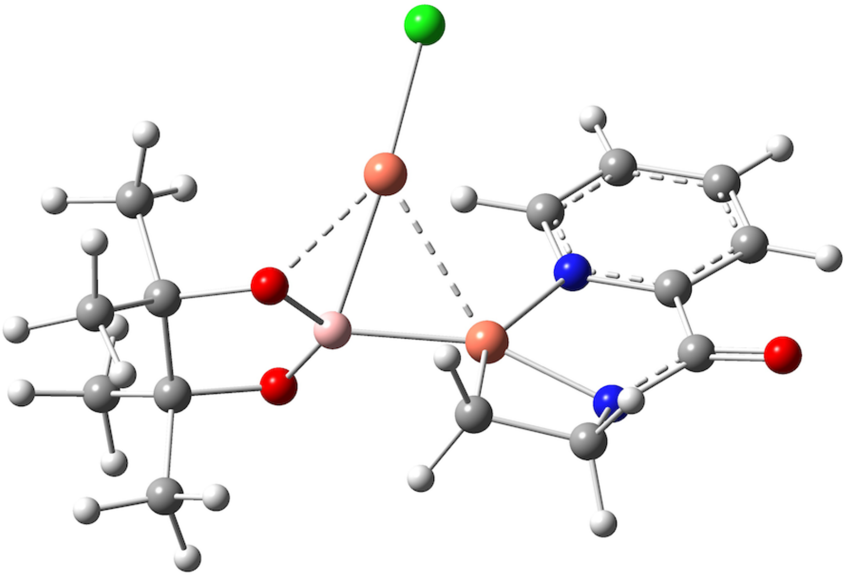

TS4 Substrate: N-(2-picolinoyl)-aziridine

CARTESIAN COORDINATES

| | | | |
|---|---|---|---|
| N | -2.100287 | 0.290725 | 0.754959 |
| C | -3.304581 | -0.267376 | 0.488042 |
| C | -4.494711 | 0.384427 | 0.789447 |
| C | -4.436181 | 1.638037 | 1.403426 |
| C | -3.190828 | 2.205166 | 1.678578 |
| C | -2.039532 | 1.502252 | 1.325043 |
| C | -3.276817 | -1.615858 | -0.222265 |
| O | -4.277090 | -2.082718 | -0.770962 |
| Cu | -0.629187 | -0.936290 | 0.034329 |
| N | -2.041223 | -2.150160 | -0.140512 |
| C | -1.435672 | -3.119524 | -1.044920 |
| C | -0.022934 | -2.511278 | -1.036204 |
| B | 1.314173 | -0.396091 | 0.194877 |
| Cu | 0.421379 | 1.168822 | -0.997942 |
| Cl | -0.444760 | 3.077071 | -1.372780 |
| O | 2.222218 | -0.164770 | -0.837061 |
| C | 3.520002 | 0.175727 | -0.208055 |
| C | 4.628738 | -0.365023 | -1.101572 |
| O | 1.924814 | -0.492018 | 1.414822 |
| C | 3.377565 | -0.528203 | 1.187689 |
| C | 3.776938 | -2.007380 | 1.168451 |
| C | 3.580121 | 1.702623 | -0.127722 |
| C | 4.058843 | 0.198857 | 2.341183 |
| H | -5.429809 | -0.098000 | 0.524619 |
| H | -5.349944 | 2.170829 | 1.651955 |
| H | -1.047815 | 1.915593 | 1.465416 |
| H | -3.100928 | 3.185473 | 2.134624 |
| H | -1.473886 | -4.144496 | -0.653981 |
| H | 0.736604 | -3.061981 | -0.479514 |
| H | 0.357459 | -2.120650 | -1.980098 |
| H | 4.542088 | 2.040202 | 0.271445 |
| H | 3.452028 | 2.119648 | -1.131238 |
| H | 2.777559 | 2.101949 | 0.500639 |

| | | | |
|---|---|---|---|
| H | 4.633481 | 0.176872 | -2.052530 |
| H | 5.604508 | -0.223509 | -0.622083 |
| H | 4.494832 | -1.427528 | -1.316479 |
| H | 3.899168 | -0.355742 | 3.271266 |
| H | 5.138647 | 0.269122 | 2.164137 |
| H | 3.658680 | 1.206303 | 2.474533 |
| H | 4.860638 | -2.126730 | 1.064208 |
| H | 3.464644 | -2.471159 | 2.109120 |
| H | 3.287200 | -2.540970 | 0.347218 |
| H | -1.903336 | -3.110968 | -2.037872 |

Electronic Energy = -1760.70515508 (Hartree/Particle)
Dipole Moment (Debye): 7.3206
index 0
Harmonic frequencies
Number of imaginary frequencies= 1
Negatives Eigenvalues: -78.759

THERMOCHEMICAL DATA

Temperature 298.150 Kelvin. Pressure 1.00000 Atm.
Zero-point correction= 0.334875
(Hartree/Particle)
Thermal correction to Energy= 0.359345
Thermal correction to Enthalpy= 0.360290
Thermal correction to Gibbs Free Energy= 0.278794
Sum of electronic and zero-point Energies= -1760.370280
Sum of electronic and thermal Energies= -1760.345810
Sum of electronic and thermal Enthalpies= -1760.344866
Sum of electronic and thermal Free Energies= -1760.426361
PCM-SP (solvent=THF;6-311+g(2d,p)) = -1761.01401254
Corrected Free Energy = -1760.73521846

---

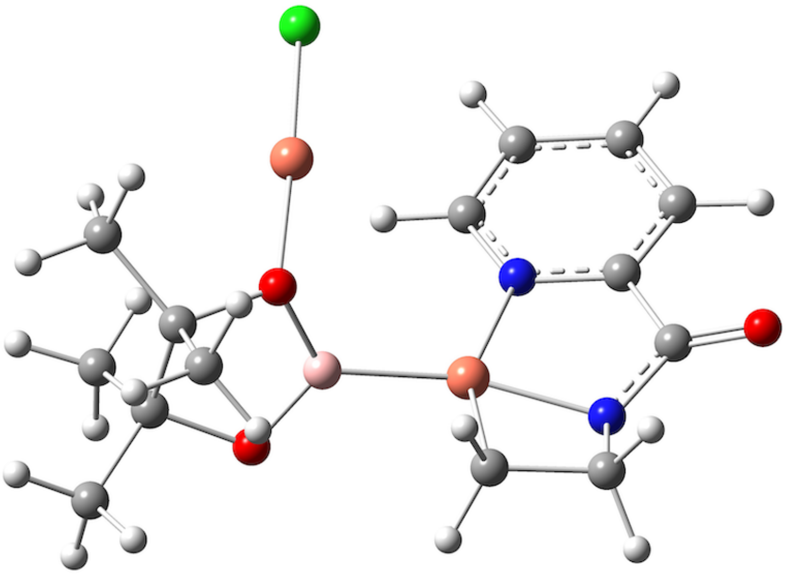

I3 Substrate: N-(2-picolinoyl)-aziridine

CARTESIAN COORDINATES

| | | | |
|---|---|---|---|
| C | -1.891963 | -3.351253 | -0.697271 |
| Cu | -0.856482 | -1.243462 | 0.283068 |

| | | | |
|---|---|---|---|
| B | 1.064280 | -0.843109 | 0.325983 |
| O | 1.734952 | -0.056576 | -0.652584 |
| C | 3.198178 | -0.351491 | -0.529570 |
| C | 3.283065 | -0.759259 | 0.975253 |
| O | 1.966229 | -1.361454 | 1.207323 |
| Cu | 1.013390 | 1.742606 | -0.713238 |
| Cl | 0.276893 | 3.709918 | -0.604308 |
| C | -0.399450 | -2.999230 | -0.521252 |
| N | -2.447133 | -2.199304 | 0.008223 |
| C | -3.500874 | -1.453234 | -0.370113 |
| C | -3.361709 | -0.043993 | 0.203375 |
| C | -4.422494 | 0.853988 | 0.221136 |
| C | -4.206817 | 2.143880 | 0.714117 |
| C | -2.933727 | 2.498173 | 1.161417 |
| C | -1.913870 | 1.549129 | 1.099766 |
| N | -2.124105 | 0.309219 | 0.634976 |
| O | -4.462426 | -1.765434 | -1.079059 |
| H | -5.381479 | 0.525345 | -0.165739 |
| H | -5.018118 | 2.866393 | 0.735811 |
| H | -0.900542 | 1.790849 | 1.397389 |
| H | -2.710504 | 3.495717 | 1.523793 |
| H | -2.166952 | -4.305404 | -0.228580 |
| H | 0.151638 | -3.591230 | 0.211044 |
| H | 0.169613 | -2.856325 | -1.441823 |
| C | 3.997043 | 0.880633 | -0.926124 |
| H | 5.067407 | 0.684590 | -0.794243 |
| H | 3.820360 | 1.118251 | -1.979937 |
| H | 3.732456 | 1.758574 | -0.331652 |
| C | 3.455589 | -1.505105 | -1.500178 |
| H | 3.155314 | -1.200181 | -2.507242 |
| H | 4.517980 | -1.768354 | -1.519507 |
| H | 2.881692 | -2.395321 | -1.224818 |
| C | 3.395236 | 0.441301 | 1.920811 |
| H | 3.223255 | 0.100324 | 2.946019 |
| H | 4.388503 | 0.899302 | 1.868155 |
| H | 2.646518 | 1.205985 | 1.685682 |
| C | 4.349143 | -1.802222 | 1.289799 |
| H | 5.344859 | -1.422544 | 1.032008 |
| H | 4.335589 | -2.028547 | 2.360462 |
| H | 4.174620 | -2.733072 | 0.745231 |
| H | -2.206601 | -3.382176 | -1.748629 |

Electronic Energy = -1760.71525541 (Hartree/Particle)
Dipole Moment (Debye): 7.2813
index 0
Harmonic frequencies
Number of imaginary frequencies= 0

THERMOCHEMICAL DATA

Temperature 298.150 Kelvin. Pressure 1.00000 Atm.
Zero-point correction= 0.335833
(Hartree/Particle)
Thermal correction to Energy= 0.360675
Thermal correction to Enthalpy= 0.361619
Thermal correction to Gibbs Free Energy= 0.278963
Sum of electronic and zero-point Energies= -1760.379422
Sum of electronic and thermal Energies= -1760.354581
Sum of electronic and thermal Enthalpies= -1760.353637
Sum of electronic and thermal Free Energies= -1760.436292

PCM-SP (solvent=THF;6-311+g(2d,p)) = -1761.02421944
Corrected Free Energy = -1760.74525603

------------------------------------------------------

TS5 Substrate: N-(2-picolinoyl)-aziridine

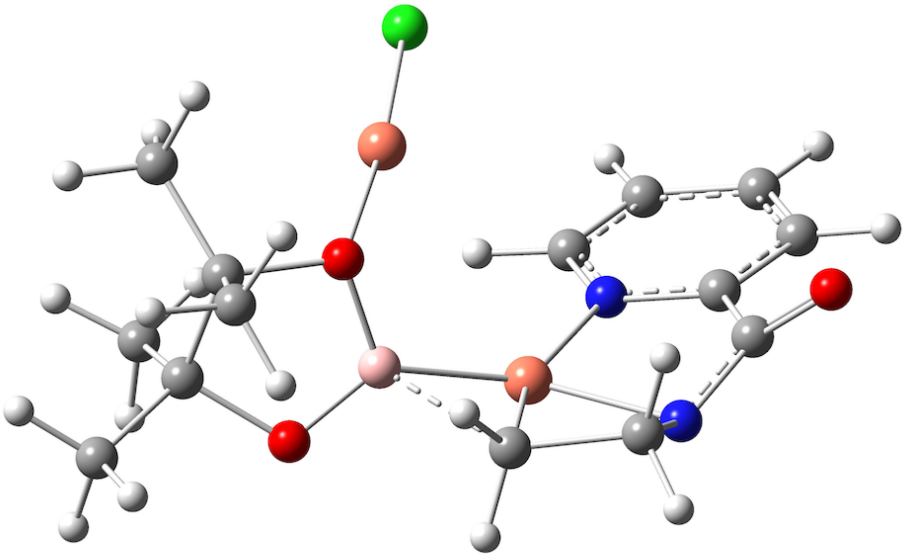

CARTESIAN COORDINATES

| | | | |
|---|---|---|---|
| C | -0.970595 | -3.159042 | -0.728286 |
| Cu | -0.681748 | -1.094072 | 0.591138 |
| B | 1.247819 | -0.880856 | 0.331049 |
| O | 1.731145 | -0.084541 | -0.744432 |
| C | 3.220107 | -0.012842 | -0.592490 |
| C | 3.369381 | -0.225359 | 0.951699 |
| O | 2.229658 | -1.091504 | 1.259199 |
| Cu | 0.603790 | 1.513936 | -0.784849 |
| Cl | -0.440746 | 3.337481 | -0.689218 |
| C | 0.373003 | -2.650978 | -0.104653 |
| N | -1.983416 | -2.411904 | 0.019840 |
| C | -2.984521 | -1.767115 | -0.600969 |
| C | -3.288180 | -0.441246 | 0.109069 |
| C | -4.524490 | 0.190349 | 0.017202 |
| C | -4.695337 | 1.438409 | 0.618727 |
| C | -3.613286 | 2.038029 | 1.266442 |
| C | -2.392608 | 1.368249 | 1.290509 |
| N | -2.238881 | 0.153872 | 0.739020 |
| O | -3.584613 | -2.043437 | -1.649469 |
| H | -5.315525 | -0.304580 | -0.536113 |
| H | -5.655031 | 1.945794 | 0.568359 |
| H | -1.505967 | 1.814823 | 1.723787 |
| H | -3.693628 | 3.021300 | 1.717733 |
| H | -1.041709 | -4.249836 | -0.605718 |
| H | 0.662475 | -3.232283 | 0.774952 |
| H | 1.189914 | -2.667179 | -0.832395 |
| C | 3.713414 | 1.324088 | -1.123895 |
| H | 4.796081 | 1.403220 | -0.973006 |
| H | 3.510617 | 1.397876 | -2.196892 |
| H | 3.234327 | 2.172490 | -0.628940 |

| | | | |
|---|---|---|---|
| C | 3.769704 | -1.166850 | -1.431538 |
| H | 3.419361 | -1.060411 | -2.462640 |
| H | 4.864241 | -1.155726 | -1.434294 |
| H | 3.440566 | -2.138777 | -1.052056 |
| C | 3.187389 | 1.062068 | 1.762946 |
| H | 3.094154 | 0.800850 | 2.821233 |
| H | 4.045500 | 1.731456 | 1.643721 |
| H | 2.281856 | 1.602194 | 1.465599 |
| C | 4.645576 | -0.949275 | 1.366891 |
| H | 5.527717 | -0.374925 | 1.060865 |
| H | 4.667787 | -1.056268 | 2.455749 |
| H | 4.704801 | -1.947777 | 0.927861 |
| H | -1.023925 | -2.940027 | -1.803051 |

Electronic Energy = -1760.70833509 (Hartree/Particle)
Dipole Moment (Debye): 8.2841
index 0
Harmonic frequencies
Number of imaginary frequencies= 1
Negatives Eigenvalues: -142.730

THERMOCHEMICAL DATA

Temperature 298.150 Kelvin. Pressure 1.00000 Atm.
Zero-point correction= 0.335397 (Hartree/Particle)
Thermal correction to Energy= 0.359422
Thermal correction to Enthalpy= 0.360366
Thermal correction to Gibbs Free Energy= 0.280878
Sum of electronic and zero-point Energies= -1760.372938
Sum of electronic and thermal Energies= -1760.348914
Sum of electronic and thermal Enthalpies= -1760.347969
Sum of electronic and thermal Free Energies= -1760.427457
PCM-SP (solvent=THF;6-311+g(2d,p)) = -1761.02086132
Corrected Free Energy = -1760.73998323

------------------------------------------------------

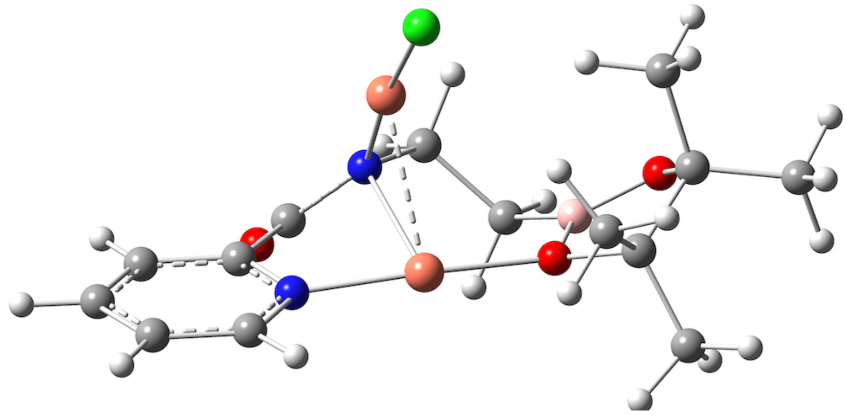

P2 Substrate: N-(2-picolinoyl)-aziridine

CARTESIAN COORDINATES

| | | | |
|---|---|---|---|
| C | -0.070191 | -2.352295 | -1.352086 |
| Cu | -0.378070 | 0.106828 | 0.976671 |
| B | 1.890236 | -1.540129 | 0.161698 |
| O | 3.181716 | -1.408897 | -0.240285 |
| C | 3.635354 | -0.041736 | 0.027538 |

| | | | |
|---|---|---|---|
| C | 2.692794 | 0.413978 | 1.198690 |
| O | 1.498603 | -0.436267 | 0.947713 |
| Cu | -0.535985 | 0.493520 | -1.572702 |
| Cl | 0.084588 | 2.462996 | -2.067859 |
| C | 0.925930 | -2.718295 | -0.212518 |
| N | -0.968834 | -1.258650 | -0.947311 |
| C | -2.159055 | -1.637799 | -0.424154 |
| C | -2.959638 | -0.528077 | 0.243425 |
| C | -4.347951 | -0.481046 | 0.114322 |
| C | -5.057745 | 0.527274 | 0.762329 |
| C | -4.363095 | 1.446728 | 1.554880 |
| C | -2.983664 | 1.317673 | 1.671667 |
| N | -2.294312 | 0.355063 | 1.028223 |
| O | -2.640013 | -2.780931 | -0.381210 |
| H | -4.836087 | -1.237015 | -0.490762 |
| H | -6.136760 | 0.596306 | 0.655771 |
| H | -2.396570 | 1.991029 | 2.286716 |
| H | -4.876732 | 2.245716 | 2.079734 |
| H | -0.659066 | -3.237519 | -1.612411 |
| H | 0.331563 | -3.032394 | 0.656206 |
| H | 1.521664 | -3.582869 | -0.533113 |
| C | 3.420477 | 0.749261 | -1.265265 |
| H | 3.798339 | 1.772599 | -1.173540 |
| H | 3.960994 | 0.250191 | -2.075483 |
| H | 2.363310 | 0.807732 | -1.541051 |
| C | 5.114693 | -0.090214 | 0.394635 |
| H | 5.693346 | -0.419716 | -0.473790 |
| H | 5.469188 | 0.906015 | 0.683342 |
| H | 5.308743 | -0.785597 | 1.214794 |
| C | 2.258040 | 1.872066 | 1.150333 |
| H | 1.598425 | 2.087957 | 1.999313 |
| H | 3.131803 | 2.528754 | 1.228064 |
| H | 1.720170 | 2.108411 | 0.228460 |
| C | 3.187024 | 0.022985 | 2.591185 |
| H | 4.048826 | 0.630659 | 2.884991 |
| H | 2.383656 | 0.189974 | 3.316082 |
| H | 3.471822 | -1.032984 | 2.633024 |
| H | 0.483532 | -2.036776 | -2.242350 |

Electronic Energy = -1760.81585981 (Hartree/Particle)
Dipole Moment (Debye): 6.7494
index 0
Harmonic frequencies
Number of imaginary frequencies= 0

THERMOCHEMICAL DATA

Temperature 298.150 Kelvin. Pressure 1.00000 Atm.
Zero-point correction= 0.338359 (Hartree/Particle)
Thermal correction to Energy= 0.362615
Thermal correction to Enthalpy= 0.363559
Thermal correction to Gibbs Free Energy= 0.284403
Sum of electronic and zero-point Energies= -1760.477501
Sum of electronic and thermal Energies= -1760.453245
Sum of electronic and thermal Enthalpies= -1760.452300
Sum of electronic and thermal Free Energies= -1760.531457
PCM-SP (solvent=THF;6-311+g(2d,p)) = -1761.12387972
Corrected Free Energy = -1760.83947691

------------------------------------------------------------

SUBSTITUTION OF CHLORIDE WITH ALKOXIDE ANION (REF 30, MAIN TEXT, TABLE S1)

Complex-2 (Ref 33)   Substrate:  N-(2picolinoyl)-aziridine
                     Alkoxide =  Methoxide

CARTESIAN COORDINATES

| | | | |
|---|---|---|---|
| C | -0.464538 | -2.792596 | 1.058214 |
| N | -1.116269 | -2.175923 | -0.157270 |
| C | -2.543619 | -2.319220 | -0.242166 |
| O | -3.100970 | -3.377926 | -0.459882 |
| C | -0.314904 | -3.406339 | -0.280158 |
| C | -3.286800 | -1.030181 | -0.056949 |
| C | -4.672964 | -1.030943 | 0.098084 |
| C | -5.314324 | 0.196614 | 0.284344 |
| C | -4.550389 | 1.364076 | 0.309722 |
| C | -3.162554 | 1.283824 | 0.138230 |
| N | -2.553861 | 0.101696 | -0.047749 |
| Cu | 0.241600 | 1.978102 | 0.022459 |
| H | -5.213830 | -1.970914 | 0.074152 |
| H | -6.392614 | 0.237162 | 0.411147 |
| H | -2.510978 | 2.164106 | 0.150794 |
| H | -5.012156 | 2.335394 | 0.458040 |
| H | -0.871682 | -4.313852 | -0.496958 |
| H | 0.608539 | -3.257898 | -0.830603 |
| H | -1.147921 | -3.267452 | 1.758528 |
| Cu | -0.500919 | -0.149243 | -0.313134 |
| B | 1.516709 | 0.314551 | -0.096922 |
| O | 2.035613 | -0.386974 | 1.002319 |
| O | 2.486252 | 0.430162 | -1.087831 |
| C | 3.479001 | -0.490521 | 0.841162 |
| C | 3.647278 | -0.360006 | -0.714065 |
| H | 0.331677 | -2.183407 | 1.477890 |
| C | 4.093146 | 0.682082 | 1.618098 |
| H | 3.764199 | 0.626540 | 2.660738 |
| H | 5.188363 | 0.652361 | 1.595165 |
| H | 3.756441 | 1.640454 | 1.210791 |
| C | 4.907617 | 0.372339 | -1.168292 |
| H | 5.806951 | -0.154120 | -0.825449 |
| H | 4.932729 | 0.418421 | -2.262158 |
| H | 4.933541 | 1.396327 | -0.789028 |
| C | 3.946014 | -1.817318 | 1.434517 |
| H | 5.018142 | -1.965371 | 1.256949 |
| H | 3.776575 | -1.817263 | 2.516724 |
| H | 3.403117 | -2.664631 | 1.007352 |
| C | 3.524134 | -1.699977 | -1.453184 |
| H | 3.439406 | -1.505537 | -2.526992 |
| H | 4.395918 | -2.341158 | -1.280588 |
| H | 2.625145 | -2.237939 | -1.136011 |
| O | -0.816721 | 3.459516 | 0.309901 |
| C | -0.652954 | 4.699784 | -0.318707 |
| H | -1.501627 | 5.365669 | -0.073895 |
| H | 0.266820 | 5.221530 | 0.005907 |
| H | -0.607396 | 4.623563 | -1.422336 |

Electronic Energy =  -1415.60696054 (Hartree/Particle)
Dipole Moment (Debye):      6.3125

index 0
Harmonic frequencies
Number of imaginary frequencies= 0

THERMOCHEMICAL DATA

Temperature 298.150 Kelvin. Pressure 1.00000 Atm.
Zero-point correction= 0.375106
(Hartree/Particle)
Thermal correction to Energy= 0.402580
Thermal correction to Enthalpy= 0.403525
Thermal correction to Gibbs Free Energy= 0.314215
Sum of electronic and zero-point Energies= -1415.231855
Sum of electronic and thermal Energies= -1415.204380
Sum of electronic and thermal Enthalpies= -1415.203436
Sum of electronic and thermal Free Energies= -1415.292746
PCM-SP (solvent=THF;6-311+g(2d,p)) = -1415.91385033
Corrected Free Energy = -1415.59963579

-------------------------------------------------------

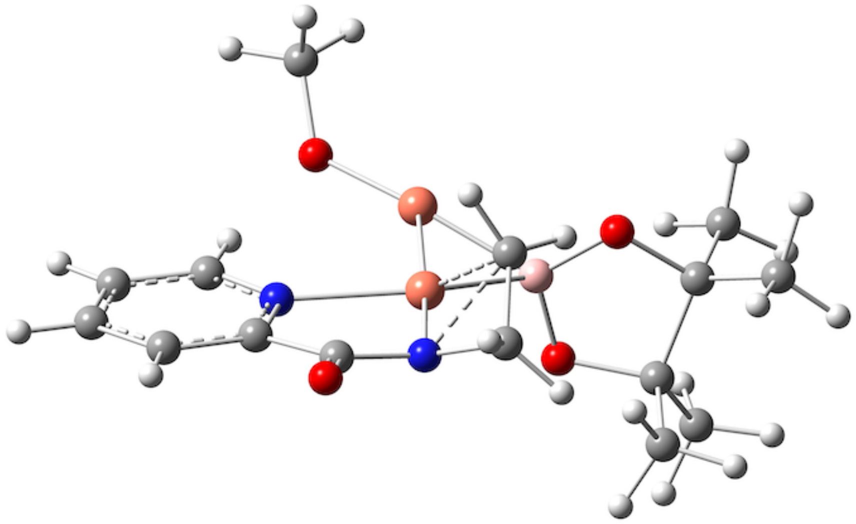

TS3 (Ref 33) Substrate: N-(2picolinoyl)-aziridine
Alkoxide = Methoxide

CARTESIAN COORDINATES

| | | | |
|---|---|---|---|
| C | 0.076705 | -1.911054 | -1.736694 |
| N | 1.109200 | -2.186978 | -0.153108 |
| C | 2.506041 | -2.298574 | -0.147079 |
| O | 3.098689 | -3.337804 | -0.413726 |
| C | 0.375026 | -3.143560 | -0.963419 |
| C | 3.235525 | -1.050953 | 0.273543 |
| C | 4.608110 | -1.073735 | 0.512445 |
| C | 5.236698 | 0.128148 | 0.850143 |
| C | 4.478970 | 1.298481 | 0.906794 |
| C | 3.106914 | 1.241154 | 0.633579 |
| N | 2.506033 | 0.077408 | 0.343464 |
| Cu | -0.166547 | 1.881830 | -0.196704 |
| H | 5.148759 | -2.009674 | 0.421572 |
| H | 6.303885 | 0.149621 | 1.053602 |
| H | 2.470776 | 2.129079 | 0.609462 |
| H | 4.933854 | 2.252998 | 1.153017 |

| | | | |
|---|---|---|---|
| H | 0.998713 | -3.900983 | -1.440844 |
| H | -0.470773 | -3.582894 | -0.432283 |
| H | 0.778115 | -1.627448 | -2.515509 |
| Cu | 0.467511 | -0.347000 | -0.129975 |
| B | -1.565016 | 0.440770 | 0.043228 |
| O | -2.441865 | 0.046484 | -0.969632 |
| O | -2.140077 | 0.233980 | 1.290602 |
| C | -3.749317 | -0.197782 | -0.372437 |
| C | -3.370682 | -0.523362 | 1.114738 |
| H | -0.916310 | -1.468631 | -1.760724 |
| C | -4.386078 | -0.063796 | 2.156735 |
| H | -5.354514 | -0.552554 | 1.993967 |
| H | -4.032047 | -0.328410 | 3.158564 |
| H | -4.528700 | 1.018710 | 2.126082 |
| C | -3.009467 | -1.998362 | 1.339643 |
| H | -2.557105 | -2.104913 | 2.330488 |
| H | -3.891689 | -2.645724 | 1.284993 |
| H | -2.277447 | -2.337833 | 0.599406 |
| C | -4.554489 | 1.099177 | -0.523542 |
| H | -4.600424 | 1.366016 | -1.584208 |
| H | -5.577351 | 0.983188 | -0.148315 |
| H | -4.073891 | 1.924003 | 0.011718 |
| C | -4.428468 | -1.334266 | -1.131451 |
| H | -5.378012 | -1.604333 | -0.653847 |
| H | -4.640222 | -1.018087 | -2.158388 |
| H | -3.796954 | -2.225592 | -1.175690 |
| O | 0.882489 | 3.378967 | -0.108019 |
| C | 1.018278 | 4.351025 | -1.106879 |
| H | 0.079342 | 4.905180 | -1.289085 |
| H | 1.778963 | 5.096030 | -0.810885 |
| H | 1.337048 | 3.927046 | -2.078549 |

Electronic Energy = -1415.57769795 (Hartree/Particle)
Dipole Moment (Debye): 4.3192
index 0
Harmonic frequencies
Number of imaginary frequencies= 1
Negatives Eigenvalues: -256.540

THERMOCHEMICAL DATA

Temperature 298.150 Kelvin. Pressure 1.00000 Atm.
Zero-point correction= 0.373554 (Hartree/Particle)
Thermal correction to Energy= 0.400500
Thermal correction to Enthalpy= 0.401444
Thermal correction to Gibbs Free Energy= 0.313876
Sum of electronic and zero-point Energies= -1415.204144
Sum of electronic and thermal Energies= -1415.177198
Sum of electronic and thermal Enthalpies= -1415.176254
Sum of electronic and thermal Free Energies= -1415.263822
PCM-SP (solvent=THF;6-311+g(2d,p)) = -1415.88517817
Corrected Free Energy = -1415.57130222

-------------------------------------------------------

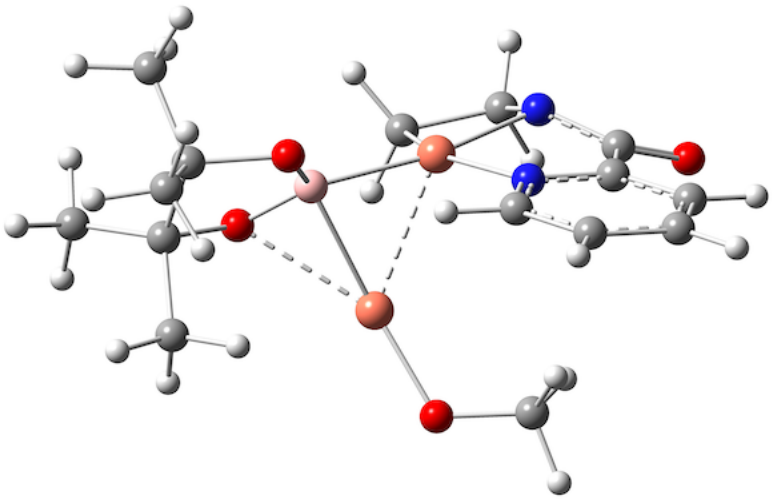

TS4 (Ref 33) Substrate: N-(2picolinoyl)-aziridine
Alkoxide = Methoxide

CARTESIAN COORDINATES

| | | | |
|---|---|---|---|
| N | 1.706603 | 0.775618 | -0.827387 |
| C | 3.011617 | 0.438676 | -0.730982 |
| C | 4.012582 | 1.405205 | -0.724480 |
| C | 3.646817 | 2.747619 | -0.839603 |
| C | 2.293610 | 3.083542 | -0.941902 |
| C | 1.345120 | 2.061957 | -0.920991 |
| C | 3.306366 | -1.049201 | -0.551871 |
| O | 4.408826 | -1.442929 | -0.165122 |
| Cu | 0.590499 | -0.897140 | -0.559400 |
| N | 2.212470 | -1.778379 | -0.859665 |
| C | 1.868574 | -3.097927 | -0.332490 |
| C | 0.368220 | -2.830052 | -0.133445 |
| B | -1.340697 | -0.306285 | -0.341275 |
| Cu | -0.362419 | -0.091775 | 1.566672 |
| O | -2.325852 | -0.841334 | 0.477652 |
| C | -3.539487 | -0.018120 | 0.294797 |
| C | -4.753006 | -0.929373 | 0.431668 |
| O | -1.839054 | 0.614395 | -1.230673 |
| C | -3.305322 | 0.573457 | -1.141679 |
| C | -3.784069 | -0.347873 | -2.268635 |
| C | -3.522605 | 1.034608 | 1.406508 |
| C | -3.835083 | 1.988831 | -1.343242 |
| H | 5.042819 | 1.083366 | -0.613924 |
| H | 4.405869 | 3.525082 | -0.837922 |
| H | 0.277290 | 2.249514 | -0.971401 |
| H | 1.972292 | 4.117077 | -1.021636 |
| H | 2.081383 | -3.906774 | -1.043557 |
| H | -0.307437 | -3.246634 | -0.882039 |
| H | -0.028959 | -2.921054 | 0.877297 |
| H | -4.422525 | 1.657743 | 1.377284 |
| H | -3.480164 | 0.528801 | 2.375994 |
| H | -2.641338 | 1.680290 | 1.332765 |
| H | -4.819989 | -1.302079 | 1.458652 |
| H | -5.671964 | -0.374524 | 0.208138 |
| H | -4.691452 | -1.789981 | -0.238162 |
| H | -3.616582 | 2.322031 | -2.362970 |
| H | -4.921820 | 2.014895 | -1.199749 |
| H | -3.373656 | 2.694369 | -0.648562 |

| | | | |
|---|---|---|---|
| H | -4.877725 | -0.388435 | -2.313463 |
| H | -3.411098 | 0.036131 | -3.223180 |
| H | -3.400793 | -1.365109 | -2.139370 |
| H | 2.395824 | -3.313668 | 0.605105 |
| O | 0.467593 | 0.841271 | 2.879802 |
| C | 1.841915 | 1.103366 | 2.747055 |
| H | 2.448794 | 0.191108 | 2.605927 |
| H | 2.068136 | 1.776929 | 1.899563 |
| H | 2.205161 | 1.600706 | 3.662560 |

Electronic Energy = -1415.58241718 (Hartree/Particle)
Dipole Moment (Debye): 8.0259
index 0
Harmonic frequencies
Number of imaginary frequencies= 1
Negatives Eigenvalues: -83.050

THERMOCHEMICAL DATA

Temperature 298.150 Kelvin. Pressure 1.00000 Atm.
Zero-point correction= 0.374539 (Hartree/Particle)
Thermal correction to Energy= 0.400817
Thermal correction to Enthalpy= 0.401761
Thermal correction to Gibbs Free Energy= 0.316848
Sum of electronic and zero-point Energies= -1415.207878
Sum of electronic and thermal Energies= -1415.181601
Sum of electronic and thermal Enthalpies= -1415.180656
Sum of electronic and thermal Free Energies= -1415.265569
PCM-SP (solvent=THF;6-311+g(2d,p)) = -1415.89420375
Corrected Free Energy = -1415.57735557

-------------------------------------------------------

Complex-2 (ref 33) Substrate: N-(2picolinoyl)-aziridine
Alkoxide = t-Butoxide

CARTESIAN COORDINATES

| | | | |
|---|---|---|---|
| C | 0.401004 | -3.365476 | 0.920998 |
| N | -0.409839 | -2.836051 | -0.239141 |
| C | -1.768830 | -3.297598 | -0.314443 |
| O | -2.075960 | -4.442393 | -0.586147 |
| C | 0.646803 | -3.839994 | -0.458783 |
| C | -2.781486 | -2.224736 | -0.046025 |
| C | -4.127899 | -2.550203 | 0.118389 |
| C | -5.027850 | -1.514004 | 0.381783 |
| C | -4.549095 | -0.206256 | 0.470956 |
| C | -3.183249 | 0.040901 | 0.285867 |
| N | -2.325081 | -0.957998 | 0.025995 |
| Cu | -0.025720 | 1.499994 | 0.190731 |
| H | -4.441331 | -3.585871 | 0.042220 |
| H | -6.084277 | -1.727875 | 0.518174 |
| H | -2.747944 | 1.043424 | 0.345841 |
| H | -5.216078 | 0.624671 | 0.679427 |
| H | 0.304728 | -4.834865 | -0.730421 |
| H | 1.495145 | -3.448608 | -1.010967 |
| H | -0.135649 | -4.028847 | 1.595413 |
| Cu | -0.274453 | -0.717028 | -0.262774 |
| B | 1.589648 | 0.179761 | -0.031200 |

| | | | |
|---|---|---|---|
| O | 2.280417 | -0.436075 | 1.023645 |
| O | 2.484026 | 0.567451 | -1.023765 |
| C | 3.704136 | -0.187205 | 0.845447 |
| C | 3.805027 | 0.053454 | -0.702219 |
| H | 1.049097 | -2.618361 | 1.371589 |
| C | 4.039575 | 1.059413 | 1.675665 |
| H | 3.753402 | 0.878628 | 2.716726 |
| H | 5.110286 | 1.290389 | 1.642742 |
| H | 3.478823 | 1.929283 | 1.319797 |
| C | 4.849854 | 1.081242 | -1.129760 |
| H | 5.854075 | 0.764303 | -0.822247 |
| H | 4.841009 | 1.183900 | -2.220032 |
| H | 4.643837 | 2.063593 | -0.698923 |
| C | 4.483258 | -1.392206 | 1.366309 |
| H | 5.555929 | -1.274257 | 1.170398 |
| H | 4.342412 | -1.482611 | 2.448825 |
| H | 4.145887 | -2.322966 | 0.902623 |
| C | 3.981679 | -1.241532 | -1.507563 |
| H | 3.830355 | -1.021906 | -2.569127 |
| H | 4.982546 | -1.668196 | -1.376820 |
| H | 3.240345 | -1.989249 | -1.208319 |
| O | -1.377936 | 2.703727 | 0.563543 |
| C | -1.599636 | 3.903380 | -0.135814 |
| C | -0.373303 | 4.829474 | -0.002876 |
| H | -0.164754 | 5.014655 | 1.057344 |
| H | 0.505805 | 4.337162 | -0.440176 |
| H | -0.520045 | 5.793615 | -0.509049 |
| C | -2.833765 | 4.586395 | 0.485819 |
| H | -3.068465 | 5.540883 | -0.004237 |
| H | -3.707421 | 3.927817 | 0.397868 |
| H | -2.655990 | 4.771290 | 1.551591 |
| C | -1.864566 | 3.611487 | -1.628838 |
| H | -0.992668 | 3.107432 | -2.065893 |
| H | -2.726587 | 2.939782 | -1.730053 |
| H | -2.065586 | 4.525413 | -2.204715 |

Electronic Energy = -1533.57718343 (Hartree/Particle)
Dipole Moment (Debye): 6.6515
index 0
Harmonic frequencies
Number of imaginary frequencies= 0

THERMOCHEMICAL DATA

Temperature 298.150 Kelvin. Pressure 1.00000 Atm.
Zero-point correction= 0.459879 (Hartree/Particle)
Thermal correction to Energy= 0.490966
Thermal correction to Enthalpy= 0.491910
Thermal correction to Gibbs Free Energy= 0.394036
Sum of electronic and zero-point Energies= -1533.117305
Sum of electronic and thermal Energies= -1533.086217
Sum of electronic and thermal Enthalpies= -1533.085273
Sum of electronic and thermal Free Energies= -1533.183147
PCM-SP (solvent=THF;6-311+g(2d,p)) = -1533.91623820
Corrected Free Energy = -1533.52220177

-----------------------------------------------------------------

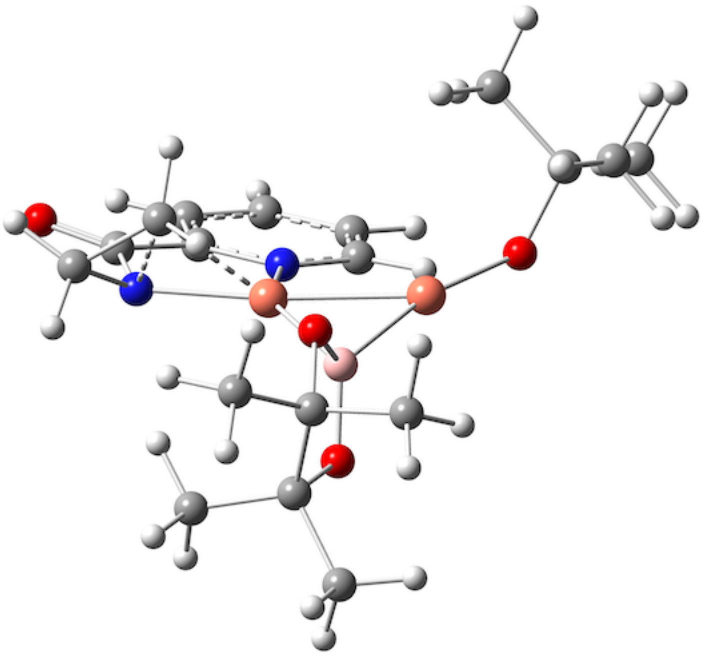

TS3 (Ref 33) Substrate: N-(2picolinoyl)-aziridine
Alkoxide = t-Butoxide

CARTESIAN COORDINATES

| | | | |
|---|---|---|---|
| C | 0.487855 | -1.951451 | 1.933296 |
| N | -0.351896 | -2.729425 | 0.402550 |
| C | -1.685449 | -3.159464 | 0.387427 |
| O | -2.047379 | -4.244651 | 0.827376 |
| C | 0.524325 | -3.333965 | 1.391324 |
| C | -2.647156 | -2.205393 | -0.269065 |
| C | -3.966053 | -2.579974 | -0.518254 |
| C | -4.824078 | -1.631280 | -1.081769 |
| C | -4.340174 | -0.349242 | -1.345301 |
| C | -3.006440 | -0.046629 | -1.047476 |
| N | -2.181252 | -0.972349 | -0.537698 |
| Cu | -0.024474 | 1.471402 | -0.205755 |
| H | -4.292244 | -3.582086 | -0.261541 |
| H | -5.857331 | -1.888041 | -1.298913 |
| H | -2.584131 | 0.951871 | -1.173208 |
| H | -4.979589 | 0.419055 | -1.769069 |
| H | 0.057440 | -4.121926 | 1.984363 |
| H | 1.480062 | -3.648605 | 0.969188 |
| H | -0.308841 | -1.708299 | 2.629854 |
| Cu | -0.138033 | -0.823954 | 0.060066 |
| B | 1.678003 | 0.381806 | -0.200974 |
| O | 2.559451 | 0.341090 | 0.881421 |
| O | 2.356301 | 0.140699 | -1.388398 |
| C | 3.921401 | 0.318967 | 0.361304 |
| C | 3.711255 | -0.286892 | -1.070446 |
| H | 1.353476 | -1.294194 | 1.899211 |
| C | 4.659862 | 0.241304 | -2.142334 |
| H | 5.700706 | 0.009961 | -1.884766 |
| H | 4.430803 | -0.231570 | -3.103037 |
| H | 4.560357 | 1.321836 | -2.267606 |
| C | 3.696375 | -1.821969 | -1.083213 |
| H | 3.340045 | -2.164421 | -2.059692 |
| H | 4.694036 | -2.239347 | -0.907643 |

| | | | |
|---|---|---|---|
| H | 3.012304 | -2.212000 | -0.322121 |
| C | 4.409932 | 1.772955 | 0.339726 |
| H | 4.332425 | 2.187588 | 1.349863 |
| H | 5.453681 | 1.841676 | 0.013641 |
| H | 3.791822 | 2.384867 | -0.324583 |
| C | 4.788503 | -0.517614 | 1.297794 |
| H | 5.800429 | -0.629288 | 0.890159 |
| H | 4.865110 | -0.022297 | 2.271557 |
| H | 4.367230 | -1.513750 | 1.457326 |
| O | -1.401342 | 2.652335 | -0.491361 |
| C | -1.970754 | 3.477877 | 0.495901 |
| C | -3.107925 | 4.281331 | -0.163807 |
| H | -3.600418 | 4.958351 | 0.546885 |
| H | -2.708080 | 4.873003 | -0.995236 |
| H | -3.862161 | 3.595388 | -0.569680 |
| C | -2.542177 | 2.617193 | 1.644662 |
| H | -3.289792 | 1.916689 | 1.252324 |
| H | -1.733869 | 2.027727 | 2.097891 |
| H | -3.012944 | 3.226262 | 2.428305 |
| C | -0.908282 | 4.444351 | 1.057049 |
| H | -0.096109 | 3.868051 | 1.520319 |
| H | -0.479264 | 5.035145 | 0.239488 |
| H | -1.322831 | 5.129329 | 1.809366 |

Electronic Energy = -1533.54822187 (Hartree/Particle)
Dipole Moment (Debye): 4.4280
index 0
Harmonic frequencies
Number of imaginary frequencies= 1
Negatives Eigenvalues: -253.913

THERMOCHEMICAL DATA

Temperature 298.150 Kelvin. Pressure 1.00000 Atm.
Zero-point correction= 0.458451
(Hartree/Particle)
Thermal correction to Energy= 0.488885
Thermal correction to Enthalpy= 0.489829
Thermal correction to Gibbs Free Energy= 0.395081
Sum of electronic and zero-point Energies= -1533.089770
Sum of electronic and thermal Energies= -1533.059337
Sum of electronic and thermal Enthalpies= -1533.058393
Sum of electronic and thermal Free Energies= -1533.153141
PCM-SP (solvent=THF;6-311+g(2d,p)) = -1533.88806121
Corrected Free Energy = -1533.49298034

---

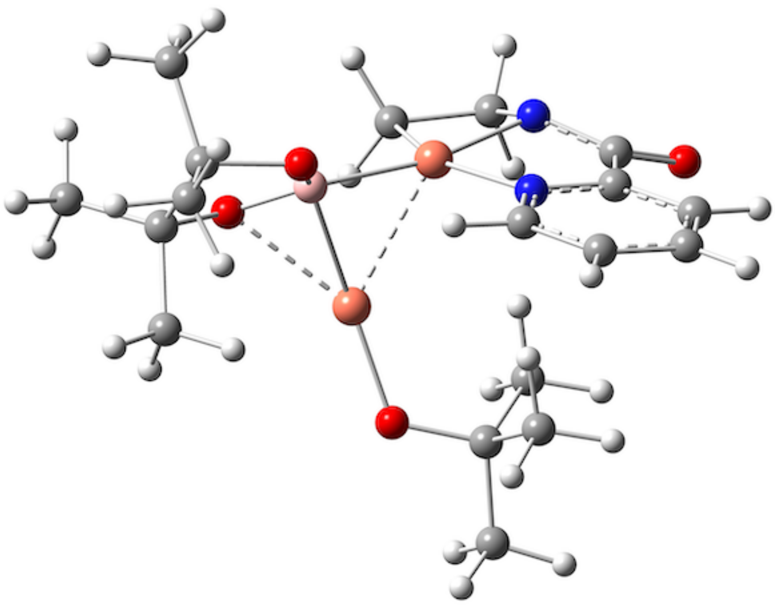

TS4 (Ref 33) Substrate: N-(2picolinoyl)-aziridine
Alkoxide = t-Butoxide

CARTESIAN COORDINATES

| | | | |
|---|---|---|---|
| N | -1.293969 | -1.581325 | 0.622329 |
| C | -2.607927 | -1.672634 | 0.315693 |
| C | -3.557067 | -1.998636 | 1.278848 |
| C | -3.127430 | -2.257569 | 2.582000 |
| C | -1.765214 | -2.175073 | 2.883662 |
| C | -0.873322 | -1.822130 | 1.871510 |
| C | -2.989372 | -1.321918 | -1.121688 |
| O | -4.161483 | -1.115969 | -1.443492 |
| Cu | -0.299703 | -0.853978 | -0.988754 |
| N | -1.881981 | -1.275899 | -1.887888 |
| C | -1.675746 | -0.519465 | -3.118809 |
| C | -0.199812 | -0.173063 | -2.867060 |
| B | 1.581539 | -0.467829 | -0.359054 |
| Cu | 0.488290 | 1.408248 | -0.193404 |
| O | 2.467606 | 0.493828 | -0.831458 |
| C | 3.677733 | 0.435851 | 0.019541 |
| C | 4.881532 | 0.765252 | -0.853993 |
| O | 2.172151 | -1.319144 | 0.543145 |
| C | 3.615676 | -1.041958 | 0.547372 |
| C | 4.256620 | -2.058998 | -0.402785 |
| C | 3.493792 | 1.491666 | 1.112419 |
| C | 4.134097 | -1.233167 | 1.968348 |
| H | -4.601669 | -2.026160 | 0.986581 |
| H | -3.846006 | -2.513370 | 3.355899 |
| H | 0.194004 | -1.717792 | 2.037697 |
| H | -1.395828 | -2.365112 | 3.886357 |
| H | -1.845245 | -1.115851 | -4.025293 |
| H | 0.537122 | -0.798390 | -3.373537 |
| H | 0.077337 | 0.880660 | -2.874199 |
| H | 4.380661 | 1.558813 | 1.751177 |
| H | 3.325563 | 2.466002 | 0.643543 |
| H | 2.622087 | 1.273898 | 1.737964 |
| H | 4.823080 | 1.807334 | -1.183570 |
| H | 5.809925 | 0.637699 | -0.284739 |
| H | 4.924366 | 0.130201 | -1.741611 |

| | | | |
|---|---|---|---|
| H | 4.040984 | -2.285467 | 2.255887 |
| H | 5.192566 | -0.953906 | 2.030286 |
| H | 3.571758 | -0.632499 | 2.686837 |
| H | 5.347495 | -1.961307 | -0.414566 |
| H | 4.001767 | -3.068677 | -0.066173 |
| H | 3.882923 | -1.937970 | -1.424547 |
| H | -2.319328 | 0.367975 | -3.162154 |
| O | -0.400853 | 2.797304 | 0.564166 |
| C | -1.802012 | 2.805041 | 0.731651 |
| C | -2.518032 | 2.370780 | -0.562501 |
| H | -2.202925 | 3.014454 | -1.392370 |
| H | -2.244451 | 1.337606 | -0.812744 |
| H | -3.610959 | 2.414939 | -0.474656 |
| C | -2.195731 | 1.858263 | 1.883321 |
| H | -1.683659 | 2.161477 | 2.803999 |
| H | -3.278937 | 1.851029 | 2.064493 |
| H | -1.877368 | 0.838714 | 1.639234 |
| C | -2.201196 | 4.250410 | 1.084639 |
| H | -3.282026 | 4.343307 | 1.254225 |
| H | -1.673574 | 4.569249 | 1.990926 |
| H | -1.913630 | 4.924959 | 0.269914 |

Electronic Energy = -1533.55523236 (Hartree/Particle)
Dipole Moment (Debye): 8.6247
index 0
Harmonic frequencies
Number of imaginary frequencies= 1
Negatives Eigenvalues: -85.071

THERMOCHEMICAL DATA

Temperature 298.150 Kelvin. Pressure 1.00000 Atm.
Zero-point correction= 0.459589
(Hartree/Particle)
Thermal correction to Energy= 0.489407
Thermal correction to Enthalpy= 0.490351
Thermal correction to Gibbs Free Energy= 0.398846
Sum of electronic and zero-point Energies= -1533.095644
Sum of electronic and thermal Energies= -1533.065826
Sum of electronic and thermal Enthalpies= -1533.064881
Sum of electronic and thermal Free Energies= -1533.156387
PCM-SP (solvent=THF;6-311+g(2d,p)) = -1533.89881221
Corrected Free Energy = -1533.49996685

---

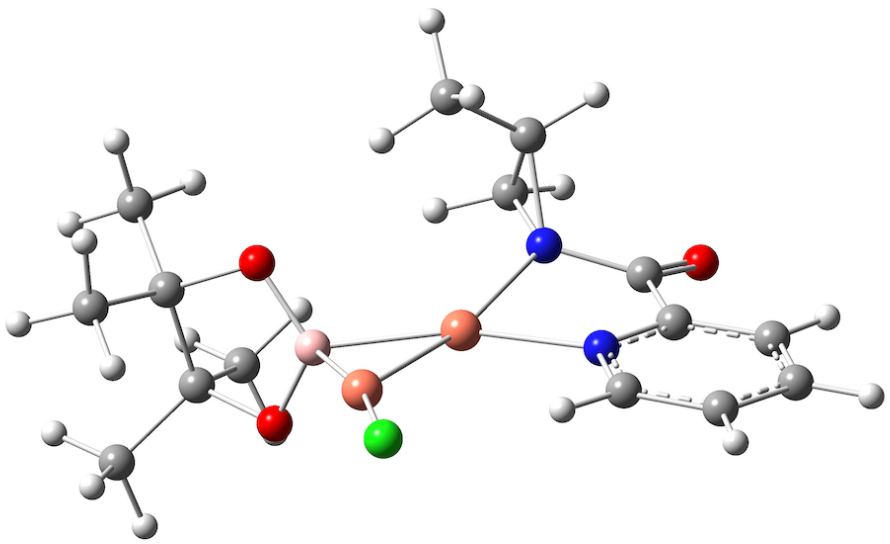

SUBSTRATE: N-(2-PICOLINOYL)-METHYL AZIRIDINE

Complex-2 Substrate: N-(2-picolinoyl)-methyl aziridine

CARTESIAN COORDINATES

| | | | |
|---|---|---|---|
| C | -1.087225 | -2.709815 | 1.493532 |
| N | -1.279327 | -2.050241 | 0.135376 |
| C | -2.613626 | -2.128474 | -0.378018 |
| O | -3.132381 | -3.156041 | -0.771487 |
| C | -0.537778 | -3.320086 | 0.260848 |
| C | -3.319844 | -0.805188 | -0.389961 |
| C | -4.693401 | -0.736458 | -0.621802 |
| C | -5.302288 | 0.521071 | -0.609400 |
| C | -4.518751 | 1.651552 | -0.372655 |
| C | -3.144100 | 1.497504 | -0.162898 |
| N | -2.562083 | 0.289555 | -0.172630 |
| Cu | 0.396799 | 2.030615 | 0.177744 |
| H | -5.250379 | -1.649475 | -0.802870 |
| H | -6.370685 | 0.614489 | -0.782370 |
| H | -2.482336 | 2.344121 | 0.011588 |
| H | -4.952717 | 2.646350 | -0.355643 |
| H | -1.023495 | -4.199195 | -0.154152 |
| H | 0.522277 | -3.205389 | 0.054132 |
| C | -0.255933 | -1.977807 | 2.513419 |
| H | 0.024414 | -2.672803 | 3.315050 |
| H | 0.654049 | -1.562353 | 2.071241 |
| Cu | -0.513246 | -0.063033 | -0.073272 |
| B | 1.521662 | 0.267394 | -0.138902 |
| O | 2.293574 | -0.272330 | 0.892179 |
| O | 2.227188 | 0.242703 | -1.335895 |
| C | 3.664502 | -0.402379 | 0.415643 |
| C | 3.463910 | -0.498260 | -1.138041 |
| Cl | -0.381468 | 4.015674 | 0.513368 |
| C | 4.403737 | 0.868607 | 0.855018 |
| H | 4.324501 | 0.965835 | 1.942413 |
| H | 5.464702 | 0.831791 | 0.583773 |
| H | 3.954620 | 1.759573 | 0.405162 |
| C | 4.294498 | -1.631495 | 1.065165 |
| H | 5.300406 | -1.807330 | 0.665108 |

| | | | |
|---|---|---|---|
| H | 4.379330 | -1.474591 | 2.145744 |
| H | 3.692664 | -2.529061 | 0.900577 |
| C | 4.564045 | 0.152230 | -1.972613 |
| H | 5.531460 | -0.326840 | -1.777921 |
| H | 4.333817 | 0.040952 | -3.037399 |
| H | 4.650475 | 1.219635 | -1.757810 |
| C | 3.203397 | -1.928218 | -1.631035 |
| H | 2.867455 | -1.888542 | -2.672015 |
| H | 4.106630 | -2.546238 | -1.577730 |
| H | 2.415657 | -2.407387 | -1.040726 |
| H | -2.005618 | -3.167583 | 1.861582 |
| H | -0.827635 | -1.155798 | 2.959016 |

Electronic Energy = -1800.05719595 (Hartree/Particle)
Dipole Moment (Debye): 8.7936
index 0
Harmonic frequencies
Number of imaginary frequencies= 0

THERMOCHEMICAL DATA

Temperature 298.150 Kelvin. Pressure 1.00000 Atm.
Zero-point correction= 0.363783
(Hartree/Particle)
Thermal correction to Energy= 0.390722
Thermal correction to Enthalpy= 0.391666
Thermal correction to Gibbs Free Energy= 0.303896
Sum of electronic and zero-point Energies= -1799.693413
Sum of electronic and thermal Energies= -1799.666474
Sum of electronic and thermal Enthalpies= -1799.665530
Sum of electronic and thermal Free Energies= -1799.753300
PCM-SP (solvent=THF;6-311+g(2d,p)) = -1800.37640687
Corrected Free Energy = -1800.07251092

-------------------------------------------------------

PATH: ANTI_MARKOVNIKOV

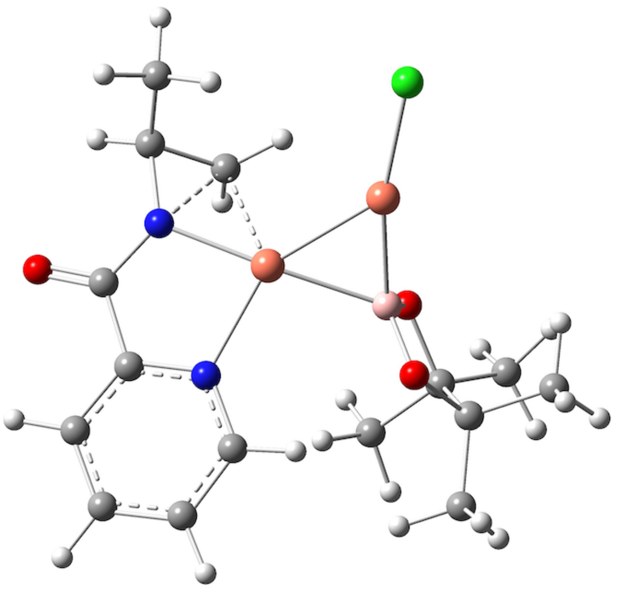

TS3 Substrate: N-(2-picolinoyl)-methyl aziridine

Path: anti-Markovnikov

CARTESIAN COORDINATES

| | | | |
|---|---|---|---|
| C | -1.758973 | -1.317001 | 1.715715 |
| N | -2.649497 | -0.334468 | 0.327132 |
| C | -3.153594 | 0.954571 | 0.487109 |
| O | -4.191008 | 1.231398 | 1.079642 |
| C | -3.152548 | -1.386208 | 1.205525 |
| C | -2.346226 | 2.015010 | -0.223906 |
| C | -2.830827 | 3.314046 | -0.368579 |
| C | -2.036131 | 4.244547 | -1.042759 |
| C | -0.788872 | 3.848972 | -1.531938 |
| C | -0.368842 | 2.532724 | -1.322117 |
| N | -1.139624 | 1.642352 | -0.686756 |
| Cu | 0.600545 | -2.181951 | -0.629164 |
| H | -3.801752 | 3.566093 | 0.044125 |
| H | -2.385421 | 5.263661 | -1.183176 |
| H | 0.597017 | 2.161612 | -1.652390 |
| H | -0.146865 | 4.542602 | -2.066065 |
| H | -3.920605 | -1.023555 | 1.892746 |
| H | -1.516336 | -0.601406 | 2.495363 |
| Cu | -0.747683 | -0.349472 | -0.111093 |
| B | 1.372404 | -0.365828 | -0.026433 |
| O | 2.104313 | 0.424744 | -0.905892 |
| O | 1.844853 | -0.228674 | 1.272567 |
| C | 3.290905 | 0.903979 | -0.202147 |
| C | 2.829088 | 0.847026 | 1.296519 |
| H | -1.062002 | -2.132270 | 1.539001 |
| C | -3.541792 | -2.659879 | 0.474734 |
| H | -3.735072 | -3.461498 | 1.196856 |
| H | -4.449437 | -2.493588 | -0.114733 |
| Cl | 0.248859 | -4.251017 | -1.059405 |
| C | 3.924315 | 0.488319 | 2.296263 |
| H | 4.727281 | 1.235083 | 2.272900 |
| H | 3.507526 | 0.465246 | 3.308623 |
| H | 4.352208 | -0.494374 | 2.086273 |
| C | 2.082781 | 2.108180 | 1.753144 |
| H | 1.622853 | 1.913195 | 2.727357 |
| H | 2.759530 | 2.963789 | 1.854500 |
| H | 1.285728 | 2.370920 | 1.050305 |
| C | 4.421664 | -0.079405 | -0.529072 |
| H | 4.544516 | -0.129161 | -1.615503 |
| H | 5.371298 | 0.237798 | -0.084288 |
| H | 4.183331 | -1.085997 | -0.171748 |
| C | 3.636302 | 2.297932 | -0.718839 |
| H | 4.482800 | 2.716169 | -0.161251 |
| H | 3.918916 | 2.239612 | -1.775275 |
| H | 2.791511 | 2.985851 | -0.629506 |
| H | -2.737564 | -2.983890 | -0.195698 |

Electronic Energy = -1800.02257319 (Hartree/Particle)
Dipole Moment (Debye): 11.1955
index 0
Harmonic frequencies
Number of imaginary frequencies= 1
Negatives Eigenvalues: -267.288

THERMOCHEMICAL DATA

Temperature 298.150 Kelvin. Pressure 1.00000 Atm.
Zero-point correction= 0.362067
(Hartree/Particle)
Thermal correction to Energy= 0.388523
Thermal correction to Enthalpy= 0.389467
Thermal correction to Gibbs Free Energy= 0.302924
Sum of electronic and zero-point Energies= -1799.660506
Sum of electronic and thermal Energies= -1799.634050
Sum of electronic and thermal Enthalpies= -1799.633106
Sum of electronic and thermal Free Energies= -1799.719649
PCM-SP (solvent=THF;6-311+g(2d,p)) = -1800.34339657
Corrected Free Energy = -1800.04047238

---

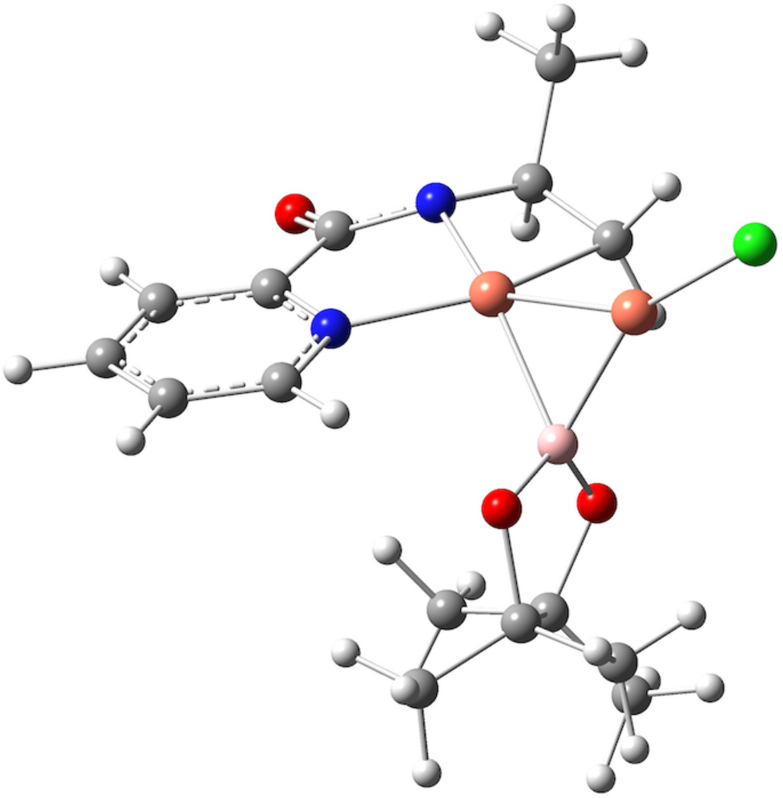

I2 Substrate: N-(2-picolinoyl)-methyl aziridine
Path: anti-Markovnikov

CARTESIAN COORDINATES

| | | | |
|---|---|---|---|
| C | 0.873396 | 2.141956 | 1.320868 |
| N | 2.503962 | 0.880601 | 0.506059 |
| C | 3.211326 | -0.272038 | 0.562793 |
| O | 4.158559 | -0.541918 | 1.302724 |
| C | 2.357484 | 1.856565 | 1.586552 |
| C | 2.688991 | -1.269389 | -0.462527 |
| C | 3.414094 | -2.393933 | -0.842724 |
| C | 2.853658 | -3.263868 | -1.780961 |
| C | 1.582560 | -2.989954 | -2.292789 |
| C | 0.902978 | -1.857436 | -1.842334 |
| N | 1.453252 | -1.020083 | -0.951015 |
| Cu | -1.174951 | 1.873924 | -0.767374 |

```
 H                4.388790   -2.559462   -0.395702
 H                3.399357   -4.145044   -2.107059
 H               -0.101567   -1.610240   -2.166588
 H                1.114830   -3.641885   -3.023688
 H                2.506464    1.374986    2.562610
 H                0.145721    1.837976    2.072339
 Cu               0.739945    0.681587   -0.063080
 B               -1.313175    0.044332    0.100537
 O               -1.852197   -0.947869   -0.690732
 O               -1.560950   -0.159024    1.441506
 C               -2.735210   -1.754466    0.158140
 C               -2.129582   -1.501785    1.584546
 H                0.644507    3.097970    0.847345
 C                3.281183    3.060898    1.450625
 H                3.059204    3.803447    2.227450
 H                4.325981    2.748559    1.552801
 Cl              -2.105846    3.580840   -1.633514
 C               -3.142920   -1.484113    2.722833
 H               -3.651705   -2.452592    2.797253
 H               -2.630365   -1.293126    3.671233
 H               -3.893178   -0.703267    2.580316
 C               -0.953257   -2.427644    1.919089
 H               -0.456844   -2.059399    2.822320
 H               -1.289443   -3.453919    2.101315
 H               -0.216622   -2.438020    1.109034
 C               -4.147333   -1.186138   -0.015926
 H               -4.413398   -1.214202   -1.076996
 H               -4.884061   -1.771729    0.544551
 H               -4.198369   -0.144337    0.315233
 C               -2.672138   -3.200555   -0.321854
 H               -3.249527   -3.851192    0.345457
 H               -3.102083   -3.274677   -1.326171
 H               -1.643757   -3.568144   -0.362765
 H                3.151301    3.530825    0.468648

 Electronic Energy =  -1800.03669862 (Hartree/Particle)
 Dipole Moment (Debye):      9.5825
 index           0
 Harmonic frequencies
 Number of imaginary frequencies=           0

 THERMOCHEMICAL DATA

 Temperature   298.150 Kelvin.  Pressure   1.00000 Atm.
 Zero-point correction=                          0.363337
(Hartree/Particle)
 Thermal correction to Energy=                   0.389975
 Thermal correction to Enthalpy=                 0.390919
 Thermal correction to Gibbs Free Energy=        0.304038
 Sum of electronic and zero-point Energies=         -1799.673362
 Sum of electronic and thermal Energies=            -1799.646724
 Sum of electronic and thermal Enthalpies=          -1799.645780
 Sum of electronic and thermal Free Energies=       -1799.732660
 PCM-SP (solvent=THF;6-311+g(2d,p)) =  -1800.35758090
 Corrected Free Energy =  -1800.05354228
```

------------------------------------------------------

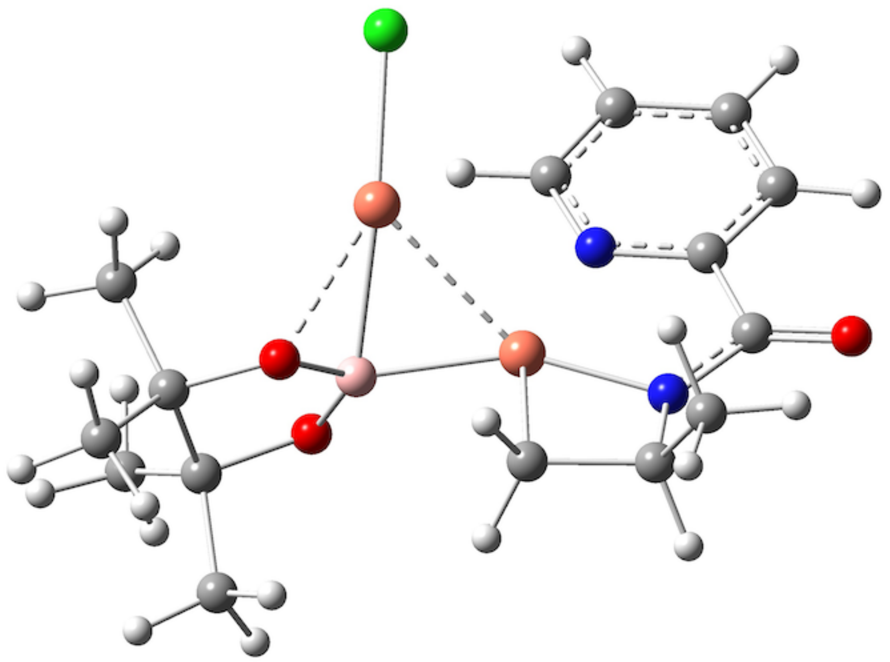

TS4 Substrate: N-(2-picolinoyl)-methyl aziridine
Path: anti-Markovnikov

CARTESIAN COORDINATES

| | | | |
|---|---|---|---|
| N | 1.970245 | -0.690627 | 0.757446 |
| C | 3.221220 | -0.172112 | 0.717847 |
| C | 4.337076 | -0.935838 | 1.038944 |
| C | 4.154277 | -2.268784 | 1.416483 |
| C | 2.862737 | -2.796199 | 1.450887 |
| C | 1.791264 | -1.972593 | 1.105352 |
| C | 3.347982 | 1.279674 | 0.261586 |
| O | 4.447550 | 1.804202 | 0.063215 |
| Cu | 0.621791 | 0.725407 | 0.162524 |
| N | 2.123101 | 1.813776 | 0.142765 |
| C | 1.613239 | 3.005727 | -0.517792 |
| C | 0.144565 | 2.524862 | -0.562388 |
| B | -1.358847 | 0.327620 | 0.279536 |
| Cu | -0.601814 | -1.075698 | -1.177419 |
| Cl | 0.119440 | -2.953817 | -1.870786 |
| O | -2.295520 | 0.350218 | -0.752997 |
| C | -3.609243 | 0.012432 | -0.158351 |
| C | -4.679926 | 0.784440 | -0.918548 |
| O | -1.946657 | 0.262555 | 1.513287 |
| C | -3.393211 | 0.453604 | 1.332335 |
| C | -3.667953 | 1.941630 | 1.574121 |
| C | -3.795015 | -1.496145 | -0.336152 |
| C | -4.120435 | -0.402589 | 2.362643 |
| H | 5.315031 | -0.470157 | 0.974044 |
| H | 5.009145 | -2.889820 | 1.669877 |
| H | 0.772182 | -2.339329 | 1.071331 |
| H | 2.677714 | -3.831161 | 1.718793 |
| H | 1.731166 | 3.895943 | 0.117789 |
| H | -0.547901 | 3.017608 | 0.122142 |
| H | -0.287461 | 2.371651 | -1.552452 |
| H | -4.776889 | -1.819547 | 0.024479 |
| H | -3.713647 | -1.744285 | -1.398796 |

| | | | |
|---|---|---|---|
| H | -3.020837 | -2.059392 | 0.194719 |
| H | -4.740488 | 0.415515 | -1.947266 |
| H | -5.658465 | 0.641009 | -0.445263 |
| H | -4.461037 | 1.853983 | -0.953444 |
| H | -3.905593 | -0.029428 | 3.369006 |
| H | -5.204137 | -0.355019 | 2.202615 |
| H | -3.803851 | -1.446759 | 2.313880 |
| H | -4.739055 | 2.163839 | 1.520290 |
| H | -3.306423 | 2.211264 | 2.571172 |
| H | -3.145337 | 2.566412 | 0.842338 |
| C | 2.222363 | 3.272231 | -1.893408 |
| H | 1.738998 | 4.137603 | -2.363829 |
| H | 3.294824 | 3.467284 | -1.802086 |
| H | 2.086435 | 2.398124 | -2.542600 |

Electronic Energy = -1800.02788397 (Hartree/Particle)
Dipole Moment (Debye): 7.1813
index 0
Harmonic frequencies
Number of imaginary frequencies= 1
Negatives Eigenvalues: -81.232

THERMOCHEMICAL DATA

Temperature 298.150 Kelvin. Pressure 1.00000 Atm.
Zero-point correction= 0.362842 (Hartree/Particle)
Thermal correction to Energy= 0.388810
Thermal correction to Enthalpy= 0.389755
Thermal correction to Gibbs Free Energy= 0.304384
Sum of electronic and zero-point Energies= -1799.665041
Sum of electronic and thermal Energies= -1799.639074
Sum of electronic and thermal Enthalpies= -1799.638129
Sum of electronic and thermal Free Energies= -1799.723500
PCM-SP (solvent=THF;6-311+g(2d,p)) = -1800.34679723
Corrected Free Energy = -1800.04241326

---

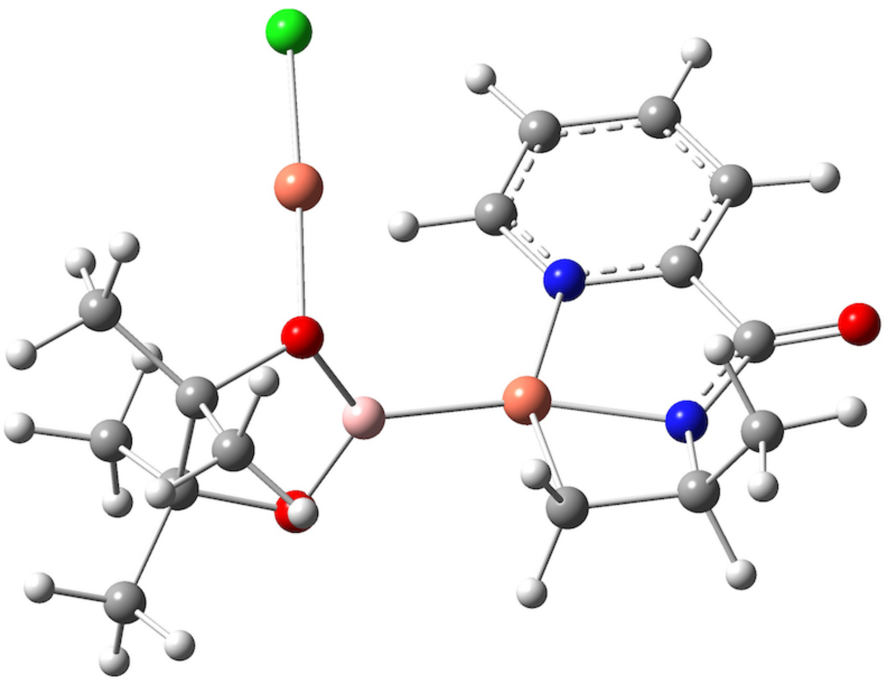

I3 Substrate: N-(2-picolinoyl)-methyl aziridine
Path: anti-Markovnikov

CARTESIAN COORDINATES

| | | | |
|---|---|---|---|
| C | 2.577785 | -3.289328 | 1.822923 |
| C | 2.144467 | -3.060149 | 0.375581 |
| Cu | 0.872765 | -0.973755 | -0.383087 |
| B | -1.080189 | -0.781058 | -0.426512 |
| O | -1.843789 | -0.192454 | 0.622188 |
| C | -3.265187 | -0.620158 | 0.429787 |
| C | -3.288794 | -0.853458 | -1.113989 |
| O | -1.913070 | -1.282121 | -1.383498 |
| Cu | -1.305629 | 1.651651 | 0.884445 |
| Cl | -0.751655 | 3.677840 | 0.988057 |
| C | 0.617349 | -2.850619 | 0.204623 |
| N | 2.543454 | -1.769039 | -0.176239 |
| C | 3.616938 | -0.992021 | 0.019846 |
| C | 3.280173 | 0.438958 | -0.405913 |
| C | 4.253994 | 1.428646 | -0.466844 |
| C | 3.878326 | 2.727181 | -0.822559 |
| C | 2.538202 | 2.995672 | -1.101177 |
| C | 1.614798 | 1.953761 | -1.016240 |
| N | 1.977021 | 0.707768 | -0.678533 |
| O | 4.735487 | -1.282325 | 0.458610 |
| H | 5.275405 | 1.157505 | -0.220519 |
| H | 4.619983 | 3.519934 | -0.870371 |
| H | 0.558886 | 2.121672 | -1.190972 |
| H | 2.193520 | 3.992323 | -1.354832 |
| H | 2.507641 | -3.888020 | -0.251805 |
| H | 0.139210 | -3.410769 | -0.600822 |
| H | 0.035844 | -2.885408 | 1.128927 |
| H | 2.186821 | -4.246313 | 2.190973 |
| H | 3.668781 | -3.293284 | 1.899003 |
| C | -4.194221 | 0.467161 | 0.947799 |
| H | -5.236361 | 0.180462 | 0.765552 |
| H | -4.057970 | 0.593441 | 2.026553 |
| H | -4.014092 | 1.432357 | 0.467984 |
| C | -3.411159 | -1.901301 | 1.252476 |
| H | -3.157327 | -1.688548 | 2.295382 |
| H | -4.439823 | -2.273886 | 1.214428 |
| H | -2.742432 | -2.687896 | 0.889500 |
| C | -3.518641 | 0.432231 | -1.915638 |
| H | -3.301470 | 0.234497 | -2.969518 |
| H | -4.555348 | 0.774864 | -1.831900 |
| H | -2.857472 | 1.237669 | -1.576888 |
| C | -4.234013 | -1.957518 | -1.573435 |
| H | -5.267354 | -1.717875 | -1.296186 |
| H | -4.184854 | -2.052125 | -2.662649 |
| H | -3.966784 | -2.923114 | -1.137946 |
| H | 2.194876 | -2.484718 | 2.463875 |

Electronic Energy = -1800.03753226 (Hartree/Particle)
Dipole Moment (Debye): 7.2974
index 0
Harmonic frequencies
Number of imaginary frequencies= 0

THERMOCHEMICAL DATA

Temperature 298.150 Kelvin. Pressure 1.00000 Atm.
Zero-point correction= 0.363855
(Hartree/Particle)
Thermal correction to Energy= 0.390129
Thermal correction to Enthalpy= 0.391074
Thermal correction to Gibbs Free Energy= 0.305216
Sum of electronic and zero-point Energies= -1799.673677
Sum of electronic and thermal Energies= -1799.647403
Sum of electronic and thermal Enthalpies= -1799.646459
Sum of electronic and thermal Free Energies= -1799.732316
PCM-SP (solvent=THF;6-311+g(2d,p)) = -1800.35648856
Corrected Free Energy = -1800.05127230

-----------------------------------------------------------------------

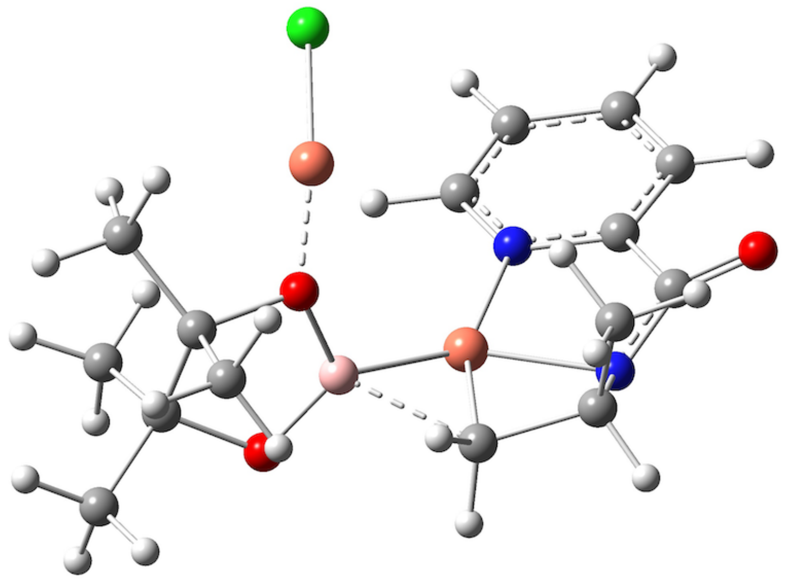

TS5 Substrate: N-(2-picolinoyl)-methyl aziridine
Path: anti-Markovnikov

CARTESIAN COORDINATES

| | | | |
|---|---|---|---|
| C | 0.977814 | -3.035306 | 0.386676 |
| Cu | 0.653820 | -0.855318 | -0.747011 |
| B | -1.286738 | -0.749970 | -0.506433 |
| O | -1.802250 | -0.127463 | 0.665187 |
| C | -3.288971 | -0.066826 | 0.507551 |
| C | -3.420860 | -0.072614 | -1.053041 |
| O | -2.257746 | -0.861486 | -1.463431 |
| Cu | -0.681246 | 1.449849 | 0.927619 |
| Cl | 0.371123 | 3.262312 | 1.105575 |
| C | -0.348440 | -2.526551 | -0.285329 |
| N | 1.968500 | -2.181067 | -0.279532 |
| C | 3.069740 | -1.642627 | 0.258913 |
| C | 3.289402 | -0.229136 | -0.309612 |
| C | 4.522536 | 0.410034 | -0.225071 |
| C | 4.642438 | 1.722945 | -0.684332 |
| C | 3.516058 | 2.373693 | -1.190994 |
| C | 2.304596 | 1.686957 | -1.221857 |
| N | 2.197492 | 0.415344 | -0.805581 |
| O | 3.848496 | -2.072126 | 1.122618 |

| | | | |
|---|---|---|---|
| H | 5.351309 | -0.134954 | 0.214322 |
| H | 5.598068 | 2.237938 | -0.632961 |
| H | 1.388445 | 2.162224 | -1.550679 |
| H | 3.557032 | 3.403773 | -1.529134 |
| H | 1.106741 | -4.100759 | 0.135878 |
| H | -0.533240 | -3.005131 | -1.251385 |
| H | -1.215104 | -2.681959 | 0.364310 |
| C | -3.814148 | 1.176594 | 1.208451 |
| H | -4.895564 | 1.260347 | 1.051486 |
| H | -3.628389 | 1.105623 | 2.284746 |
| H | -3.340755 | 2.091076 | 0.842386 |
| C | -3.819695 | -1.334515 | 1.177730 |
| H | -3.479866 | -1.360611 | 2.217456 |
| H | -4.914211 | -1.347357 | 1.169666 |
| H | -3.464953 | -2.238973 | 0.674688 |
| C | -3.263762 | 1.317215 | -1.679819 |
| H | -3.157033 | 1.204822 | -2.762808 |
| H | -4.137799 | 1.945262 | -1.479028 |
| H | -2.372670 | 1.830892 | -1.302590 |
| C | -4.675902 | -0.762523 | -1.576328 |
| H | -5.574130 | -0.254357 | -1.206594 |
| H | -4.686089 | -0.723102 | -2.669911 |
| H | -4.715256 | -1.812010 | -1.275630 |
| C | 0.938609 | -2.880857 | 1.910528 |
| H | 0.147965 | -3.508160 | 2.341213 |
| H | 1.900318 | -3.161158 | 2.346744 |
| H | 0.738713 | -1.834512 | 2.177592 |

Electronic Energy = -1800.02970367 (Hartree/Particle)
Dipole Moment (Debye): 8.3826
index 1
Number of imaginary frequencies= 1
Negatives Eigenvalues: -138.424

THERMOCHEMICAL DATA

Temperature 298.150 Kelvin. Pressure 1.00000 Atm.
Zero-point correction= 0.363414
(Hartree/Particle)
Thermal correction to Energy= 0.388806
Thermal correction to Enthalpy= 0.389750
Thermal correction to Gibbs Free Energy= 0.307359
Sum of electronic and zero-point Energies= -1799.666290
Sum of electronic and thermal Energies= -1799.640898
Sum of electronic and thermal Enthalpies= -1799.639953
Sum of electronic and thermal Free Energies= -1799.722347
PCM-SP (solvent=THF;6-311+g(2d,p)) = -1800.35173892
Corrected Free Energy = -1800.04438225

---

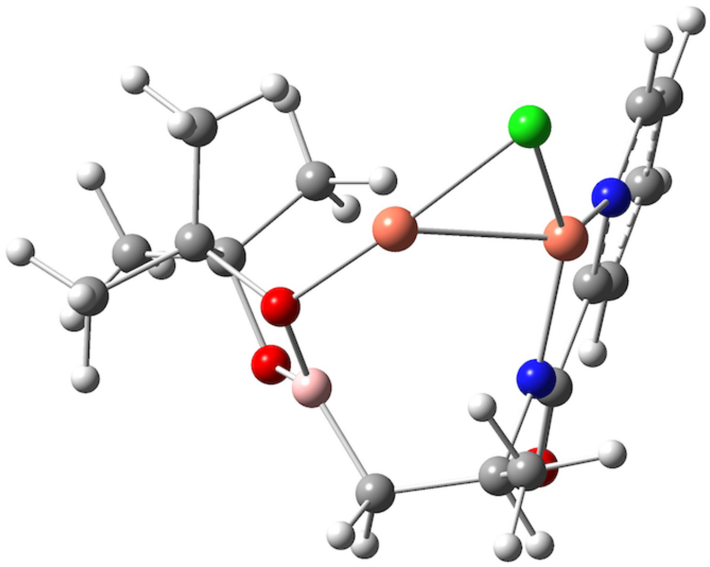

P2 Substrate: N-(2-picolinoyl)-methyl aziridine
Path: anti-Markovnikov

CARTESIAN COORDINATES

| | | | |
|---|---|---|---|
| C | -0.117690 | -0.765279 | 2.675057 |
| Cu | -1.073780 | -1.474893 | -0.163789 |
| B | 1.561516 | 0.717258 | 1.299491 |
| O | 2.256625 | 0.001471 | 0.271957 |
| C | 2.798695 | 1.030777 | -0.668094 |
| C | 1.758161 | 2.178763 | -0.470664 |
| O | 1.394989 | 2.015643 | 0.927497 |
| Cu | 1.356519 | -1.556731 | -0.501793 |
| Cl | 0.028793 | -2.920989 | -1.562432 |
| C | 1.137384 | 0.167765 | 2.703338 |
| N | -0.965938 | -0.480102 | 1.512674 |
| C | -1.765092 | 0.593837 | 1.609327 |
| C | -2.633696 | 0.840852 | 0.379470 |
| C | -3.468647 | 1.960681 | 0.332379 |
| C | -4.246804 | 2.177298 | -0.804622 |
| C | -4.169001 | 1.269863 | -1.865294 |
| C | -3.310166 | 0.177598 | -1.740347 |
| N | -2.563051 | -0.032911 | -0.646993 |
| O | -1.862462 | 1.387158 | 2.566853 |
| H | -3.481700 | 2.625844 | 1.188847 |
| H | -4.905183 | 3.040227 | -0.864782 |
| H | -3.208678 | -0.559162 | -2.533312 |
| H | -4.758201 | 1.399473 | -2.768248 |
| H | -0.698752 | -0.553834 | 3.581822 |
| H | 0.896694 | 1.051640 | 3.304469 |
| H | 1.973886 | -0.349146 | 3.194784 |
| C | 2.876775 | 0.456822 | -2.075851 |
| H | 3.265252 | 1.220234 | -2.759752 |
| H | 3.562235 | -0.397126 | -2.098751 |
| H | 1.904239 | 0.130643 | -2.453777 |
| C | 4.197263 | 1.363198 | -0.144359 |
| H | 4.795323 | 0.447194 | -0.110695 |
| H | 4.699069 | 2.081104 | -0.800880 |

| | | | |
|---|---|---|---|
| H | 4.156195 | 1.784978 | 0.864443 |
| C | 0.476703 | 1.983473 | -1.290345 |
| H | -0.283695 | 2.677899 | -0.921525 |
| H | 0.647496 | 2.181445 | -2.353838 |
| H | 0.078590 | 0.969755 | -1.177212 |
| C | 2.321569 | 3.581874 | -0.667380 |
| H | 2.701372 | 3.704686 | -1.688503 |
| H | 1.528322 | 4.318126 | -0.505240 |
| H | 3.128047 | 3.794151 | 0.038572 |
| C | 0.273565 | -2.243489 | 2.689371 |
| H | 0.803768 | -2.496397 | 3.615611 |
| H | -0.617253 | -2.875645 | 2.605831 |
| H | 0.932082 | -2.495772 | 1.845766 |

Electronic Energy = -1800.12533328 (Hartree/Particle)
Dipole Moment (Debye): 6.8363
index 0
Harmonic frequencies
Number of imaginary frequencies= 0

THERMOCHEMICAL DATA

Temperature 298.150 Kelvin. Pressure 1.00000 Atm.
Zero-point correction= 0.365152
(Hartree/Particle)
Thermal correction to Energy= 0.391099
Thermal correction to Enthalpy= 0.392044
Thermal correction to Gibbs Free Energy= 0.308863
Sum of electronic and zero-point Energies= -1799.760182
Sum of electronic and thermal Energies= -1799.734234
Sum of electronic and thermal Enthalpies= -1799.733290
Sum of electronic and thermal Free Energies= -1799.816471
PCM-SP (solvent=THF;6-311+g(2d,p)) = -1800.44170581
Corrected Free Energy = -1800.13284353

------------------------------------------------------

PATH: MARKOVNIKOV

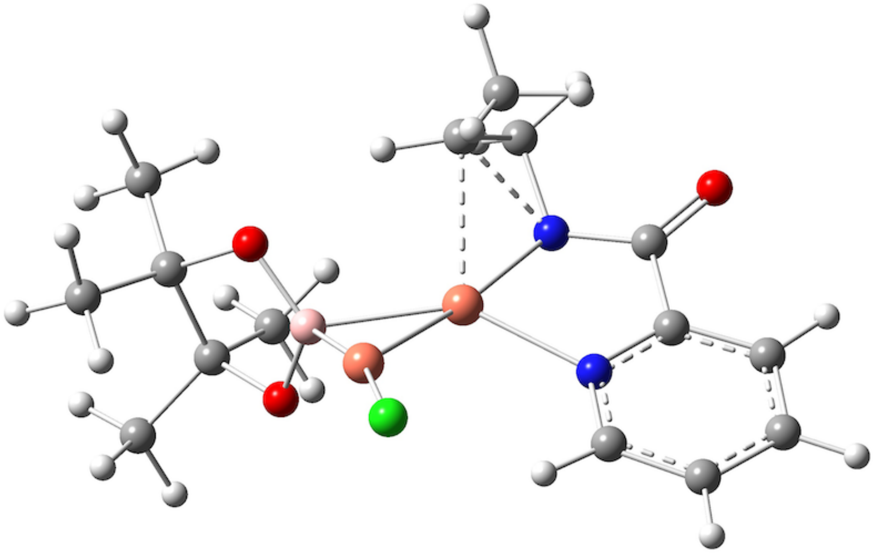

TS3 Substrate: N-(2-picolinoyl)-methyl aziridine
Path: Markovnikov

CARTESIAN COORDINATES

| | | | |
|---|---|---|---|
| C | -0.160621 | -1.906567 | 1.640076 |
| N | -1.190145 | -2.092050 | -0.028784 |
| C | -2.577668 | -2.125931 | -0.094738 |
| O | -3.258891 | -3.112122 | 0.175901 |
| C | -0.546306 | -3.084798 | 0.818246 |
| C | -3.211070 | -0.842051 | -0.568326 |
| C | -4.567554 | -0.784256 | -0.881687 |
| C | -5.107446 | 0.449584 | -1.254738 |
| C | -4.282346 | 1.575204 | -1.274700 |
| C | -2.934807 | 1.433101 | -0.930046 |
| N | -2.415826 | 0.242906 | -0.601988 |
| Cu | 0.283401 | 1.875339 | 0.478912 |
| H | -5.165402 | -1.686890 | -0.816288 |
| H | -6.159749 | 0.531835 | -1.512392 |
| H | -2.254992 | 2.279508 | -0.884483 |
| H | -4.666914 | 2.554283 | -1.542212 |
| H | -1.240306 | -3.794114 | 1.275906 |
| H | 0.278584 | -3.594155 | 0.315160 |
| Cu | -0.450071 | -0.278240 | -0.013664 |
| B | 1.582732 | 0.388204 | -0.074917 |
| O | 2.462828 | -0.171389 | 0.850326 |
| O | 2.136856 | 0.385228 | -1.344919 |
| C | 3.760238 | -0.336519 | 0.201326 |
| C | 3.355197 | -0.415387 | -1.312012 |
| Cl | -0.676595 | 3.737318 | 0.968738 |
| H | 0.866591 | -1.551076 | 1.595503 |
| C | -1.008454 | -1.440604 | 2.777451 |
| H | -0.631729 | -1.926985 | 3.692674 |
| H | -0.932919 | -0.358815 | 2.923390 |
| C | 2.956730 | -1.828197 | -1.761065 |
| H | 2.492603 | -1.767255 | -2.750297 |
| H | 3.825158 | -2.493288 | -1.822422 |
| H | 2.224415 | -2.266566 | -1.074430 |
| C | 4.365313 | 0.185852 | -2.284079 |
| H | 5.324789 | -0.341481 | -2.217922 |
| H | 3.992071 | 0.091606 | -3.309106 |
| H | 4.532255 | 1.246413 | -2.083077 |
| C | 4.586851 | 0.908439 | 0.545863 |
| H | 4.649952 | 1.002244 | 1.634591 |
| H | 5.603028 | 0.839214 | 0.142328 |
| H | 4.113698 | 1.815117 | 0.156261 |
| C | 4.428150 | -1.589873 | 0.758771 |
| H | 5.366412 | -1.794269 | 0.229286 |
| H | 4.659433 | -1.444795 | 1.819358 |
| H | 3.781480 | -2.466706 | 0.669443 |
| H | -2.058370 | -1.722831 | 2.655482 |

Electronic Energy = -1800.02714147 (Hartree/Particle)
Dipole Moment (Debye): 6.4129
index 1
Number of imaginary frequencies= 1
Negatives Eigenvalues: -190.930

THERMOCHEMICAL DATA

```
 Temperature   298.150 Kelvin.  Pressure   1.00000 Atm.
 Zero-point correction=                           0.362121
(Hartree/Particle)
 Thermal correction to Energy=                    0.388657
 Thermal correction to Enthalpy=                  0.389601
 Thermal correction to Gibbs Free Energy=         0.303194
 Sum of electronic and zero-point Energies=          -1799.665020
 Sum of electronic and thermal Energies=             -1799.638484
 Sum of electronic and thermal Enthalpies=           -1799.637540
 Sum of electronic and thermal Free Energies=        -1799.723947
 PCM-SP (solvent=THF;6-311+g(2d,p)) = -1800.34663554
 Corrected Free Energy =   -1800.04344107
```

-------------------------------------------------------

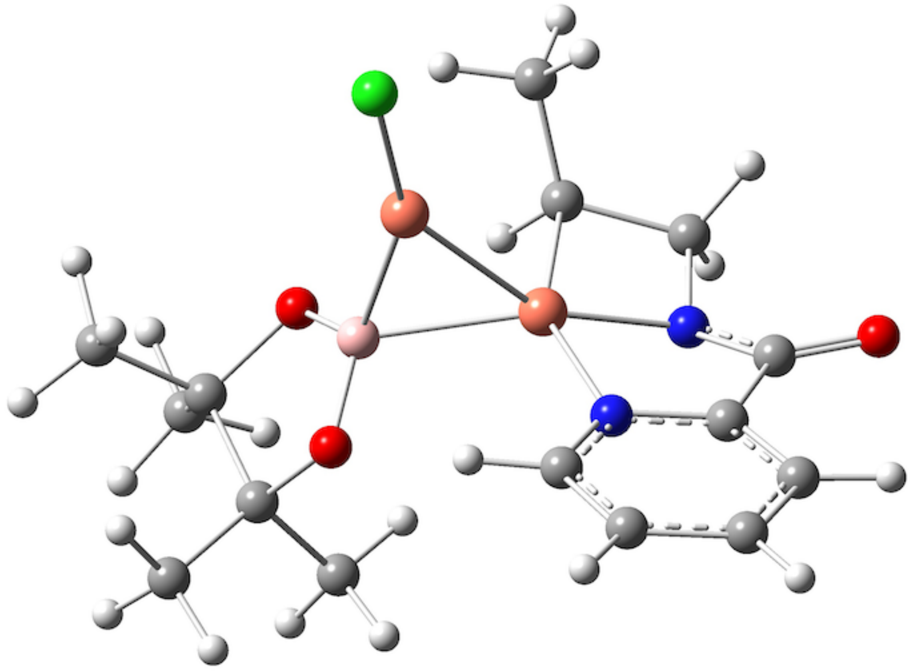

I2 Substrate: N-(2-picolinoyl)-methyl aziridine
Path: Markovnikov

CARTESIAN COORDINATES

| | | | |
|---|---|---|---|
| C | -0.394249 | -1.593597 | -2.133975 |
| N | -1.977476 | -0.078973 | -1.875421 |
| C | -3.189006 | 0.299998 | -1.400605 |
| O | -4.286754 | 0.105715 | -1.921992 |
| C | -1.703020 | -1.160293 | -2.806068 |
| C | -3.039332 | 1.053451 | -0.085581 |
| C | -4.088590 | 1.756407 | 0.496931 |
| C | -3.863247 | 2.394447 | 1.719818 |
| C | -2.604772 | 2.303441 | 2.320696 |
| C | -1.606030 | 1.567171 | 1.681643 |
| N | -1.830325 | 0.963981 | 0.507819 |
| Cu | 0.398391 | -1.444889 | 1.167350 |
| H | -5.047600 | 1.779500 | -0.010100 |
| H | -4.661485 | 2.952551 | 2.201358 |
| H | -0.607876 | 1.446070 | 2.090201 |

| | | | |
|---|---|---|---|
| H | -2.396534 | 2.783422 | 3.271482 |
| H | -2.476798 | -1.938698 | -2.781389 |
| H | -1.585086 | -0.817724 | -3.842509 |
| Cu | -0.647259 | -0.288024 | -0.596675 |
| B | 1.391171 | -0.050528 | 0.066788 |
| O | 2.418136 | -0.435105 | -0.766617 |
| O | 1.585977 | 1.212366 | 0.592868 |
| C | 3.508122 | 0.528166 | -0.594194 |
| C | 2.739274 | 1.802712 | -0.092156 |
| Cl | 0.168055 | -2.816333 | 2.777292 |
| H | 0.515373 | -1.195961 | -2.587136 |
| C | -0.287233 | -3.003572 | -1.615908 |
| H | -1.121946 | -3.262782 | -0.956228 |
| H | -0.319555 | -3.696450 | -2.474131 |
| C | 2.168411 | 2.658512 | -1.229132 |
| H | 1.481047 | 3.397994 | -0.806300 |
| H | 2.960340 | 3.188655 | -1.769017 |
| H | 1.607213 | 2.045201 | -1.941883 |
| C | 3.504842 | 2.669405 | 0.901204 |
| H | 4.425774 | 3.051863 | 0.445156 |
| H | 2.889262 | 3.525482 | 1.196638 |
| H | 3.765550 | 2.111887 | 1.803596 |
| C | 4.453896 | -0.065471 | 0.456036 |
| H | 4.789339 | -1.049661 | 0.114831 |
| H | 5.333533 | 0.569947 | 0.605357 |
| H | 3.946660 | -0.197630 | 1.416915 |
| C | 4.224245 | 0.692221 | -1.930369 |
| H | 4.990237 | 1.473775 | -1.861057 |
| H | 4.717648 | -0.246689 | -2.201645 |
| H | 3.529342 | 0.953737 | -2.732016 |
| H | 0.645828 | -3.166586 | -1.070516 |

Electronic Energy = -1800.03685215 (Hartree/Particle)
Dipole Moment (Debye): 7.8243
index 0
Harmonic frequencies
Number of imaginary frequencies= 0

THERMOCHEMICAL DATA

Temperature 298.150 Kelvin. Pressure 1.00000 Atm.
Zero-point correction= 0.363284
(Hartree/Particle)
Thermal correction to Energy= 0.390038
Thermal correction to Enthalpy= 0.390982
Thermal correction to Gibbs Free Energy= 0.303141
Sum of electronic and zero-point Energies= -1799.673568
Sum of electronic and thermal Energies= -1799.646814
Sum of electronic and thermal Enthalpies= -1799.645870
Sum of electronic and thermal Free Energies= -1799.733711
PCM-SP (solvent=THF;6-311+g(2d,p)) = -1800.35676461
Corrected Free Energy = -1800.05362346

---

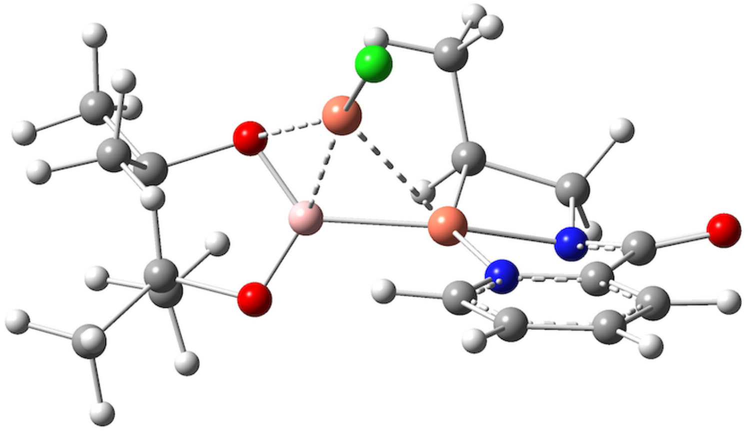

TS4 Substrate: N-(2-picolinoyl)-methyl aziridine
Path: Markovnikov

CARTESIAN COORDINATES

| | | | |
|---|---|---|---|
| N | 2.043960 | -0.513115 | 0.831478 |
| C | 3.276094 | 0.028346 | 0.698996 |
| C | 4.429482 | -0.707834 | 0.946183 |
| C | 4.300664 | -2.037184 | 1.356122 |
| C | 3.025580 | -2.589926 | 1.487773 |
| C | 1.916688 | -1.793544 | 1.202394 |
| C | 3.327838 | 1.465365 | 0.194904 |
| O | 4.376960 | 1.980285 | -0.198708 |
| Cu | 0.634487 | 0.863847 | 0.214330 |
| N | 2.100578 | 2.019708 | 0.255738 |
| C | 1.605442 | 3.149767 | -0.508679 |
| C | 0.197421 | 2.585220 | -0.771824 |
| B | -1.309955 | 0.321497 | 0.346155 |
| Cu | -0.470663 | -1.010133 | -1.165287 |
| Cl | 0.533387 | -2.712062 | -1.948726 |
| O | -2.252446 | 0.212520 | -0.681451 |
| C | -3.521272 | -0.252613 | -0.073496 |
| C | -4.670559 | 0.379590 | -0.847697 |
| O | -1.891552 | 0.217067 | 1.581360 |
| C | -3.349745 | 0.244899 | 1.404243 |
| C | -3.785615 | 1.699325 | 1.612409 |
| C | -3.540793 | -1.776007 | -0.215728 |
| C | -3.977091 | -0.660347 | 2.458291 |
| H | 5.391504 | -0.228694 | 0.797146 |
| H | 5.183653 | -2.637899 | 1.556442 |
| H | 0.904736 | -2.180386 | 1.240531 |
| H | 2.882755 | -3.624438 | 1.782163 |
| H | 1.602463 | 4.082249 | 0.071190 |
| H | -4.481020 | -2.196656 | 0.155400 |
| H | -3.433112 | -2.038780 | -1.272612 |
| H | -2.709364 | -2.237653 | 0.326532 |
| H | -4.692393 | -0.018626 | -1.867087 |
| H | -5.627173 | 0.142640 | -0.367263 |
| H | -4.569248 | 1.465433 | -0.908806 |
| H | -3.800437 | -0.242216 | 3.454314 |
| H | -5.060161 | -0.734198 | 2.303855 |
| H | -3.549508 | -1.665071 | 2.432409 |
| H | -4.874774 | 1.801774 | 1.558743 |
| H | -3.453261 | 2.030480 | 2.600994 |

| | | | |
|---|---|---|---|
| H | -3.335254 | 2.359963 | 0.864265 |
| H | 2.175976 | 3.318793 | -1.432641 |
| C | -0.140344 | 2.249048 | -2.206475 |
| H | 0.638038 | 1.628188 | -2.664334 |
| H | -1.095603 | 1.725126 | -2.289959 |
| H | -0.198847 | 3.182544 | -2.790232 |
| H | -0.605884 | 3.077540 | -0.220315 |

Electronic Energy = -1800.02792406 (Hartree/Particle)
Dipole Moment (Debye): 7.6917
index 0
Harmonic frequencies
Number of imaginary frequencies= 1
Negatives Eigenvalues: -82.020

THERMOCHEMICAL DATA

Temperature 298.150 Kelvin. Pressure 1.00000 Atm.
Zero-point correction= 0.363138 (Hartree/Particle)
Thermal correction to Energy= 0.389075
Thermal correction to Enthalpy= 0.390020
Thermal correction to Gibbs Free Energy= 0.305246
Sum of electronic and zero-point Energies= -1799.664786
Sum of electronic and thermal Energies= -1799.638849
Sum of electronic and thermal Enthalpies= -1799.637904
Sum of electronic and thermal Free Energies= -1799.722678
PCM-SP (solvent=THF;6-311+g(2d,p)) = -1800.34779585
Corrected Free Energy = -1800.04254979

------------------------------------------------------

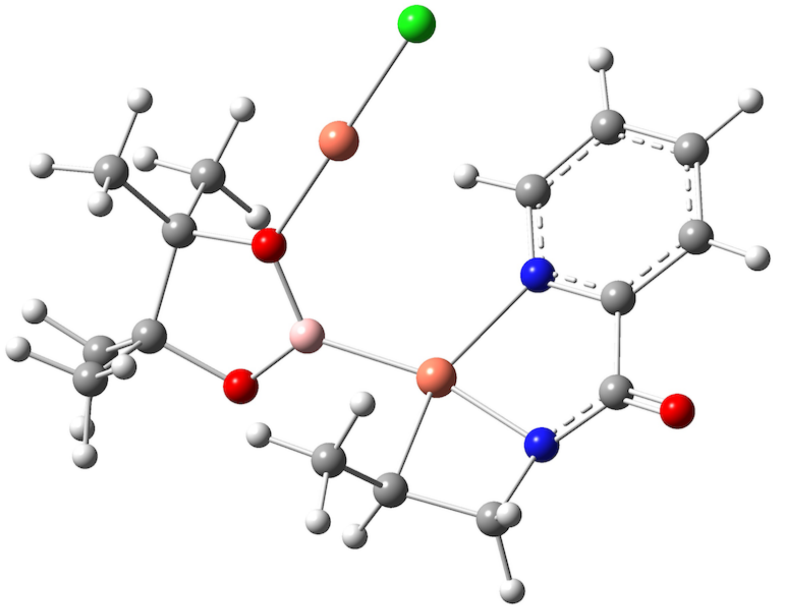

I3 Substrate: N-(2-picolinoyl)-methyl aziridine
Path: Markovnikov

CARTESIAN COORDINATES

| | | | |
|---|---|---|---|
| N | 1.929477 | -0.266595 | 0.910762 |
| C | 3.196659 | 0.026109 | 0.530825 |
| C | 4.223969 | -0.905395 | 0.636390 |

| | | | |
|---|---|---|---|
| C | 3.937961 | -2.167786 | 1.161555 |
| C | 2.632848 | -2.460595 | 1.558735 |
| C | 1.654564 | -1.479016 | 1.410398 |
| C | 3.418273 | 1.405606 | -0.084362 |
| O | 4.454277 | 1.677867 | -0.697846 |
| Cu | 0.711937 | 1.304926 | 0.362896 |
| N | 2.346652 | 2.186277 | 0.147739 |
| C | 1.920214 | 3.336509 | -0.634493 |
| C | 0.412127 | 3.012296 | -0.655240 |
| B | -1.201238 | 0.817754 | 0.405110 |
| Cu | -0.666254 | -1.841094 | -0.865156 |
| Cl | 0.466756 | -3.497035 | -1.483125 |
| O | -1.715865 | -0.375680 | -0.165072 |
| C | -3.125254 | -0.553350 | 0.290347 |
| C | -3.910528 | -1.224721 | -0.827597 |
| O | -2.226595 | 1.533668 | 0.952782 |
| C | -3.512794 | 0.930328 | 0.596910 |
| C | -4.030013 | 1.688651 | -0.630205 |
| C | -3.060293 | -1.450247 | 1.528234 |
| C | -4.467820 | 1.104054 | 1.773316 |
| H | 5.212132 | -0.624108 | 0.287568 |
| H | 4.719182 | -2.918070 | 1.245703 |
| H | 0.618690 | -1.665443 | 1.674333 |
| H | 2.360692 | -3.436696 | 1.945375 |
| H | 2.152346 | 4.290590 | -0.142285 |
| H | -4.063636 | -1.673457 | 1.904775 |
| H | -2.575082 | -2.394848 | 1.259307 |
| H | -2.480473 | -0.981342 | 2.329223 |
| H | -3.541270 | -2.244545 | -0.981429 |
| H | -4.971558 | -1.282447 | -0.559143 |
| H | -3.815444 | -0.684951 | -1.771958 |
| H | -4.680606 | 2.168035 | 1.916339 |
| H | -5.415414 | 0.587796 | 1.579486 |
| H | -4.039698 | 0.718353 | 2.701191 |
| H | -5.031318 | 1.349323 | -0.915966 |
| H | -4.079264 | 2.755223 | -0.390918 |
| H | -3.362152 | 1.562824 | -1.487720 |
| H | 2.367951 | 3.349413 | -1.638201 |
| C | -0.193975 | 2.761190 | -2.019234 |
| H | 0.357062 | 1.989166 | -2.567858 |
| H | -1.242673 | 2.456245 | -1.955873 |
| H | -0.143674 | 3.685568 | -2.617348 |
| H | -0.202339 | 3.636653 | -0.003395 |

Electronic Energy = -1800.03653571 (Hartree/Particle)
Dipole Moment (Debye): 8.8354
index 0
Harmonic frequencies
Number of imaginary frequencies= 0

THERMOCHEMICAL DATA

Temperature 298.150 Kelvin. Pressure 1.00000 Atm.
Zero-point correction= 0.363978
(Hartree/Particle)
Thermal correction to Energy= 0.390383
Thermal correction to Enthalpy= 0.391327
Thermal correction to Gibbs Free Energy= 0.304940
Sum of electronic and zero-point Energies= -1799.672558
Sum of electronic and thermal Energies= -1799.646152

Sum of electronic and thermal Enthalpies= -1799.645208
Sum of electronic and thermal Free Energies= -1799.731596
PCM-SP (solvent=THF;6-311+g(2d,p)) = -1800.35759687
Corrected Free Energy = -1800.05265716

-------------------------------------------------------

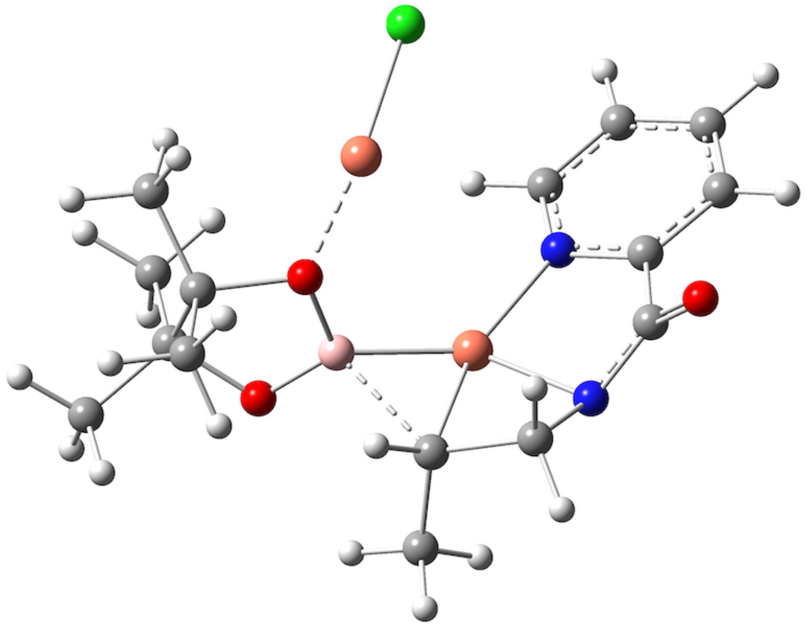

TS5 Substrate: N-(2-picolinoyl)-methyl aziridine
Path: Markovnikov

CARTESIAN COORDINATES

| | | | |
|---|---|---|---|
| C | 0.709728 | 2.906877 | -1.048611 |
| Cu | 0.614894 | 1.068904 | 0.589712 |
| B | -1.299127 | 0.728479 | 0.296749 |
| O | -1.654433 | -0.141069 | -0.773337 |
| C | -3.132654 | -0.360112 | -0.680011 |
| C | -3.368754 | -0.128029 | 0.850278 |
| O | -2.335529 | 0.855337 | 1.180298 |
| Cu | -0.372549 | -1.619071 | -0.720429 |
| Cl | 0.821985 | -3.339922 | -0.519811 |
| C | -0.548898 | 2.530941 | -0.196989 |
| N | 1.815604 | 2.345359 | -0.270894 |
| C | 2.790361 | 1.641074 | -0.867206 |
| C | 3.212113 | 0.454122 | 0.008174 |
| C | 4.469071 | -0.135867 | -0.085775 |
| C | 4.746359 | -1.268627 | 0.680510 |
| C | 3.745319 | -1.804724 | 1.493493 |
| C | 2.496860 | -1.189595 | 1.509786 |
| N | 2.242020 | -0.079495 | 0.798919 |
| O | 3.285856 | 1.760898 | -1.997597 |
| H | 5.191824 | 0.298655 | -0.768136 |
| H | 5.724529 | -1.739492 | 0.631343 |
| H | 1.666704 | -1.598924 | 2.073217 |
| H | 3.908673 | -2.703550 | 2.078661 |
| H | 0.750321 | 4.001616 | -1.165677 |
| H | -1.425262 | 2.444515 | -0.850461 |
| C | -3.466340 | -1.752196 | -1.194023 |

```
 H              -4.541101   -1.937293   -1.085038
 H              -3.211039   -1.829719   -2.255469
 H              -2.925905   -2.535842   -0.657310
 C              -3.759636    0.711825   -1.572261
 H              -3.354833    0.618539   -2.584469
 H              -4.846158    0.588040   -1.620163
 H              -3.548966    1.721412   -1.206832
 C              -3.094459   -1.371196    1.703383
 H              -3.074374   -1.077377    2.757080
 H              -3.875242   -2.126536    1.567379
 H              -2.127319   -1.823533    1.458383
 C              -4.727193    0.473628    1.193610
 H              -5.534062   -0.193757    0.869262
 H              -4.806240    0.604343    2.277264
 H              -4.866966    1.449993    0.724028
 C              -0.810851    3.528060    0.933153
 H               0.097697    3.693462    1.520959
 H              -1.101771    4.493651    0.495966
 H              -1.608280    3.195577    1.602378
 H               0.664971    2.467879   -2.054677

Electronic Energy =  -1800.02666080 (Hartree/Particle)
Dipole Moment (Debye):      8.6356
index          0
Harmonic frequencies
Number of imaginary frequencies=           1
Negatives Eigenvalues:           -143.049

THERMOCHEMICAL DATA

Temperature   298.150 Kelvin.  Pressure   1.00000 Atm.
Zero-point correction=                           0.363669
(Hartree/Particle)
Thermal correction to Energy=                    0.389141
Thermal correction to Enthalpy=                  0.390085
Thermal correction to Gibbs Free Energy=         0.307696
Sum of electronic and zero-point Energies=          -1799.662992
Sum of electronic and thermal Energies=             -1799.637520
Sum of electronic and thermal Enthalpies=           -1799.636576
Sum of electronic and thermal Free Energies=        -1799.718964
PCM-SP (solvent=THF;6-311+g(2d,p)) =   -1800.35044651
Corrected Free Energy =   -1800.04274971
```

---

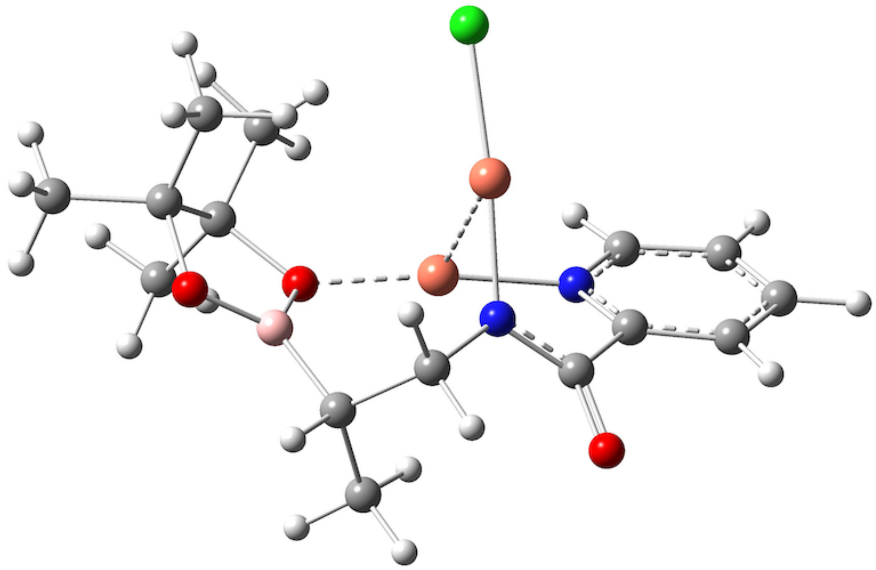

P2 Substrate: N-(2-picolinoyl)-methyl aziridine
Path: Markovnikov

CARTESIAN COORDINATES

| | | | |
|---|---|---|---|
| C | -0.071125 | 1.952779 | 1.759067 |
| Cu | -0.387242 | -0.018054 | -0.964649 |
| B | 1.911430 | 1.392783 | 0.177509 |
| O | 3.199224 | 1.161057 | 0.544167 |
| C | 3.635264 | -0.127104 | -0.000813 |
| C | 2.682442 | -0.322711 | -1.234822 |
| O | 1.502691 | 0.476932 | -0.813087 |
| Cu | -0.541361 | -0.882801 | 1.454538 |
| Cl | 0.058004 | -2.913554 | 1.609404 |
| C | 0.981534 | 2.521149 | 0.758422 |
| N | -0.962647 | 0.958963 | 1.136070 |
| C | -2.191803 | 1.395492 | 0.766261 |
| C | -2.977228 | 0.438783 | -0.123422 |
| C | -4.364611 | 0.350993 | -0.006133 |
| C | -5.066710 | -0.507014 | -0.849763 |
| C | -4.365328 | -1.233825 | -1.816727 |
| C | -2.987110 | -1.069778 | -1.903873 |
| N | -2.305104 | -0.255453 | -1.075129 |
| O | -2.721428 | 2.487233 | 1.020806 |
| H | -4.857239 | 0.959324 | 0.744228 |
| H | -6.144725 | -0.607402 | -0.759179 |
| H | -2.395295 | -1.594846 | -2.645800 |
| H | -4.872346 | -1.910803 | -2.496697 |
| H | -0.666239 | 2.782655 | 2.156970 |
| H | 1.628242 | 3.186480 | 1.348758 |
| C | 3.417396 | -1.162994 | 1.104657 |
| H | 3.778891 | -2.150982 | 0.801854 |
| H | 3.971474 | -0.847802 | 1.994270 |
| H | 2.361660 | -1.261497 | 1.374029 |
| C | 5.113594 | -0.019870 | -0.359903 |
| H | 5.699514 | 0.120160 | 0.553682 |
| H | 5.455819 | -0.940004 | -0.847523 |
| H | 5.312247 | 0.825834 | -1.022648 |
| C | 2.223753 | -1.753880 | -1.477710 |
| H | 1.557544 | -1.783928 | -2.348222 |

| | | | |
|---|---|---|---|
| H | 3.085861 | -2.395620 | -1.691446 |
| H | 1.684819 | -2.161184 | -0.618210 |
| C | 3.178973 | 0.332879 | -2.523174 |
| H | 4.030889 | -0.214844 | -2.938532 |
| H | 2.370774 | 0.326720 | -3.261805 |
| H | 3.478726 | 1.371756 | -2.353358 |
| C | 0.307700 | 3.353227 | -0.353533 |
| H | -0.359840 | 2.736681 | -0.967663 |
| H | -0.301911 | 4.155651 | 0.076081 |
| H | 1.050132 | 3.803251 | -1.024334 |
| H | 0.447177 | 1.476576 | 2.598487 |

Electronic Energy = -1800.13343971 (Hartree/Particle)
Dipole Moment (Debye): 6.9196
index 0
Harmonic frequencies
Number of imaginary frequencies= 0

THERMOCHEMICAL DATA

Temperature 298.150 Kelvin. Pressure 1.00000 Atm.
Zero-point correction= 0.366778 (Hartree/Particle)
Thermal correction to Energy= 0.392409
Thermal correction to Enthalpy= 0.393353
Thermal correction to Gibbs Free Energy= 0.311753
Sum of electronic and zero-point Energies= -1799.766661
Sum of electronic and thermal Energies= -1799.741031
Sum of electronic and thermal Enthalpies= -1799.740087
Sum of electronic and thermal Free Energies= -1799.821687
PCM-SP (solvent=THF;6-311+g(2d,p)) = -1800.45193594
Corrected Free Energy = -1800.14018323

---

SUBSTRATE: 1c

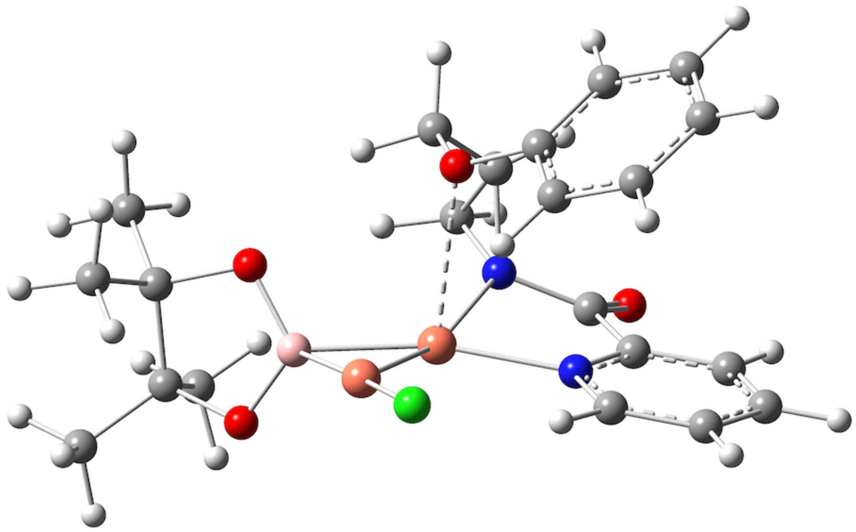

Complex-2 Substrate: 1c

CARTESIAN COORDINATES

| | | | |
|---|---|---|---|
| C | -0.675681 | -2.517430 | 1.139506 |

| | | | |
|---|---|---|---|
| N | -0.432016 | -2.201179 | -0.310580 |
| C | -1.535437 | -2.522324 | -1.171984 |
| O | -1.810629 | -3.654713 | -1.517914 |
| C | 0.271995 | -3.322403 | 0.323363 |
| C | -2.316464 | -1.319282 | -1.602499 |
| C | -3.592535 | -1.461115 | -2.145870 |
| C | -4.289292 | -0.303807 | -2.502135 |
| C | -3.683483 | 0.937935 | -2.309055 |
| C | -2.394078 | 0.991380 | -1.770933 |
| N | -1.727688 | -0.118935 | -1.428000 |
| Cu | 0.833851 | 2.030227 | -0.401055 |
| H | -4.013040 | -2.453091 | -2.271067 |
| H | -5.289730 | -0.372946 | -2.919638 |
| H | -1.872960 | 1.930400 | -1.597296 |
| H | -4.192925 | 1.861066 | -2.566739 |
| H | -0.025342 | -4.320214 | 0.012045 |
| H | 1.337243 | -3.142118 | 0.438163 |
| H | -1.646657 | -2.975014 | 1.316127 |
| C | -0.210824 | -1.476625 | 2.143267 |
| H | -0.460860 | -1.810209 | 3.160258 |
| H | 0.868345 | -1.331853 | 2.065798 |
| O | -0.736407 | -0.181682 | 1.879816 |
| C | -2.073765 | 0.099188 | 2.023030 |
| C | -2.450303 | 1.375413 | 1.580329 |
| C | -3.024113 | -0.774918 | 2.561703 |
| C | -3.782791 | 1.767219 | 1.670248 |
| H | -1.691728 | 2.036763 | 1.169444 |
| C | -4.362337 | -0.364396 | 2.644305 |
| H | -2.744078 | -1.755564 | 2.932025 |
| C | -4.750022 | 0.899137 | 2.197414 |
| H | -4.064148 | 2.758875 | 1.325185 |
| H | -5.098319 | -1.044714 | 3.065803 |
| H | -5.789077 | 1.209490 | 2.265522 |
| Cu | 0.189212 | -0.147938 | -0.676365 |
| B | 2.138701 | 0.364279 | -0.230853 |
| O | 2.592316 | -0.109124 | 1.006430 |
| O | 3.171758 | 0.337051 | -1.160214 |
| C | 4.045003 | -0.201506 | 0.958281 |
| C | 4.311314 | -0.355442 | -0.580075 |
| Cl | -0.294217 | 3.879461 | -0.448615 |
| C | 4.588959 | 1.109561 | 1.541243 |
| H | 4.188747 | 1.240583 | 2.551666 |
| H | 5.683325 | 1.102916 | 1.596782 |
| H | 4.270833 | 1.967270 | 0.940568 |
| C | 4.489262 | -1.384273 | 1.814838 |
| H | 5.572531 | -1.535423 | 1.734771 |
| H | 4.248997 | -1.189967 | 2.865745 |
| H | 3.987932 | -2.309503 | 1.517935 |
| C | 5.594766 | 0.300397 | -1.082312 |
| H | 6.472124 | -0.142531 | -0.594956 |
| H | 5.688444 | 0.147777 | -2.162619 |
| H | 5.593935 | 1.376389 | -0.894447 |
| C | 4.234630 | -1.809234 | -1.068026 |
| H | 4.223965 | -1.814799 | -2.162478 |
| H | 5.091412 | -2.399168 | -0.723402 |
| H | 3.313205 | -2.289366 | -0.721977 |

Electronic Energy = -2106.33708712 (Hartree/Particle)
Dipole Moment (Debye): 8.9849
index 0

Harmonic frequencies
Number of imaginary frequencies= 0

THERMOCHEMICAL DATA

Temperature 298.150 Kelvin. Pressure 1.00000 Atm.
Zero-point correction= 0.450209
(Hartree/Particle)
Thermal correction to Energy= 0.482319
Thermal correction to Enthalpy= 0.483263
Thermal correction to Gibbs Free Energy= 0.383962
Sum of electronic and zero-point Energies= -2105.886878
Sum of electronic and thermal Energies= -2105.854768
Sum of electronic and thermal Enthalpies= -2105.853824
Sum of electronic and thermal Free Energies= -2105.953125
PCM-SP (solvent=THF;6-311+g(2d,p)) = -2106.73911105
Corrected Free Energy = -2106.35514893

------------------------------------------------------

PATH: ANTI_MARKOVNIKOV

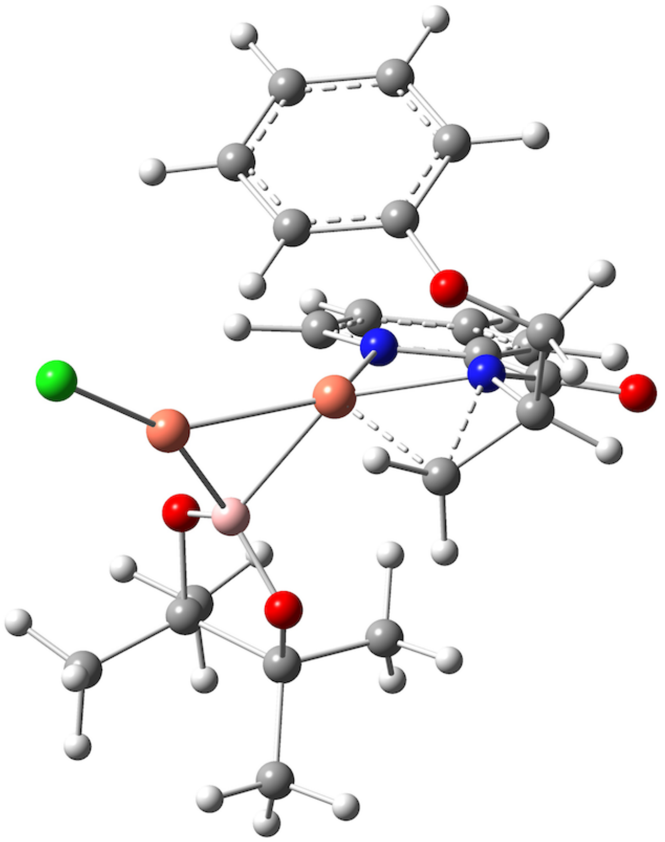

TS3 Substrate: 1c
Path: anti-Markovnikov

CARTESIAN COORDINATES

| | | | |
|---|---|---|---|
| C | -0.939688 | 0.028723 | 2.339981 |
| N | -1.423217 | 1.330809 | 1.138654 |
| C | -0.898309 | 2.604318 | 0.919365 |
| O | -1.123135 | 3.571763 | 1.640774 |
| C | -1.995004 | 1.062073 | 2.443570 |

| | | | |
|---|---|---|---|
| C | -0.112207 | 2.740730 | -0.356306 |
| C | 0.348875 | 3.987204 | -0.780848 |
| C | 1.082851 | 4.050986 | -1.966591 |
| C | 1.344807 | 2.872669 | -2.671151 |
| C | 0.868578 | 1.663316 | -2.161227 |
| N | 0.143678 | 1.609923 | -1.037686 |
| Cu | 0.136511 | -2.333519 | -0.203162 |
| H | 0.133632 | 4.863855 | -0.179649 |
| H | 1.452393 | 5.005127 | -2.331839 |
| H | 1.080744 | 0.709440 | -2.632014 |
| H | 1.914669 | 2.882407 | -3.595015 |
| H | -1.814171 | 1.863169 | 3.165469 |
| H | 0.046986 | 0.211354 | 2.751928 |
| Cu | -0.341206 | -0.045167 | 0.123862 |
| B | 1.590508 | -0.961121 | 0.139814 |
| O | 2.498910 | -0.745689 | -0.887758 |
| O | 2.164013 | -0.669447 | 1.375345 |
| C | 3.816703 | -0.535938 | -0.299235 |
| C | 3.454674 | -0.029718 | 1.141185 |
| H | -1.223014 | -0.999818 | 2.131932 |
| C | -3.461233 | 0.637461 | 2.444179 |
| H | -3.746775 | 0.342171 | 3.458564 |
| H | -4.081705 | 1.493632 | 2.152378 |
| O | -3.715304 | -0.508041 | 1.641302 |
| C | -3.664212 | -0.397643 | 0.261020 |
| C | -3.096085 | -1.482167 | -0.412953 |
| C | -4.153191 | 0.702384 | -0.450919 |
| C | -2.980357 | -1.453589 | -1.805647 |
| H | -2.734723 | -2.337779 | 0.147841 |
| C | -4.035741 | 0.719399 | -1.843961 |
| H | -4.602907 | 1.545293 | 0.063499 |
| C | -3.443042 | -0.348516 | -2.527207 |
| H | -2.509986 | -2.298253 | -2.300953 |
| H | -4.410073 | 1.578438 | -2.395727 |
| H | -3.349729 | -0.321108 | -3.609511 |
| Cl | -0.756515 | -4.256818 | -0.573978 |
| C | 4.421278 | -0.458722 | 2.240323 |
| H | 5.427023 | -0.068251 | 2.043127 |
| H | 4.084848 | -0.063661 | 3.204873 |
| H | 4.476871 | -1.546422 | 2.322462 |
| C | 3.208557 | 1.484053 | 1.206981 |
| H | 2.761298 | 1.730865 | 2.175688 |
| H | 4.140887 | 2.049818 | 1.103851 |
| H | 2.516902 | 1.804487 | 0.422483 |
| C | 4.526194 | -1.895385 | -0.305733 |
| H | 4.558645 | -2.272695 | -1.332563 |
| H | 5.552465 | -1.814845 | 0.069538 |
| H | 3.983551 | -2.625665 | 0.302473 |
| C | 4.577618 | 0.466848 | -1.162487 |
| H | 5.539998 | 0.721381 | -0.702447 |
| H | 4.775005 | 0.028932 | -2.146680 |
| H | 4.006041 | 1.386812 | -1.309566 |

Electronic Energy = -2106.30303380 (Hartree/Particle)
Dipole Moment (Debye): 11.4790
index 1
Number of imaginary frequencies= 1
Negatives Eigenvalues: -279.645

THERMOCHEMICAL DATA

Temperature 298.150 Kelvin. Pressure 1.00000 Atm.
Zero-point correction= 0.448004
(Hartree/Particle)
Thermal correction to Energy= 0.479707
Thermal correction to Enthalpy= 0.480651
Thermal correction to Gibbs Free Energy= 0.381979
Sum of electronic and zero-point Energies= -2105.855030
Sum of electronic and thermal Energies= -2105.823327
Sum of electronic and thermal Enthalpies= -2105.822383
Sum of electronic and thermal Free Energies= -2105.921054
PCM-SP (solvent=THF;6-311+g(2d,p)) = -2106.70700049
Corrected Free Energy = -2106.32502069

------------------------------------------------------

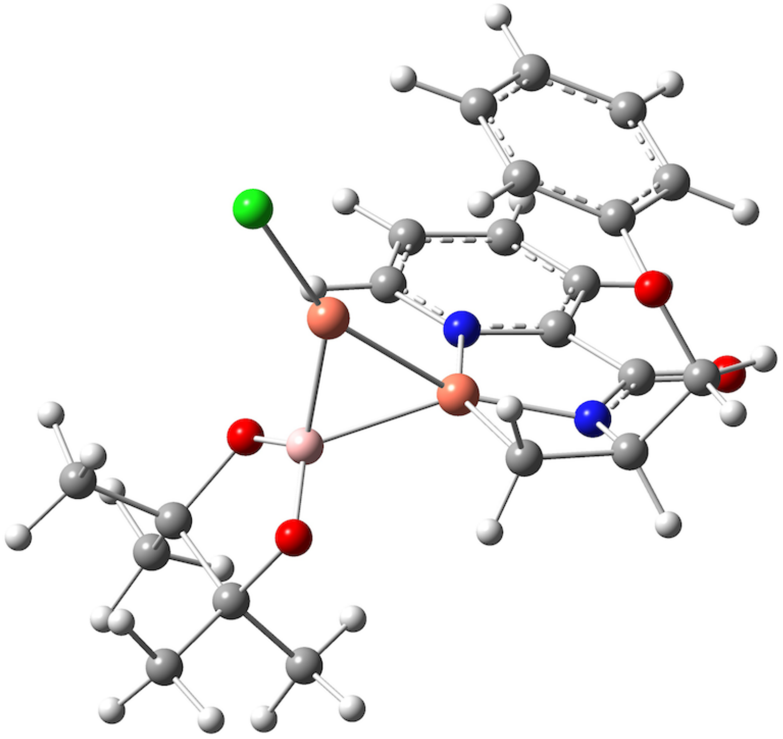

I2 Substrate: 1c
Path: anti-Markovnikov

CARTESIAN COORDINATES

| | | | |
|---|---|---|---|
| C | -0.283339 | -0.873174 | 2.354926 |
| N | -1.396000 | 0.898409 | 1.698411 |
| C | -2.231250 | 1.811555 | 1.175858 |
| O | -3.250876 | 2.286255 | 1.683143 |
| C | -1.507183 | -0.047942 | 2.793760 |
| C | -1.760038 | 2.208110 | -0.217429 |
| C | -2.411621 | 3.180882 | -0.966555 |
| C | -1.956610 | 3.443826 | -2.260614 |
| C | -0.867303 | 2.726886 | -2.761813 |
| C | -0.258153 | 1.770567 | -1.948485 |
| N | -0.700879 | 1.527112 | -0.708070 |
| Cu | 0.776041 | -1.696960 | -0.578191 |

| | | | |
|---|---|---|---|
| H | -3.261154 | 3.691718 | -0.525185 |
| H | -2.448636 | 4.194512 | -2.873100 |
| H | 0.602034 | 1.190994 | -2.264664 |
| H | -0.489295 | 2.899543 | -3.764370 |
| H | -1.379483 | 0.436734 | 3.773751 |
| H | 0.655213 | -0.694154 | 2.876973 |
| Cu | -0.098904 | 0.134248 | 0.630504 |
| B | 1.966812 | -0.218746 | 0.131727 |
| O | 2.506183 | 0.635197 | -0.809763 |
| O | 2.808401 | -0.428521 | 1.199036 |
| C | 3.928362 | 0.790766 | -0.486892 |
| C | 3.943525 | 0.485868 | 1.053915 |
| H | -0.460189 | -1.915965 | 2.097661 |
| C | -2.827646 | -0.837854 | 2.839763 |
| H | -2.865246 | -1.415004 | 3.768373 |
| H | -3.669326 | -0.137162 | 2.829079 |
| O | -2.940000 | -1.825212 | 1.809180 |
| C | -3.120957 | -1.430968 | 0.499478 |
| C | -2.367964 | -2.114352 | -0.463444 |
| C | -4.027792 | -0.439717 | 0.109253 |
| C | -2.481784 | -1.773026 | -1.813943 |
| H | -1.697077 | -2.908552 | -0.150879 |
| C | -4.130517 | -0.102725 | -1.243795 |
| H | -4.618551 | 0.092789 | 0.845926 |
| C | -3.356627 | -0.756083 | -2.209356 |
| H | -1.863337 | -2.298608 | -2.535829 |
| H | -4.822169 | 0.682585 | -1.538842 |
| H | -3.440714 | -0.480680 | -3.257179 |
| Cl | 0.822977 | -3.480758 | -1.755720 |
| C | 3.619879 | 1.709924 | 1.918964 |
| H | 3.449143 | 1.381485 | 2.949084 |
| H | 4.444144 | 2.431235 | 1.917379 |
| H | 2.712530 | 2.212884 | 1.568257 |
| C | 5.199650 | -0.214329 | 1.559610 |
| H | 6.083736 | 0.409632 | 1.382008 |
| H | 5.115979 | -0.390856 | 2.636889 |
| H | 5.345786 | -1.179342 | 1.069496 |
| C | 4.359062 | 2.201954 | -0.870712 |
| H | 5.393449 | 2.384933 | -0.556339 |
| H | 4.307726 | 2.320551 | -1.958039 |
| H | 3.715532 | 2.958640 | -0.415610 |
| C | 4.678354 | -0.252497 | -1.322517 |
| H | 4.453264 | -0.088463 | -2.380878 |
| H | 5.761442 | -0.172184 | -1.180482 |
| H | 4.361771 | -1.268427 | -1.066974 |

Electronic Energy = -2106.32252478 (Hartree/Particle)
Dipole Moment (Debye): 8.6048
index 0
Harmonic frequencies
Number of imaginary frequencies= 0

THERMOCHEMICAL DATA

Temperature 298.150 Kelvin. Pressure 1.00000 Atm.
Zero-point correction= 0.449823
(Hartree/Particle)
Thermal correction to Energy= 0.481425
Thermal correction to Enthalpy= 0.482369
Thermal correction to Gibbs Free Energy= 0.384450

Sum of electronic and zero-point Energies= -2105.872702
Sum of electronic and thermal Energies= -2105.841100
Sum of electronic and thermal Enthalpies= -2105.840155
Sum of electronic and thermal Free Energies= -2105.938075
PCM-SP (solvent=THF;6-311+g(2d,p)) = -2106.72339381
Corrected Free Energy = -2106.33894402

------------------------------------------------------

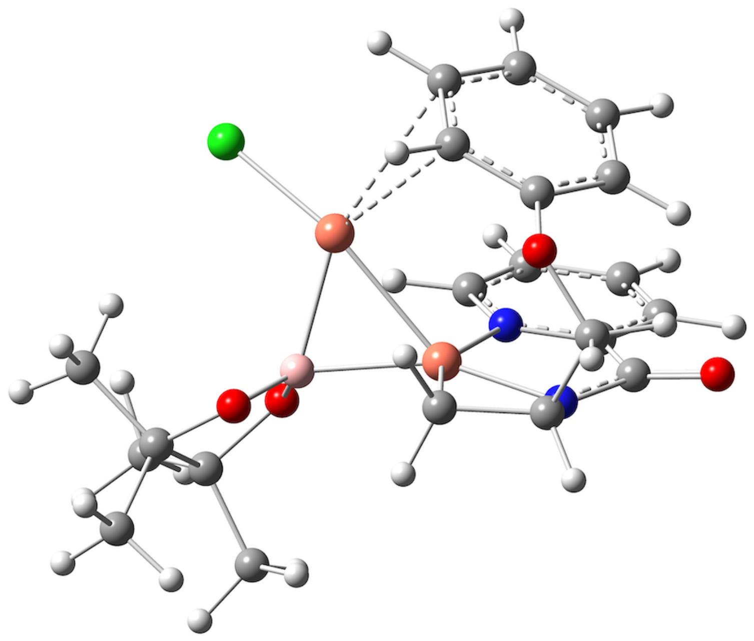

TS4a Substrate: 1c
Path: anti-Markovnikov

CARTESIAN COORDINATES

| | | | |
|---|---|---|---|
| C | 2.660184 | 2.131518 | -1.777140 |
| C | 1.723007 | 2.827917 | -1.002036 |
| C | 1.656489 | 2.613450 | 0.382520 |
| C | 2.557616 | 1.723888 | 1.001747 |
| C | 3.524163 | 1.066490 | 0.235847 |
| C | 3.555349 | 1.263544 | -1.149457 |
| O | 2.441203 | 1.611210 | 2.361643 |
| C | 2.701716 | 0.363947 | 3.026877 |
| C | 1.602986 | -0.693372 | 2.801663 |
| Cu | 0.216913 | -0.542848 | 0.661100 |
| B | -1.787492 | -0.046869 | 0.272021 |
| O | -2.692477 | 0.361046 | 1.220751 |
| C | -4.017865 | -0.059683 | 0.753052 |
| C | -3.665331 | -1.286609 | -0.167750 |
| O | -2.311967 | -0.954868 | -0.623247 |
| Cu | -0.540191 | 1.442483 | -0.449974 |
| Cl | -1.619551 | 2.608662 | -1.936964 |
| C | 0.197037 | -0.090774 | 2.595477 |
| N | 1.647472 | -1.374862 | 1.511890 |
| C | 2.656400 | -1.832971 | 0.757398 |
| C | 2.207622 | -1.928965 | -0.700134 |
| C | 2.992643 | -2.522740 | -1.681697 |
| C | 2.536640 | -2.509905 | -3.002836 |
| C | 1.315167 | -1.901166 | -3.301648 |
| C | 0.580452 | -1.323334 | -2.265496 |

| | | | |
|---|---|---|---|
| N | 1.025849 | -1.344003 | -1.002153 |
| O | 3.810684 | -2.119061 | 1.096819 |
| H | 3.939526 | -2.965798 | -1.390546 |
| H | 3.129823 | -2.965149 | -3.791385 |
| H | -0.374882 | -0.832173 | -2.421367 |
| H | 0.932962 | -1.865423 | -4.316675 |
| H | 1.658116 | -1.401631 | 3.642687 |
| H | -0.629737 | -0.663498 | 3.015268 |
| H | 0.081009 | 0.982505 | 2.723939 |
| H | 2.721156 | 0.638211 | 4.084949 |
| H | 3.678739 | -0.047447 | 2.754758 |
| H | 0.969158 | 3.177412 | 1.008822 |
| H | 4.229472 | 0.385756 | 0.697506 |
| H | 1.040938 | 3.531570 | -1.466979 |
| H | 4.296724 | 0.727503 | -1.736690 |
| H | 2.692070 | 2.278100 | -2.852466 |
| C | -4.610891 | 1.128257 | -0.011571 |
| H | -5.625819 | 0.907390 | -0.360225 |
| H | -4.655226 | 1.991578 | 0.659885 |
| H | -3.986740 | 1.407639 | -0.865110 |
| C | -4.869887 | -0.397324 | 1.971908 |
| H | -5.048523 | 0.510603 | 2.556876 |
| H | -5.841155 | -0.799358 | 1.659524 |
| H | -4.380635 | -1.127859 | 2.620939 |
| C | -4.556030 | -1.444442 | -1.394985 |
| H | -4.233251 | -2.314661 | -1.976442 |
| H | -5.598967 | -1.601081 | -1.094516 |
| H | -4.503088 | -0.564671 | -2.039885 |
| C | -3.548296 | -2.611612 | 0.593499 |
| H | -4.528162 | -2.970198 | 0.926936 |
| H | -3.109574 | -3.364703 | -0.068953 |
| H | -2.896846 | -2.508200 | 1.467489 |

Electronic Energy = -2106.32217014 (Hartree/Particle)
Dipole Moment (Debye): 4.8578
index 0
Harmonic frequencies
Number of imaginary frequencies= 1
Negatives Eigenvalues: -14.406

THERMOCHEMICAL DATA

Temperature 298.150 Kelvin. Pressure 1.00000 Atm.
Zero-point correction= 0.450137 (Hartree/Particle)
Thermal correction to Energy= 0.480568
Thermal correction to Enthalpy= 0.481512
Thermal correction to Gibbs Free Energy= 0.389003
Sum of electronic and zero-point Energies= -2105.872033
Sum of electronic and thermal Energies= -2105.841602
Sum of electronic and thermal Enthalpies= -2105.840658
Sum of electronic and thermal Free Energies= -2105.933167
PCM-SP (solvent=THF;6-311+g(2d,p)) = -2106.72148533
Corrected Free Energy = -2106.33248219

---

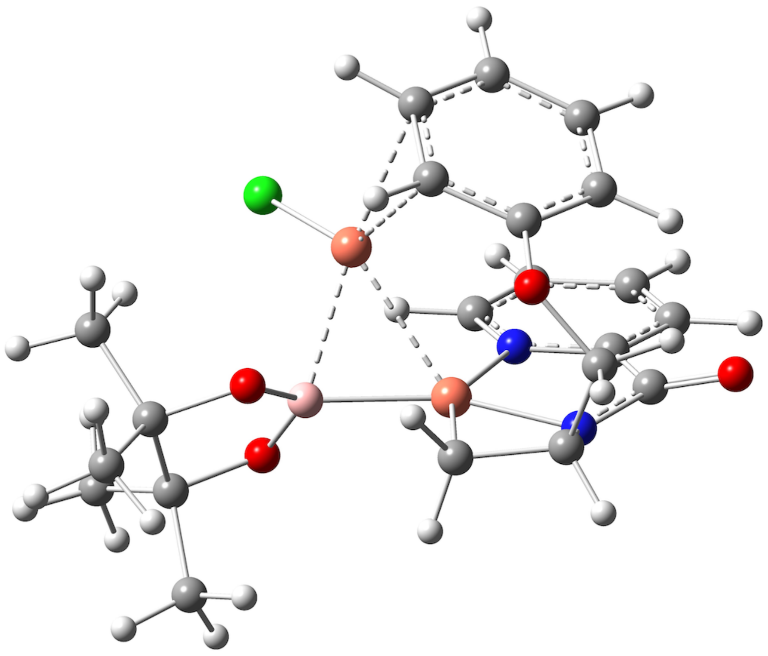

I3a Substrate: 1c
Path: anti-Markovnikov

CARTESIAN COORDINATES

| | | | |
|---|---|---|---|
| C | 2.159132 | 1.378848 | 2.728342 |
| C | 0.953236 | 0.727009 | 3.047906 |
| C | 0.739034 | -0.603004 | 2.631879 |
| C | 1.765017 | -1.299385 | 1.943310 |
| C | 2.968601 | -0.655902 | 1.653292 |
| C | 3.146301 | 0.682066 | 2.035613 |
| O | 1.488869 | -2.605038 | 1.667989 |
| C | 2.050154 | -3.276880 | 0.524879 |
| C | 1.353440 | -2.895621 | -0.795316 |
| Cu | 0.242587 | -0.605690 | -0.838832 |
| B | -1.699531 | -0.236807 | -0.369641 |
| O | -2.505148 | -0.891604 | 0.538049 |
| C | -3.890561 | -0.484890 | 0.268614 |
| C | -3.810342 | -0.005840 | -1.232468 |
| O | -2.402474 | 0.373796 | -1.374995 |
| Cu | -0.416858 | 0.783836 | 1.140867 |
| Cl | -1.191633 | 2.841412 | 1.137121 |
| C | -0.140429 | -2.545851 | -0.610157 |
| N | 1.743211 | -1.606450 | -1.353298 |
| C | 2.927275 | -0.992976 | -1.443394 |
| C | 2.706824 | 0.509344 | -1.598573 |
| C | 3.738087 | 1.370514 | -1.953727 |
| C | 3.468715 | 2.738641 | -2.051610 |
| C | 2.179911 | 3.200559 | -1.780642 |
| C | 1.196006 | 2.280649 | -1.413124 |
| N | 1.460635 | 0.968720 | -1.326818 |
| O | 4.067009 | -1.475969 | -1.383221 |
| H | 4.721215 | 0.949848 | -2.138505 |
| H | 4.255573 | 3.434217 | -2.331185 |
| H | 0.189270 | 2.583986 | -1.145914 |
| H | 1.930208 | 4.255185 | -1.834822 |
| H | 1.526865 | -3.723051 | -1.501555 |

```
 H              -0.775292   -2.823435   -1.453569
 H              -0.598010   -2.758249    0.352202
 H               1.868067   -4.333743    0.736582
 H               3.129114   -3.115045    0.445333
 H              -0.142248   -1.160620    2.940262
 H               3.759158   -1.169065    1.119029
 H               0.210772    1.229326    3.661990
 H               4.082185    1.174388    1.784363
 H               2.305607    2.414157    3.017558
 C              -4.224680    0.634279    1.258488
 H              -5.265043    0.959711    1.149092
 H              -4.087741    0.255353    2.276904
 H              -3.562941    1.494336    1.130920
 C              -4.788699   -1.695059    0.508323
 H              -4.764151   -1.964321    1.569295
 H              -5.825656   -1.460770    0.239644
 H              -4.465017   -2.564446   -0.068974
 C              -4.663731    1.215225   -1.559011
 H              -4.536985    1.480117   -2.613905
 H              -5.724982    1.001599   -1.383503
 H              -4.369863    2.076369   -0.955215
 C              -4.055485   -1.126195   -2.249384
 H              -5.103471   -1.445127   -2.251318
 H              -3.801794   -0.758739   -3.248768
 H              -3.425354   -1.996723   -2.039035

 Electronic Energy =  -2106.32353478 (Hartree/Particle)
 Dipole Moment (Debye):      1.8400
 index          0
 Harmonic frequencies
 Number of imaginary frequencies=           0

 THERMOCHEMICAL DATA

 Temperature   298.150 Kelvin.  Pressure   1.00000 Atm.
 Zero-point correction=                           0.450115
(Hartree/Particle)
 Thermal correction to Energy=                    0.481459
 Thermal correction to Enthalpy=                  0.482404
 Thermal correction to Gibbs Free Energy=         0.386793
 Sum of electronic and zero-point Energies=          -2105.873420
 Sum of electronic and thermal Energies=             -2105.842075
 Sum of electronic and thermal Enthalpies=           -2105.841131
 Sum of electronic and thermal Free Energies=        -2105.936742
 PCM-SP (solvent=THF;6-311+g(2d,p)) =   -2106.72173924
 Corrected Free Energy =   -2106.33494643

------------------------------------------------------
```

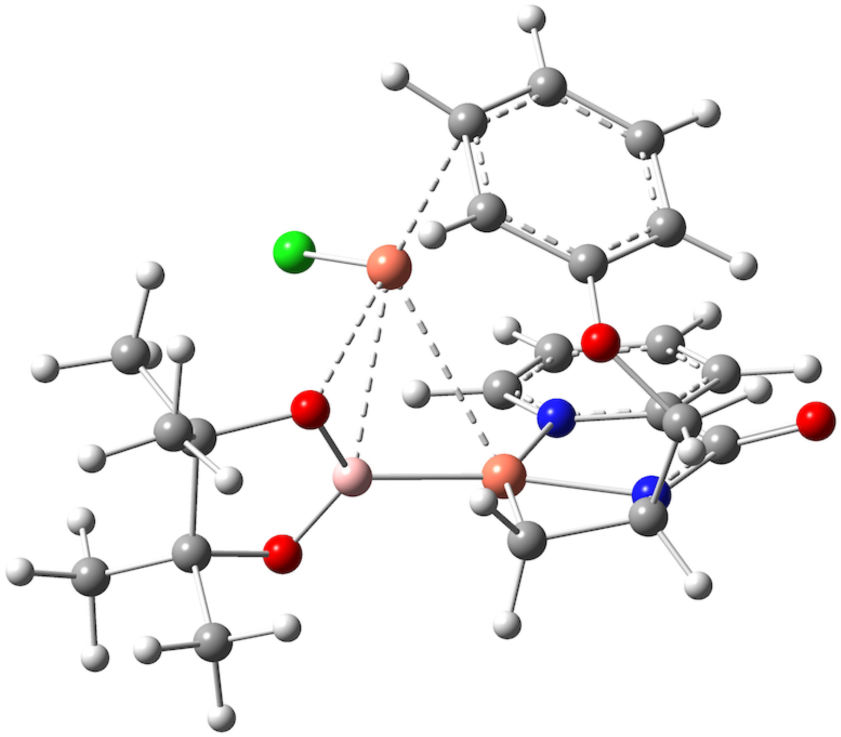

TS4b Substrate: 1c
Path: anti-Markovnikov

CARTESIAN COORDINATES

| | | | |
|---|---|---|---|
| C | -1.974853 | -1.635708 | 2.888312 |
| C | -0.654161 | -1.179125 | 3.077791 |
| C | -0.295134 | 0.136154 | 2.698227 |
| C | -1.288613 | 1.009766 | 2.173894 |
| C | -2.593421 | 0.549897 | 1.993873 |
| C | -2.918660 | -0.770095 | 2.346047 |
| O | -0.859899 | 2.280692 | 1.958525 |
| C | -1.557986 | 3.205639 | 1.104072 |
| C | -1.173619 | 3.039641 | -0.376918 |
| Cu | -0.255169 | 0.771107 | -1.036235 |
| B | 1.643732 | 0.335200 | -0.726511 |
| O | 2.232533 | 0.324009 | 0.528693 |
| C | 3.669639 | 0.039738 | 0.360731 |
| C | 3.893832 | 0.378603 | -1.163703 |
| O | 2.553772 | 0.219678 | -1.737527 |
| Cu | 0.410445 | -1.205092 | 1.112692 |
| Cl | 0.813709 | -3.112659 | 0.130510 |
| C | 0.306489 | 2.616331 | -0.564946 |
| N | -1.768220 | 1.892287 | -1.043154 |
| C | -2.988665 | 1.360848 | -1.017845 |
| C | -2.900095 | -0.089403 | -1.491126 |
| C | -4.037393 | -0.827835 | -1.794905 |
| C | -3.889921 | -2.152319 | -2.216853 |
| C | -2.608985 | -2.695856 | -2.311761 |
| C | -1.511818 | -1.902121 | -1.970227 |
| N | -1.657029 | -0.632202 | -1.567159 |
| O | -4.066783 | 1.856549 | -0.652186 |
| H | -5.004970 | -0.347362 | -1.692126 |
| H | -4.762802 | -2.750389 | -2.465188 |
| H | -0.500378 | -2.288363 | -1.976912 |

| | | | |
|---|---|---|---|
| H | -2.447168 | -3.721694 | -2.626266 |
| H | -1.417925 | 3.990664 | -0.878139 |
| H | 0.773386 | 3.028502 | -1.461826 |
| H | 0.946915 | 2.686791 | 0.311557 |
| H | -1.222419 | 4.184781 | 1.457196 |
| H | -2.642402 | 3.138728 | 1.228316 |
| H | 0.667728 | 0.564437 | 2.966227 |
| H | -3.361745 | 1.192399 | 1.582261 |
| H | 0.061978 | -1.801874 | 3.609540 |
| H | -3.939274 | -1.109748 | 2.190531 |
| H | -2.239456 | -2.652204 | 3.159700 |
| C | 3.891615 | -1.431635 | 0.708301 |
| H | 4.956153 | -1.686219 | 0.662406 |
| H | 3.535823 | -1.615114 | 1.727609 |
| H | 3.328987 | -2.094881 | 0.049675 |
| C | 4.426955 | 0.935100 | 1.337762 |
| H | 4.190121 | 0.636982 | 2.364477 |
| H | 5.508888 | 0.829999 | 1.194784 |
| H | 4.158232 | 1.987559 | 1.220440 |
| C | 4.841108 | -0.565594 | -1.896467 |
| H | 4.915913 | -0.265576 | -2.946742 |
| H | 5.844042 | -0.524396 | -1.455090 |
| H | 4.483432 | -1.596732 | -1.862668 |
| C | 4.290032 | 1.838137 | -1.416524 |
| H | 5.302660 | 2.048645 | -1.055898 |
| H | 4.256737 | 2.032124 | -2.493201 |
| H | 3.594477 | 2.528694 | -0.928278 |

Electronic Energy = -2106.32190725 (Hartree/Particle)
Dipole Moment (Debye): 2.5374
index 0
Harmonic frequencies
Number of imaginary frequencies= 1
Negatives Eigenvalues: -36.673

THERMOCHEMICAL DATA

Temperature 298.150 Kelvin. Pressure 1.00000 Atm.
Zero-point correction= 0.450034 (Hartree/Particle)
Thermal correction to Energy= 0.480564
Thermal correction to Enthalpy= 0.481508
Thermal correction to Gibbs Free Energy= 0.387797
Sum of electronic and zero-point Energies= -2105.871874
Sum of electronic and thermal Energies= -2105.841344
Sum of electronic and thermal Enthalpies= -2105.840399
Sum of electronic and thermal Free Energies= -2105.934110
PCM-SP (solvent=THF;6-311+g(2d,p)) = -2106.72076964
Corrected Free Energy = -2106.33297239

---

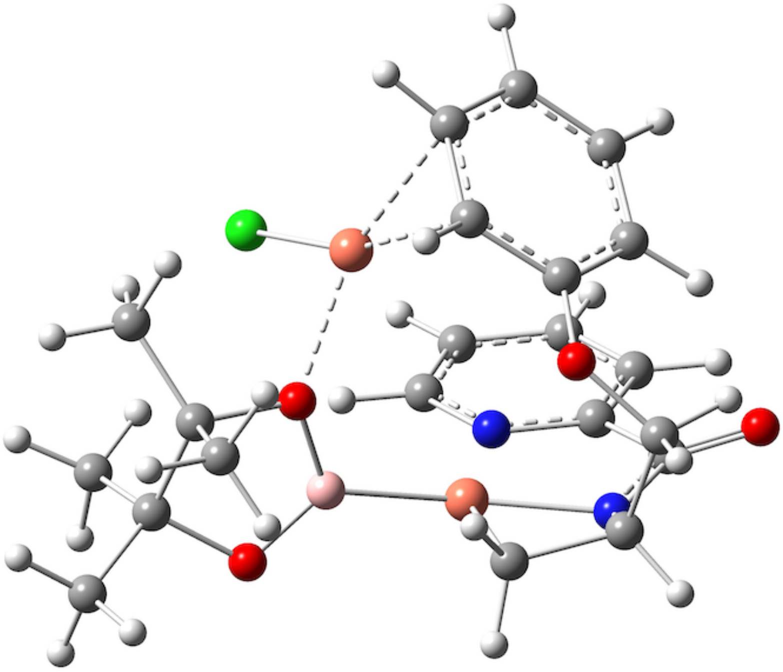

I3b Substrate: 1c
Path: anti-Markovnikov

CARTESIAN COORDINATES

| | | | |
|---|---|---|---|
| C | 1.754428 | 2.540452 | 2.416257 |
| C | 0.489977 | 2.008294 | 2.754929 |
| C | 0.259486 | 0.613870 | 2.669706 |
| C | 1.321433 | -0.252122 | 2.275870 |
| C | 2.564270 | 0.285030 | 1.944938 |
| C | 2.763648 | 1.676104 | 2.014787 |
| O | 1.005827 | -1.572582 | 2.323559 |
| C | 1.885195 | -2.620910 | 1.872592 |
| C | 1.579296 | -3.058589 | 0.428387 |
| Cu | 0.356585 | -1.344391 | -0.942199 |
| B | -1.539943 | -0.954695 | -0.704466 |
| O | -1.946179 | -0.235116 | 0.413975 |
| C | -3.421785 | -0.295710 | 0.471656 |
| C | -3.782832 | -0.600131 | -1.024766 |
| O | -2.589106 | -1.320377 | -1.495054 |
| Cu | -0.616164 | 1.538132 | 0.912203 |
| Cl | -1.145271 | 3.028486 | -0.603153 |
| C | 0.062960 | -2.992337 | 0.101135 |
| N | 2.028692 | -2.128220 | -0.601764 |
| C | 3.123952 | -1.376596 | -0.713839 |
| C | 2.797856 | -0.137759 | -1.551828 |
| C | 3.788454 | 0.729373 | -1.995419 |
| C | 3.415114 | 1.887237 | -2.684867 |
| C | 2.061487 | 2.147255 | -2.896521 |
| C | 1.118418 | 1.236940 | -2.416322 |
| N | 1.480564 | 0.122249 | -1.766047 |
| O | 4.248187 | -1.526844 | -0.208919 |
| H | 4.822551 | 0.483596 | -1.776785 |
| H | 4.171290 | 2.582108 | -3.040530 |
| H | 0.054803 | 1.413653 | -2.514224 |
| H | 1.724215 | 3.046480 | -3.400954 |
| H | 2.005224 | -4.067867 | 0.306205 |

| | | | |
|---|---|---|---|
| H | -0.269346 | -3.751690 | -0.609561 |
| H | -0.610145 | -2.899093 | 0.951842 |
| H | 1.670097 | -3.453602 | 2.549243 |
| H | 2.937506 | -2.344759 | 1.977364 |
| H | -0.635170 | 0.158638 | 3.087742 |
| H | 3.386393 | -0.347493 | 1.635982 |
| H | -0.262791 | 2.652556 | 3.205114 |
| H | 3.740686 | 2.069915 | 1.747454 |
| H | 1.921271 | 3.611150 | 2.468514 |
| C | -3.944020 | 1.030868 | 1.007871 |
| H | -5.039537 | 1.050566 | 0.969353 |
| H | -3.640384 | 1.150689 | 2.054250 |
| H | -3.547612 | 1.876718 | 0.443023 |
| C | -3.751252 | -1.443122 | 1.430838 |
| H | -3.284420 | -1.242290 | 2.400582 |
| H | -4.832180 | -1.537971 | 1.579814 |
| H | -3.364226 | -2.397182 | 1.058587 |
| C | -3.903395 | 0.660949 | -1.884760 |
| H | -3.941013 | 0.365606 | -2.938342 |
| H | -4.818994 | 1.213769 | -1.647091 |
| H | -3.049019 | 1.328602 | -1.738178 |
| C | -4.992209 | -1.508205 | -1.218231 |
| H | -5.890609 | -1.043107 | -0.795113 |
| H | -5.162046 | -1.668184 | -2.287731 |
| H | -4.846960 | -2.484267 | -0.749007 |

Electronic Energy = -2106.32816499 (Hartree/Particle)
Dipole Moment (Debye): 2.6979
index 0
Harmonic frequencies
Number of imaginary frequencies= 0

THERMOCHEMICAL DATA

Temperature 298.150 Kelvin. Pressure 1.00000 Atm.
Zero-point correction= 0.450490
(Hartree/Particle)
Thermal correction to Energy= 0.481544
Thermal correction to Enthalpy= 0.482488
Thermal correction to Gibbs Free Energy= 0.388415
Sum of electronic and zero-point Energies= -2105.877675
Sum of electronic and thermal Energies= -2105.846621
Sum of electronic and thermal Enthalpies= -2105.845677
Sum of electronic and thermal Free Energies= -2105.939750
PCM-SP (solvent=THF;6-311+g(2d,p)) = -2106.72721471
Corrected Free Energy = -2106.33879995

-------------------------------------------------------

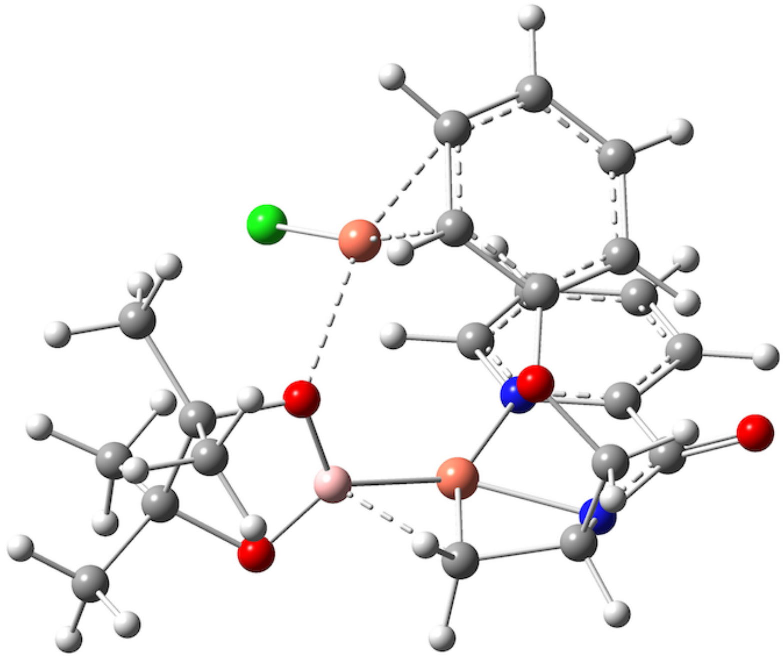

TS5 Substrate: 1c
Path: anti-Markovnikov

CARTESIAN COORDINATES

| | | | |
|---|---|---|---|
| C | 1.827383 | 2.705139 | 2.263997 |
| C | 0.464807 | 2.420218 | 2.492870 |
| C | 0.011318 | 1.079558 | 2.513008 |
| C | 0.942024 | 0.017735 | 2.317520 |
| C | 2.289352 | 0.310892 | 2.103049 |
| C | 2.713366 | 1.650439 | 2.076926 |
| O | 0.394947 | -1.219992 | 2.411009 |
| C | 1.124655 | -2.440841 | 2.174690 |
| C | 0.927827 | -2.981615 | 0.742473 |
| Cu | 0.321747 | -1.245030 | -0.933674 |
| B | -1.585258 | -1.072687 | -0.471139 |
| O | -1.984959 | -0.108835 | 0.438447 |
| C | -3.457610 | -0.038173 | 0.379985 |
| C | -3.752363 | -0.623664 | -1.048845 |
| O | -2.613048 | -1.527800 | -1.252520 |
| Cu | -0.494460 | 1.788107 | 0.566962 |
| Cl | -0.873969 | 2.849401 | -1.286681 |
| C | -0.516976 | -2.686208 | 0.195337 |
| N | 1.755564 | -2.374141 | -0.291773 |
| C | 2.949485 | -1.803390 | -0.208164 |
| C | 3.028158 | -0.634152 | -1.205054 |
| C | 4.247694 | -0.065834 | -1.560709 |
| C | 4.260945 | 1.053577 | -2.395945 |
| C | 3.048615 | 1.589539 | -2.832745 |
| C | 1.859885 | 0.983867 | -2.424083 |
| N | 1.849957 | -0.103777 | -1.637278 |
| O | 3.905775 | -2.009775 | 0.566032 |
| H | 5.154474 | -0.508768 | -1.162170 |
| H | 5.203189 | 1.507427 | -2.692221 |
| H | 0.890359 | 1.389625 | -2.688413 |
| H | 3.009747 | 2.471557 | -3.463854 |
| H | 1.073425 | -4.073579 | 0.807091 |

| | | | |
|---|---|---|---|
| H | -0.828551 | -3.453147 | -0.518225 |
| H | -1.255957 | -2.610644 | 0.996913 |
| H | 0.681506 | -3.145610 | 2.884566 |
| H | 2.188266 | -2.334546 | 2.399794 |
| H | -0.986103 | 0.820868 | 2.859124 |
| H | 3.019221 | -0.473932 | 1.951586 |
| H | -0.218188 | 3.221634 | 2.765748 |
| H | 3.766956 | 1.851933 | 1.902124 |
| H | 2.169232 | 3.734619 | 2.238697 |
| C | -3.882213 | 1.410382 | 0.583758 |
| H | -4.965470 | 1.510905 | 0.448438 |
| H | -3.638249 | 1.726748 | 1.604376 |
| H | -3.369422 | 2.079720 | -0.109707 |
| C | -3.964429 | -0.923327 | 1.522601 |
| H | -3.543206 | -0.563798 | 2.466882 |
| H | -5.056742 | -0.890290 | 1.594931 |
| H | -3.658259 | -1.965998 | 1.388898 |
| C | -3.688490 | 0.428348 | -2.159146 |
| H | -3.693077 | -0.082454 | -3.127483 |
| H | -4.554255 | 1.098294 | -2.116711 |
| H | -2.779579 | 1.032507 | -2.087575 |
| C | -5.033696 | -1.444133 | -1.150788 |
| H | -5.904262 | -0.822399 | -0.910405 |
| H | -5.150813 | -1.813950 | -2.174374 |
| H | -5.020597 | -2.305849 | -0.478920 |

Electronic Energy = -2106.32405558 (Hartree/Particle)
Dipole Moment (Debye): 3.1867
index 0
Harmonic frequencies
Number of imaginary frequencies= 1
Negatives Eigenvalues: -115.830

THERMOCHEMICAL DATA

Temperature 298.150 Kelvin. Pressure 1.00000 Atm.
Zero-point correction= 0.449998
(Hartree/Particle)
Thermal correction to Energy= 0.480427
Thermal correction to Enthalpy= 0.481371
Thermal correction to Gibbs Free Energy= 0.389242
Sum of electronic and zero-point Energies= -2105.874058
Sum of electronic and thermal Energies= -2105.843629
Sum of electronic and thermal Enthalpies= -2105.842685
Sum of electronic and thermal Free Energies= -2105.934814
PCM-SP (solvent=THF;6-311+g(2d,p)) = -2106.72383744
Corrected Free Energy = -2106.33459586

-------------------------------------------------------

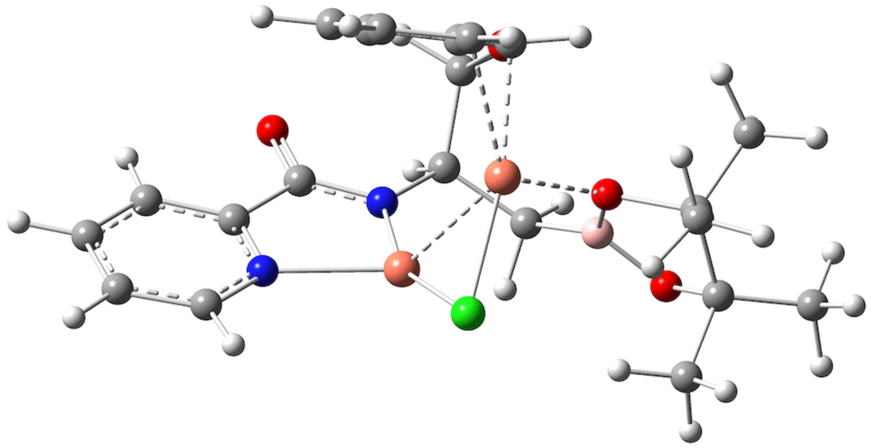

P2 Substrate: 1c
Path: anti-Markovnikov

CARTESIAN COORDINATES

| | | | |
|---|---|---|---|
| C | 1.483707 | 3.382353 | -1.148678 |
| C | 0.086295 | 3.321469 | -1.072899 |
| C | -0.547765 | 2.732736 | 0.047943 |
| C | 0.242956 | 2.171721 | 1.091331 |
| C | 1.642399 | 2.255121 | 1.011962 |
| C | 2.241094 | 2.862351 | -0.094962 |
| O | -0.457786 | 1.624229 | 2.106575 |
| C | 0.160079 | 0.678326 | 3.006171 |
| C | 0.293310 | -0.752204 | 2.450505 |
| Cu | 0.829147 | -1.040192 | -0.576212 |
| B | -2.154864 | -1.000968 | 1.149092 |
| O | -2.247122 | 0.107197 | 0.312047 |
| C | -3.642163 | 0.190348 | -0.153850 |
| C | -4.095409 | -1.303924 | -0.047703 |
| O | -3.286501 | -1.779233 | 1.063118 |
| Cu | -0.599910 | 0.950224 | -1.058064 |
| Cl | -0.242868 | -0.723498 | -2.525857 |
| C | -1.045360 | -1.454650 | 2.171870 |
| N | 1.211877 | -0.872004 | 1.315155 |
| C | 2.513125 | -0.777159 | 1.630550 |
| C | 3.484473 | -0.961462 | 0.474920 |
| C | 4.861855 | -0.870677 | 0.698019 |
| C | 5.729625 | -1.023020 | -0.383858 |
| C | 5.198662 | -1.254038 | -1.656265 |
| C | 3.811162 | -1.326170 | -1.791461 |
| N | 2.979335 | -1.188514 | -0.751906 |
| O | 2.992745 | -0.523103 | 2.759702 |
| H | 5.211395 | -0.683380 | 1.707251 |
| H | 6.805176 | -0.960543 | -0.238656 |
| H | 3.338815 | -1.496662 | -2.756095 |
| H | 5.839223 | -1.375348 | -2.524844 |
| H | 0.754060 | -1.284440 | 3.298776 |
| H | -0.810631 | -2.494136 | 1.899992 |
| H | -1.586432 | -1.546714 | 3.128850 |
| H | -0.512788 | 0.667773 | 3.868644 |
| H | 1.137525 | 1.036607 | 3.336277 |
| H | -1.615603 | 2.845956 | 0.218558 |
| H | 2.264456 | 1.827621 | 1.788771 |
| H | -0.527060 | 3.773047 | -1.847650 |

| | | | |
|---|---|---|---|
| H | 3.326295 | 2.906001 | -0.137821 |
| H | 1.966554 | 3.825331 | -2.013817 |
| C | -3.671689 | 0.771002 | -1.562967 |
| H | -4.696547 | 0.759266 | -1.951610 |
| H | -3.332130 | 1.813267 | -1.547560 |
| H | -3.031360 | 0.211848 | -2.248011 |
| C | -4.364049 | 1.121465 | 0.826001 |
| H | -3.852114 | 2.088597 | 0.847467 |
| H | -5.403584 | 1.286128 | 0.523322 |
| H | -4.354904 | 0.713783 | 1.841580 |
| C | -3.706986 | -2.137233 | -1.274938 |
| H | -3.847852 | -3.196509 | -1.037752 |
| H | -4.332429 | -1.889932 | -2.139843 |
| H | -2.658429 | -1.981342 | -1.547046 |
| C | -5.568154 | -1.502121 | 0.296575 |
| H | -6.206914 | -1.059257 | -0.476944 |
| H | -5.790385 | -2.572673 | 0.351214 |
| H | -5.821305 | -1.055409 | 1.261207 |

Electronic Energy = -2106.41494402 (Hartree/Particle)
Dipole Moment (Debye): 4.5968
index 0
Harmonic frequencies
Number of imaginary frequencies= 0

THERMOCHEMICAL DATA

Temperature 298.150 Kelvin. Pressure 1.00000 Atm.
Zero-point correction= 0.452033
(Hartree/Particle)
Thermal correction to Energy= 0.482922
Thermal correction to Enthalpy= 0.483866
Thermal correction to Gibbs Free Energy= 0.390290
Sum of electronic and zero-point Energies= -2105.962911
Sum of electronic and thermal Energies= -2105.932022
Sum of electronic and thermal Enthalpies= -2105.931078
Sum of electronic and thermal Free Energies= -2106.024654
PCM-SP (solvent=THF;6-311+g(2d,p)) = -2106.81097231
Corrected Free Energy = -2106.42068229

---

PATH: MARKOVNIKOV

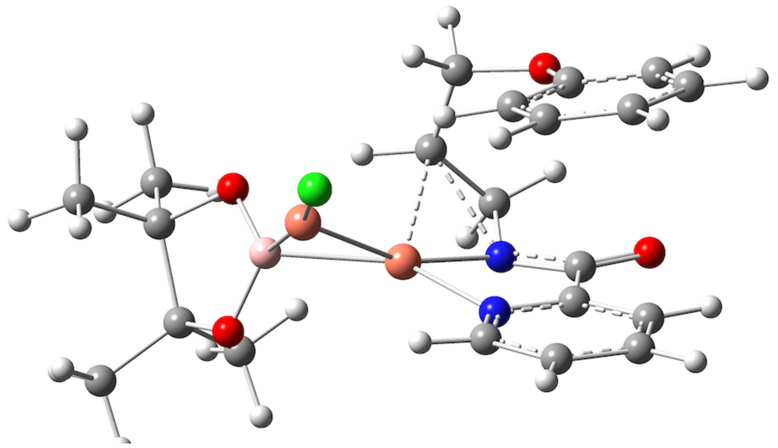

TS3 Substrate: 1c
Path: Markovnikov

CARTESIAN COORDINATES

| | | | |
|---|---|---|---|
| C | -0.299954 | -2.133977 | -0.903735 |
| N | 0.505324 | -2.010048 | 0.797575 |
| C | 1.846446 | -1.927262 | 1.194917 |
| O | 2.590148 | -2.897131 | 1.256590 |
| C | 0.119273 | -3.189824 | 0.042762 |
| C | 2.286174 | -0.561469 | 1.647054 |
| C | 3.573990 | -0.358022 | 2.136036 |
| C | 3.940959 | 0.935722 | 2.512240 |
| C | 3.019513 | 1.973200 | 2.370023 |
| C | 1.754605 | 1.684792 | 1.852050 |
| N | 1.398629 | 0.440133 | 1.515330 |
| Cu | -0.853872 | 1.806031 | -0.488126 |
| H | 4.256808 | -1.198063 | 2.192231 |
| H | 4.939124 | 1.132201 | 2.893344 |
| H | 1.016169 | 2.458557 | 1.667193 |
| H | 3.271381 | 2.995202 | 2.634664 |
| H | 0.965987 | -3.806960 | -0.261248 |
| H | -0.678178 | -3.772059 | 0.510047 |
| Cu | -0.300795 | -0.224979 | 0.498804 |
| B | -2.286793 | 0.418879 | 0.002206 |
| O | -2.954982 | -0.296066 | -0.992306 |
| O | -3.108434 | 0.615078 | 1.100267 |
| C | -4.367932 | -0.367920 | -0.630241 |
| C | -4.309996 | -0.193431 | 0.927324 |
| Cl | 0.328855 | 3.505234 | -1.087284 |
| H | -1.355090 | -1.876269 | -0.970107 |
| C | 0.506557 | -1.775499 | -2.120919 |
| H | 0.150643 | -2.460237 | -2.909203 |
| H | 0.280354 | -0.759938 | -2.455587 |
| O | 1.888525 | -2.023258 | -1.984725 |
| C | 2.763000 | -0.986923 | -1.705146 |
| C | 2.403725 | 0.358664 | -1.595944 |
| C | 4.094176 | -1.381518 | -1.520397 |
| C | 3.386896 | 1.312045 | -1.307936 |
| H | 1.381499 | 0.703522 | -1.701163 |
| C | 5.063870 | -0.422375 | -1.235396 |
| H | 4.340343 | -2.436381 | -1.589952 |
| C | 4.716718 | 0.931344 | -1.128985 |
| H | 3.076124 | 2.349204 | -1.218146 |
| H | 6.095076 | -0.735568 | -1.091914 |
| H | 5.475109 | 1.676415 | -0.904362 |
| C | -4.057282 | -1.509015 | 1.676643 |
| H | -3.822598 | -1.281918 | 2.721155 |
| H | -4.934570 | -2.164660 | 1.649817 |
| H | -3.202771 | -2.046141 | 1.250741 |
| C | -5.494292 | 0.547725 | 1.539571 |
| H | -6.428522 | 0.006103 | 1.347598 |
| H | -5.360030 | 0.626477 | 2.623358 |
| H | -5.585346 | 1.559218 | 1.137424 |
| C | -5.066105 | 0.794131 | -1.347219 |
| H | -4.884134 | 0.708211 | -2.423144 |
| H | -6.147842 | 0.779764 | -1.174425 |
| H | -4.668756 | 1.757835 | -1.013860 |
| C | -4.927489 | -1.703125 | -1.111884 |
| H | -5.964622 | -1.828874 | -0.778783 |
| H | -4.914462 | -1.736171 | -2.206474 |
| H | -4.339774 | -2.546510 | -0.739907 |

Electronic Energy = -2106.31037510 (Hartree/Particle)
Dipole Moment (Debye): 5.6783
index 0
Harmonic frequencies
Number of imaginary frequencies= 1
Negatives Eigenvalues: -222.555

THERMOCHEMICAL DATA

Temperature 298.150 Kelvin. Pressure 1.00000 Atm.
Zero-point correction= 0.448491
(Hartree/Particle)
Thermal correction to Energy= 0.480080
Thermal correction to Enthalpy= 0.481024
Thermal correction to Gibbs Free Energy= 0.383276
Sum of electronic and zero-point Energies= -2105.861884
Sum of electronic and thermal Energies= -2105.830295
Sum of electronic and thermal Enthalpies= -2105.829351
Sum of electronic and thermal Free Energies= -2105.927099
PCM-SP (solvent=THF;6-311+g(2d,p)) = -2106.71119542
Corrected Free Energy = -2106.3279193

-------------------------------------------------------

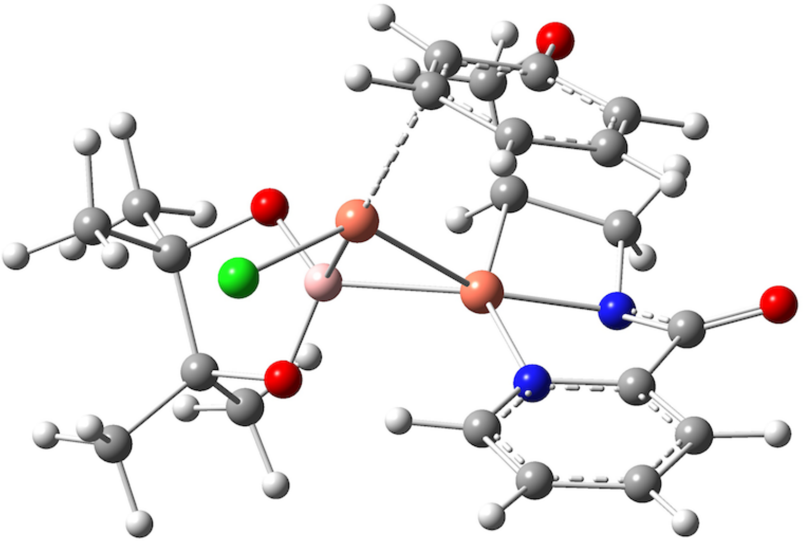

I2 Substrate: 1c
Path: Markovnikov

CARTESIAN COORDINATES

| | | | |
|---|---|---|---|
| C | -0.057418 | 2.570992 | 0.108653 |
| N | 1.571234 | 1.948037 | -1.305556 |
| C | 2.820716 | 1.454063 | -1.405226 |
| O | 3.894834 | 2.053311 | -1.287525 |
| C | 1.182839 | 3.157450 | -0.586313 |
| C | 2.773586 | -0.045025 | -1.677522 |
| C | 3.885741 | -0.750732 | -2.122126 |
| C | 3.772507 | -2.130266 | -2.310966 |
| C | 2.557308 | -2.760342 | -2.035000 |
| C | 1.488624 | -1.991053 | -1.571418 |
| N | 1.602749 | -0.664901 | -1.403404 |

| | | | |
|---|---|---|---|
| Cu | -0.359946 | -1.014285 | 0.968246 |
| H | 4.808386 | -0.208028 | -2.297597 |
| H | 4.623906 | -2.705900 | -2.662962 |
| H | 0.533115 | -2.429834 | -1.300477 |
| H | 2.432438 | -3.831190 | -2.159423 |
| H | 1.952722 | 3.485566 | 0.120944 |
| H | 0.948792 | 3.988366 | -1.264598 |
| Cu | 0.318941 | 0.730882 | -0.612939 |
| B | -1.638840 | 0.122467 | -0.245235 |
| O | -2.562874 | 0.710847 | 0.595742 |
| O | -2.197208 | -0.367266 | -1.398998 |
| C | -3.891275 | 0.313646 | 0.112551 |
| C | -3.609916 | 0.012528 | -1.407845 |
| Cl | -1.088680 | -3.084695 | 0.905591 |
| H | -1.013835 | 2.855380 | -0.333353 |
| C | -0.126923 | 2.635171 | 1.619910 |
| H | -0.333626 | 3.670328 | 1.922057 |
| H | -0.935588 | 2.000568 | 1.993088 |
| O | 1.126199 | 2.340094 | 2.265144 |
| C | 1.539717 | 1.032027 | 2.277203 |
| C | 2.803608 | 0.737344 | 1.766100 |
| C | 0.735607 | 0.006662 | 2.821969 |
| C | 3.263253 | -0.585393 | 1.769994 |
| H | 3.401169 | 1.536872 | 1.340628 |
| C | 1.217176 | -1.317127 | 2.833636 |
| H | -0.198766 | 0.258648 | 3.317955 |
| C | 2.477280 | -1.615025 | 2.288688 |
| H | 4.236440 | -0.804321 | 1.339847 |
| H | 0.617278 | -2.101071 | 3.284962 |
| H | 2.825163 | -2.643263 | 2.269858 |
| C | -3.720580 | 1.246901 | -2.310310 |
| H | -3.327770 | 0.998937 | -3.301743 |
| H | -4.761606 | 1.569188 | -2.419635 |
| H | -3.134558 | 2.083363 | -1.914327 |
| C | -4.410704 | -1.150007 | -1.984621 |
| H | -5.485486 | -0.942072 | -1.921312 |
| H | -4.148451 | -1.291523 | -3.038217 |
| H | -4.197498 | -2.081012 | -1.454904 |
| C | -4.859730 | 1.463844 | 0.366357 |
| H | -5.835990 | 1.239652 | -0.079251 |
| H | -5.001517 | 1.602082 | 1.443413 |
| H | -4.491940 | 2.404210 | -0.051025 |
| C | -4.297427 | -0.922388 | 0.921121 |
| H | -4.309126 | -0.657632 | 1.983876 |
| H | -5.298171 | -1.268577 | 0.641358 |
| H | -3.584009 | -1.740022 | 0.786862 |

Electronic Energy = -2106.32541596 (Hartree/Particle)
Dipole Moment (Debye): 2.1292
index 0
Harmonic frequencies
Number of imaginary frequencies= 0

THERMOCHEMICAL DATA

Temperature 298.150 Kelvin. Pressure 1.00000 Atm.
Zero-point correction= 0.450402 (Hartree/Particle)
Thermal correction to Energy= 0.481773
Thermal correction to Enthalpy= 0.482717

Thermal correction to Gibbs Free Energy= 0.387207
Sum of electronic and zero-point Energies= -2105.875014
Sum of electronic and thermal Energies= -2105.843643
Sum of electronic and thermal Enthalpies= -2105.842699
Sum of electronic and thermal Free Energies= -2105.938209
PCM-SP (solvent=THF;6-311+g(2d,p)) = -2106.72471889
Corrected Free Energy = -2106.33751193

-------------------------------------------------------

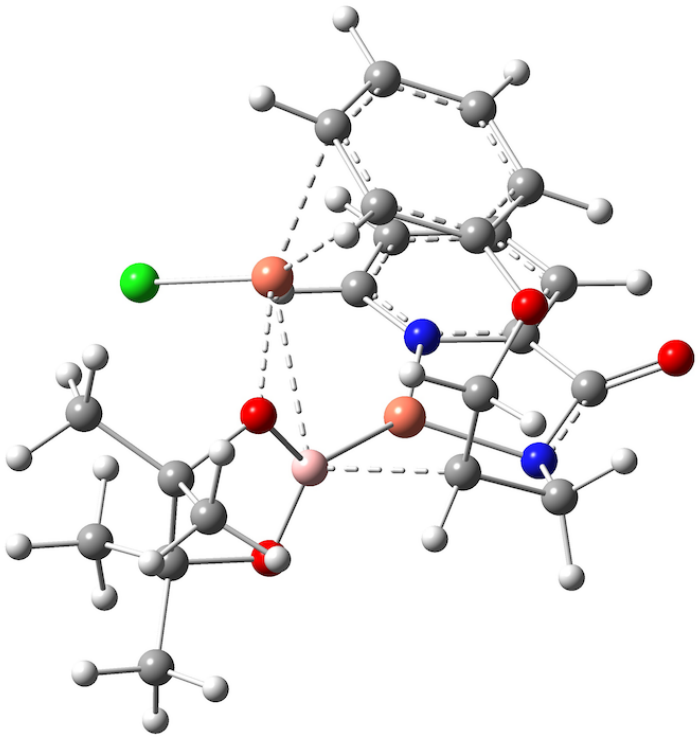

TS4=TS5 Substrate: 1c
Path: Markovnikov

CARTESIAN COORDINATES

| | | | |
|---|---|---|---|
| C | -0.489118 | 2.369361 | 0.359369 |
| N | 1.484755 | 2.314936 | -1.004360 |
| C | 2.752835 | 1.988157 | -0.748233 |
| O | 3.649341 | 2.600000 | -0.141840 |
| C | 0.691116 | 3.241829 | -0.214445 |
| C | 3.027961 | 0.571130 | -1.270638 |
| C | 4.315673 | 0.141887 | -1.580054 |
| C | 4.508268 | -1.172390 | -2.009566 |
| C | 3.407150 | -2.027129 | -2.095285 |
| C | 2.148229 | -1.541479 | -1.740944 |
| N | 1.965666 | -0.273000 | -1.340373 |
| Cu | -0.378771 | -1.434918 | 0.626164 |
| H | 5.132527 | 0.847587 | -1.470317 |
| H | 5.502663 | -1.526051 | -2.270020 |
| H | 1.263170 | -2.169534 | -1.749382 |
| H | 3.511073 | -3.058471 | -2.417449 |
| H | 1.258439 | 3.697927 | 0.608589 |
| H | 0.286074 | 4.040688 | -0.846966 |
| Cu | 0.320256 | 0.792853 | -0.704347 |
| B | -1.622878 | 0.746999 | -0.340274 |

| | | | |
|---|---|---|---|
| O | -2.289373 | 0.115562 | 0.687472 |
| O | -2.418016 | 1.041838 | -1.409794 |
| C | -3.713757 | 0.020914 | 0.314745 |
| C | -3.693660 | 0.331938 | -1.239394 |
| Cl | -0.928354 | -3.044566 | -0.739170 |
| H | -1.452883 | 2.830369 | 0.119779 |
| C | -0.416264 | 2.205897 | 1.878280 |
| H | -0.535966 | 3.197480 | 2.329153 |
| H | -1.212954 | 1.561229 | 2.255836 |
| O | 0.871516 | 1.777137 | 2.339867 |
| C | 1.217768 | 0.459166 | 2.270308 |
| C | 2.557506 | 0.177824 | 2.002885 |
| C | 0.302588 | -0.596180 | 2.527433 |
| C | 3.001739 | -1.149939 | 1.986120 |
| H | 3.227823 | 1.004174 | 1.787364 |
| C | 0.778672 | -1.932399 | 2.524639 |
| H | -0.688843 | -0.373252 | 2.912080 |
| C | 2.128611 | -2.206330 | 2.239475 |
| H | 4.045298 | -1.350957 | 1.759457 |
| H | 0.118699 | -2.736595 | 2.841133 |
| H | 2.477655 | -3.233731 | 2.222237 |
| C | -4.806231 | 1.263267 | -1.714662 |
| H | -4.689760 | 1.446717 | -2.787206 |
| H | -5.787136 | 0.801662 | -1.551690 |
| H | -4.782834 | 2.228472 | -1.203030 |
| C | -3.628404 | -0.912986 | -2.125571 |
| H | -4.567117 | -1.475988 | -2.081941 |
| H | -3.463257 | -0.596675 | -3.160530 |
| H | -2.808201 | -1.573212 | -1.835801 |
| C | -4.424448 | 1.085826 | 1.153778 |
| H | -5.503614 | 1.084385 | 0.968628 |
| H | -4.256907 | 0.875409 | 2.215073 |
| H | -4.037895 | 2.087964 | 0.940556 |
| C | -4.192653 | -1.378458 | 0.682042 |
| H | -4.156081 | -1.504800 | 1.769580 |
| H | -5.228655 | -1.526826 | 0.356552 |
| H | -3.560143 | -2.144723 | 0.228670 |

Electronic Energy = -2106.31540616 (Hartree/Particle)
Dipole Moment (Debye): 4.4348
index 0
Harmonic frequencies
Number of imaginary frequencies= 1
Negatives Eigenvalues: -148.079

THERMOCHEMICAL DATA

Temperature 298.150 Kelvin. Pressure 1.00000 Atm.
Zero-point correction= 0.449959
(Hartree/Particle)
Thermal correction to Energy= 0.480696
Thermal correction to Enthalpy= 0.481640
Thermal correction to Gibbs Free Energy= 0.388353
Sum of electronic and zero-point Energies= -2105.865447
Sum of electronic and thermal Energies= -2105.834710
Sum of electronic and thermal Enthalpies= -2105.833766
Sum of electronic and thermal Free Energies= -2105.927053
PCM-SP (solvent=THF;6-311+g(2d,p)) = -2106.71773050
Corrected Free Energy = -2106.32937735

-------------------------------------------------------

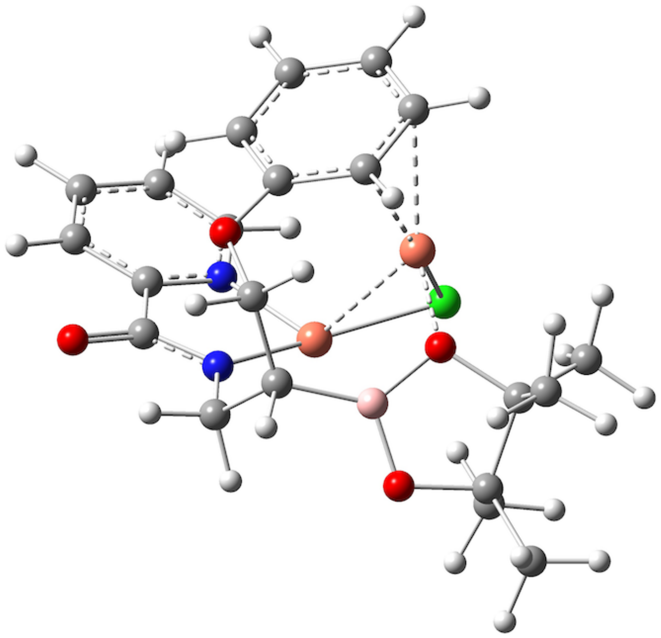

P2 Substrate: 1c
Path: Markovnikov

CARTESIAN COORDINATES

| | | | |
|---|---|---|---|
| C | 1.072742 | -1.770882 | 1.701063 |
| N | -0.940251 | -1.857041 | 0.164318 |
| C | -2.235096 | -2.099564 | 0.446090 |
| O | -2.695818 | -2.900337 | 1.285298 |
| C | 0.027140 | -2.629699 | 0.936395 |
| C | -3.239210 | -1.269419 | -0.345865 |
| C | -4.604080 | -1.359113 | -0.052577 |
| C | -5.498297 | -0.555947 | -0.760907 |
| C | -5.007411 | 0.312522 | -1.741259 |
| C | -3.631597 | 0.334201 | -1.975340 |
| N | -2.776615 | -0.439757 | -1.297414 |
| Cu | 0.579201 | 1.445785 | -0.100120 |
| H | -4.921399 | -2.049181 | 0.721426 |
| H | -6.564099 | -0.603774 | -0.551474 |
| H | -3.185993 | 0.989787 | -2.720346 |
| H | -5.670388 | 0.955144 | -2.313387 |
| H | -0.508770 | -3.261430 | 1.653840 |
| H | 0.582167 | -3.292953 | 0.259516 |
| Cu | -0.612655 | -0.495797 | -1.150102 |
| B | 2.126607 | -1.095298 | 0.743675 |
| O | 2.411161 | 0.261094 | 0.577951 |
| O | 3.003599 | -1.882216 | 0.047360 |
| C | 3.725866 | 0.353295 | -0.090319 |
| C | 3.814418 | -1.035729 | -0.817896 |
| Cl | 0.180208 | 1.274535 | -2.305330 |
| H | 1.712482 | -2.523346 | 2.198415 |
| C | 0.483178 | -0.981160 | 2.889972 |
| H | 0.230323 | -1.687102 | 3.685299 |
| H | 1.209717 | -0.266652 | 3.298508 |
| O | -0.777460 | -0.334769 | 2.651956 |
| C | -0.896286 | 0.894393 | 2.119627 |

| | | | |
|---|---|---|---|
| C | -2.199825 | 1.273792 | 1.757026 |
| C | 0.171006 | 1.815750 | 1.948765 |
| C | -2.451140 | 2.542257 | 1.237365 |
| H | -2.994826 | 0.547786 | 1.888915 |
| C | -0.108502 | 3.099610 | 1.404986 |
| H | 1.150535 | 1.609553 | 2.366724 |
| C | -1.414653 | 3.463178 | 1.049463 |
| H | -3.468006 | 2.805844 | 0.959697 |
| H | 0.697086 | 3.827026 | 1.340710 |
| H | -1.611434 | 4.444236 | 0.629346 |
| C | 5.220816 | -1.623534 | -0.890682 |
| H | 5.185663 | -2.590429 | -1.402387 |
| H | 5.884558 | -0.960380 | -1.457994 |
| H | 5.646950 | -1.783044 | 0.102794 |
| C | 3.153177 | -1.047868 | -2.198699 |
| H | 3.734914 | -0.471578 | -2.926169 |
| H | 3.088600 | -2.083628 | -2.546510 |
| H | 2.140640 | -0.638430 | -2.159723 |
| C | 4.749771 | 0.524497 | 1.036404 |
| H | 5.761316 | 0.650419 | 0.636809 |
| H | 4.495004 | 1.416719 | 1.617782 |
| H | 4.747675 | -0.336709 | 1.712456 |
| C | 3.732592 | 1.567585 | -1.009142 |
| H | 3.627171 | 2.483063 | -0.415829 |
| H | 4.683999 | 1.622863 | -1.550591 |
| H | 2.918262 | 1.534358 | -1.734816 |

Electronic Energy = -2106.40715696 (Hartree/Particle)
Dipole Moment (Debye): 6.9395
index 0
Harmonic frequencies
Number of imaginary frequencies= 0

THERMOCHEMICAL DATA

Temperature 298.150 Kelvin. Pressure 1.00000 Atm.
Zero-point correction= 0.452124 (Hartree/Particle)
Thermal correction to Energy= 0.483169
Thermal correction to Enthalpy= 0.484114
Thermal correction to Gibbs Free Energy= 0.389808
Sum of electronic and zero-point Energies= -2105.955033
Sum of electronic and thermal Energies= -2105.923987
Sum of electronic and thermal Enthalpies= -2105.923043
Sum of electronic and thermal Free Energies= -2106.017349
PCM-SP (solvent=THF;6-311+g(2d,p)) = -2106.80650319
Corrected Free Energy = -2106.41669523

-----------------------------------------------------------

TS3-B and correponding intermediate I3-B Ref 34 (main text)

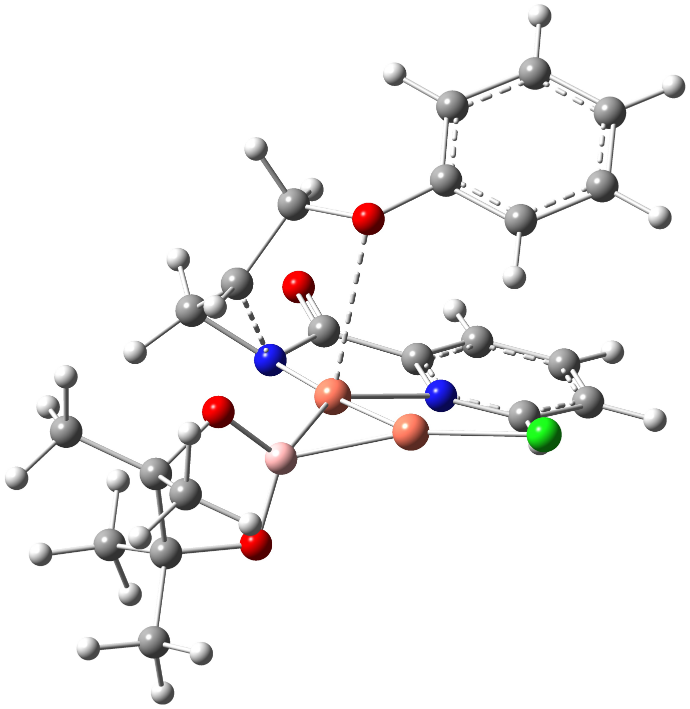

TS3-B (Ref 34)     Substrate: 1c
Path: Markovnikov

CARTESIAN COORDINATES

| | | | |
|---|---|---|---|
| C | 0.307167 | -1.789726 | 1.469768 |
| N | -0.124300 | -2.388471 | -0.375046 |
| C | -1.426480 | -2.679262 | -0.769972 |
| O | -1.991829 | -3.743583 | -0.531902 |
| C | 0.404440 | -3.107495 | 0.772249 |
| C | -2.105535 | -1.573850 | -1.536112 |
| C | -3.362010 | -1.767478 | -2.104850 |
| C | -3.976231 | -0.682631 | -2.736276 |
| C | -3.321267 | 0.548565 | -2.759474 |
| C | -2.066305 | 0.660748 | -2.153289 |
| N | -1.472673 | -0.385768 | -1.566648 |
| Cu | 0.717306 | 1.809347 | -0.334041 |
| H | -3.834073 | -2.740929 | -2.027526 |
| H | -4.956690 | -0.795970 | -3.190328 |
| H | -1.527935 | 1.603916 | -2.111060 |
| H | -3.769233 | 1.419731 | -3.226821 |
| H | -0.243078 | -3.909665 | 1.136877 |
| H | 1.422777 | -3.463202 | 0.606676 |
| Cu | 0.298143 | -0.494479 | -0.401520 |
| B | 2.211347 | 0.433584 | -0.093348 |
| O | 2.813493 | 0.208233 | 1.141766 |
| O | 3.121133 | 0.268171 | -1.124469 |
| C | 4.256861 | 0.134498 | 0.935320 |
| C | 4.346387 | -0.296360 | -0.570903 |
| Cl | -0.323420 | 3.661415 | -0.721303 |
| H | 1.202765 | -1.237785 | 1.747666 |
| C | -0.980254 | -1.448232 | 2.157016 |
| H | -1.796404 | -2.066333 | 1.766062 |
| H | -0.868980 | -1.672865 | 3.232324 |
| O | -1.249900 | -0.064943 | 1.972179 |

| | | | |
|---|---|---|---|
| C | -2.561047 | 0.357867 | 1.899331 |
| C | -3.644027 | -0.360137 | 2.418124 |
| C | -2.757795 | 1.588588 | 1.261593 |
| C | -4.937287 | 0.161018 | 2.277978 |
| H | -3.498152 | -1.301007 | 2.939271 |
| C | -4.049363 | 2.095313 | 1.136874 |
| H | -1.902971 | 2.129266 | 0.867304 |
| C | -5.147867 | 1.383139 | 1.637474 |
| H | -5.778284 | -0.396986 | 2.682011 |
| H | -4.192914 | 3.050499 | 0.638483 |
| H | -6.153713 | 1.781276 | 1.534986 |
| C | 4.254791 | -1.814623 | -0.775269 |
| H | 4.111001 | -2.020822 | -1.840353 |
| H | 5.164782 | -2.323802 | -0.439223 |
| H | 3.398326 | -2.231424 | -0.234938 |
| C | 5.537905 | 0.270838 | -1.336074 |
| H | 6.480045 | -0.065126 | -0.886017 |
| H | 5.509710 | -0.077566 | -2.373709 |
| H | 5.523228 | 1.362911 | -1.347527 |
| C | 4.810612 | 1.539920 | 1.200083 |
| H | 4.526441 | 1.848106 | 2.211176 |
| H | 5.903202 | 1.561743 | 1.122582 |
| H | 4.392518 | 2.266598 | 0.496608 |
| C | 4.841073 | -0.863821 | 1.930195 |
| H | 5.912127 | -1.009722 | 1.745715 |
| H | 4.718782 | -0.483506 | 2.949840 |
| H | 4.343159 | -1.835104 | 1.868628 |

Electronic Energy = -2106.30448992 (Hartree/Particle)
Dipole Moment (Debye): 6.6327
index 1
Number of imaginary frequencies= 1
Negatives Eigenvalues: -164.577

THERMOCHEMICAL DATA

Temperature 298.150 Kelvin. Pressure 1.00000 Atm.
Zero-point correction= 0.448288 (Hartree/Particle)
Thermal correction to Energy= 0.480008
Thermal correction to Enthalpy= 0.480953
Thermal correction to Gibbs Free Energy= 0.382024
Sum of electronic and zero-point Energies= -2105.856202
Sum of electronic and thermal Energies= -2105.824481
Sum of electronic and thermal Enthalpies= -2105.823537
Sum of electronic and thermal Free Energies= -2105.922465
PCM-SP (solvent=THF;6-311+g(2d,p)) = -2106.70645868
Corrected Free Energy = -2106.32443378

---

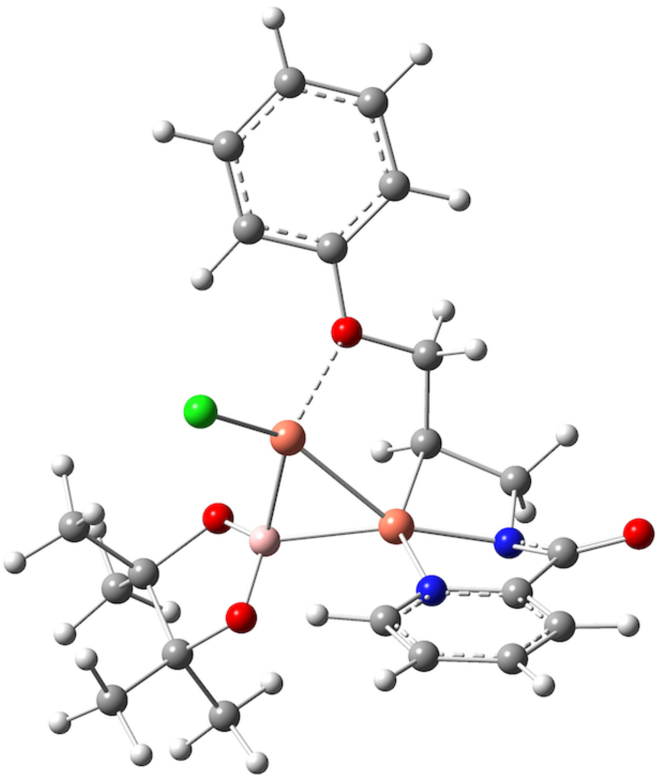

I2-B (Ref 34)     Substrate: 1c
                  Path: Markovnikov

CARTESIAN COORDINATES

| | | | |
|---|---|---|---|
| C | 0.508959 | -0.951067 | -2.166175 |
| N | -1.263181 | -2.261577 | -1.729352 |
| C | -1.587622 | -3.352998 | -0.999813 |
| O | -1.427235 | -4.541536 | -1.286086 |
| C | -0.234266 | -2.168476 | -2.756774 |
| C | -2.215215 | -2.927481 | 0.327868 |
| C | -2.889162 | -3.817871 | 1.156593 |
| C | -3.386137 | -3.348743 | 2.376072 |
| C | -3.180704 | -2.014016 | 2.734832 |
| C | -2.478477 | -1.182109 | 1.861366 |
| N | -2.021116 | -1.637908 | 0.687838 |
| Cu | 0.681220 | 0.416354 | 0.758278 |
| H | -2.996755 | -4.849077 | 0.836685 |
| H | -3.920445 | -4.019545 | 3.043404 |
| H | -2.264826 | -0.140616 | 2.078295 |
| H | -3.543870 | -1.619573 | 3.678337 |
| H | 0.382014 | -3.075950 | -2.832679 |
| H | -0.667072 | -1.962838 | -3.742067 |
| Cu | -0.838395 | -0.753486 | -0.700583 |
| B | -0.814301 | 1.311144 | -0.302817 |
| O | -0.301958 | 2.287962 | -1.124943 |
| O | -2.013663 | 1.665341 | 0.275806 |
| C | -1.035438 | 3.520284 | -0.811288 |
| C | -2.409554 | 2.955984 | -0.297503 |
| Cl | 1.139317 | 1.205019 | 2.709054 |
| H | 0.516270 | -0.033412 | -2.755428 |
| C | 1.851245 | -1.318316 | -1.584617 |
| H | 1.796908 | -2.282005 | -1.060772 |

| | | | |
|---|---|---|---|
| H | 2.570810 | -1.415022 | -2.413444 |
| O | 2.318903 | -0.308549 | -0.683772 |
| C | 3.642540 | -0.352308 | -0.262236 |
| C | 4.511079 | -1.412355 | -0.537006 |
| C | 4.068556 | 0.746248 | 0.492210 |
| C | 5.826927 | -1.353822 | -0.057973 |
| H | 4.187484 | -2.279645 | -1.101040 |
| C | 5.377333 | 0.785145 | 0.966337 |
| H | 3.359351 | 1.534299 | 0.723753 |
| C | 6.266629 | -0.261626 | 0.689854 |
| H | 6.502638 | -2.177870 | -0.272096 |
| H | 5.700223 | 1.635347 | 1.560895 |
| H | 7.287002 | -0.226919 | 1.061115 |
| C | -3.402295 | 2.644669 | -1.422288 |
| H | -4.236881 | 2.068327 | -1.010460 |
| H | -3.800513 | 3.561975 | -1.869411 |
| H | -2.931868 | 2.046158 | -2.209220 |
| C | -3.072797 | 3.781492 | 0.799028 |
| H | -3.291712 | 4.793223 | 0.437028 |
| H | -4.017125 | 3.312673 | 1.095359 |
| H | -2.435985 | 3.853604 | 1.683443 |
| C | -0.227765 | 4.238457 | 0.276109 |
| H | 0.780843 | 4.429406 | -0.103835 |
| H | -0.685032 | 5.197432 | 0.543177 |
| H | -0.132105 | 3.622364 | 1.175857 |
| C | -1.120000 | 4.367473 | -2.075340 |
| H | -1.738928 | 5.255355 | -1.898787 |
| H | -0.117614 | 4.701338 | -2.362126 |
| H | -1.542960 | 3.806045 | -2.911973 |

Electronic Energy =  -2106.31437690 (Hartree/Particle)
Dipole Moment (Debye):      3.9688
index          0
Harmonic frequencies
Number of imaginary frequencies=            0

THERMOCHEMICAL DATA

Temperature   298.150 Kelvin.  Pressure   1.00000 Atm.
Zero-point correction=                          0.449496
(Hartree/Particle)
Thermal correction to Energy=                   0.481514
Thermal correction to Enthalpy=                 0.482458
Thermal correction to Gibbs Free Energy=        0.381930
Sum of electronic and zero-point Energies=         -2105.864881
Sum of electronic and thermal Energies=            -2105.832863
Sum of electronic and thermal Enthalpies=          -2105.831918
Sum of electronic and thermal Free Energies=       -2105.932447
PCM-SP (solvent=THF;6-311+g(2d,p)) =   -2106.71642467
Corrected Free Energy =   -2106.33449477

---

PATH: ANTI-OPENING. TS-ANTI and the corresponding minima: Complex-ANTI and intermediate I-ANTI (Ref 35 main text)

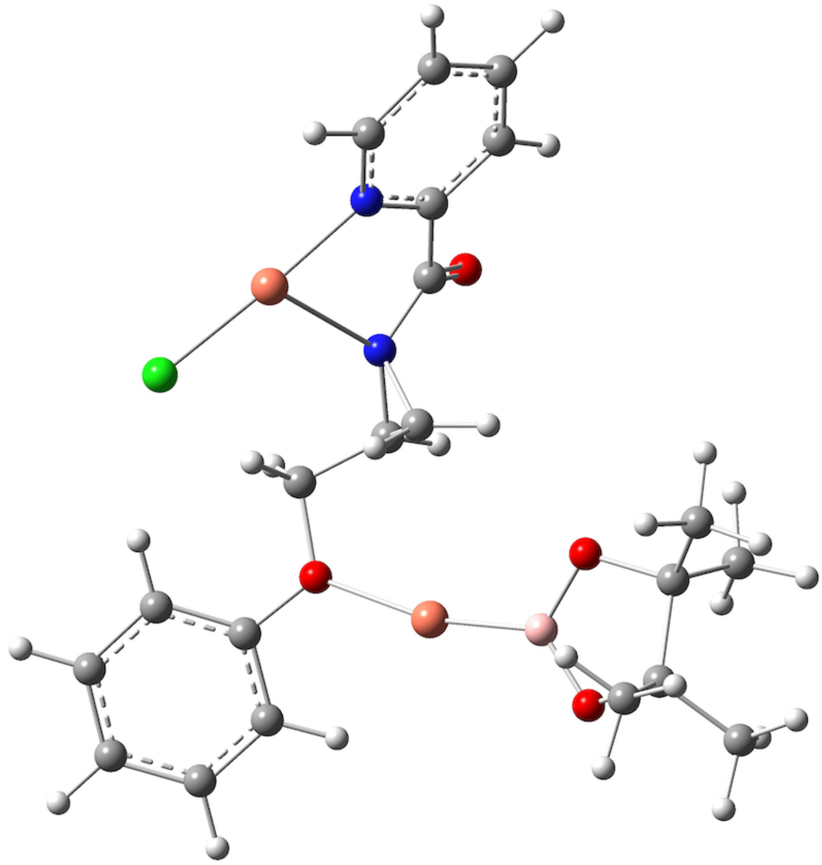

Complex-ANTI (Ref 35)   Substrate: 1c

CARTESIAN COORDINATES

| | | | |
|---|---|---|---|
| C | -0.530065 | 0.642188 | 1.258186 |
| N | -1.836307 | 0.158957 | 0.855423 |
| C | -2.620921 | -0.649634 | 1.669422 |
| O | -2.510553 | -0.746241 | 2.881582 |
| C | -0.635360 | -0.337330 | 0.132573 |
| C | -3.719808 | -1.357982 | 0.917332 |
| C | -4.309111 | -2.489386 | 1.481823 |
| C | -5.328212 | -3.141615 | 0.788718 |
| C | -5.732845 | -2.630434 | -0.445569 |
| C | -5.109124 | -1.485224 | -0.937710 |
| N | -4.118765 | -0.852977 | -0.277117 |
| Cu | 2.231466 | 0.517867 | 0.292361 |
| H | -3.957012 | -2.830176 | 2.449377 |
| H | -5.797712 | -4.028693 | 1.203795 |
| H | -5.402124 | -1.042360 | -1.883491 |
| H | -6.523758 | -3.099745 | -1.021777 |
| B | 2.850331 | -1.294343 | -0.057517 |
| O | 4.135623 | -1.744986 | -0.312077 |
| O | 1.949510 | -2.366317 | -0.075815 |
| C | 4.072686 | -3.134461 | -0.740838 |
| C | 2.707210 | -3.609543 | -0.131192 |
| H | -0.309394 | -1.356868 | 0.319053 |
| H | -0.548085 | 0.017374 | -0.891159 |
| H | -0.143118 | 0.272996 | 2.206290 |
| C | -0.282934 | 2.094681 | 0.930792 |
| H | -0.825522 | 2.359066 | 0.018716 |
| H | -0.590969 | 2.753133 | 1.750933 |

```
 O              1.145236    2.247915    0.732599
 C              1.634477    3.533407    0.498175
 C              2.993089    3.728716    0.760693
 C              0.835782    4.567345    0.006139
 C              3.562100    4.979790    0.524268
 H              3.583634    2.898829    1.139909
 C              1.421720    5.819088   -0.218115
 H             -0.211858    4.411791   -0.226152
 C              2.777852    6.032218    0.037217
 H              4.619172    5.131169    0.725320
 H              0.805208    6.625379   -0.606138
 H              3.221955    7.006942   -0.143857
 Cu            -3.341995    0.785494   -1.005406
 Cl            -2.604011    2.537813   -1.964477
 C              4.080800   -3.125632   -2.276158
 H              4.974792   -2.595791   -2.620303
 H              4.098252   -4.141864   -2.686799
 H              3.205474   -2.600054   -2.670919
 C              5.304830   -3.867045   -0.215221
 H              5.247530   -4.938009   -0.446815
 H              6.204024   -3.461588   -0.691092
 H              5.414007   -3.746396    0.865194
 C              1.935917   -4.618565   -0.978595
 H              2.520807   -5.536343   -1.116814
 H              0.999531   -4.884778   -0.475358
 H              1.686968   -4.210994   -1.961360
 C              2.830637   -4.118754    1.311782
 H              1.826556   -4.238177    1.732765
 H              3.342977   -5.086641    1.356850
 H              3.375212   -3.402574    1.935031

 Electronic Energy =  -2106.30357832 (Hartree/Particle)
 Dipole Moment (Debye):      7.6683
 index           0
 Harmonic frequencies
 Number of imaginary frequencies=           0

 THERMOCHEMICAL DATA

 Temperature   298.150 Kelvin.  Pressure   1.00000 Atm.
 Zero-point correction=                            0.449832
(Hartree/Particle)
 Thermal correction to Energy=                     0.482532
 Thermal correction to Enthalpy=                   0.483476
 Thermal correction to Gibbs Free Energy=          0.378741
 Sum of electronic and zero-point Energies=          -2105.853746
 Sum of electronic and thermal Energies=             -2105.821046
 Sum of electronic and thermal Enthalpies=           -2105.820102
 Sum of electronic and thermal Free Energies=        -2105.924837
 PCM-SP (solvent=THF;6-311+g(2d,p)) =  -2106.70537461
 Corrected Free Energy =  -2106.32663329
```

---------------------------------------------------------------

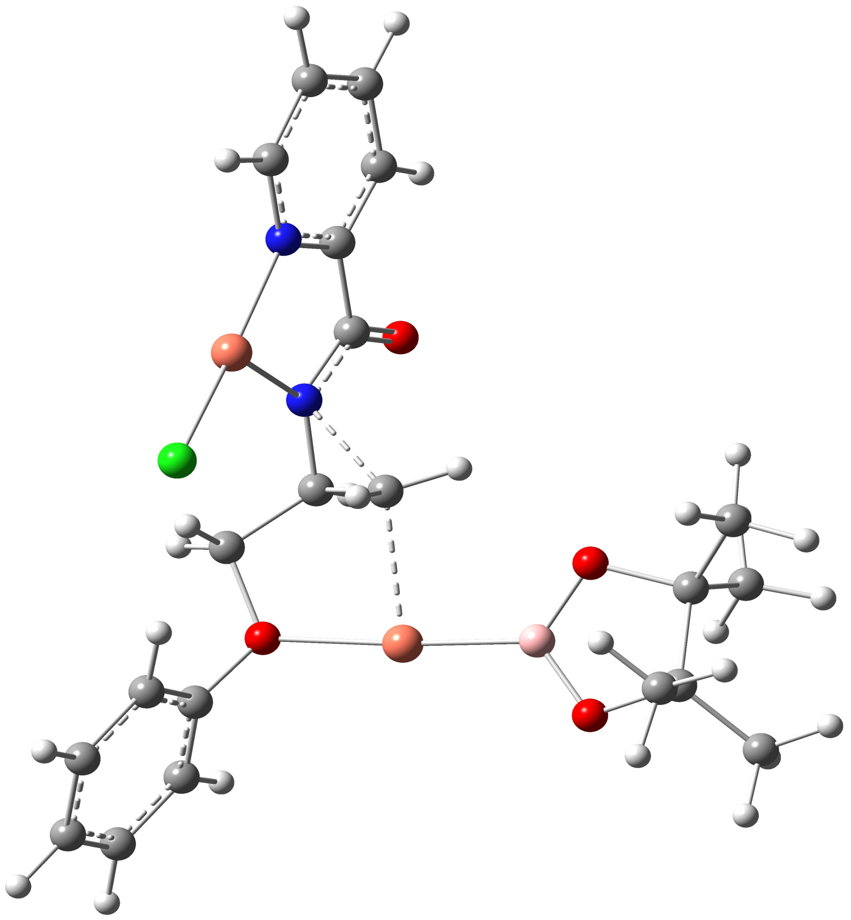

TS-ANTI (Ref 35)     Substrate: 1c
Path: anti-Markovnikov

CARTESIAN COORDINATES

| | | | |
|---|---|---|---|
| C | 0.749321 | 0.642419 | -1.470800 |
| N | 1.999441 | 0.203127 | -0.899466 |
| C | 2.706054 | -0.772974 | -1.566444 |
| O | 2.428491 | -1.276487 | -2.656195 |
| C | 0.277393 | -0.285548 | -0.415727 |
| C | 3.957394 | -1.191754 | -0.823493 |
| C | 4.794979 | -2.156873 | -1.385523 |
| C | 5.950190 | -2.536110 | -0.702670 |
| C | 6.231432 | -1.937492 | 0.526792 |
| C | 5.345676 | -0.982905 | 1.025899 |
| N | 4.231648 | -0.610142 | 0.369993 |
| Cu | -1.915096 | 0.432228 | -0.585254 |
| H | 4.519104 | -2.584999 | -2.342863 |
| H | 6.617376 | -3.284432 | -1.121156 |
| H | 5.517698 | -0.491081 | 1.977465 |
| H | 7.117973 | -2.198925 | 1.095942 |
| B | -2.669768 | -1.309688 | -0.104701 |
| O | -4.005133 | -1.548146 | 0.147224 |
| O | -1.912966 | -2.462894 | 0.034051 |
| C | -4.131232 | -2.891824 | 0.708693 |
| C | -2.825686 | -3.592892 | 0.188257 |
| H | 0.170005 | -1.340163 | -0.637690 |

| | | | |
|---|---|---|---|
| H | 0.276586 | 0.035752 | 0.622041 |
| H | 0.551829 | 0.311027 | -2.492424 |
| C | 0.451269 | 2.106578 | -1.209662 |
| H | 0.904737 | 2.412881 | -0.263882 |
| H | 0.790205 | 2.754003 | -2.024678 |
| O | -1.001527 | 2.202949 | -1.120577 |
| C | -1.519477 | 3.421567 | -0.647300 |
| C | -2.417219 | 4.096002 | -1.471385 |
| C | -1.182489 | 3.890328 | 0.623018 |
| C | -2.997360 | 5.281523 | -1.009594 |
| H | -2.653795 | 3.686658 | -2.448998 |
| C | -1.763049 | 5.082012 | 1.065974 |
| H | -0.487070 | 3.338250 | 1.250078 |
| C | -2.668469 | 5.777129 | 0.256166 |
| H | -3.702550 | 5.815606 | -1.640713 |
| H | -1.509435 | 5.459676 | 2.052642 |
| H | -3.118206 | 6.700078 | 0.611950 |
| Cu | 2.941048 | 0.704754 | 1.097341 |
| Cl | 1.700973 | 1.989016 | 2.312967 |
| C | -2.984185 | -4.222020 | -1.201978 |
| H | -1.995777 | -4.506446 | -1.577287 |
| H | -3.614113 | -5.117983 | -1.168142 |
| H | -3.422650 | -3.510677 | -1.909049 |
| C | -2.201149 | -4.596278 | 1.153544 |
| H | -2.899087 | -5.416285 | 1.362144 |
| H | -1.296448 | -5.023156 | 0.707427 |
| H | -1.921098 | -4.125227 | 2.098622 |
| C | -5.436861 | -3.506602 | 0.212915 |
| H | -5.523555 | -4.548137 | 0.546090 |
| H | -6.284992 | -2.945564 | 0.619126 |
| H | -5.506756 | -3.479741 | -0.877008 |
| C | -4.167242 | -2.728082 | 2.233731 |
| H | -4.993608 | -2.060569 | 2.497817 |
| H | -4.320914 | -3.688963 | 2.737690 |
| H | -3.240030 | -2.280368 | 2.605231 |

Electronic Energy = -2106.29170211 (Hartree/Particle)
Dipole Moment (Debye): 4.4089
index 1
Number of imaginary frequencies= 1
Negatives Eigenvalues: -368.166

THERMOCHEMICAL DATA

Temperature 298.150 Kelvin. Pressure 1.00000 Atm.
Zero-point correction= 0.448483 (Hartree/Particle)
Thermal correction to Energy= 0.480537
Thermal correction to Enthalpy= 0.481481
Thermal correction to Gibbs Free Energy= 0.380349
Sum of electronic and zero-point Energies= -2105.843219
Sum of electronic and thermal Energies= -2105.811165
Sum of electronic and thermal Enthalpies= -2105.810221
Sum of electronic and thermal Free Energies= -2105.911353
PCM-SP (solvent=THF;6-311+g(2d,p)) = -2106.69111316
Corrected Free Energy = -2106.310764052

---

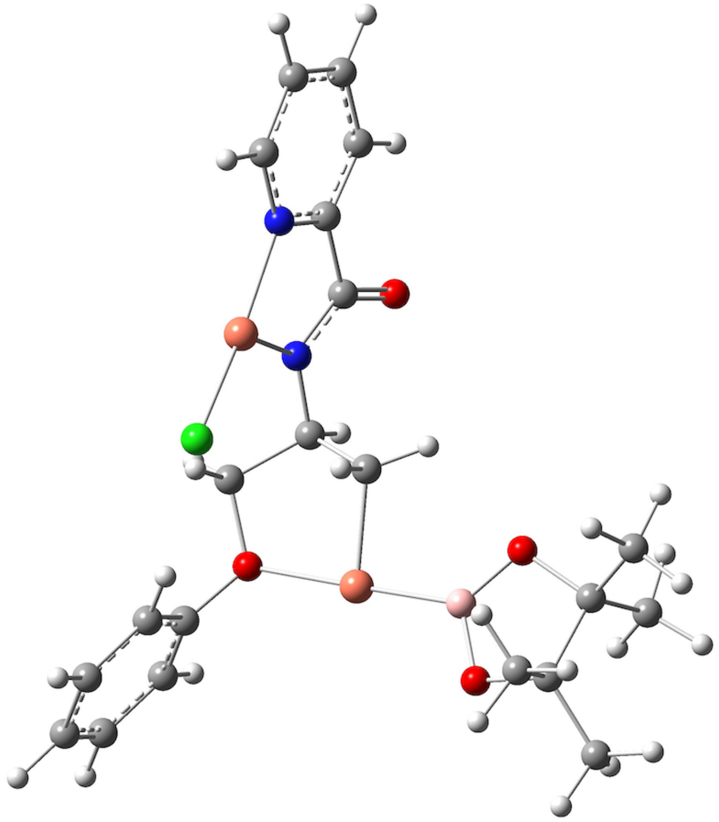

I-ANTI (Ref 35)      Substrate: 1c
Path: anti-Markovnikov

CARTESIAN COORDINATES

| | | | |
|---|---|---|---|
| C | 0.870390 | 0.483220 | -1.391602 |
| N | 2.163295 | 0.148304 | -0.863028 |
| C | 2.891909 | -0.789973 | -1.503370 |
| O | 2.635123 | -1.379778 | -2.567586 |
| C | 0.012736 | -0.433938 | -0.515526 |
| C | 4.186542 | -1.101449 | -0.766692 |
| C | 5.167254 | -1.883078 | -1.379666 |
| C | 6.346795 | -2.158255 | -0.688183 |
| C | 6.506738 | -1.649025 | 0.602734 |
| C | 5.476443 | -0.885490 | 1.151537 |
| N | 4.340703 | -0.612882 | 0.486959 |
| Cu | -1.760464 | 0.423450 | -0.784135 |
| H | 4.976409 | -2.254938 | -2.380450 |
| H | 7.126934 | -2.760550 | -1.146143 |
| H | 5.544872 | -0.474916 | 2.154092 |
| H | 7.405974 | -1.839122 | 1.180715 |
| B | -2.602849 | -1.273764 | -0.269085 |
| O | -3.894121 | -1.184739 | 0.179168 |
| O | -2.103652 | -2.543617 | -0.270648 |
| C | -4.236650 | -2.497121 | 0.758239 |
| C | -3.218893 | -3.451063 | 0.030454 |
| H | 0.040428 | -1.480861 | -0.807503 |
| H | 0.142433 | -0.270237 | 0.558583 |
| H | 0.750683 | 0.227880 | -2.451365 |
| C | 0.601585 | 1.961569 | -1.132569 |
| H | 0.924892 | 2.221517 | -0.122864 |
| H | 1.084493 | 2.605867 | -1.872072 |

| | | | |
|---|---|---|---|
| O | -0.852806 | 2.165049 | -1.241774 |
| C | -1.369796 | 3.351751 | -0.685853 |
| C | -2.100466 | 4.184701 | -1.529969 |
| C | -1.194000 | 3.634822 | 0.669746 |
| C | -2.678032 | 5.342253 | -0.998931 |
| H | -2.207418 | 3.921161 | -2.578064 |
| C | -1.770673 | 4.800235 | 1.182216 |
| H | -0.616275 | 2.967726 | 1.306160 |
| C | -2.511859 | 5.651731 | 0.354910 |
| H | -3.251827 | 6.001070 | -1.645064 |
| H | -1.639753 | 5.035814 | 2.234649 |
| H | -2.958234 | 6.553833 | 0.764252 |
| Cu | 2.767511 | 0.464167 | 1.169616 |
| Cl | 1.336044 | 1.443179 | 2.504410 |
| C | -2.686910 | -4.597144 | 0.883765 |
| H | -3.508313 | -5.250351 | 1.201400 |
| H | -1.980776 | -5.194541 | 0.298232 |
| H | -2.165293 | -4.231553 | 1.770958 |
| C | -3.726108 | -3.974791 | -1.317437 |
| H | -2.897288 | -4.456557 | -1.845232 |
| H | -4.527072 | -4.709613 | -1.182616 |
| H | -4.100373 | -3.160071 | -1.945657 |
| C | -5.706708 | -2.779725 | 0.472706 |
| H | -5.974603 | -3.786030 | 0.816274 |
| H | -6.331542 | -2.057929 | 1.008436 |
| H | -5.935051 | -2.700874 | -0.592556 |
| C | -3.992703 | -2.376814 | 2.265923 |
| H | -4.597808 | -1.554231 | 2.659383 |
| H | -4.275670 | -3.297290 | 2.787881 |
| H | -2.942602 | -2.159121 | 2.484695 |

Electronic Energy = -2106.29855042 (Hartree/Particle)
Dipole Moment (Debye): 4.8348
index 0
Harmonic frequencies
Number of imaginary frequencies= 0

THERMOCHEMICAL DATA

Temperature 298.150 Kelvin. Pressure 1.00000 Atm.
Zero-point correction= 0.449219
(Hartree/Particle)
Thermal correction to Energy= 0.481494
Thermal correction to Enthalpy= 0.482438
Thermal correction to Gibbs Free Energy= 0.380654
Sum of electronic and zero-point Energies= -2105.849331
Sum of electronic and thermal Energies= -2105.817057
Sum of electronic and thermal Enthalpies= -2105.816113
Sum of electronic and thermal Free Energies= -2105.917897
PCM-SP (solvent=THF;6-311+g(2d,p)) = -2106.70346197
Corrected Free Energy = -2106.32280855

---

---

MODEL: [Az-(Cu)2-Bpin]+/Et2O

SUBSTRATE: N-(2-PICOLINOYL)-METHYL AZIRIDINE

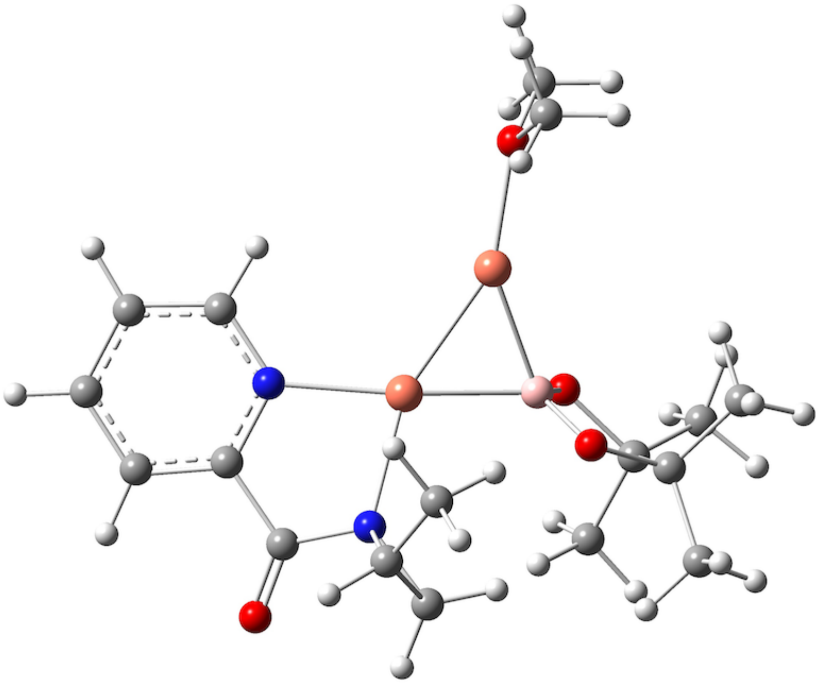

Complex-3     Substrate: N-(2-picolinoyl)-methyl aziridine

CARTESIAN COORDINATES

| | | | |
|---|---|---|---|
| C | -1.622828 | -2.024526 | 2.067654 |
| N | -1.634373 | -1.650290 | 0.586061 |
| C | -2.920781 | -1.735007 | -0.032439 |
| O | -3.503346 | -2.773999 | -0.262503 |
| C | -1.027365 | -2.916992 | 1.050105 |
| C | -3.494921 | -0.391474 | -0.384133 |
| C | -4.831857 | -0.281133 | -0.764719 |
| C | -5.334804 | 0.984322 | -1.074148 |
| C | -4.481917 | 2.086885 | -0.998723 |
| C | -3.151618 | 1.886600 | -0.623648 |
| N | -2.664364 | 0.674298 | -0.319376 |
| Cu | 0.947242 | 1.800979 | 0.156688 |
| H | -5.444890 | -1.174806 | -0.810273 |
| H | -6.372484 | 1.106273 | -1.369936 |
| H | -2.445156 | 2.708560 | -0.567791 |
| H | -4.831799 | 3.087196 | -1.232769 |
| H | -1.552506 | -3.825486 | 0.768996 |
| H | 0.053315 | -2.914088 | 0.949190 |
| C | -0.805652 | -1.160460 | 2.990741 |
| H | -0.663354 | -1.685524 | 3.942945 |
| H | 0.176536 | -0.946576 | 2.560435 |
| Cu | -0.702231 | 0.172544 | 0.022006 |
| B | 1.308136 | -0.220973 | -0.175610 |
| O | 1.875196 | -1.050364 | 0.774563 |
| O | 1.841024 | -0.422532 | -1.430556 |
| C | 3.045029 | -1.694165 | 0.157435 |
| C | 2.697105 | -1.614414 | -1.372602 |
| C | 4.258412 | -0.845567 | 0.553975 |
| H | 4.314497 | -0.791679 | 1.645614 |
| H | 5.190617 | -1.281199 | 0.180554 |
| H | 4.170398 | 0.174663 | 0.165507 |
| C | 3.180497 | -3.105550 | 0.717114 |

| | | | |
|---|---|---|---|
| H | 4.012817 | -3.628842 | 0.232995 |
| H | 3.385786 | -3.060820 | 1.791434 |
| H | 2.273736 | -3.696218 | 0.567509 |
| C | 3.892455 | -1.399642 | -2.294478 |
| H | 4.596018 | -2.235799 | -2.209405 |
| H | 3.552979 | -1.344598 | -3.333430 |
| H | 4.422050 | -0.473021 | -2.062121 |
| C | 1.840844 | -2.783234 | -1.872497 |
| H | 1.484522 | -2.556505 | -2.881810 |
| H | 2.416587 | -3.713658 | -1.909704 |
| H | 0.965057 | -2.938180 | -1.234536 |
| H | -2.610052 | -2.315554 | 2.425265 |
| H | -1.322988 | -0.216858 | 3.199391 |
| O | 1.572752 | 3.670284 | 0.377573 |
| C | 2.232441 | 4.326414 | -0.726013 |
| H | 3.310902 | 4.134849 | -0.687419 |
| H | 1.810673 | 3.908406 | -1.641204 |
| H | 2.035915 | 5.403058 | -0.678657 |
| C | 2.009796 | 4.141289 | 1.669142 |
| H | 3.078182 | 3.936560 | 1.803448 |
| H | 1.816329 | 5.216585 | 1.749058 |
| H | 1.427450 | 3.600703 | 2.416660 |

Electronic Energy = -1494.66752736 (Hartree/Particle)
Dipole Moment (Debye): 6.5515
index 0
Harmonic frequencies
Number of imaginary frequencies= 0

THERMOCHEMICAL DATA

Temperature 298.150 Kelvin. Pressure 1.00000 Atm.
Zero-point correction= 0.445587 (Hartree/Particle)
Thermal correction to Energy= 0.476418
Thermal correction to Enthalpy= 0.477363
Thermal correction to Gibbs Free Energy= 0.379819
Sum of electronic and zero-point Energies= -1494.221940
Sum of electronic and thermal Energies= -1494.191109
Sum of electronic and thermal Enthalpies= -1494.190165
Sum of electronic and thermal Free Energies= -1494.287708
PCM-SP (solvent=THF;6-311+g(2d,p)) = -1495.03244674
Corrected Free Energy = -1494.65262738

------------------------------------------------------

PATH: ANTI_MARKOVNIKOV

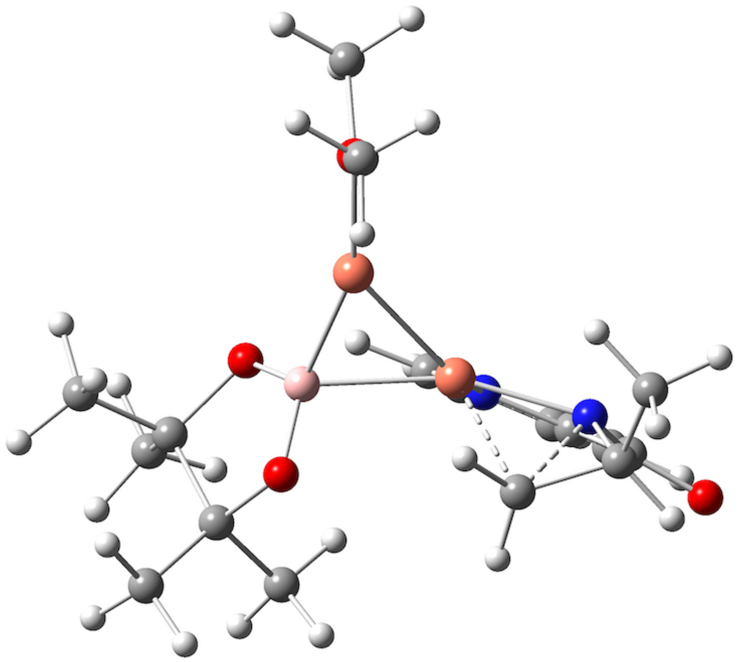

TS6 Substrate: N-(2-picolinoyl)-methyl aziridine
Path: anti-markovnikov

CARTESIAN COORDINATES

| | | | |
|---|---|---|---|
| C | -0.519753 | -1.705512 | 2.098655 |
| N | -1.719718 | -2.022773 | 0.601200 |
| C | -2.995355 | -1.471731 | 0.395808 |
| O | -4.003184 | -1.879996 | 0.950359 |
| C | -1.498241 | -2.791461 | 1.821734 |
| C | -3.040690 | -0.386718 | -0.649237 |
| C | -4.254505 | 0.077438 | -1.150180 |
| C | -4.228434 | 1.095576 | -2.107426 |
| C | -2.998119 | 1.618888 | -2.511181 |
| C | -1.827458 | 1.111109 | -1.941894 |
| N | -1.856035 | 0.122734 | -1.038155 |
| Cu | 1.981935 | -0.858003 | -0.428744 |
| H | -5.179916 | -0.354950 | -0.785013 |
| H | -5.154679 | 1.476381 | -2.527602 |
| H | -0.843833 | 1.498488 | -2.187151 |
| H | -2.938516 | 2.409737 | -3.252164 |
| H | -2.375483 | -2.792596 | 2.473371 |
| H | -0.844070 | -0.817819 | 2.633829 |
| Cu | -0.333172 | -0.793036 | 0.063376 |
| B | 1.061889 | 0.841975 | 0.192948 |
| O | 1.058368 | 1.890486 | -0.697158 |
| O | 0.952632 | 1.252091 | 1.503048 |
| C | 1.151060 | 3.137260 | 0.083044 |
| C | 0.615447 | 2.686069 | 1.489779 |
| H | 0.548843 | -1.902779 | 2.067133 |
| C | -0.943630 | -4.185011 | 1.587081 |
| H | -0.623849 | -4.629252 | 2.536527 |
| H | -1.710626 | -4.827488 | 1.143777 |
| C | 1.288237 | 3.354821 | 2.682209 |
| H | 1.124357 | 4.438083 | 2.653733 |
| H | 0.860112 | 2.971006 | 3.613512 |
| H | 2.363488 | 3.164827 | 2.700114 |

| | | | |
|---|---|---|---|
| C | -0.910379 | 2.760797 | 1.617866 |
| H | -1.215735 | 2.258963 | 2.541564 |
| H | -1.257633 | 3.797781 | 1.661980 |
| H | -1.404421 | 2.261070 | 0.778622 |
| C | 2.629940 | 3.534851 | 0.087982 |
| H | 2.974456 | 3.640730 | -0.945239 |
| H | 2.783503 | 4.488742 | 0.602556 |
| H | 3.245018 | 2.772351 | 0.576786 |
| C | 0.310877 | 4.201599 | -0.613891 |
| H | 0.290343 | 5.121860 | -0.019333 |
| H | 0.747717 | 4.437747 | -1.589415 |
| H | -0.717762 | 3.868413 | -0.771322 |
| H | -0.085759 | -4.149322 | 0.905038 |
| O | 3.361647 | -2.111517 | -1.077823 |
| C | 4.444972 | -2.553912 | -0.233470 |
| H | 4.656621 | -3.609277 | -0.436225 |
| H | 5.336814 | -1.944269 | -0.417279 |
| H | 4.114077 | -2.431505 | 0.798944 |
| C | 3.638435 | -2.244064 | -2.488692 |
| H | 2.744799 | -1.913405 | -3.019858 |
| H | 4.494284 | -1.617113 | -2.762803 |
| H | 3.844412 | -3.294376 | -2.720994 |

Electronic Energy = -1494.63027914 (Hartree/Particle)
Dipole Moment (Debye): 4.9667
index 1
Number of imaginary frequencies= 1
Negatives Eigenvalues: -218.939

THERMOCHEMICAL DATA

Temperature 298.150 Kelvin. Pressure 1.00000 Atm.
Zero-point correction= 0.443468
(Hartree/Particle)
Thermal correction to Energy= 0.473912
Thermal correction to Enthalpy= 0.474856
Thermal correction to Gibbs Free Energy= 0.378379
Sum of electronic and zero-point Energies= -1494.186811
Sum of electronic and thermal Energies= -1494.156367
Sum of electronic and thermal Enthalpies= -1494.155423
Sum of electronic and thermal Free Energies= -1494.251900
PCM-SP (solvent=THF;6-311+g(2d,p)) = -1494.99838714
Corrected Free Energy = -1494.62000800

------------------------------------------------------

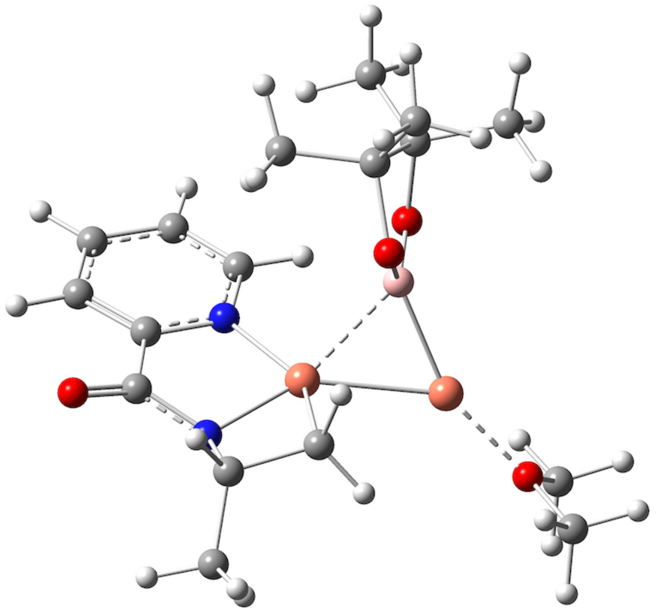

I4 Substrate: N-(2-picolinoyl)-methyl aziridine
Path: anti-markovnikov

CARTESIAN COORDINATES

| | | | |
|---|---|---|---|
| C | -0.062028 | -2.001004 | -1.752783 |
| N | 1.753341 | -1.993420 | -0.575061 |
| C | 2.983136 | -1.418280 | -0.382117 |
| O | 4.003913 | -1.698031 | -0.996859 |
| C | 1.327208 | -2.625282 | -1.822674 |
| C | 2.954379 | -0.402353 | 0.740642 |
| C | 4.116387 | 0.086603 | 1.326314 |
| C | 3.998336 | 1.034123 | 2.346793 |
| C | 2.726734 | 1.468135 | 2.730198 |
| C | 1.608305 | 0.949359 | 2.076509 |
| N | 1.727403 | 0.028766 | 1.107499 |
| Cu | -1.966066 | -0.792231 | 0.433375 |
| H | 5.075211 | -0.277581 | 0.972147 |
| H | 4.885035 | 1.430387 | 2.832972 |
| H | 0.600248 | 1.279466 | 2.299837 |
| H | 2.595456 | 2.202660 | 3.518193 |
| H | 1.905548 | -2.242211 | -2.672862 |
| H | -0.313732 | -1.201991 | -2.449388 |
| Cu | 0.344827 | -0.885595 | -0.095731 |
| B | -1.004531 | 0.854947 | -0.243478 |
| O | -1.024645 | 1.916502 | 0.621517 |
| O | -0.806096 | 1.213386 | -1.550870 |
| C | -1.027522 | 3.148409 | -0.197301 |
| C | -0.421140 | 2.640006 | -1.555985 |
| H | -0.886819 | -2.667617 | -1.497809 |
| C | 1.375893 | -4.145541 | -1.787769 |
| H | 0.938095 | -4.560918 | -2.703743 |
| H | 2.412269 | -4.489441 | -1.712392 |
| C | -0.998498 | 3.292098 | -2.805458 |
| H | -0.801736 | 4.370097 | -2.796002 |
| H | -0.525661 | 2.868517 | -3.696815 |
| H | -2.076193 | 3.134557 | -2.885096 |
| C | 1.110591 | 2.658003 | -1.591227 |
| H | 1.454262 | 2.109901 | -2.473756 |

```
 H              1.494413    3.681147   -1.651002
 H              1.535996    2.176745   -0.705038
 C             -2.488846    3.590973   -0.302729
 H             -2.891567    3.738874    0.703897
 H             -2.577820    4.533828   -0.851482
 H             -3.099208    2.835681   -0.808368
 C             -0.194168    4.200046    0.525115
 H             -0.101918    5.101267   -0.091295
 H             -0.683076    4.479723    1.463607
 H              0.808916    3.834537    0.756945
 H              0.819416   -4.528156   -0.923835
 O             -3.418709   -1.934754    1.095072
 C             -4.371119   -2.556540    0.204661
 H             -4.549977   -3.587601    0.527299
 H             -5.307444   -1.987852    0.204326
 H             -3.928182   -2.548745   -0.792378
 C             -3.853824   -1.897223    2.472678
 H             -4.768279   -1.300084    2.557718
 H             -4.029237   -2.919087    2.824891
 H             -3.049285   -1.437309    3.048025

 Electronic Energy =  -1494.63618139 (Hartree/Particle)
 Dipole Moment (Debye):      7.4110
 index          0
 Harmonic frequencies
 Number of imaginary frequencies=           0

 THERMOCHEMICAL DATA

 Temperature   298.150 Kelvin.  Pressure   1.00000 Atm.
 Zero-point correction=                           0.444581
(Hartree/Particle)
 Thermal correction to Energy=                    0.475281
 Thermal correction to Enthalpy=                  0.476226
 Thermal correction to Gibbs Free Energy=         0.379534
 Sum of electronic and zero-point Energies=          -1494.191600
 Sum of electronic and thermal Energies=             -1494.160900
 Sum of electronic and thermal Enthalpies=           -1494.159956
 Sum of electronic and thermal Free Energies=        -1494.256648
 PCM-SP (solvent=THF;6-311+g(2d,p)) =   -1495.00805742
 Corrected Free Energy =  -1494.62852403
```

------------------------------------------------------

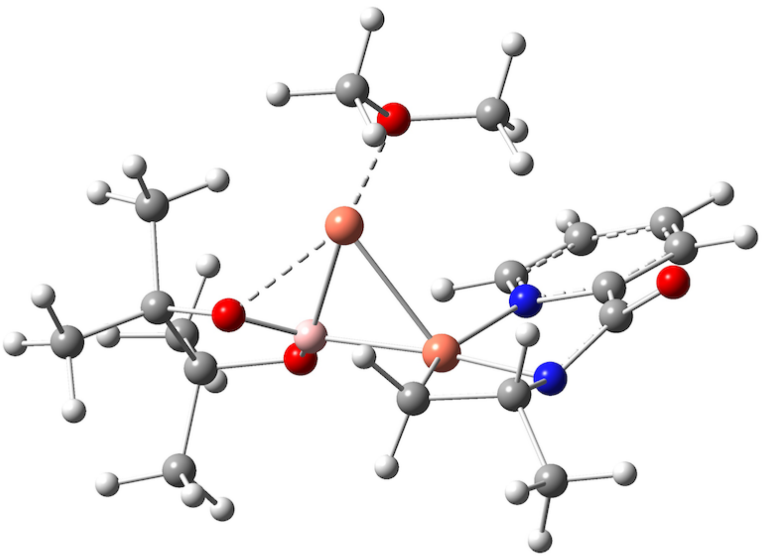

TS7 Substrate: N-(2-picolinoyl)-methyl aziridine
Path: anti-markovnikov

CARTESIAN COORDINATES

| | | | |
|---|---|---|---|
| N | -1.341523 | 1.690711 | -0.070809 |
| C | -2.684775 | 1.534832 | -0.119351 |
| C | -3.502407 | 2.443409 | -0.781734 |
| C | -2.910435 | 3.562834 | -1.373802 |
| C | -1.523400 | 3.724743 | -1.301954 |
| C | -0.763043 | 2.755033 | -0.648428 |
| C | -3.209900 | 0.271926 | 0.547683 |
| O | -4.284303 | -0.233400 | 0.224959 |
| Cu | -0.562472 | 0.093511 | 0.886909 |
| N | -2.319811 | -0.181747 | 1.469437 |
| C | -2.235886 | -1.576381 | 1.921501 |
| C | -0.707266 | -1.660718 | 1.857030 |
| B | 1.462592 | 0.134015 | 0.448949 |
| Cu | 0.431260 | -1.188648 | -0.888183 |
| O | 2.363783 | -0.895944 | 0.217601 |
| C | 3.673057 | -0.258370 | -0.116340 |
| C | 4.782175 | -1.139407 | 0.439383 |
| O | 2.050516 | 1.356521 | 0.527906 |
| C | 3.518661 | 1.142944 | 0.575730 |
| C | 3.905101 | 1.144256 | 2.056170 |
| C | 3.730811 | -0.194077 | -1.643313 |
| C | 4.186280 | 2.295649 | -0.162979 |
| H | -4.571130 | 2.258782 | -0.817415 |
| H | -3.522486 | 4.300528 | -1.884668 |
| H | 0.317634 | 2.811055 | -0.565052 |
| H | -1.033003 | 4.584801 | -1.746180 |
| H | -0.182884 | -1.438534 | 2.790043 |
| H | 4.683342 | 0.220136 | -1.986966 |
| H | 3.633915 | -1.206263 | -2.049235 |
| H | 2.919230 | 0.420683 | -2.048309 |
| H | 4.802071 | -2.095663 | -0.092327 |
| H | 5.753718 | -0.652405 | 0.298711 |
| H | 4.645185 | -1.342891 | 1.503165 |
| H | 4.019044 | 3.229122 | 0.382641 |
| H | 5.267521 | 2.129226 | -0.227008 |

| | | | |
|---|---|---|---|
| H | 3.791654 | 2.415976 | -1.174536 |
| H | 4.988740 | 1.053170 | 2.180345 |
| H | 3.584812 | 2.087833 | 2.507353 |
| H | 3.422265 | 0.325597 | 2.599449 |
| O | -0.689347 | -2.076670 | -2.220289 |
| C | -0.553617 | -3.499652 | -2.439165 |
| H | -0.985002 | -3.756069 | -3.412089 |
| H | 0.514781 | -3.719563 | -2.435495 |
| H | -1.060676 | -4.053788 | -1.641053 |
| C | -2.067504 | -1.621729 | -2.246827 |
| H | -2.621747 | -2.032478 | -1.396887 |
| H | -2.040318 | -0.533938 | -2.180929 |
| H | -2.527580 | -1.927980 | -3.191290 |
| H | -2.702179 | -2.243221 | 1.183174 |
| H | -0.255068 | -2.500671 | 1.329066 |
| C | -2.845078 | -1.820622 | 3.296219 |
| H | -3.925950 | -1.647315 | 3.261933 |
| H | -2.668238 | -2.853344 | 3.620507 |
| H | -2.407946 | -1.138705 | 4.034511 |

Electronic Energy = -1494.62665898 (Hartree/Particle)
Dipole Moment (Debye): 6.1152
index 1
Number of imaginary frequencies= 1
Negatives Eigenvalues: -54.390

THERMOCHEMICAL DATA

Temperature 298.150 Kelvin. Pressure 1.00000 Atm.
Zero-point correction= 0.444377
(Hartree/Particle)
Thermal correction to Energy= 0.474024
Thermal correction to Enthalpy= 0.474969
Thermal correction to Gibbs Free Energy= 0.382269
Sum of electronic and zero-point Energies= -1494.182282
Sum of electronic and thermal Energies= -1494.152635
Sum of electronic and thermal Enthalpies= -1494.151690
Sum of electronic and thermal Free Energies= -1494.244390
PCM-SP (solvent=THF;6-311+g(2d,p)) = -1494.99926829
Corrected Free Energy = -1494.61699931

---

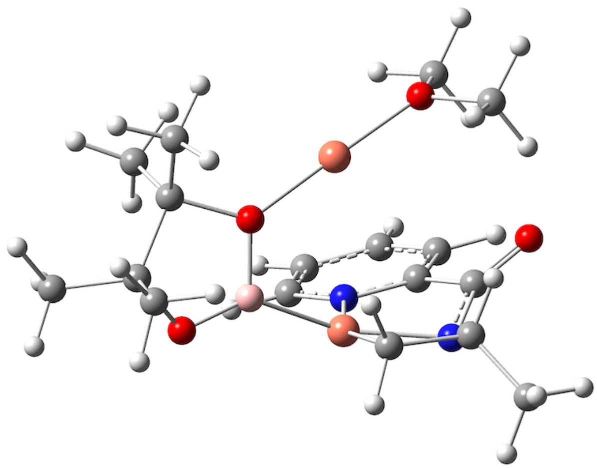

I5 Substrate: N-(2-picolinoyl)-methyl aziridine

Path: anti-markovnikov

CARTESIAN COORDINATES

| | | | |
|---|---|---|---|
| N | -1.434097 | -1.638047 | -0.767203 |
| C | -2.649461 | -1.148160 | -0.417649 |
| C | -3.636065 | -1.965398 | 0.125796 |
| C | -3.373430 | -3.329660 | 0.270682 |
| C | -2.122259 | -3.829460 | -0.104622 |
| C | -1.170744 | -2.945663 | -0.610968 |
| C | -2.808743 | 0.359624 | -0.587834 |
| O | -3.539707 | 0.998669 | 0.187096 |
| Cu | -0.340116 | -0.082714 | -1.448442 |
| N | -2.013303 | 0.805357 | -1.574585 |
| C | -1.473320 | 2.176900 | -1.619696 |
| C | 0.016351 | 1.823660 | -1.825586 |
| B | 1.530134 | -0.243412 | -0.914414 |
| Cu | 0.338963 | 0.680745 | 1.525958 |
| O | 1.788771 | 0.174682 | 0.426023 |
| C | 3.169524 | -0.285741 | 0.802068 |
| C | 3.770533 | 0.747484 | 1.740781 |
| O | 2.665175 | -0.685046 | -1.490635 |
| C | 3.819084 | -0.374396 | -0.621639 |
| C | 4.382676 | 0.959057 | -1.118187 |
| C | 2.986785 | -1.633639 | 1.496175 |
| C | 4.843619 | -1.491428 | -0.773607 |
| H | -4.583933 | -1.521771 | 0.412717 |
| H | -4.132990 | -3.996244 | 0.669090 |
| H | -0.179343 | -3.273734 | -0.907490 |
| H | -1.883479 | -4.883650 | -0.009003 |
| H | 3.946115 | -2.026369 | 1.845797 |
| H | 2.334704 | -1.506119 | 2.367775 |
| H | 2.530730 | -2.370203 | 0.828033 |
| H | 3.223816 | 0.753912 | 2.690726 |
| H | 4.813707 | 0.494663 | 1.958524 |
| H | 3.738274 | 1.752842 | 1.316107 |
| H | 5.240187 | -1.485872 | -1.792840 |
| H | 5.679521 | -1.339826 | -0.081336 |
| H | 4.405353 | -2.474245 | -0.587125 |
| H | 5.287801 | 1.236537 | -0.568746 |
| H | 4.638018 | 0.862060 | -2.177016 |
| H | 3.650472 | 1.767358 | -1.018880 |
| H | -1.623902 | 2.670902 | -0.649855 |
| H | 0.354515 | 1.849430 | -2.864799 |
| O | -1.128337 | 1.206688 | 2.594277 |
| C | -1.710505 | 2.532993 | 2.434893 |
| H | -2.496308 | 2.491043 | 1.675962 |
| H | -2.108224 | 2.851620 | 3.403412 |
| H | -0.899964 | 3.196632 | 2.130444 |
| C | -2.098750 | 0.221998 | 3.046172 |
| H | -1.570055 | -0.728874 | 3.127626 |
| H | -2.468293 | 0.528838 | 4.029712 |
| H | -2.913205 | 0.161182 | 2.319473 |
| C | -2.076999 | 3.030513 | -2.729071 |
| H | -3.145715 | 3.183838 | -2.543538 |
| H | -1.586443 | 4.010205 | -2.778216 |
| H | -1.963316 | 2.531049 | -3.697910 |
| H | 0.729527 | 2.315747 | -1.162369 |

Electronic Energy =  -1494.63850993 (Hartree/Particle)
Dipole Moment (Debye):      6.7992
index           0
Harmonic frequencies
Number of imaginary frequencies=            0

THERMOCHEMICAL DATA

Temperature   298.150 Kelvin.  Pressure   1.00000 Atm.
Zero-point correction=                              0.445809
(Hartree/Particle)
Thermal correction to Energy=                       0.475584
Thermal correction to Enthalpy=                     0.476528
Thermal correction to Gibbs Free Energy=            0.384442
Sum of electronic and zero-point Energies=             -1494.192701
Sum of electronic and thermal Energies=                -1494.162926
Sum of electronic and thermal Enthalpies=              -1494.161982
Sum of electronic and thermal Free Energies=           -1494.254068
PCM-SP (solvent=THF;6-311+g(2d,p)) =  -1495.01084685
Corrected Free Energy =   -1494.62640492

-------------------------------------------------------

PATH: MARKOVNIKOV

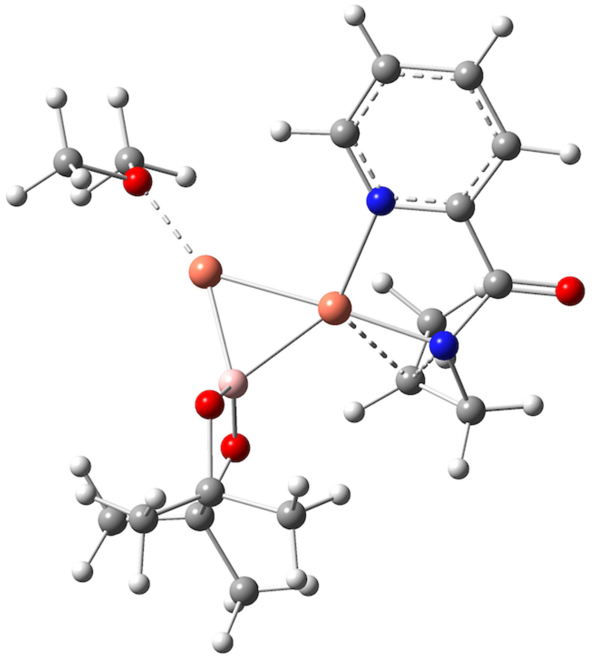

TS6          Substrate: N-(2-picolinoyl)-methyl aziridine
             Path: markovnikov

CARTESIAN COORDINATES

| | | | |
|---|---|---|---|
| C | -0.207839 | -1.111433 | 2.371665 |
| N | -1.256211 | -1.980627 | 0.910721 |
| C | -2.640289 | -1.948111 | 0.759195 |
| O | -3.432390 | -2.569946 | 1.455146 |
| C | -0.735294 | -2.488354 | 2.172693 |
| C | -3.104664 | -1.122357 | -0.417849 |
| C | -4.402095 | -1.243019 | -0.912000 |

| | | | |
|---|---|---|---|
| C | -4.769734 | -0.458747 | -2.008001 |
| C | -3.835881 | 0.422796 | -2.559042 |
| C | -2.564921 | 0.497990 | -1.986265 |
| N | -2.210552 | -0.261058 | -0.940778 |
| Cu | 0.437545 | 1.820116 | 0.175225 |
| H | -5.086838 | -1.937735 | -0.437564 |
| H | -5.768660 | -0.535792 | -2.427154 |
| H | -1.798579 | 1.164872 | -2.371501 |
| H | -4.081311 | 1.039468 | -3.417809 |
| H | -1.510656 | -2.810036 | 2.872385 |
| H | 0.019425 | -3.264003 | 2.028047 |
| Cu | -0.460280 | -0.358051 | 0.193814 |
| B | 1.575895 | 0.185087 | -0.221178 |
| O | 2.542670 | -0.164502 | 0.694833 |
| O | 1.833476 | -0.288448 | -1.483571 |
| C | 3.654995 | -0.781104 | -0.052551 |
| C | 2.937527 | -1.258266 | -1.366179 |
| H | 0.853032 | -0.935684 | 2.203752 |
| C | -0.978055 | -0.109178 | 3.165886 |
| H | -0.699108 | -0.246028 | 4.224384 |
| H | -0.733493 | 0.919644 | 2.885710 |
| C | 2.273567 | -2.633656 | -1.236696 |
| H | 1.638340 | -2.806184 | -2.110595 |
| H | 3.018330 | -3.434107 | -1.186005 |
| H | 1.638166 | -2.686288 | -0.345726 |
| C | 3.786544 | -1.185293 | -2.628915 |
| H | 4.664277 | -1.834910 | -2.534957 |
| H | 3.201488 | -1.525749 | -3.488801 |
| H | 4.125484 | -0.166975 | -2.831192 |
| C | 4.687211 | 0.325900 | -0.284997 |
| H | 4.990293 | 0.740782 | 0.681247 |
| H | 5.578394 | -0.062798 | -0.787953 |
| H | 4.271361 | 1.136990 | -0.891581 |
| C | 4.244722 | -1.897115 | 0.801391 |
| H | 5.016449 | -2.437982 | 0.242075 |
| H | 4.708840 | -1.473358 | 1.697552 |
| H | 3.481829 | -2.612436 | 1.118139 |
| H | -2.058352 | -0.265877 | 3.090474 |
| O | -0.167776 | 3.669906 | 0.538758 |
| C | -1.235795 | 4.254974 | -0.233158 |
| H | -0.826514 | 4.800894 | -1.090949 |
| H | -1.865066 | 3.431499 | -0.575153 |
| H | -1.818698 | 4.929383 | 0.403447 |
| C | 0.728137 | 4.642986 | 1.118499 |
| H | 0.162030 | 5.311173 | 1.776202 |
| H | 1.467053 | 4.087178 | 1.697643 |
| H | 1.223030 | 5.216378 | 0.326624 |

Electronic Energy = -1494.63033337 (Hartree/Particle)
Dipole Moment (Debye): 6.6498
index 1
Number of imaginary frequencies= 1
Negatives Eigenvalues: -149.298

THERMOCHEMICAL DATA

Temperature 298.150 Kelvin. Pressure 1.00000 Atm.
Zero-point correction= 0.443409 (Hartree/Particle)
Thermal correction to Energy= 0.474038

Thermal correction to Enthalpy= 0.474982
Thermal correction to Gibbs Free Energy= 0.377125
Sum of electronic and zero-point Energies= -1494.186925
Sum of electronic and thermal Energies= -1494.156296
Sum of electronic and thermal Enthalpies= -1494.155351
Sum of electronic and thermal Free Energies= -1494.253208
PCM-SP (solvent=THF;6-311+g(2d,p)) = -1495.00034032
Corrected Free Energy = -1494.62321195

---

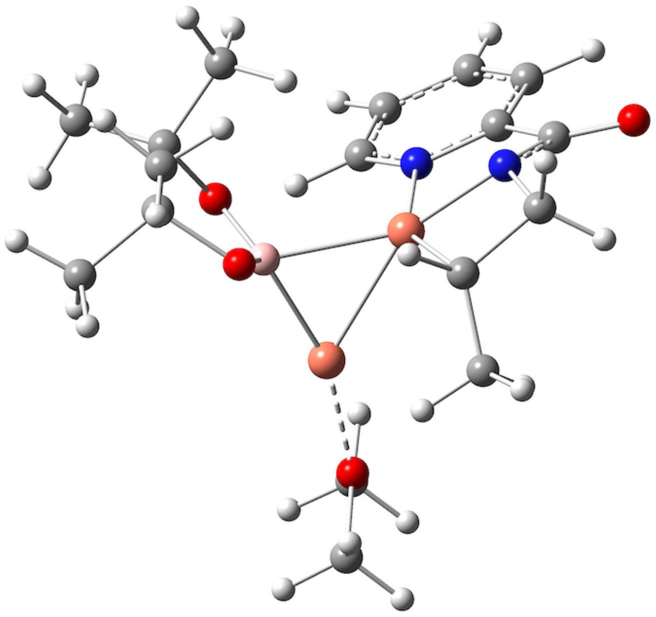

I4 Substrate: N-(2-picolinoyl)-methyl aziridine
Path: markovnikov

CARTESIAN COORDINATES

| | | | |
|---|---|---|---|
| C | -0.357889 | 0.490724 | 2.648771 |
| N | -1.809593 | -0.875464 | 1.805430 |
| C | -3.039872 | -1.069525 | 1.238066 |
| O | -4.100541 | -1.151938 | 1.844122 |
| C | -1.574261 | -0.302065 | 3.117428 |
| C | -2.934576 | -1.236452 | -0.266082 |
| C | -3.978053 | -1.742480 | -1.033700 |
| C | -3.781757 | -1.878209 | -2.411055 |
| C | -2.556353 | -1.501981 | -2.969169 |
| C | -1.564841 | -0.985581 | -2.133491 |
| N | -1.762154 | -0.854831 | -0.814331 |
| Cu | 0.484806 | 1.747456 | -0.420414 |
| H | -4.906384 | -2.018050 | -0.544085 |
| H | -4.571933 | -2.277743 | -3.040103 |
| H | -0.585071 | -0.690279 | -2.495722 |
| H | -2.365706 | -1.608013 | -4.032304 |
| H | -2.404575 | 0.327787 | 3.459406 |
| H | -1.360878 | -1.056073 | 3.883533 |
| Cu | -0.585827 | -0.066793 | 0.673700 |
| B | 1.469040 | 0.009017 | -0.128314 |
| O | 2.430936 | -0.111671 | 0.836529 |

| | | | |
|---|---|---|---|
| O | 1.579317 | -0.912136 | -1.138298 |
| C | 3.413238 | -1.103945 | 0.351664 |
| C | 2.563683 | -1.919743 | -0.689027 |
| H | 0.612248 | 0.025605 | 2.829238 |
| C | -0.399096 | 1.990898 | 2.748313 |
| H | -1.301183 | 2.409965 | 2.288970 |
| H | -0.425101 | 2.266180 | 3.815878 |
| C | 1.740045 | -3.047697 | -0.058957 |
| H | 1.035787 | -3.433872 | -0.802031 |
| H | 2.379901 | -3.871827 | 0.271309 |
| H | 1.161179 | -2.688516 | 0.799227 |
| C | 3.335576 | -2.421652 | -1.901717 |
| H | 4.130810 | -3.107130 | -1.587661 |
| H | 2.662812 | -2.967337 | -2.570721 |
| H | 3.784953 | -1.601077 | -2.465225 |
| C | 4.556465 | -0.305746 | -0.280413 |
| H | 4.965709 | 0.383546 | 0.464318 |
| H | 5.361175 | -0.967438 | -0.616185 |
| H | 4.208565 | 0.281368 | -1.136670 |
| C | 3.909262 | -1.906942 | 1.547605 |
| H | 4.571820 | -2.714005 | 1.215011 |
| H | 4.476926 | -1.257141 | 2.220759 |
| H | 3.083867 | -2.345585 | 2.113007 |
| H | 0.484272 | 2.454017 | 2.298235 |
| O | 0.021816 | 3.576142 | -0.971194 |
| C | -0.639535 | 3.844954 | -2.226717 |
| H | -1.402509 | 4.616094 | -2.076981 |
| H | 0.093408 | 4.171816 | -2.972489 |
| H | -1.109496 | 2.912777 | -2.543604 |
| C | 0.659228 | 4.735891 | -0.391944 |
| H | 1.444747 | 5.103322 | -1.061239 |
| H | -0.091624 | 5.514128 | -0.219567 |
| H | 1.093114 | 4.419371 | 0.557556 |

Electronic Energy = -1494.63510643 (Hartree/Particle)
Dipole Moment (Debye): 7.3136
index 0
Harmonic frequencies
Number of imaginary frequencies= 0

THERMOCHEMICAL DATA

Temperature 298.150 Kelvin. Pressure 1.00000 Atm.
Zero-point correction= 0.444520 (Hartree/Particle)
Thermal correction to Energy= 0.475391
Thermal correction to Enthalpy= 0.476335
Thermal correction to Gibbs Free Energy= 0.378135
Sum of electronic and zero-point Energies= -1494.190586
Sum of electronic and thermal Energies= -1494.159715
Sum of electronic and thermal Enthalpies= -1494.158771
Sum of electronic and thermal Free Energies= -1494.256972
PCM-SP (solvent=THF;6-311+g(2d,p)) = -1495.00722031
Corrected Free Energy = -1494.62908588

---

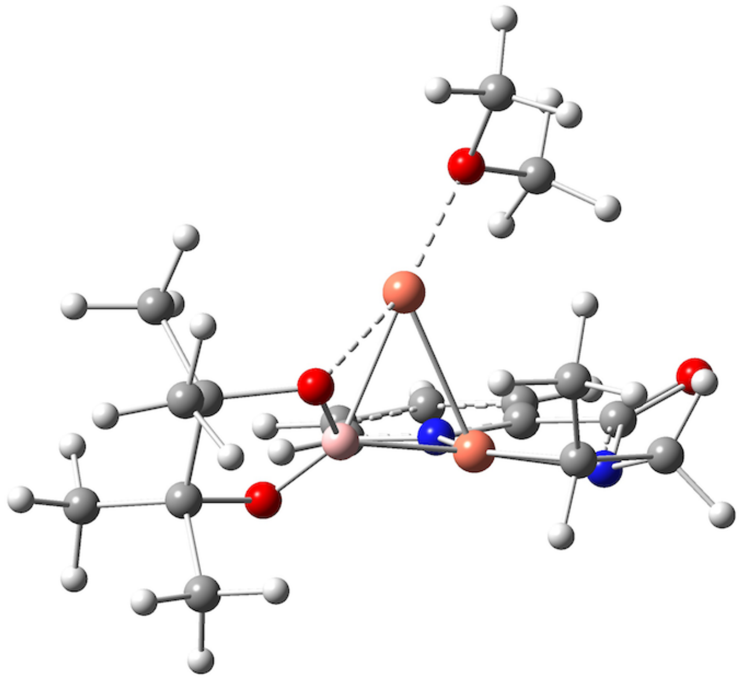

TS7 Substrate: N-(2-picolinoyl)-methyl aziridine
Path: markovnikov

CARTESIAN COORDINATES

| | | | |
|---|---|---|---|
| N | -1.591252 | 1.537604 | -0.111311 |
| C | -2.915977 | 1.277911 | -0.037970 |
| C | -3.849470 | 2.030045 | -0.742960 |
| C | -3.394139 | 3.103632 | -1.513588 |
| C | -2.023728 | 3.376030 | -1.569155 |
| C | -1.144791 | 2.557762 | -0.859103 |
| C | -3.297987 | 0.082106 | 0.823876 |
| O | -4.362111 | -0.516763 | 0.670703 |
| Cu | -0.607052 | 0.125341 | 0.986720 |
| N | -2.302648 | -0.200995 | 1.700358 |
| C | -2.101730 | -1.487937 | 2.354725 |
| C | -0.573848 | -1.510556 | 2.218384 |
| B | 1.354694 | 0.277164 | 0.428169 |
| Cu | 0.413875 | -1.317192 | -0.729786 |
| O | 2.276754 | -0.739005 | 0.161304 |
| C | 3.511188 | -0.086433 | -0.374204 |
| C | 4.712858 | -0.884832 | 0.108310 |
| O | 1.905767 | 1.516272 | 0.338504 |
| C | 3.374811 | 1.356516 | 0.227484 |
| C | 3.930458 | 1.484012 | 1.647769 |
| C | 3.387197 | -0.139685 | -1.897509 |
| C | 3.904895 | 2.470608 | -0.666192 |
| H | -4.899163 | 1.764239 | -0.671618 |
| H | -4.098613 | 3.721605 | -2.062871 |
| H | -0.069253 | 2.706705 | -0.864758 |
| H | -1.637410 | 4.206010 | -2.151900 |
| H | 4.274522 | 0.276168 | -2.383904 |
| H | 3.286102 | -1.184198 | -2.211440 |
| H | 2.508560 | 0.415148 | -2.245277 |
| H | 4.711556 | -1.878395 | -0.350549 |
| H | 5.640502 | -0.378260 | -0.180934 |

| | | | |
|---|---|---|---|
| H | 4.708274 | -1.009937 | 1.192759 |
| H | 3.759632 | 3.437034 | -0.174486 |
| H | 4.978128 | 2.337552 | -0.843458 |
| H | 3.392634 | 2.499216 | -1.630719 |
| H | 5.024058 | 1.438984 | 1.649942 |
| H | 3.624021 | 2.447746 | 2.064450 |
| H | 3.547894 | 0.693862 | 2.302012 |
| O | -0.757111 | -2.426913 | -1.819069 |
| C | -0.381420 | -2.849147 | -3.148787 |
| H | -0.648082 | -2.075912 | -3.878157 |
| H | 0.697965 | -3.005530 | -3.138598 |
| H | -0.892338 | -3.787989 | -3.385964 |
| C | -2.186526 | -2.247252 | -1.652459 |
| H | -2.698202 | -3.177263 | -1.918286 |
| H | -2.361374 | -2.013830 | -0.603352 |
| H | -2.536236 | -1.422409 | -2.282299 |
| H | -2.603761 | -2.310758 | 1.829976 |
| H | -0.166766 | -2.356778 | 1.661201 |
| C | 0.240912 | -1.126482 | 3.429535 |
| H | 0.133938 | -1.918913 | 4.188091 |
| H | 1.304019 | -1.022386 | 3.194680 |
| H | -0.117220 | -0.195341 | 3.880580 |
| H | -2.440048 | -1.491288 | 3.399123 |

Electronic Energy = -1494.62566448 (Hartree/Particle)
Dipole Moment (Debye): 6.8825
index 0
Harmonic frequencies
Number of imaginary frequencies= 1
Negatives Eigenvalues: -59.462

THERMOCHEMICAL DATA

Temperature 298.150 Kelvin. Pressure 1.00000 Atm.
Zero-point correction= 0.444587
(Hartree/Particle)
Thermal correction to Energy= 0.474370
Thermal correction to Enthalpy= 0.475314
Thermal correction to Gibbs Free Energy= 0.381934
Sum of electronic and zero-point Energies= -1494.181077
Sum of electronic and thermal Energies= -1494.151295
Sum of electronic and thermal Enthalpies= -1494.150351
Sum of electronic and thermal Free Energies= -1494.243731
PCM-SP (solvent=THF;6-311+g(2d,p)) = -1494.99785619
Corrected Free Energy = -1494.61592271

-------------------------------------------------------

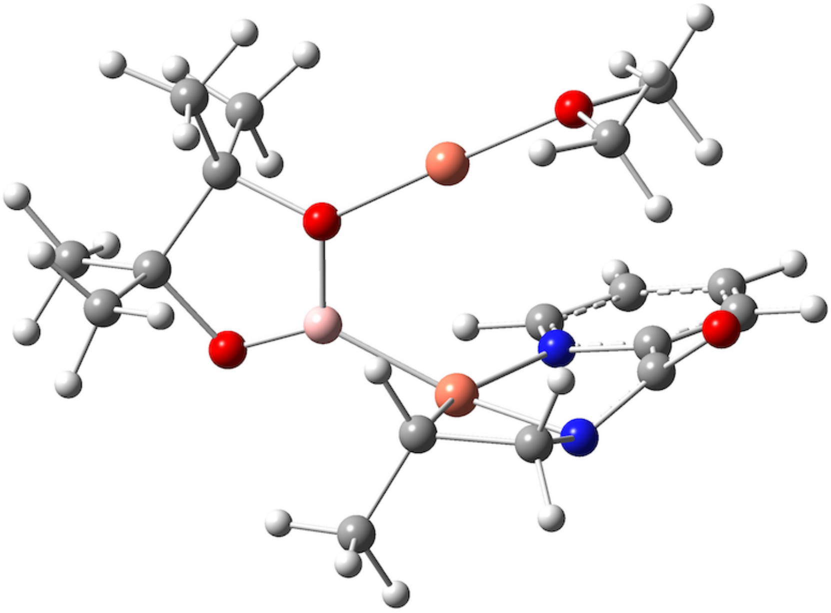

I5 Substrate: N-(2-picolinoyl)-methyl aziridine
Path: markovnikov

CARTESIAN COORDINATES

| | | | |
|---|---|---|---|
| N | 1.627319 | 1.413291 | 0.948592 |
| C | 2.809399 | 0.923798 | 0.502038 |
| C | 3.916954 | 0.813098 | 1.337781 |
| C | 3.809360 | 1.259183 | 2.657036 |
| C | 2.589732 | 1.774491 | 3.107582 |
| C | 1.514402 | 1.824378 | 2.221765 |
| C | 2.801745 | 0.434396 | -0.942610 |
| O | 3.526810 | -0.515149 | -1.282304 |
| Cu | 0.318347 | 1.318076 | -0.611509 |
| N | 1.888566 | 1.109135 | -1.659743 |
| C | 1.233792 | 0.552823 | -2.847430 |
| C | -0.239100 | 0.828294 | -2.473132 |
| B | -1.482074 | 0.846121 | -0.018148 |
| Cu | -0.185738 | -1.702856 | 0.099528 |
| O | -1.671105 | -0.546841 | 0.234083 |
| C | -2.999604 | -0.730145 | 0.911462 |
| C | -3.583639 | -2.056304 | 0.452871 |
| O | -2.633252 | 1.513691 | 0.203163 |
| C | -3.738346 | 0.554249 | 0.400583 |
| C | -4.396175 | 0.373366 | -0.969732 |
| C | -2.708214 | -0.735204 | 2.410394 |
| C | -4.717401 | 1.153968 | 1.401721 |
| H | 4.834301 | 0.392156 | 0.939136 |
| H | 4.663713 | 1.207806 | 3.325964 |
| H | 0.541075 | 2.202804 | 2.518669 |
| H | 2.469266 | 2.132541 | 4.124852 |
| H | 1.528361 | 1.074953 | -3.767162 |
| H | -3.623204 | -0.904972 | 2.985617 |
| H | -2.007699 | -1.545968 | 2.640064 |
| H | -2.262130 | 0.209543 | 2.734962 |
| H | -2.974254 | -2.884938 | 0.831389 |

| | | | |
|---|---|---|---|
| H | -4.596449 | -2.178500 | 0.851411 |
| H | -3.628030 | -2.128637 | -0.635767 |
| H | -5.180854 | 2.045238 | 0.969204 |
| H | -5.511567 | 0.436373 | 1.636952 |
| H | -4.221684 | 1.447823 | 2.329420 |
| H | -5.273291 | -0.278411 | -0.907222 |
| H | -4.719265 | 1.351043 | -1.338018 |
| H | -3.696641 | -0.049597 | -1.698702 |
| H | 1.453059 | -0.514812 | -2.974850 |
| O | 1.313575 | -2.839247 | -0.081794 |
| C | 1.746176 | -3.267299 | -1.405794 |
| H | 2.457277 | -2.538571 | -1.804251 |
| H | 2.201338 | -4.258378 | -1.314443 |
| H | 0.849405 | -3.327285 | -2.024168 |
| C | 2.412684 | -2.766437 | 0.867854 |
| H | 1.990764 | -2.426442 | 1.814732 |
| H | 2.833451 | -3.770048 | 0.985246 |
| H | 3.163312 | -2.064810 | 0.494764 |
| H | -0.873195 | -0.061969 | -2.474843 |
| C | -0.886963 | 2.030090 | -3.127885 |
| H | -1.011968 | 1.829757 | -4.203449 |
| H | -1.869725 | 2.252060 | -2.702218 |
| H | -0.258885 | 2.922188 | -3.033276 |

Electronic Energy = -1494.63626144 (Hartree/Particle)
Dipole Moment (Debye): 6.3941
index 0
Harmonic frequencies
Number of imaginary frequencies= 0

THERMOCHEMICAL DATA

Temperature 298.150 Kelvin. Pressure 1.00000 Atm.
Zero-point correction= 0.446040
(Hartree/Particle)
Thermal correction to Energy= 0.475915
Thermal correction to Enthalpy= 0.476860
Thermal correction to Gibbs Free Energy= 0.384339
Sum of electronic and zero-point Energies= -1494.190221
Sum of electronic and thermal Energies= -1494.160346
Sum of electronic and thermal Enthalpies= -1494.159402
Sum of electronic and thermal Free Energies= -1494.251923
PCM-SP (solvent=THF;6-311+g(2d,p)) = -1495.00833143
Corrected Free Energy = -1494.62399296

----------------------------------------------------------------
----------------------------------------------------------------

MODEL: [Az-(Cu)2-Bpin]+

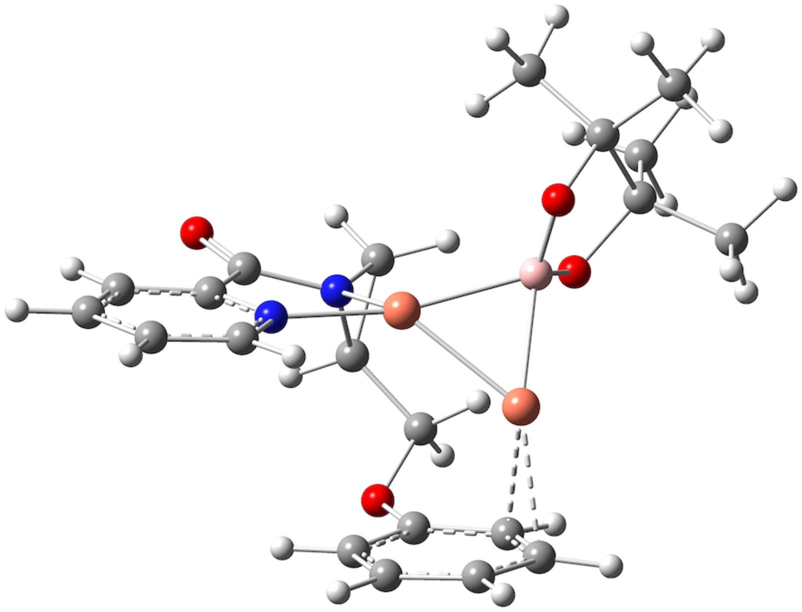

SUBSTRATE: 1c

Complex-4 Substrate: 1c

CARTESIAN COORDINATES

| | | | |
|---|---|---|---|
| C | -0.752006 | -0.888987 | 2.640171 |
| N | -0.723731 | -1.508100 | 1.256852 |
| C | -1.831681 | -2.383315 | 0.993373 |
| O | -2.083445 | -3.375334 | 1.643643 |
| C | 0.227233 | -1.943225 | 2.309504 |
| C | -2.664487 | -1.962356 | -0.183873 |
| C | -3.893055 | -2.580490 | -0.416932 |
| C | -4.649106 | -2.172392 | -1.517892 |
| C | -4.145894 | -1.169037 | -2.347536 |
| C | -2.901776 | -0.608245 | -2.050518 |
| N | -2.174349 | -0.992845 | -0.990466 |
| Cu | 0.535840 | 1.854037 | -0.589879 |
| H | -4.229995 | -3.359860 | 0.257974 |
| H | -5.610827 | -2.632135 | -1.724887 |
| H | -2.461207 | 0.165344 | -2.670810 |
| H | -4.697968 | -0.826064 | -3.216699 |
| H | 0.069069 | -2.936844 | 2.719276 |
| H | 1.247587 | -1.650900 | 2.078128 |
| H | -1.614743 | -1.190585 | 3.231554 |
| C | -0.371901 | 0.564190 | 2.739267 |
| H | -0.094320 | 0.801741 | 3.772332 |
| H | 0.475554 | 0.771198 | 2.079813 |
| O | -1.524208 | 1.354828 | 2.384622 |
| C | -1.426943 | 2.256444 | 1.369414 |
| C | -2.537511 | 2.394180 | 0.527561 |
| C | -0.271907 | 3.039374 | 1.142745 |
| C | -2.481158 | 3.247135 | -0.578666 |
| H | -3.422383 | 1.802133 | 0.738083 |
| C | -0.219733 | 3.890552 | 0.012176 |
| H | 0.540671 | 3.038307 | 1.863101 |
| C | -1.320968 | 3.976376 | -0.862607 |

| | | | |
|---|---|---|---|
| H | -3.345711 | 3.332332 | -1.230633 |
| H | 0.637822 | 4.545979 | -0.122159 |
| H | -1.276081 | 4.631672 | -1.726204 |
| Cu | -0.326354 | -0.308026 | -0.534819 |
| B | 1.707464 | 0.122023 | -0.577131 |
| O | 2.435831 | 0.037622 | 0.601424 |
| O | 2.360311 | -0.455787 | -1.636602 |
| C | 3.785269 | -0.440057 | 0.249650 |
| C | 3.517451 | -1.189166 | -1.103190 |
| C | 4.659328 | 0.808726 | 0.095346 |
| H | 4.631539 | 1.382638 | 1.026995 |
| H | 5.700233 | 0.542269 | -0.113692 |
| H | 4.295447 | 1.449783 | -0.714124 |
| C | 4.303181 | -1.316873 | 1.383729 |
| H | 5.278195 | -1.742281 | 1.120707 |
| H | 4.429631 | -0.716265 | 2.290366 |
| H | 3.621984 | -2.140654 | 1.610474 |
| C | 4.651765 | -1.113760 | -2.118206 |
| H | 5.559076 | -1.575247 | -1.711644 |
| H | 4.371650 | -1.655056 | -3.027076 |
| H | 4.877831 | -0.081999 | -2.395900 |
| C | 3.054554 | -2.638808 | -0.916649 |
| H | 2.692911 | -3.022416 | -1.875331 |
| H | 3.873152 | -3.279875 | -0.574152 |
| H | 2.233188 | -2.703259 | -0.194178 |

Electronic Energy = -1645.88840862 (Hartree/Particle)
Dipole Moment (Debye): 3.6725
index 0
Harmonic frequencies
Number of imaginary frequencies= 0

THERMOCHEMICAL DATA

Temperature 298.150 Kelvin. Pressure 1.00000 Atm.
Zero-point correction= 0.449779
(Hartree/Particle)
Thermal correction to Energy= 0.479705
Thermal correction to Enthalpy= 0.480649
Thermal correction to Gibbs Free Energy= 0.387147
Sum of electronic and zero-point Energies= -1645.438629
Sum of electronic and thermal Energies= -1645.408704
Sum of electronic and thermal Enthalpies= -1645.407759
Sum of electronic and thermal Free Energies= -1645.501262
PCM-SP (solvent=THF;6-311+g(2d,p)) = -1646.28883721
Corrected Free Energy = -1645.90169059

------------------------------------------------------

PATH: ANTI_MARKOVNIKOV

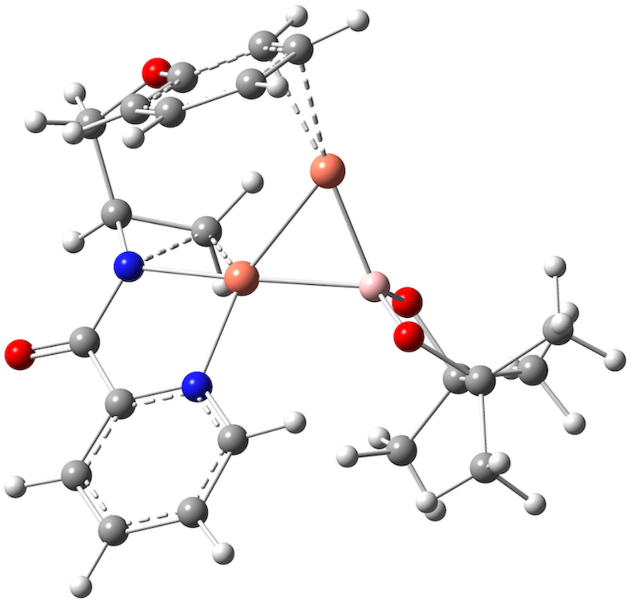

TS8 Substrate: 1c
Path: anti-markovnikov

CARTESIAN COORDINATES

| | | | |
|---|---|---|---|
| C | -1.277074 | 0.149042 | 2.278648 |
| N | -1.854696 | 1.299599 | 0.810837 |
| C | -1.640396 | 2.680957 | 0.672199 |
| O | -2.326525 | 3.523218 | 1.229023 |
| C | -2.567346 | 0.835535 | 1.989605 |
| C | -0.548881 | 3.036966 | -0.303171 |
| C | -0.360611 | 4.350409 | -0.725053 |
| C | 0.675869 | 4.612387 | -1.626511 |
| C | 1.485642 | 3.559950 | -2.058088 |
| C | 1.240543 | 2.272662 | -1.570855 |
| N | 0.239217 | 2.024814 | -0.717163 |
| Cu | -0.325657 | -1.971591 | -0.458528 |
| H | -1.011819 | 5.130347 | -0.344861 |
| H | 0.848500 | 5.623455 | -1.983365 |
| H | 1.843507 | 1.413943 | -1.850620 |
| H | 2.296981 | 3.725459 | -2.759752 |
| H | -2.832336 | 1.652986 | 2.667214 |
| H | -0.530535 | 0.681534 | 2.859852 |
| Cu | -0.321519 | 0.272325 | 0.252444 |
| B | 1.417622 | -0.983334 | 0.079124 |
| O | 2.369566 | -0.759766 | -0.889861 |
| O | 1.955733 | -1.056744 | 1.344820 |
| C | 3.692109 | -0.907189 | -0.256103 |
| C | 3.363276 | -0.633654 | 1.254940 |
| H | -1.199309 | -0.931678 | 2.218539 |
| C | -3.796407 | -0.040742 | 1.730080 |
| H | -4.320224 | -0.206185 | 2.674236 |
| H | -4.482345 | 0.463891 | 1.041589 |
| O | -3.470203 | -1.365789 | 1.286235 |
| C | -3.086709 | -1.632951 | 0.008661 |
| C | -2.390104 | -2.855323 | -0.153411 |
| C | -3.327754 | -0.818266 | -1.104073 |
| C | -1.907129 | -3.227485 | -1.426612 |
| H | -2.300850 | -3.519888 | 0.701404 |

| | | | |
|---|---|---|---|
| C | -2.838197 | -1.195802 | -2.361604 |
| H | -3.857996 | 0.119762 | -0.997592 |
| C | -2.114142 | -2.378107 | -2.534592 |
| H | -1.445542 | -4.203187 | -1.561062 |
| H | -3.021181 | -0.546662 | -3.212803 |
| H | -1.738013 | -2.659272 | -3.512433 |
| C | 4.181533 | -1.444821 | 2.252135 |
| H | 5.247532 | -1.211882 | 2.149109 |
| H | 3.876050 | -1.195563 | 3.273137 |
| H | 4.043620 | -2.518775 | 2.110262 |
| C | 3.378995 | 0.853969 | 1.625747 |
| H | 2.954352 | 0.975687 | 2.627192 |
| H | 4.397967 | 1.253534 | 1.634701 |
| H | 2.777604 | 1.444122 | 0.925928 |
| C | 4.147135 | -2.342874 | -0.534891 |
| H | 4.159273 | -2.510012 | -1.616219 |
| H | 5.154456 | -2.522979 | -0.146049 |
| H | 3.464687 | -3.070925 | -0.084677 |
| C | 4.647825 | 0.091704 | -0.898940 |
| H | 5.623053 | 0.062140 | -0.400064 |
| H | 4.797838 | -0.165010 | -1.952411 |
| H | 4.265452 | 1.114095 | -0.846585 |

Electronic Energy = -1645.85590322 (Hartree/Particle)
Dipole Moment (Debye): 2.0153
index 1
Number of imaginary frequencies= 1
Negatives Eigenvalues: -217.650

THERMOCHEMICAL DATA

Temperature 298.150 Kelvin. Pressure 1.00000 Atm.
Zero-point correction= 0.448034
(Hartree/Particle)
Thermal correction to Energy= 0.477323
Thermal correction to Enthalpy= 0.478267
Thermal correction to Gibbs Free Energy= 0.387322
Sum of electronic and zero-point Energies= -1645.407869
Sum of electronic and thermal Energies= -1645.378580
Sum of electronic and thermal Enthalpies= -1645.377636
Sum of electronic and thermal Free Energies= -1645.468581
PCM-SP (solvent=THF;6-311+g(2d,p)) = -1646.25760073
Corrected Free Energy = -1645.87027851

---

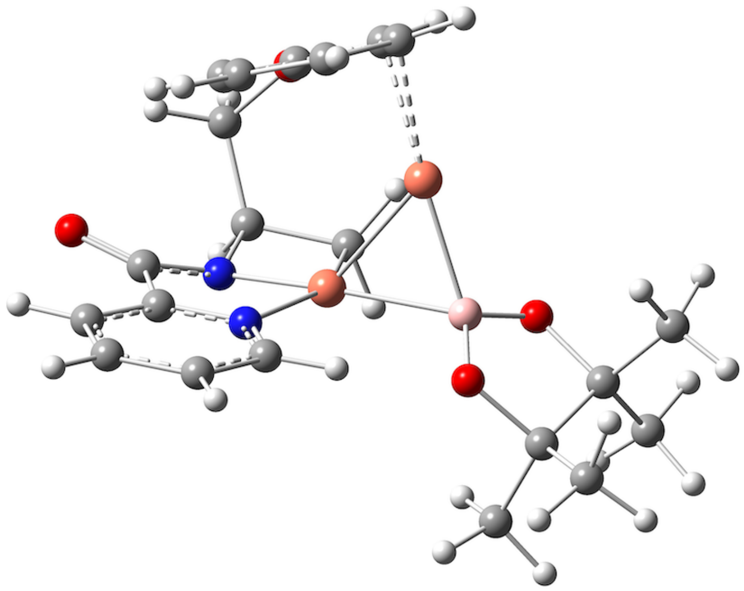

I6 Substrate: 1c
Path: anti-markovnikov

CARTESIAN COORDINATES

| | | | |
|---|---|---|---|
| C | 1.767079 | -0.951000 | 3.032536 |
| C | 1.035503 | -2.092204 | 2.639836 |
| C | 1.279393 | -2.688790 | 1.379925 |
| C | 2.260972 | -2.136945 | 0.513205 |
| C | 3.030585 | -1.048557 | 0.945850 |
| C | 2.770333 | -0.462663 | 2.190903 |
| O | 2.378861 | -2.754096 | -0.684820 |
| C | 2.822173 | -2.055014 | -1.870897 |
| C | 1.763436 | -1.090717 | -2.453697 |
| Cu | 0.170367 | 0.141797 | -0.811250 |
| B | -1.807140 | -0.184354 | 0.018670 |
| O | -2.684467 | -1.087722 | -0.509987 |
| C | -4.046551 | -0.559165 | -0.277293 |
| C | -3.775387 | 0.981531 | -0.118490 |
| O | -2.386633 | 0.996511 | 0.398479 |
| Cu | -0.285272 | -1.174645 | 1.080928 |
| C | 0.314629 | -1.427363 | -2.074194 |
| N | 1.691249 | 0.238478 | -1.860256 |
| C | 2.672767 | 1.056243 | -1.411480 |
| C | 2.119501 | 2.087336 | -0.445807 |
| C | 2.866378 | 3.177242 | -0.016027 |
| C | 2.288624 | 4.059134 | 0.902671 |
| C | 0.992126 | 3.814664 | 1.362490 |
| C | 0.307616 | 2.691684 | 0.894437 |
| N | 0.868678 | 1.853600 | 0.010148 |
| O | 3.868475 | 0.988837 | -1.692129 |
| H | 3.871122 | 3.309830 | -0.404314 |
| H | 2.842200 | 4.925219 | 1.253689 |
| H | -0.701835 | 2.444774 | 1.205125 |
| H | 0.511377 | 4.479487 | 2.072767 |
| H | 1.934322 | -1.042479 | -3.537944 |
| H | -0.442975 | -1.179274 | -2.817603 |
| H | 0.118615 | -2.348443 | -1.532926 |
| H | 2.994650 | -2.865416 | -2.581233 |
| H | 3.766527 | -1.531671 | -1.703063 |

| | | | |
|---|---|---|---|
| H | 0.799458 | -3.621063 | 1.091303 |
| H | 3.811300 | -0.642354 | 0.313052 |
| H | 0.348815 | -2.570083 | 3.335379 |
| H | 3.365438 | 0.391543 | 2.501049 |
| H | 1.577694 | -0.489587 | 3.995960 |
| C | -4.550461 | -1.229791 | 1.003774 |
| H | -5.582454 | -0.938397 | 1.222623 |
| H | -4.519541 | -2.315792 | 0.874443 |
| H | -3.925696 | -0.968544 | 1.864552 |
| C | -4.918299 | -0.941210 | -1.466719 |
| H | -5.040434 | -2.027965 | -1.502393 |
| H | -5.912040 | -0.489837 | -1.368287 |
| H | -4.480354 | -0.617123 | -2.413264 |
| C | -4.670887 | 1.694902 | 0.885752 |
| H | -4.406125 | 2.755875 | 0.932480 |
| H | -5.719499 | 1.621305 | 0.576343 |
| H | -4.573438 | 1.274041 | 1.888848 |
| C | -3.735141 | 1.739381 | -1.448354 |
| H | -4.735500 | 1.826295 | -1.883659 |
| H | -3.346474 | 2.747583 | -1.275561 |
| H | -3.082939 | 1.241458 | -2.173510 |

Electronic Energy = -1645.86109934 (Hartree/Particle)
Dipole Moment (Debye): 4.5232
index 0
Harmonic frequencies
Number of imaginary frequencies= 0

THERMOCHEMICAL DATA

Temperature 298.150 Kelvin. Pressure 1.00000 Atm.
Zero-point correction= 0.449619
(Hartree/Particle)
Thermal correction to Energy= 0.478914
Thermal correction to Enthalpy= 0.479858
Thermal correction to Gibbs Free Energy= 0.389413
Sum of electronic and zero-point Energies= -1645.411480
Sum of electronic and thermal Energies= -1645.382185
Sum of electronic and thermal Enthalpies= -1645.381241
Sum of electronic and thermal Free Energies= -1645.471686
PCM-SP (solvent=THF;6-311+g(2d,p)) = -1646.26400759
Corrected Free Energy = -1645.87459425

-------------------------------------------------------

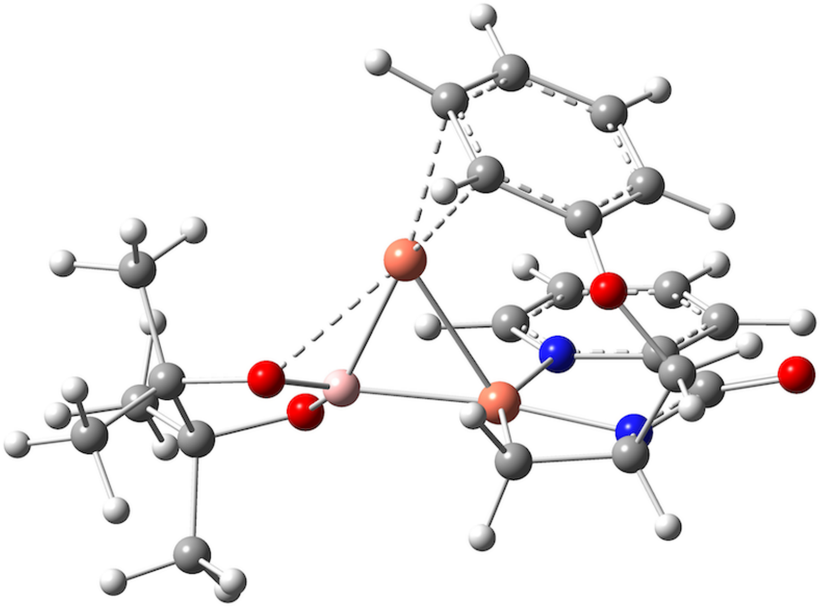

TS9 Substrate: 1c
Path: anti-markovnikov

CARTESIAN COORDINATES

| | | | |
|---|---|---|---|
| N | 0.980383 | 1.907399 | 0.001226 |
| C | 2.304504 | 2.038867 | -0.242523 |
| C | 3.053556 | 3.066137 | 0.317922 |
| C | 2.405402 | 3.994029 | 1.138924 |
| C | 1.034843 | 3.858486 | 1.374067 |
| C | 0.350477 | 2.792986 | 0.787385 |
| C | 2.926147 | 0.973514 | -1.131166 |
| O | 4.144960 | 0.824751 | -1.225917 |
| Cu | 0.277715 | 0.268838 | -0.951907 |
| N | 1.961026 | 0.238917 | -1.720737 |
| C | 1.996916 | -1.063031 | -2.370089 |
| C | 0.483799 | -1.298119 | -2.212571 |
| C | 2.895454 | -2.139040 | -1.720988 |
| O | 2.261450 | -2.842107 | -0.627205 |
| C | 2.039887 | -2.251971 | 0.568403 |
| C | 0.921635 | -2.756060 | 1.288990 |
| C | 0.562179 | -2.170591 | 2.528919 |
| C | 1.315476 | -1.096006 | 3.045358 |
| C | 2.450787 | -0.664890 | 2.352772 |
| C | 2.824267 | -1.235878 | 1.130724 |
| Cu | -0.473932 | -1.193648 | 0.804863 |
| O | -2.572137 | -1.002215 | -0.359773 |
| C | -3.916106 | -0.494263 | 0.025031 |
| C | -4.964483 | -1.311508 | -0.716740 |
| B | -1.722900 | 0.084580 | -0.376139 |
| O | -2.365234 | 1.280832 | -0.263284 |
| C | -3.817974 | 1.014338 | -0.402057 |
| C | -4.160702 | 1.260928 | -1.872900 |
| C | -4.025143 | -0.703417 | 1.537265 |
| C | -4.565920 | 1.986094 | 0.501406 |
| H | 4.115453 | 3.116705 | 0.099302 |
| H | 2.961321 | 4.813121 | 1.585961 |
| H | -0.713996 | 2.634013 | 0.922671 |
| H | 0.497458 | 4.563644 | 1.999791 |

| | | | |
|---|---|---|---|
| H | 2.311692 | -0.980840 | -3.420230 |
| H | -0.129081 | -0.993868 | -3.061818 |
| H | 0.153726 | -2.225886 | -1.752346 |
| H | 3.083336 | -2.934024 | -2.444781 |
| H | 3.852823 | -1.705311 | -1.421362 |
| H | 0.434315 | -3.657925 | 0.924103 |
| H | 3.704294 | -0.871608 | 0.613525 |
| H | -0.235166 | -2.615897 | 3.119777 |
| H | 3.061674 | 0.133612 | 2.764258 |
| H | 1.039011 | -0.644888 | 3.992530 |
| H | -5.008539 | -0.401835 | 1.910308 |
| H | -3.885069 | -1.765364 | 1.762570 |
| H | -3.260105 | -0.131505 | 2.074577 |
| H | -4.948816 | -2.347004 | -0.363393 |
| H | -5.963370 | -0.902307 | -0.527762 |
| H | -4.787845 | -1.316547 | -1.794140 |
| H | -4.432964 | 3.008536 | 0.135011 |
| H | -5.638236 | 1.760360 | 0.494867 |
| H | -4.207446 | 1.943263 | 1.532481 |
| H | -5.233994 | 1.142796 | -2.051923 |
| H | -3.877319 | 2.283381 | -2.139382 |
| H | -3.620534 | 0.574033 | -2.532401 |

Electronic Energy = -1645.86006202 (Hartree/Particle)
Dipole Moment (Debye): 5.4811
index 0
Harmonic frequencies
Number of imaginary frequencies= 1
Negatives Eigenvalues: -31.689

THERMOCHEMICAL DATA

Temperature 298.150 Kelvin. Pressure 1.00000 Atm.
Zero-point correction= 0.449488
(Hartree/Particle)
Thermal correction to Energy= 0.477931
Thermal correction to Enthalpy= 0.478875
Thermal correction to Gibbs Free Energy= 0.391230
Sum of electronic and zero-point Energies= -1645.410574
Sum of electronic and thermal Energies= -1645.382131
Sum of electronic and thermal Enthalpies= -1645.381187
Sum of electronic and thermal Free Energies= -1645.468832
PCM-SP (solvent=THF;6-311+g(2d,p)) = -1646.26274602
Corrected Free Energy = -1645.87151600

-------------------------------------------------------

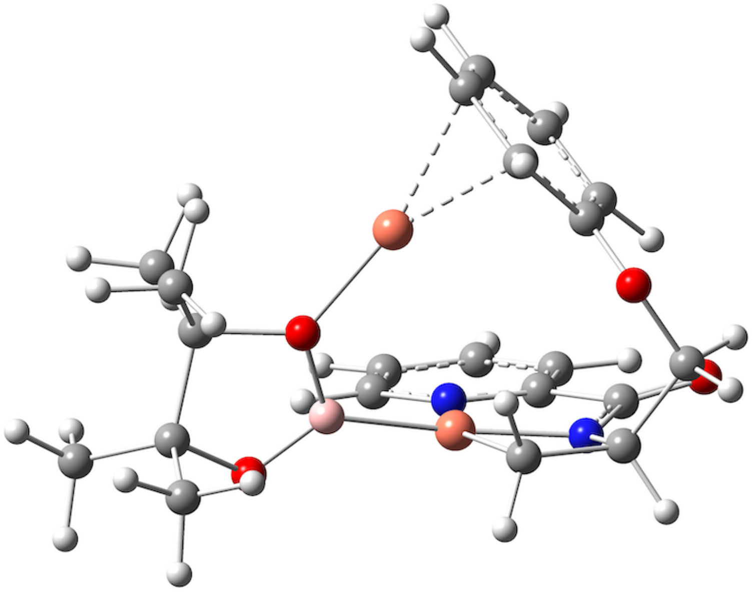

I7 Substrate: 1c
Path: anti-markovnikov

CARTESIAN COORDINATES

| | | | |
|---|---|---|---|
| N | 1.323786 | -2.078771 | -0.246752 |
| C | 2.666406 | -1.894885 | -0.181564 |
| C | 3.540813 | -2.621378 | -0.981687 |
| C | 3.017370 | -3.579132 | -1.854764 |
| C | 1.634563 | -3.772759 | -1.905967 |
| C | 0.817203 | -2.995438 | -1.085445 |
| C | 3.160862 | -0.829299 | 0.795439 |
| O | 4.323782 | -0.411191 | 0.763951 |
| Cu | 0.408279 | -0.838101 | 1.057134 |
| N | 2.142415 | -0.445324 | 1.571418 |
| C | 1.920724 | 0.659521 | 2.490742 |
| C | 0.372327 | 0.566223 | 2.474035 |
| C | 2.512082 | 2.029495 | 2.096743 |
| O | 1.645216 | 2.821533 | 1.248361 |
| C | 1.492941 | 2.585728 | -0.066151 |
| C | 0.382424 | 3.259728 | -0.664173 |
| C | 0.121940 | 3.087368 | -2.052014 |
| C | 0.946985 | 2.253367 | -2.829939 |
| C | 2.049245 | 1.640519 | -2.228477 |
| C | 2.331073 | 1.796897 | -0.865512 |
| Cu | -1.002117 | 1.728850 | -0.677451 |
| O | -2.098605 | 0.301640 | -0.018178 |
| C | -3.479145 | -0.088086 | -0.459443 |
| C | -4.357292 | 1.151851 | -0.403149 |
| B | -1.520627 | -0.800369 | 0.675197 |
| O | -2.465521 | -1.739232 | 0.912464 |
| C | -3.801176 | -1.200804 | 0.593973 |
| C | -4.369791 | -0.652890 | 1.905582 |
| C | -3.330341 | -0.600768 | -1.890796 |
| C | -4.661834 | -2.339888 | 0.061678 |
| H | 4.604564 | -2.423280 | -0.896932 |
| H | 3.678411 | -4.169066 | -2.483152 |
| H | -0.262973 | -3.102012 | -1.079827 |
| H | 1.190954 | -4.511567 | -2.565411 |

```
 H              2.333362    0.423746    3.482470
 H             -0.076723    0.118427    3.361245
 H             -0.161361    1.458586    2.149137
 H              2.606647    2.653440    2.987117
 H              3.501214    1.904775    1.647544
 H             -0.112314    4.040588   -0.087771
 H              3.206915    1.319967   -0.443042
 H             -0.652636    3.686276   -2.525979
 H              2.716978    1.027443   -2.827503
 H              0.749785    2.127238   -3.889248
 H             -4.303184   -0.866507   -2.314894
 H             -2.891482    0.186887   -2.513989
 H             -2.677892   -1.477759   -1.936473
 H             -4.025980    1.879598   -1.153356
 H             -5.395642    0.888797   -0.631523
 H             -4.327147    1.629416    0.578216
 H             -4.829120   -3.072410    0.856477
 H             -5.637453   -1.962204   -0.264713
 H             -4.185489   -2.853499   -0.776372
 H             -5.393984   -0.289699    1.773439
 H             -4.382020   -1.456010    2.647677
 H             -3.755660    0.163411    2.299944

 Electronic Energy =  -1645.86850731 (Hartree/Particle)
 Dipole Moment (Debye):      7.3570
 index          0
 Harmonic frequencies
 Number of imaginary frequencies=            0

 THERMOCHEMICAL DATA

 Temperature   298.150 Kelvin.  Pressure   1.00000 Atm.
 Zero-point correction=                           0.449876
(Hartree/Particle)
 Thermal correction to Energy=                    0.479002
 Thermal correction to Enthalpy=                  0.479946
 Thermal correction to Gibbs Free Energy=         0.389496
 Sum of electronic and zero-point Energies=          -1645.418631
 Sum of electronic and thermal Energies=             -1645.389506
 Sum of electronic and thermal Enthalpies=           -1645.388562
 Sum of electronic and thermal Free Energies=        -1645.479011
 PCM-SP (solvent=THF;6-311+g(2d,p)) =   -1646.27329686
 Corrected Free Energy =  -1645.88380055

------------------------------------------------------

PATH: MARKOVNIKOV
```

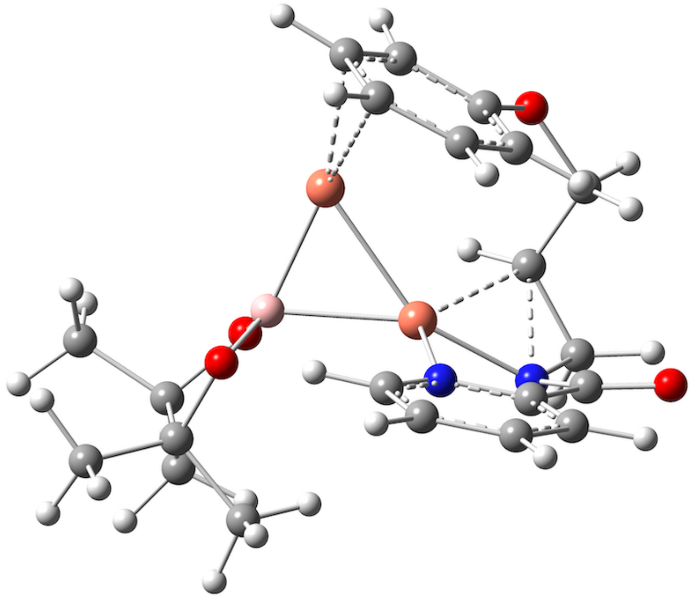

TS8 Substrate: 1c
Path: Markovnikov

CARTESIAN COORDINATES

| | | | |
|---|---|---|---|
| C | 1.303245 | -0.774821 | 2.519760 |
| N | 1.099895 | 1.139769 | 2.023652 |
| C | 2.143063 | 1.837370 | 1.402146 |
| O | 3.215448 | 2.099559 | 1.931126 |
| C | 1.386728 | 0.493161 | 3.294479 |
| C | 1.807906 | 2.288702 | 0.001752 |
| C | 2.590731 | 3.228503 | -0.665976 |
| C | 2.218776 | 3.589358 | -1.964779 |
| C | 1.098618 | 2.990074 | -2.547193 |
| C | 0.384219 | 2.037285 | -1.814962 |
| N | 0.735633 | 1.707992 | -0.567438 |
| Cu | -0.233174 | -1.783257 | -0.630598 |
| H | 3.458067 | 3.652190 | -0.170891 |
| H | 2.795320 | 4.328564 | -2.513276 |
| H | -0.492860 | 1.528376 | -2.203897 |
| H | 0.777264 | 3.255036 | -3.549597 |
| H | 2.368831 | 0.736704 | 3.707867 |
| Cu | 0.131029 | 0.140906 | 0.685551 |
| B | -1.790751 | -0.575125 | -0.107235 |
| O | -2.632392 | -0.841451 | 0.941650 |
| O | -2.328893 | 0.299136 | -1.024538 |
| C | -3.940642 | -0.235209 | 0.627510 |
| C | -3.544991 | 0.870384 | -0.415585 |
| H | 0.426581 | -1.412146 | 2.611825 |
| C | -4.570904 | 1.119998 | -1.513448 |
| H | -5.516613 | 1.464013 | -1.079412 |
| H | -4.207389 | 1.897693 | -2.192765 |
| H | -4.763493 | 0.219265 | -2.100201 |
| C | -3.107048 | 2.190119 | 0.230151 |
| H | -2.666853 | 2.835073 | -0.536691 |
| H | -3.955733 | 2.717144 | 0.677113 |
| H | -2.350922 | 2.022023 | 1.004931 |
| C | -4.801825 | -1.355325 | 0.036635 |
| H | -4.861431 | -2.177376 | 0.756235 |

| | | | |
|---|---|---|---|
| H | -5.817806 | -1.004599 | -0.171388 |
| H | -4.369874 | -1.743922 | -0.891243 |
| C | -4.550223 | 0.288158 | 1.922414 |
| H | -5.482594 | 0.825526 | 1.714778 |
| H | -4.781734 | -0.549658 | 2.587332 |
| H | -3.869333 | 0.961340 | 2.448429 |
| C | 2.603710 | -1.412157 | 2.123628 |
| H | 3.342449 | -0.665540 | 1.812104 |
| H | 2.999723 | -1.884955 | 3.034272 |
| O | 2.472143 | -2.486074 | 1.195929 |
| C | 2.289929 | -2.208783 | -0.127107 |
| C | 2.872803 | -1.125500 | -0.800346 |
| C | 1.476786 | -3.128421 | -0.836491 |
| C | 2.616245 | -0.938345 | -2.164087 |
| H | 3.520870 | -0.430991 | -0.277641 |
| C | 1.223140 | -2.915654 | -2.208075 |
| H | 1.143717 | -4.030268 | -0.327489 |
| C | 1.782417 | -1.808778 | -2.869951 |
| H | 3.070545 | -0.092570 | -2.671840 |
| H | 0.622141 | -3.636496 | -2.754707 |
| H | 1.581039 | -1.647391 | -3.923773 |
| H | 0.590822 | 0.652364 | 4.022748 |

Electronic Energy = -1645.85266128 (Hartree/Particle)
Dipole Moment (Debye): 2.7354
index 1
Number of imaginary frequencies= 1
Negatives Eigenvalues: -168.079

THERMOCHEMICAL DATA

Temperature 298.150 Kelvin. Pressure 1.00000 Atm.
Zero-point correction= 0.447730
(Hartree/Particle)
Thermal correction to Energy= 0.477240
Thermal correction to Enthalpy= 0.478184
Thermal correction to Gibbs Free Energy= 0.385894
Sum of electronic and zero-point Energies= -1645.404931
Sum of electronic and thermal Energies= -1645.375421
Sum of electronic and thermal Enthalpies= -1645.374477
Sum of electronic and thermal Free Energies= -1645.466767
PCM-SP (solvent=THF;6-311+g(2d,p)) = -1646.25513609
Corrected Free Energy = -1645.86924181

---

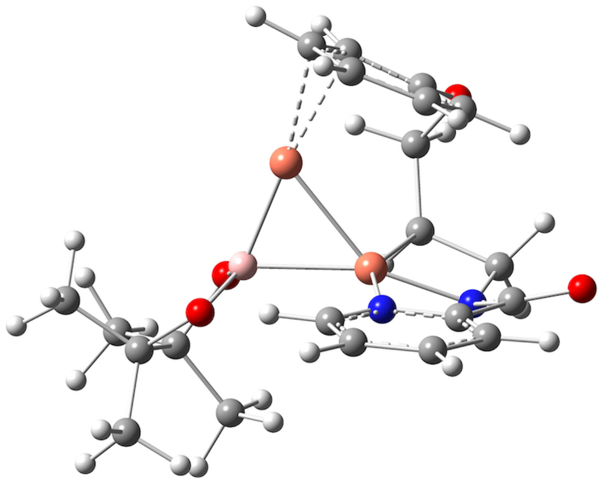

I6 Substrate: 1c
Path: Markovnikov

CARTESIAN COORDINATES

| | | | |
|---|---|---|---|
| C | -0.400877 | 1.540152 | 2.053085 |
| N | -1.335375 | -0.429743 | 2.182612 |
| C | -2.413971 | -1.165678 | 1.772516 |
| O | -3.542630 | -1.114419 | 2.247875 |
| C | -1.439197 | 0.828390 | 2.913207 |
| C | -2.045302 | -2.100135 | 0.637668 |
| C | -2.844747 | -3.172053 | 0.258324 |
| C | -2.422017 | -3.967004 | -0.811634 |
| C | -1.227468 | -3.655219 | -1.466917 |
| C | -0.490130 | -2.549064 | -1.040940 |
| N | -0.899811 | -1.797403 | -0.009194 |
| Cu | 0.151703 | 1.126694 | -1.258914 |
| H | -3.766496 | -3.359875 | 0.799383 |
| H | -3.015576 | -4.819780 | -1.128247 |
| H | 0.445719 | -2.250805 | -1.502135 |
| H | -0.866087 | -4.256374 | -2.295003 |
| H | -2.438897 | 1.269240 | 2.841541 |
| H | -1.162897 | 0.727776 | 3.968420 |
| Cu | -0.204076 | -0.046291 | 0.762754 |
| B | 1.736913 | 0.258179 | -0.274238 |
| O | 2.593016 | 0.977573 | 0.511551 |
| O | 2.295451 | -0.868357 | -0.817027 |
| C | 3.801308 | 0.146707 | 0.708675 |
| C | 3.748455 | -0.813800 | -0.534808 |
| H | 0.619200 | 1.554331 | 2.444066 |
| C | -0.787223 | 2.853305 | 1.405542 |
| H | -0.892875 | 3.600512 | 2.203694 |
| H | -0.010192 | 3.198535 | 0.715675 |
| O | -2.079508 | 2.827293 | 0.772564 |
| C | -2.159651 | 2.226261 | -0.451173 |
| C | -3.070849 | 1.179994 | -0.624553 |
| C | -1.361284 | 2.661315 | -1.540336 |
| C | -3.143129 | 0.513021 | -1.854364 |
| H | -3.693015 | 0.883633 | 0.214554 |
| C | -1.427151 | 1.961215 | -2.768582 |
| H | -0.788959 | 3.582481 | -1.456077 |
| C | -2.308206 | 0.871957 | -2.915802 |
| H | -3.845892 | -0.306873 | -1.970859 |

| | | | |
|---|---|---|---|
| H | -0.855614 | 2.322212 | -3.619823 |
| H | -2.357696 | 0.341815 | -3.861368 |
| C | 4.237101 | -2.233229 | -0.275459 |
| H | 4.155783 | -2.826802 | -1.191393 |
| H | 5.289876 | -2.222229 | 0.028430 |
| H | 3.654439 | -2.727790 | 0.504923 |
| C | 4.404594 | -0.231955 | -1.789408 |
| H | 5.494126 | -0.207045 | -1.688317 |
| H | 4.153527 | -0.859711 | -2.649778 |
| H | 4.051144 | 0.784347 | -1.991710 |
| C | 3.595571 | -0.571456 | 2.046180 |
| H | 4.466050 | -1.181135 | 2.307440 |
| H | 3.451085 | 0.174077 | 2.834135 |
| H | 2.712111 | -1.218765 | 2.021609 |
| C | 5.016676 | 1.062552 | 0.765969 |
| H | 4.961255 | 1.697220 | 1.655712 |
| H | 5.935763 | 0.469065 | 0.828710 |
| H | 5.079029 | 1.711012 | -0.110516 |

Electronic Energy = -1645.85932718 (Hartree/Particle)
Dipole Moment (Debye): 5.2390
index 0
Harmonic frequencies
Number of imaginary frequencies= 0

THERMOCHEMICAL DATA

Temperature 298.150 Kelvin. Pressure 1.00000 Atm.
Zero-point correction= 0.449249
(Hartree/Particle)
Thermal correction to Energy= 0.478828
Thermal correction to Enthalpy= 0.479773
Thermal correction to Gibbs Free Energy= 0.387601
Sum of electronic and zero-point Energies= -1645.410078
Sum of electronic and thermal Energies= -1645.380499
Sum of electronic and thermal Enthalpies= -1645.379554
Sum of electronic and thermal Free Energies= -1645.471727
PCM-SP (solvent=THF;6-311+g(2d,p)) = -1646.26409937
Corrected Free Energy = -1645.87649919

-------------------------------------------------------

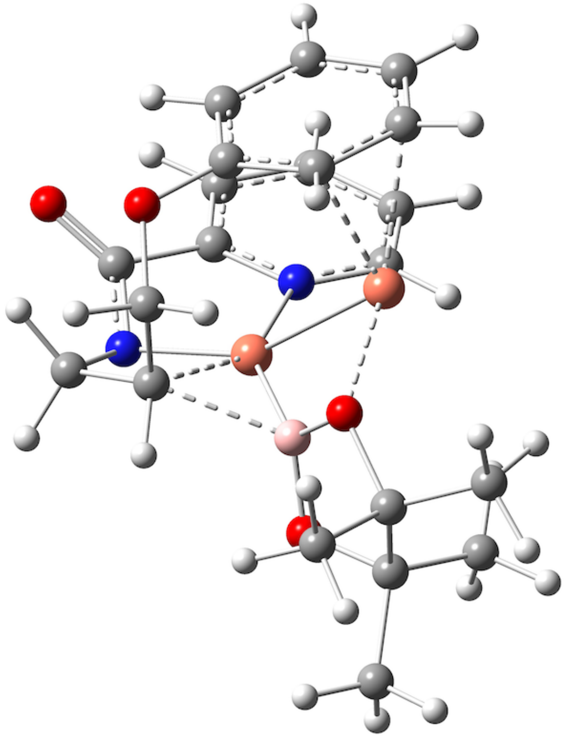

TS9 Substrate: 1c
Path: Markovnikov

CARTESIAN COORDINATES

| | | | |
|---|---|---|---|
| C | 0.597771 | -0.676364 | 2.051608 |
| N | -1.357094 | -1.897916 | 1.399656 |
| C | -2.644232 | -1.540901 | 1.240642 |
| O | -3.521502 | -1.307586 | 2.076809 |
| C | -0.563276 | -1.604011 | 2.583794 |
| C | -2.938850 | -1.347152 | -0.251955 |
| C | -4.221614 | -1.452440 | -0.780344 |
| C | -4.414273 | -1.218681 | -2.142915 |
| C | -3.319225 | -0.861538 | -2.936490 |
| C | -2.068547 | -0.742155 | -2.337408 |
| N | -1.883808 | -0.981052 | -1.026649 |
| Cu | 0.498479 | 1.220316 | -0.777353 |
| H | -5.034752 | -1.715115 | -0.111501 |
| H | -5.403017 | -1.313256 | -2.582718 |
| H | -1.188176 | -0.451402 | -2.900288 |
| H | -3.427867 | -0.672435 | -3.999451 |
| H | -1.134985 | -1.080317 | 3.359925 |
| H | -0.144520 | -2.521262 | 3.009869 |
| Cu | -0.305568 | -0.900470 | 0.171755 |
| B | 1.655566 | -0.561247 | 0.297977 |
| O | 2.381768 | 0.622177 | 0.185946 |
| O | 2.360588 | -1.664613 | -0.073717 |
| C | 3.775567 | 0.231574 | -0.190711 |
| C | 3.539180 | -1.186828 | -0.822660 |
| H | 1.568095 | -1.117207 | 2.307496 |
| C | 0.551293 | 0.739232 | 2.644495 |
| H | 0.711147 | 0.643316 | 3.723404 |
| H | 1.340732 | 1.375482 | 2.238596 |
| O | -0.735668 | 1.366900 | 2.540588 |

| | | | |
|---|---|---|---|
| C | -1.102863 | 1.936936 | 1.358194 |
| C | -2.424381 | 1.753896 | 0.938638 |
| C | -0.216436 | 2.724337 | 0.578772 |
| C | -2.845555 | 2.267443 | -0.292775 |
| H | -3.092158 | 1.173895 | 1.568960 |
| C | -0.649859 | 3.211388 | -0.683294 |
| H | 0.732013 | 3.053759 | 0.995416 |
| C | -1.964486 | 2.962206 | -1.124282 |
| H | -3.870324 | 2.098662 | -0.610527 |
| H | -0.003964 | 3.883993 | -1.243611 |
| H | -2.294602 | 3.346409 | -2.084073 |
| C | 4.676331 | -2.177120 | -0.613478 |
| H | 4.421145 | -3.134633 | -1.076654 |
| H | 5.594761 | -1.804760 | -1.081145 |
| H | 4.868711 | -2.354538 | 0.446659 |
| C | 3.122192 | -1.134281 | -2.295811 |
| H | 3.959272 | -0.843925 | -2.937705 |
| H | 2.774696 | -2.124639 | -2.602772 |
| H | 2.300049 | -0.424745 | -2.454348 |
| C | 4.559628 | 0.200323 | 1.120126 |
| H | 5.613810 | -0.028656 | 0.936281 |
| H | 4.501441 | 1.180661 | 1.602477 |
| H | 4.162332 | -0.550980 | 1.810072 |
| C | 4.316934 | 1.289467 | -1.138780 |
| H | 4.431516 | 2.242043 | -0.612388 |
| H | 5.300956 | 0.990260 | -1.515551 |
| H | 3.654475 | 1.445858 | -1.994187 |

Electronic Energy = -1645.83478468 (Hartree/Particle)
Dipole Moment (Debye): 8.7563
index 0
Harmonic frequencies
Number of imaginary frequencies= 1
Negatives Eigenvalues: -170.316

THERMOCHEMICAL DATA

Temperature 298.150 Kelvin. Pressure 1.00000 Atm.
Zero-point correction= 0.449085
(Hartree/Particle)
Thermal correction to Energy= 0.477711
Thermal correction to Enthalpy= 0.478655
Thermal correction to Gibbs Free Energy= 0.390923
Sum of electronic and zero-point Energies= -1645.385699
Sum of electronic and thermal Energies= -1645.357074
Sum of electronic and thermal Enthalpies= -1645.356129
Sum of electronic and thermal Free Energies= -1645.443862
PCM-SP (solvent=THF;6-311+g(2d,p)) = -1646.24680146
Corrected Free Energy = -1645.85587878

----------------------------------------------------------------
----------------------------------------------------------------

Compound **1a** $^{1}H$ NMR (250 MHz, $CDCl_3$)

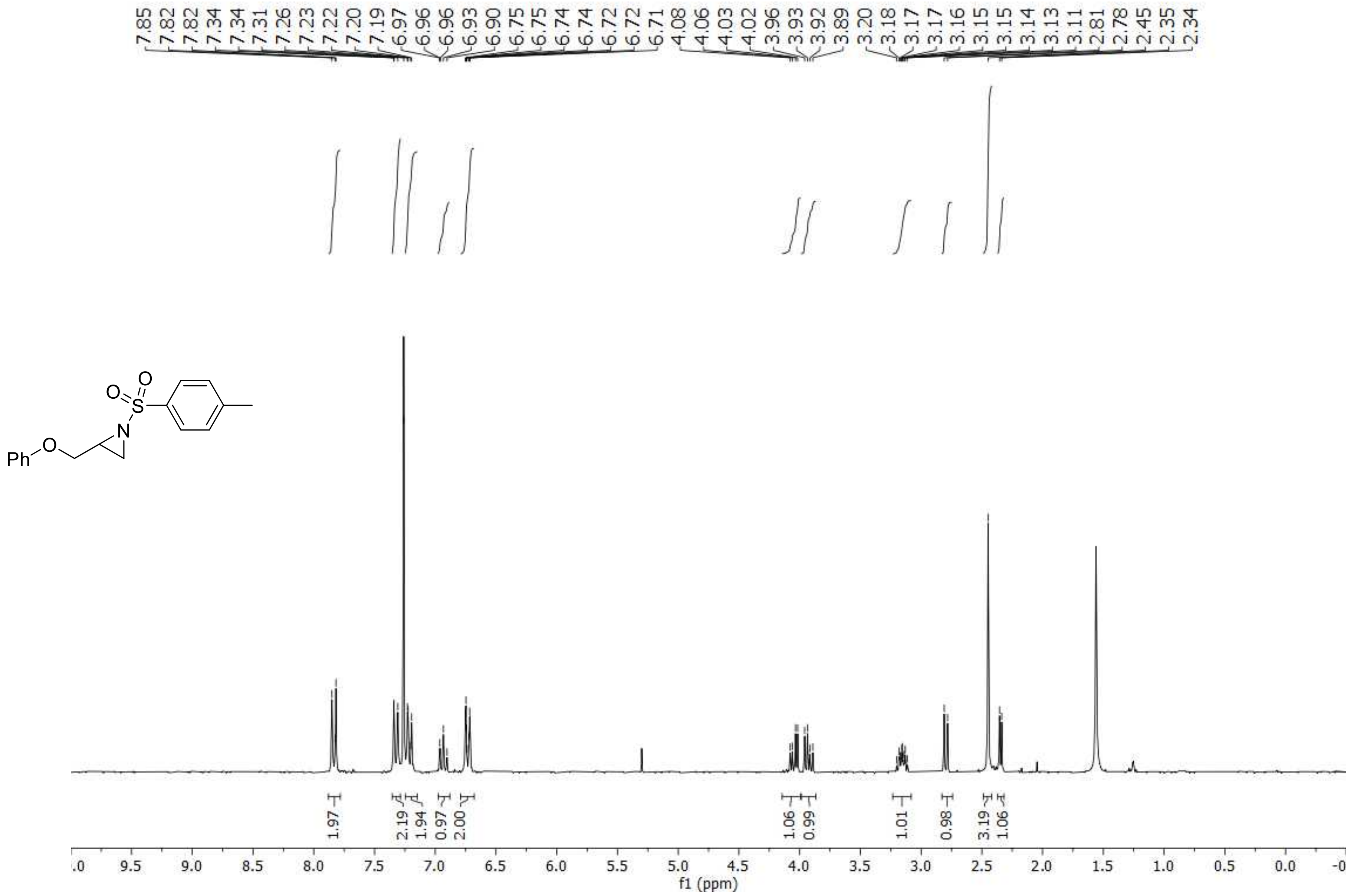

Compound **1b** $^1H$ NMR (250 MHz, $CDCl_3$)

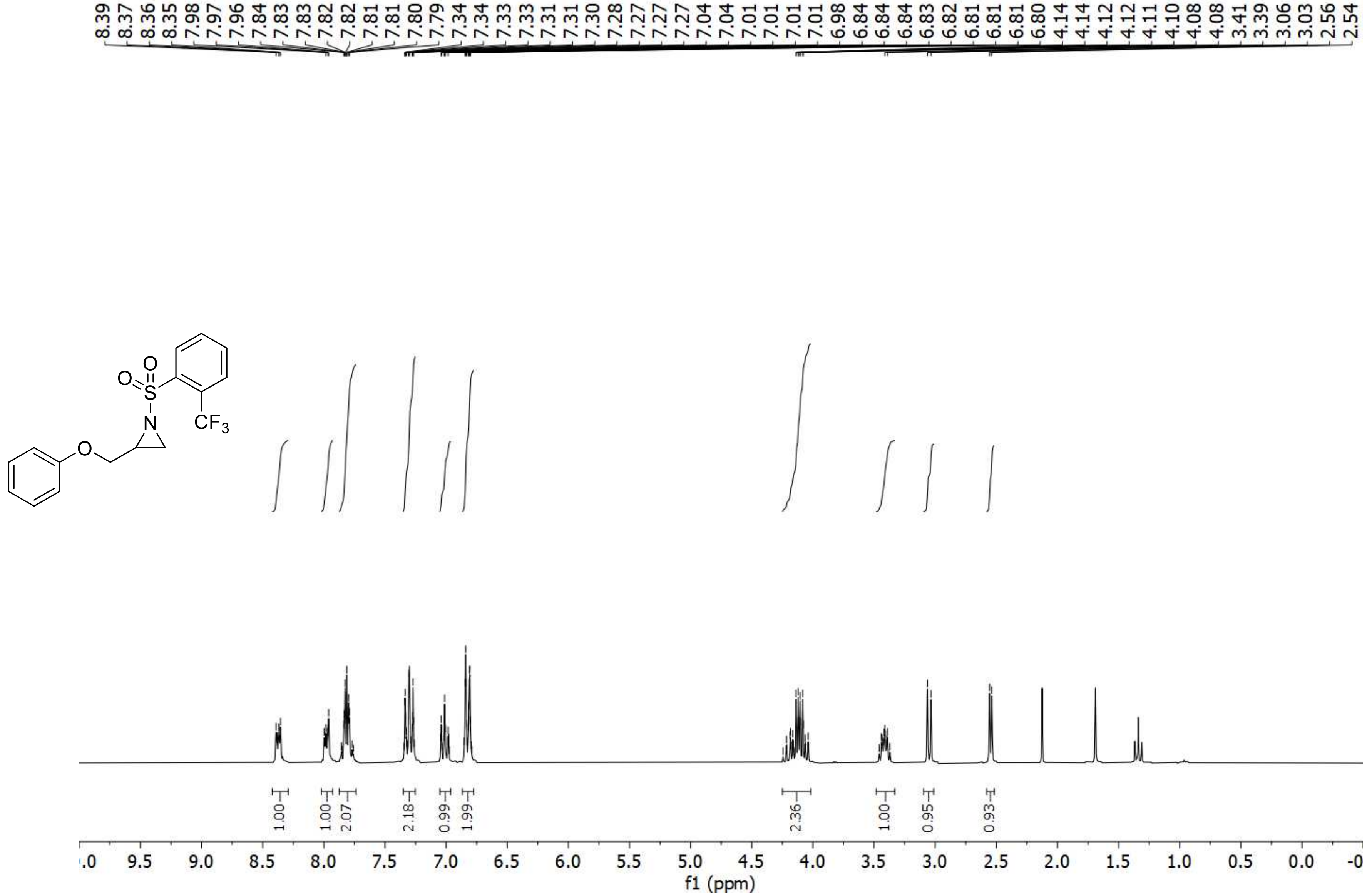

Compound **1b** $^{13}C$ NMR (63 MHz, $CDCl_3$)

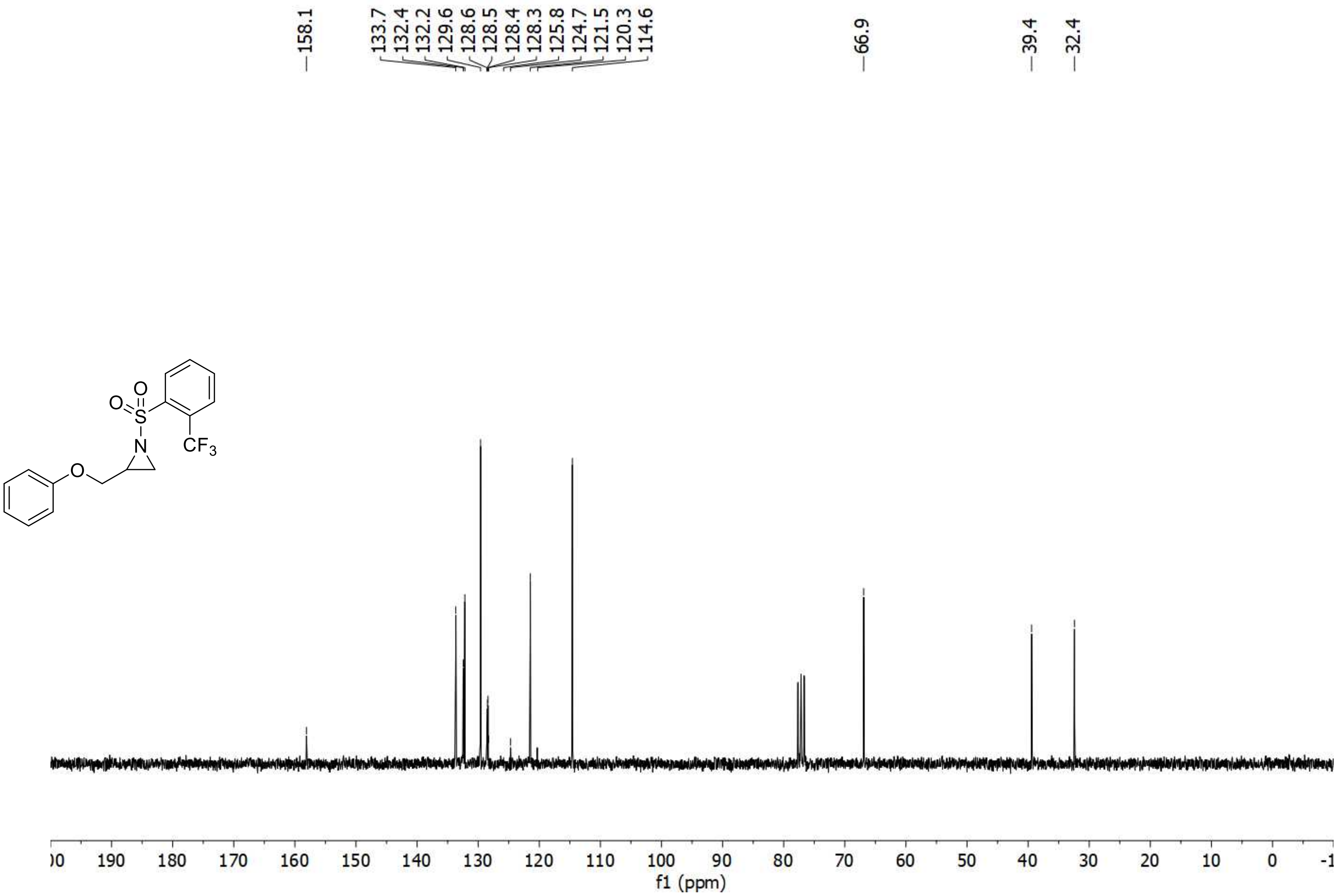

Compound **1c** $^{1}$H NMR (250 MHz, $CDCl_3$)

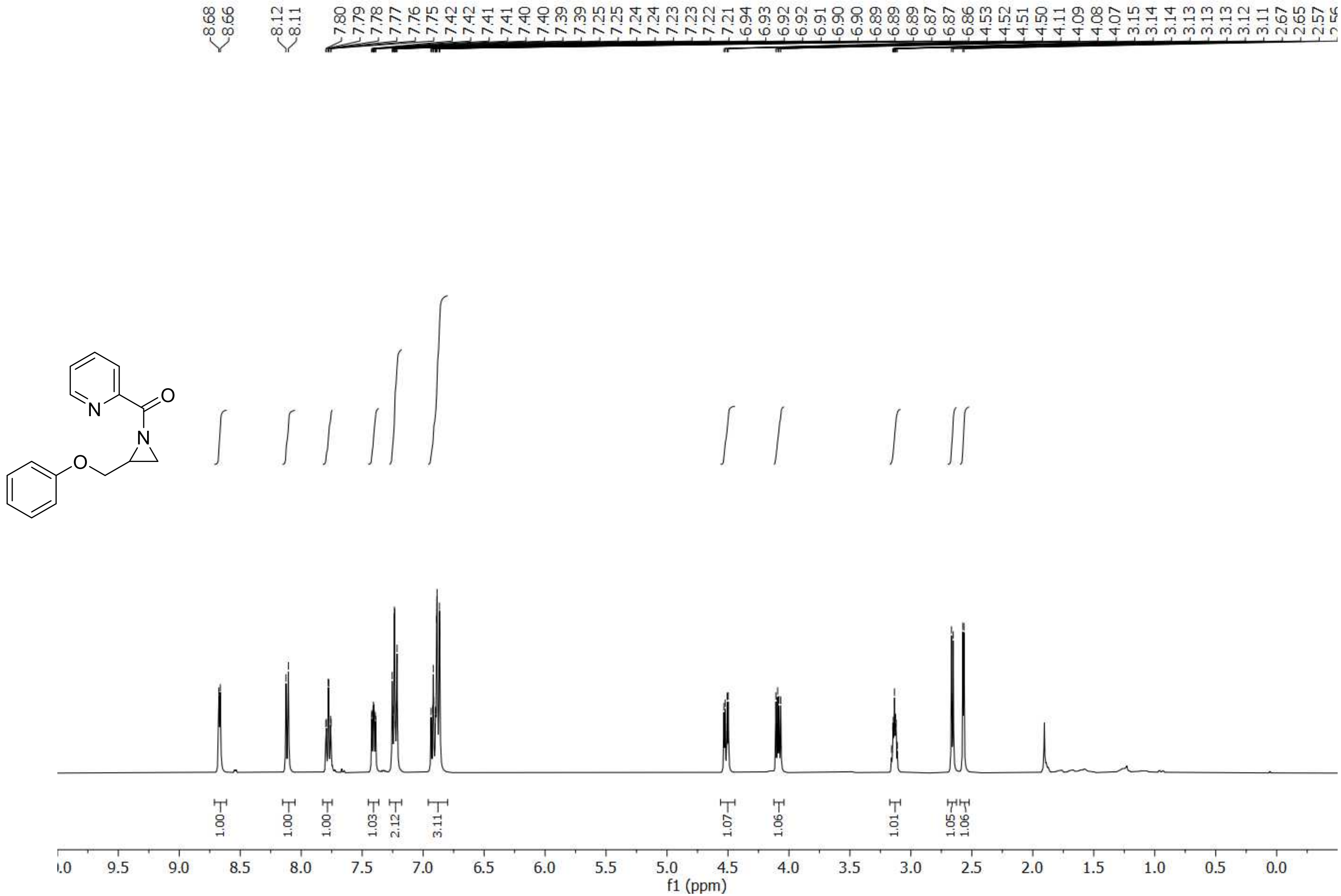

Compound **1c** $^{13}C$ NMR (101 MHz, $CDCl_3$)

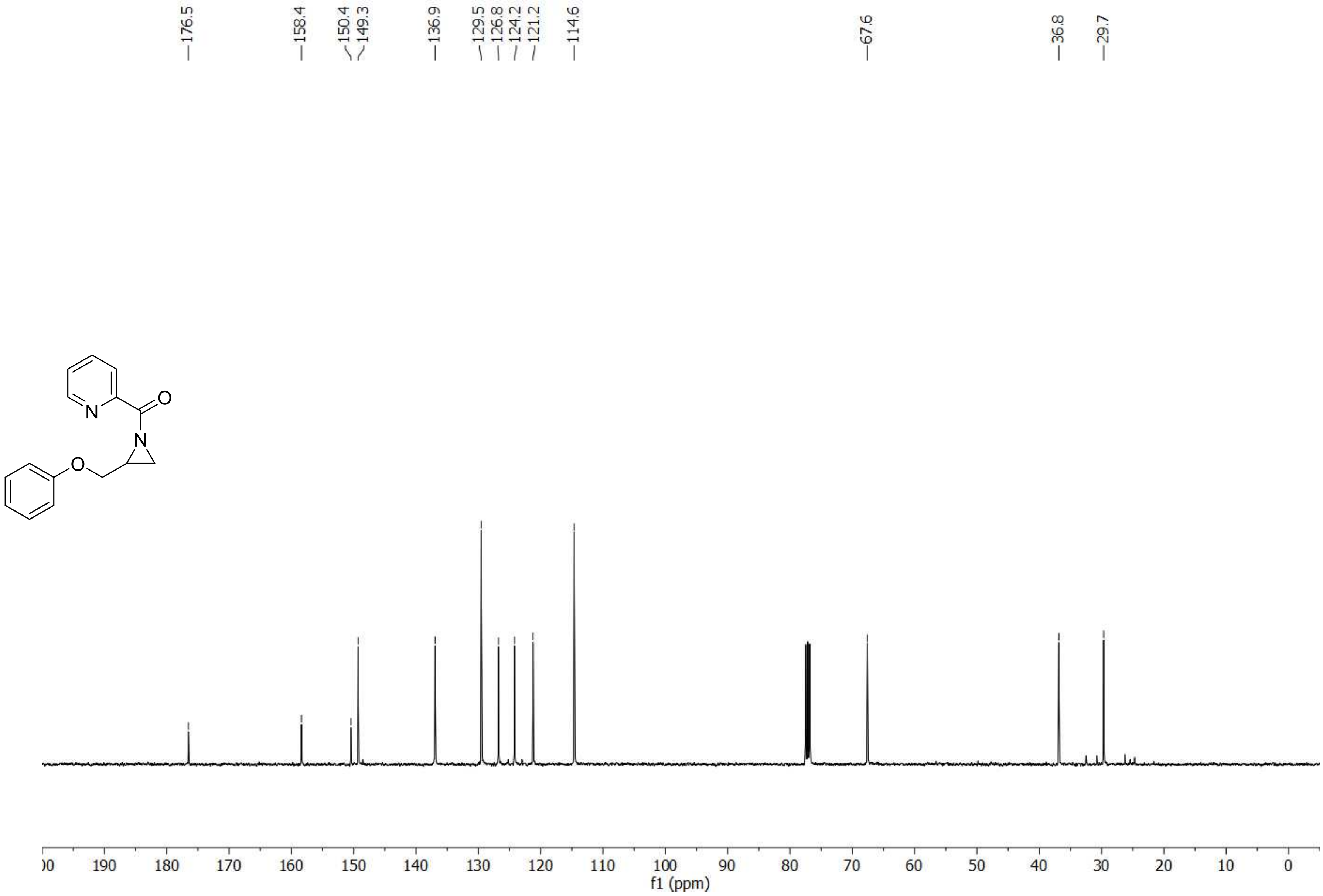

Compound **4c** $^{1}$H NMR (250 MHz, $CDCl_3$)

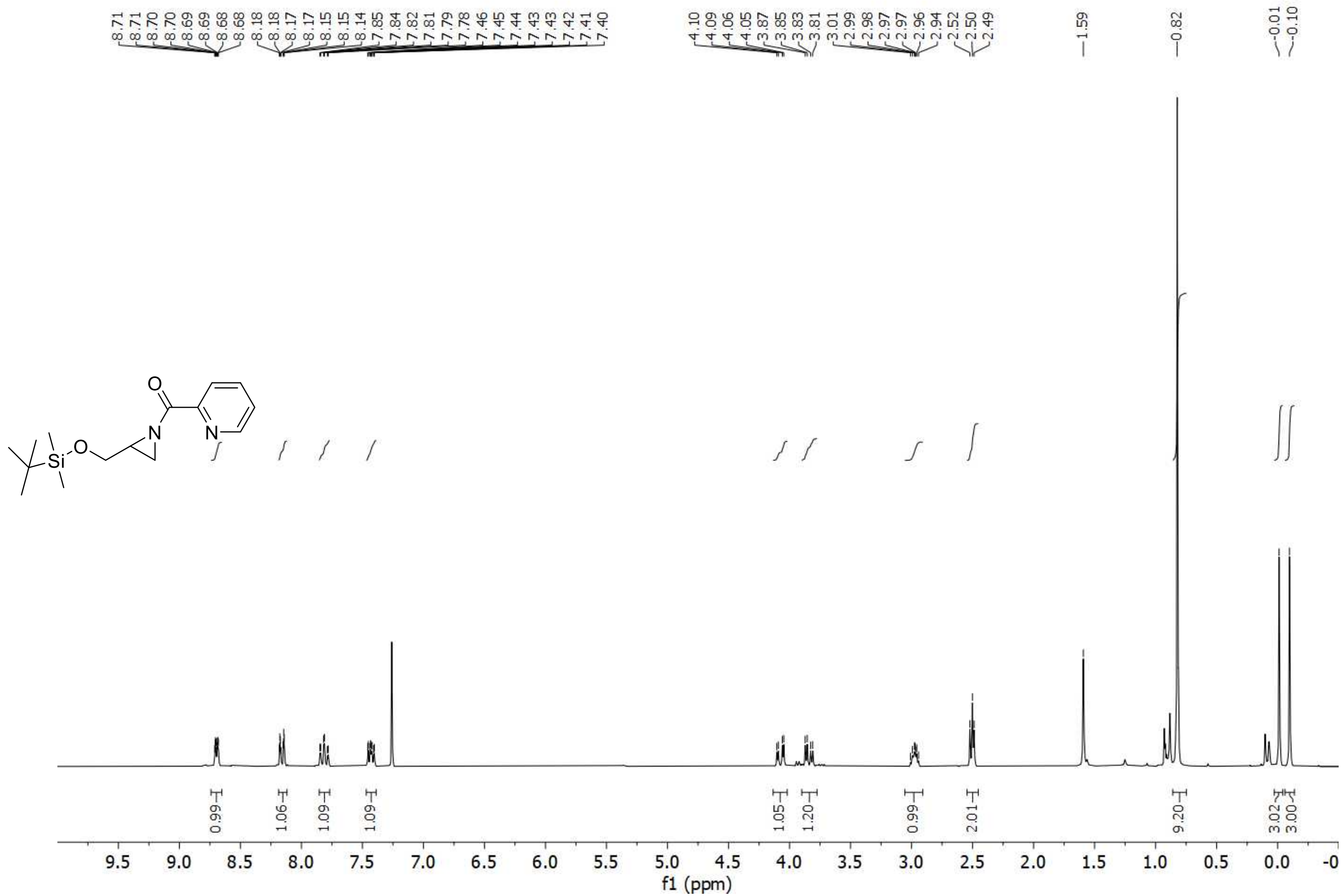

Compound **4c** $^{13}C$ NMR (63 MHz, $CDCl_3$)

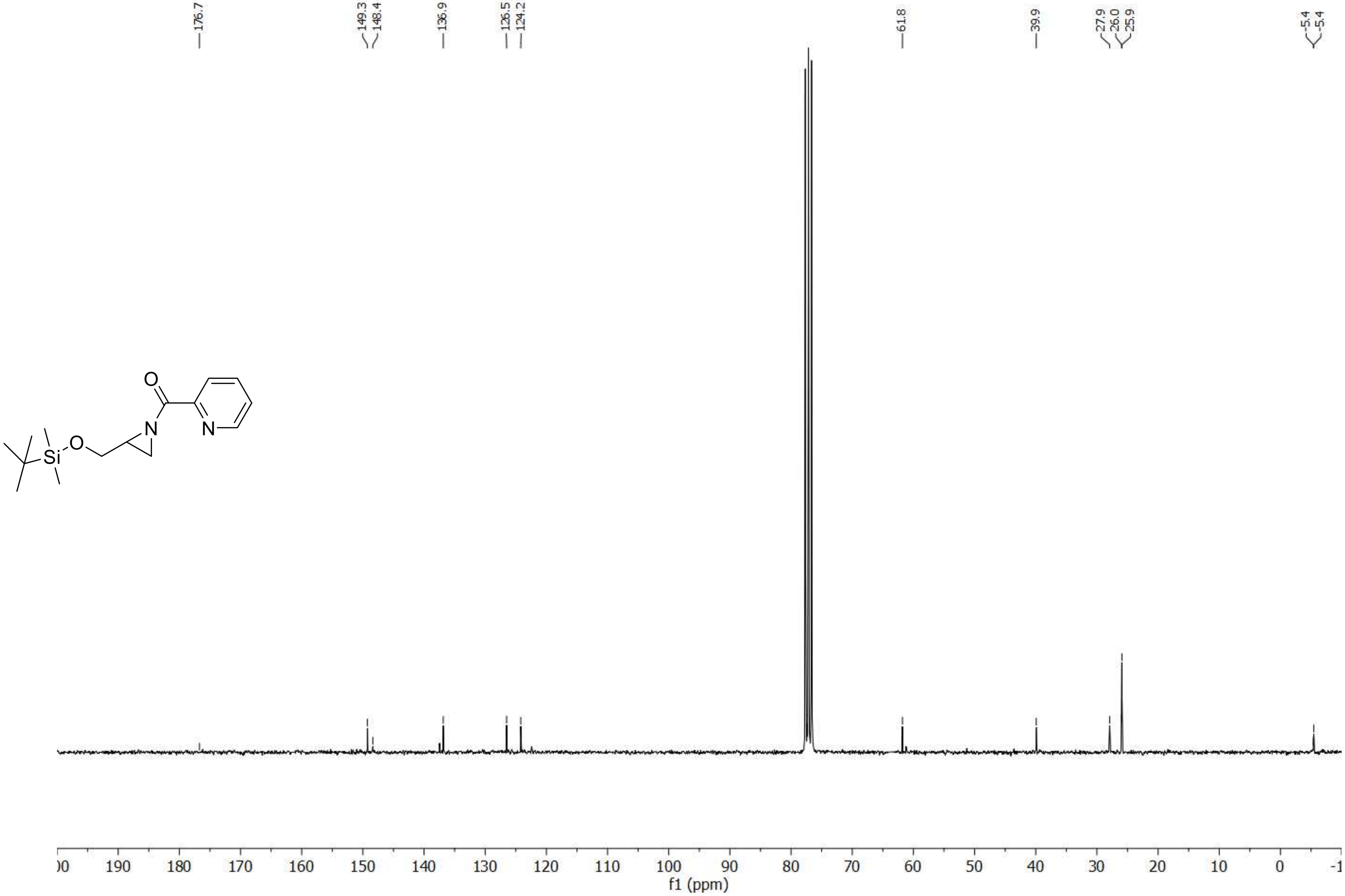

Compound **6c** $^{1}H$ NMR (400 MHz, $CDCl_3$)

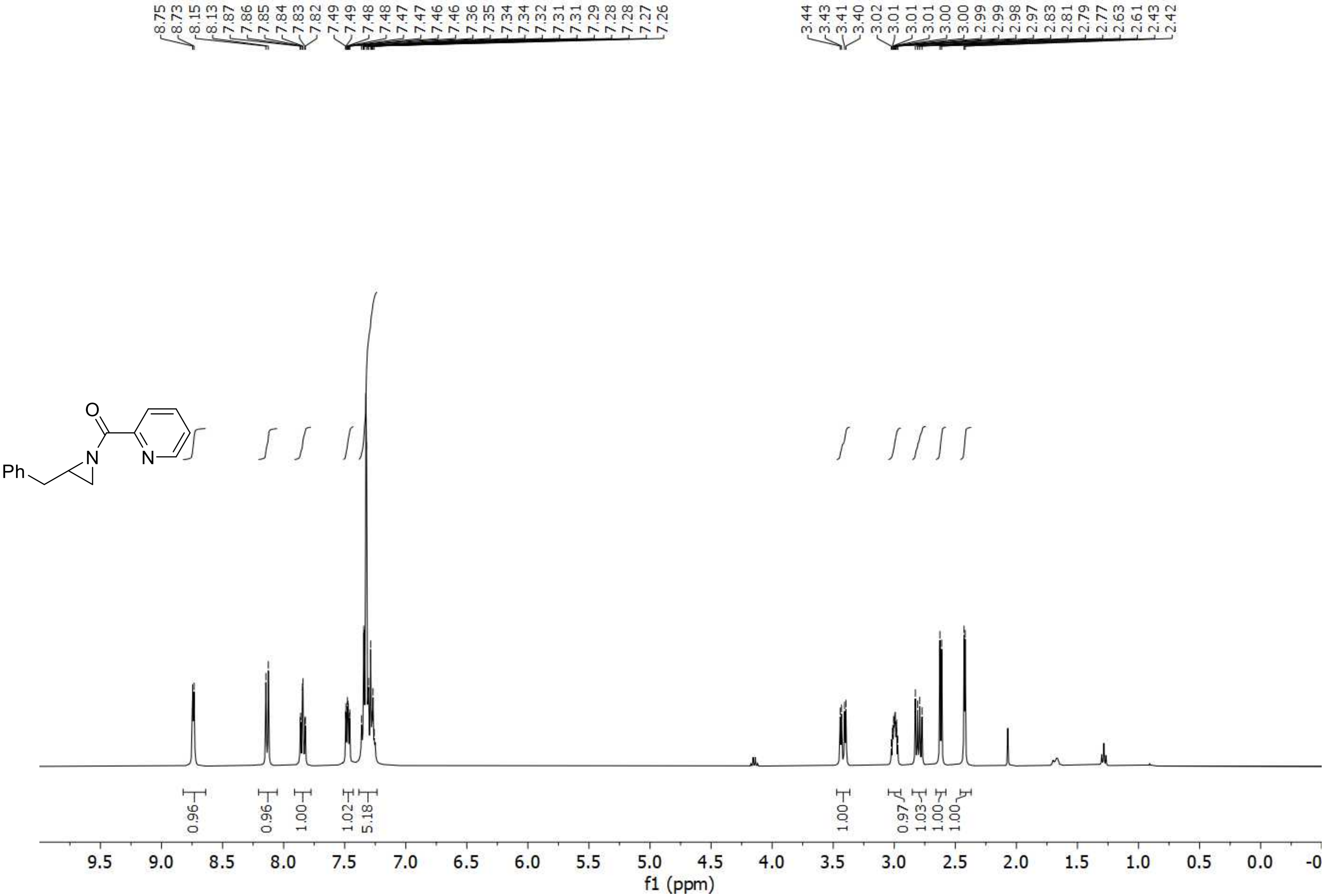

Compound **6c** $^{13}$C NMR (101 MHz, $CDCl_3$)

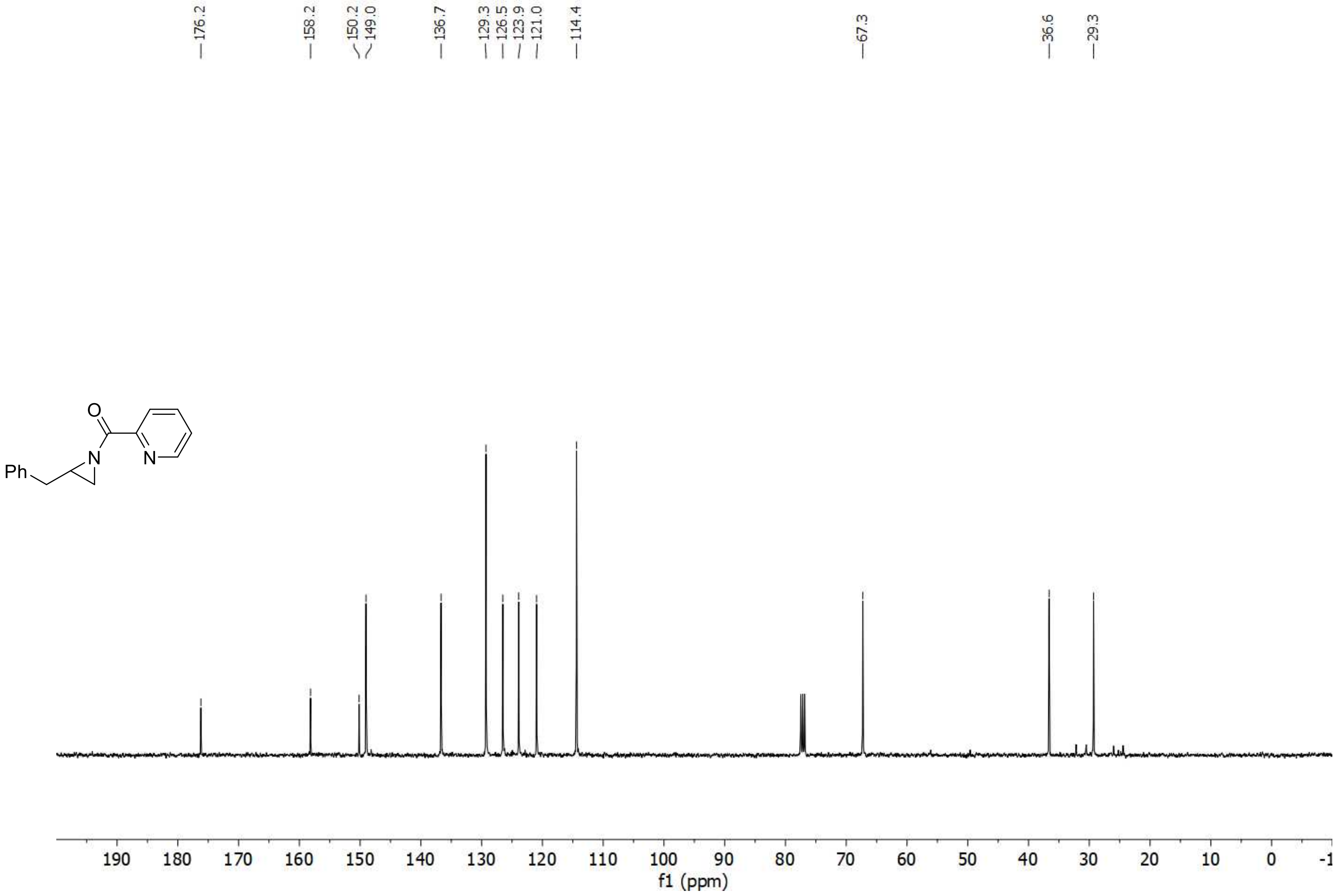

Compound **7c** $^{1}H$ NMR (250 MHz, $CDCl_3$)

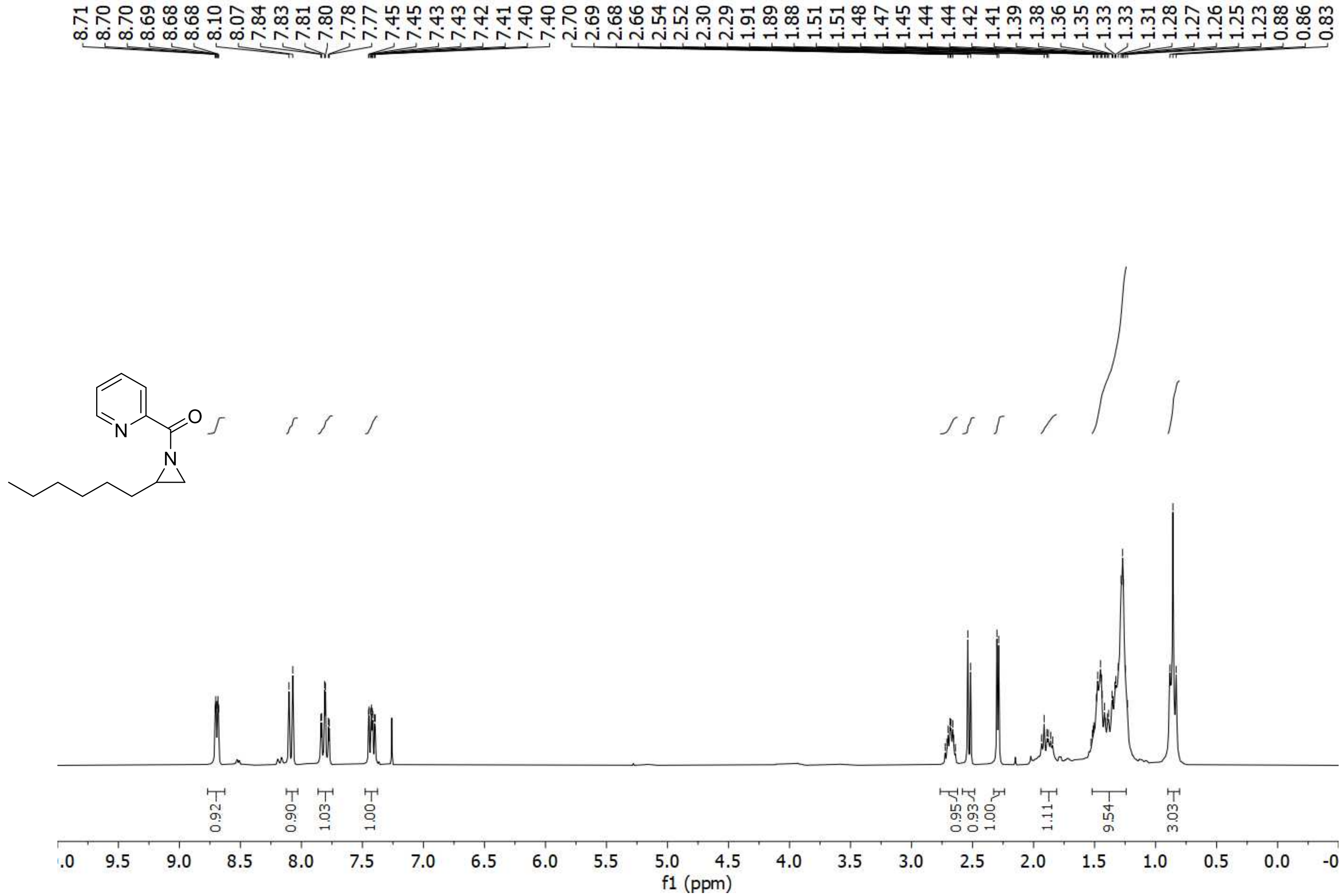

Compound **7c** $^{13}C$ NMR (101 MHz, $CDCl_3$)

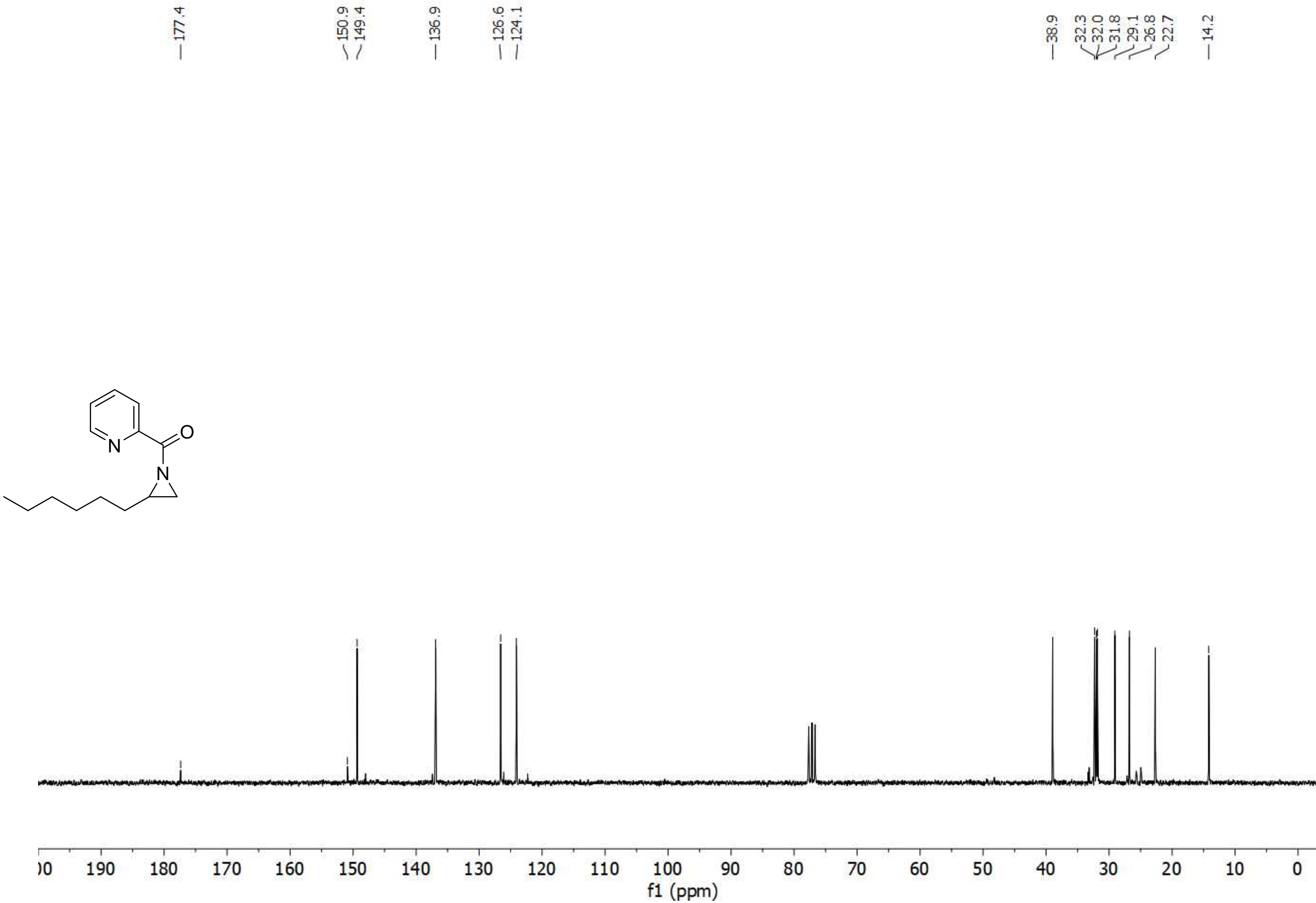

Compound **10** $^{1}$H NMR (250 MHz, $CDCl_3$)

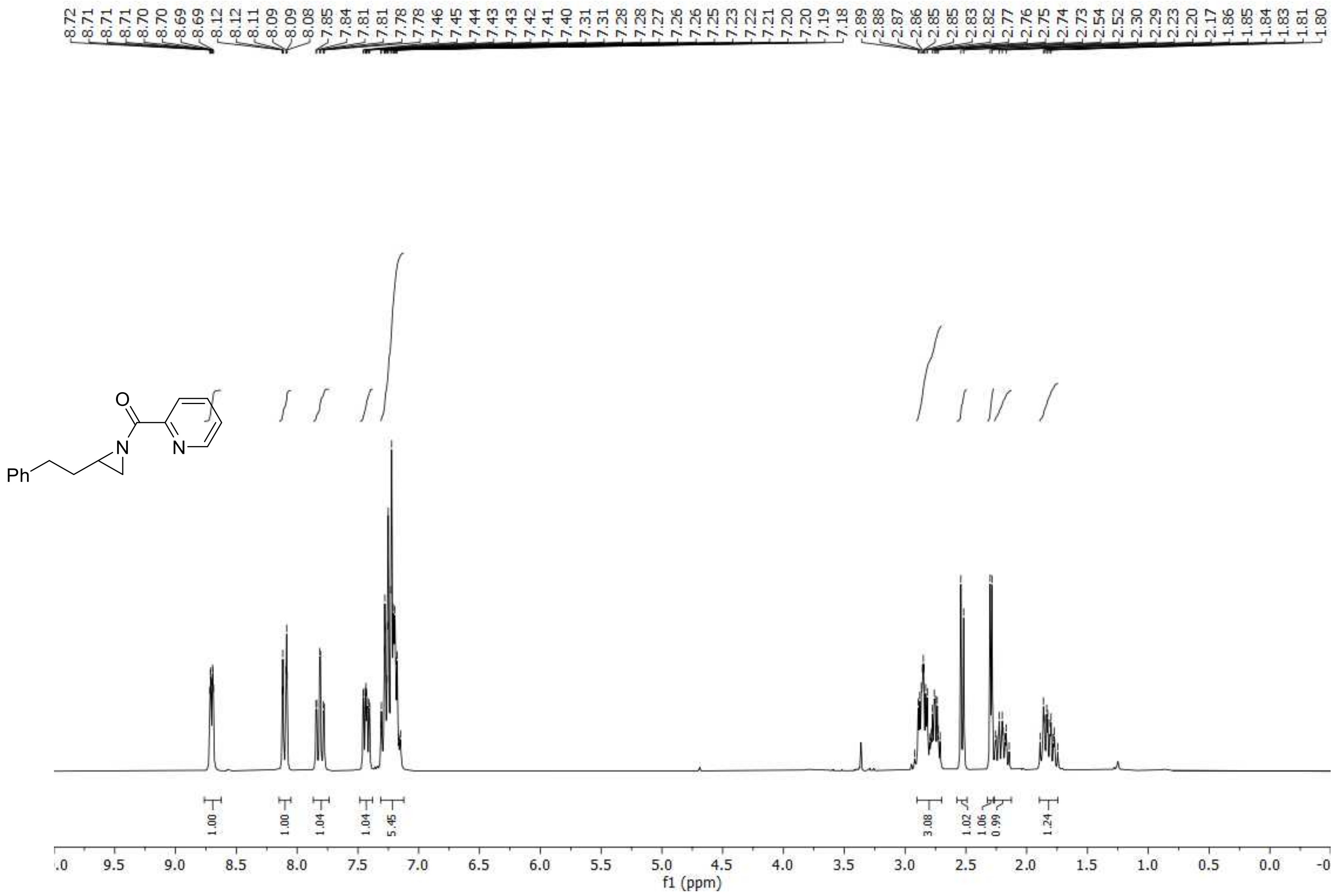

Compound **10** $^{13}$C NMR (101 MHz, $CDCl_3$)

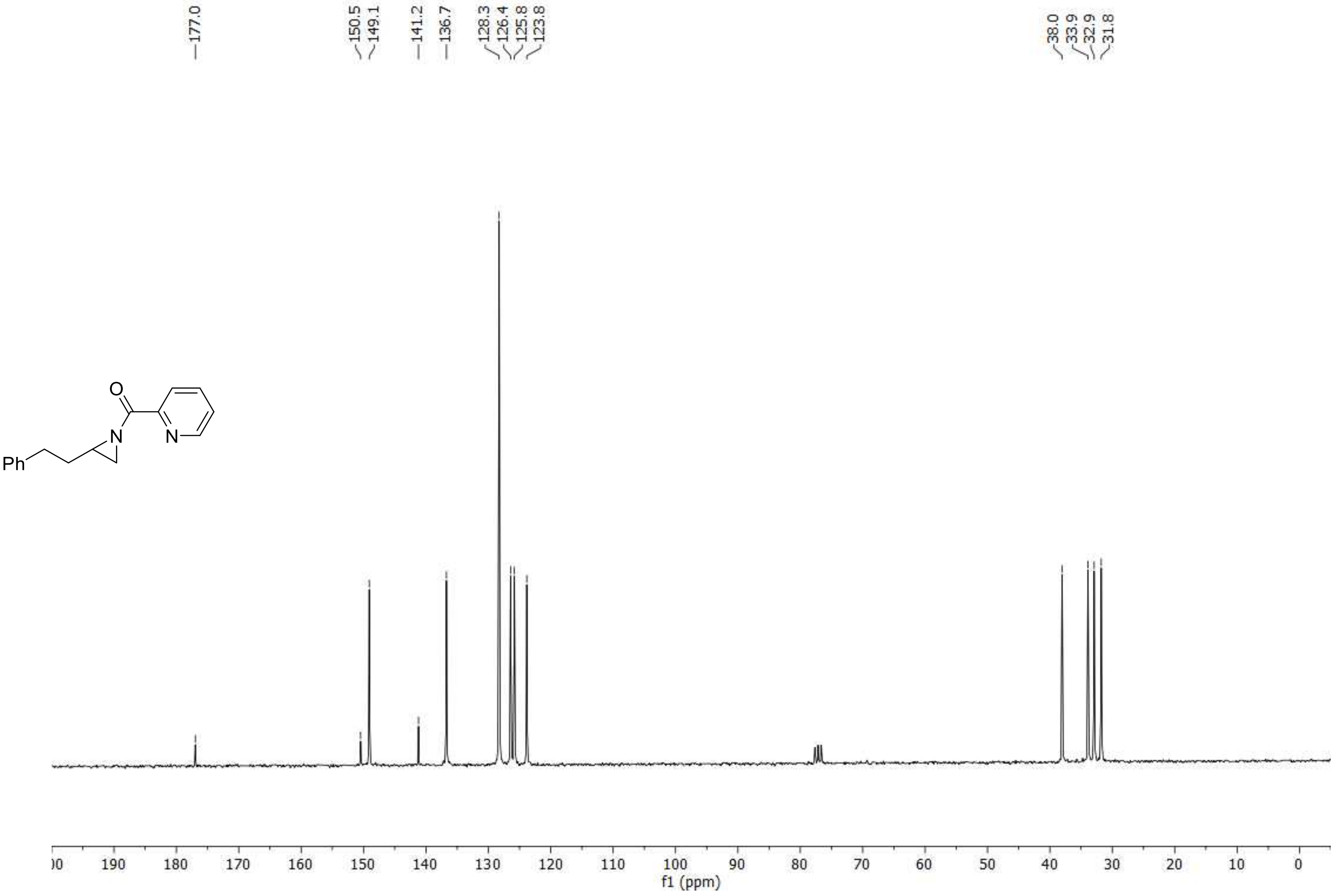

Compound **2c** $^{1}H$ NMR (250 MHz, $CDCl_3$)

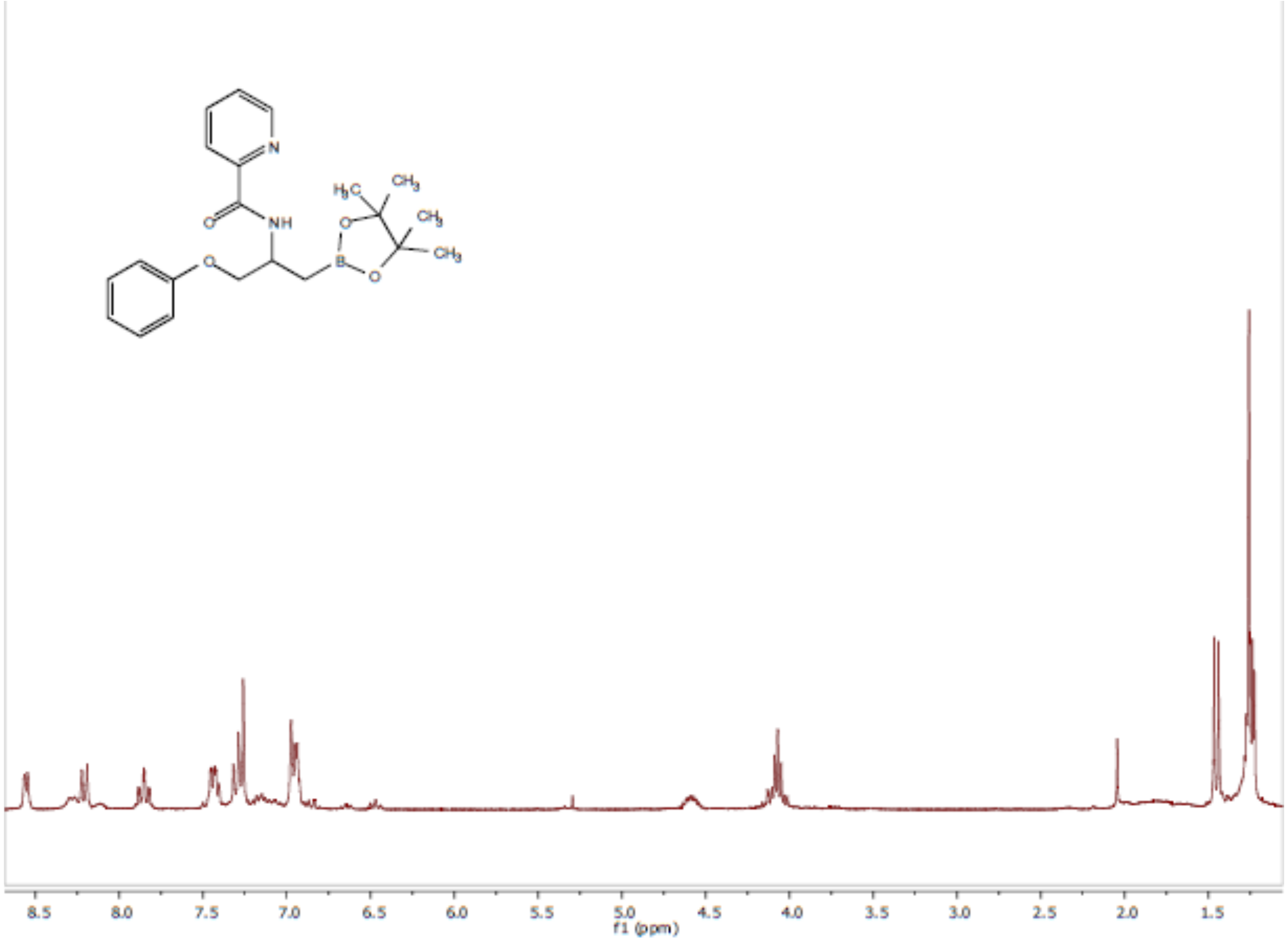

Compound **2c** $^{13}C$ NMR (63 MHz, $CDCl_3$)

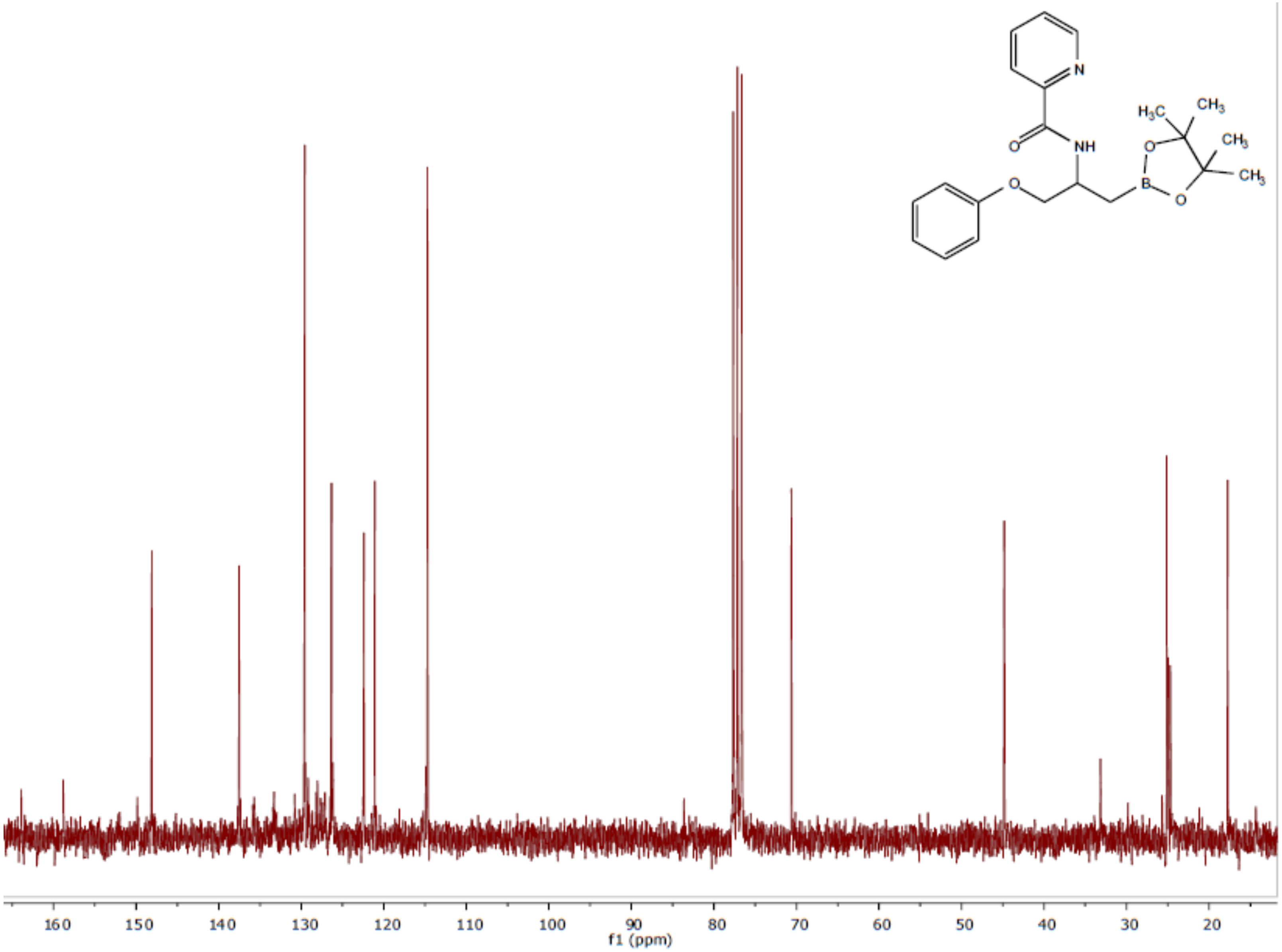

Compound **3c** $^{1}$H NMR (250 MHz, $CDCl_3$)

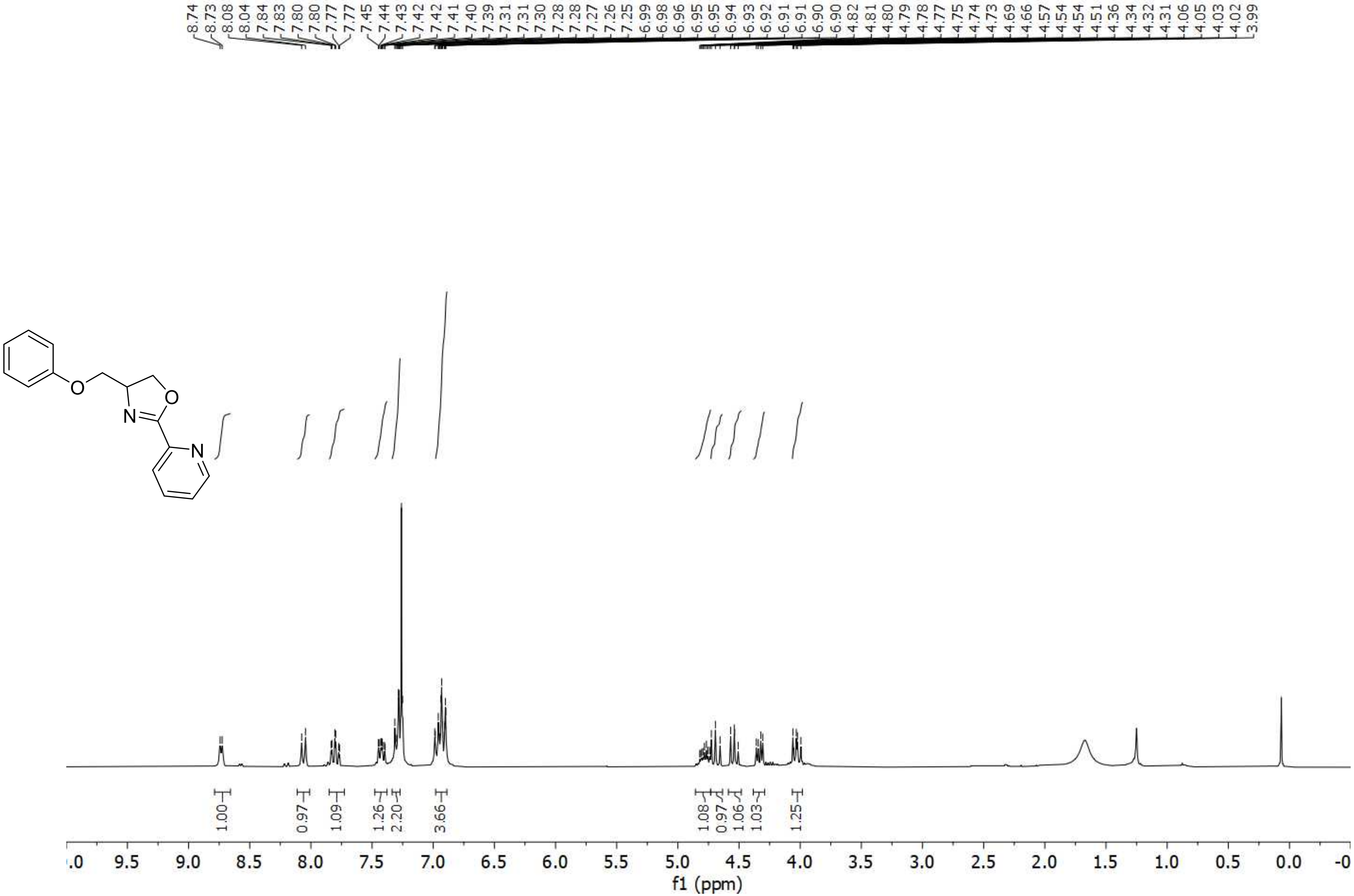

Compound **3c** $^{13}C$ NMR (63 MHz, $CDCl_3$)

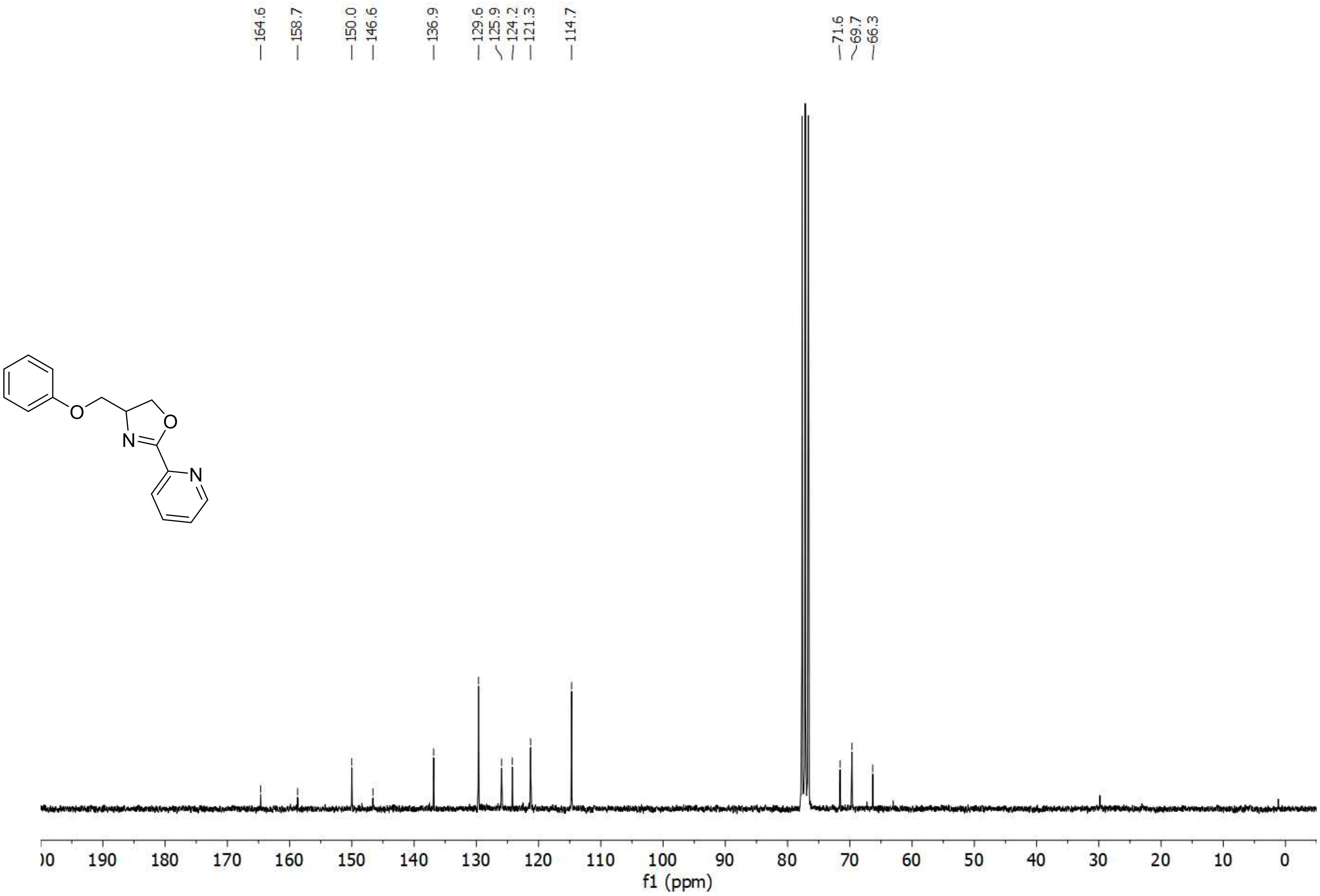

Compound **11** $^{1}H$ NMR (400 MHz, $CDCl_3$)

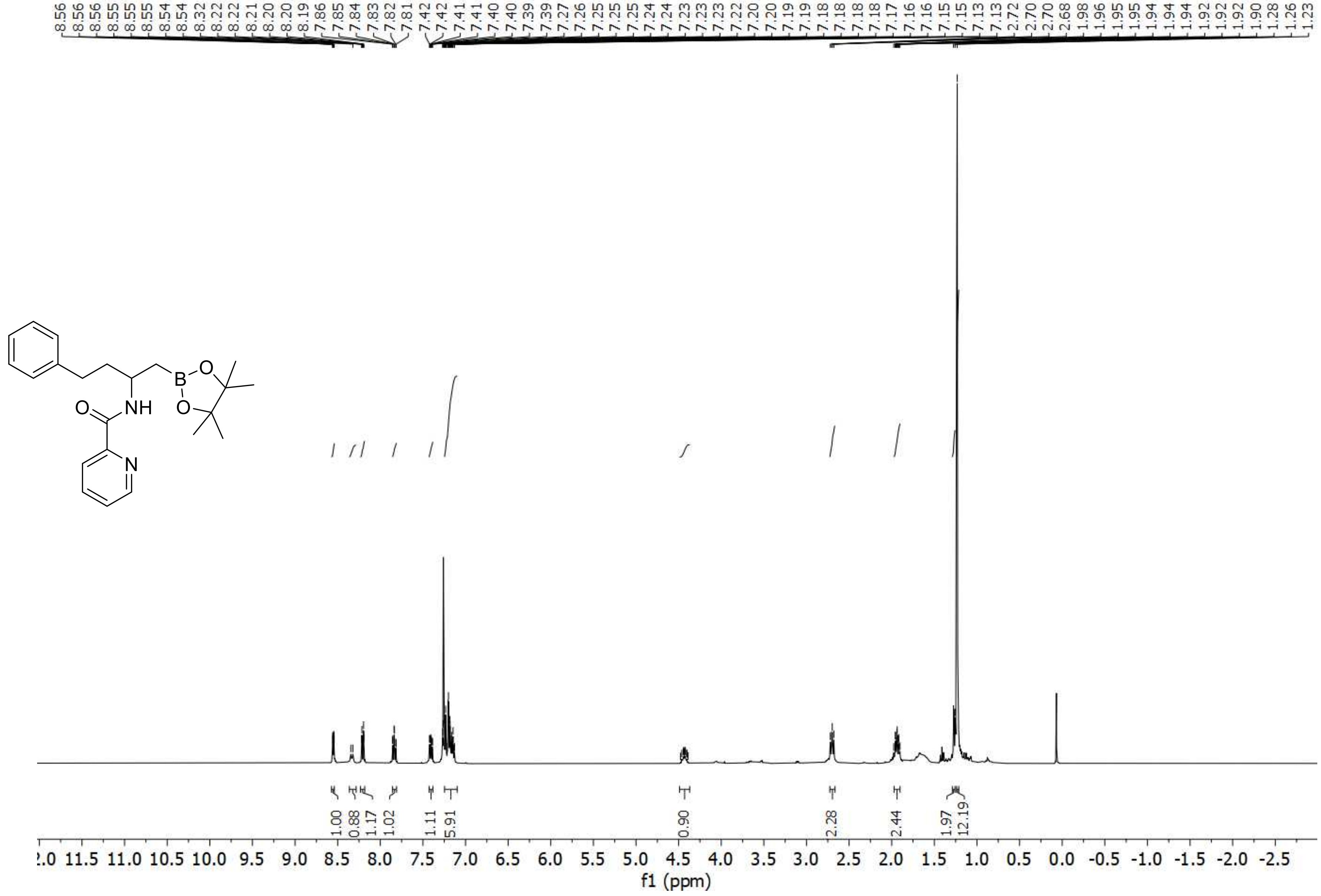

Compound **12** $^1H$ NMR (400 MHz, $CDCl_3$)

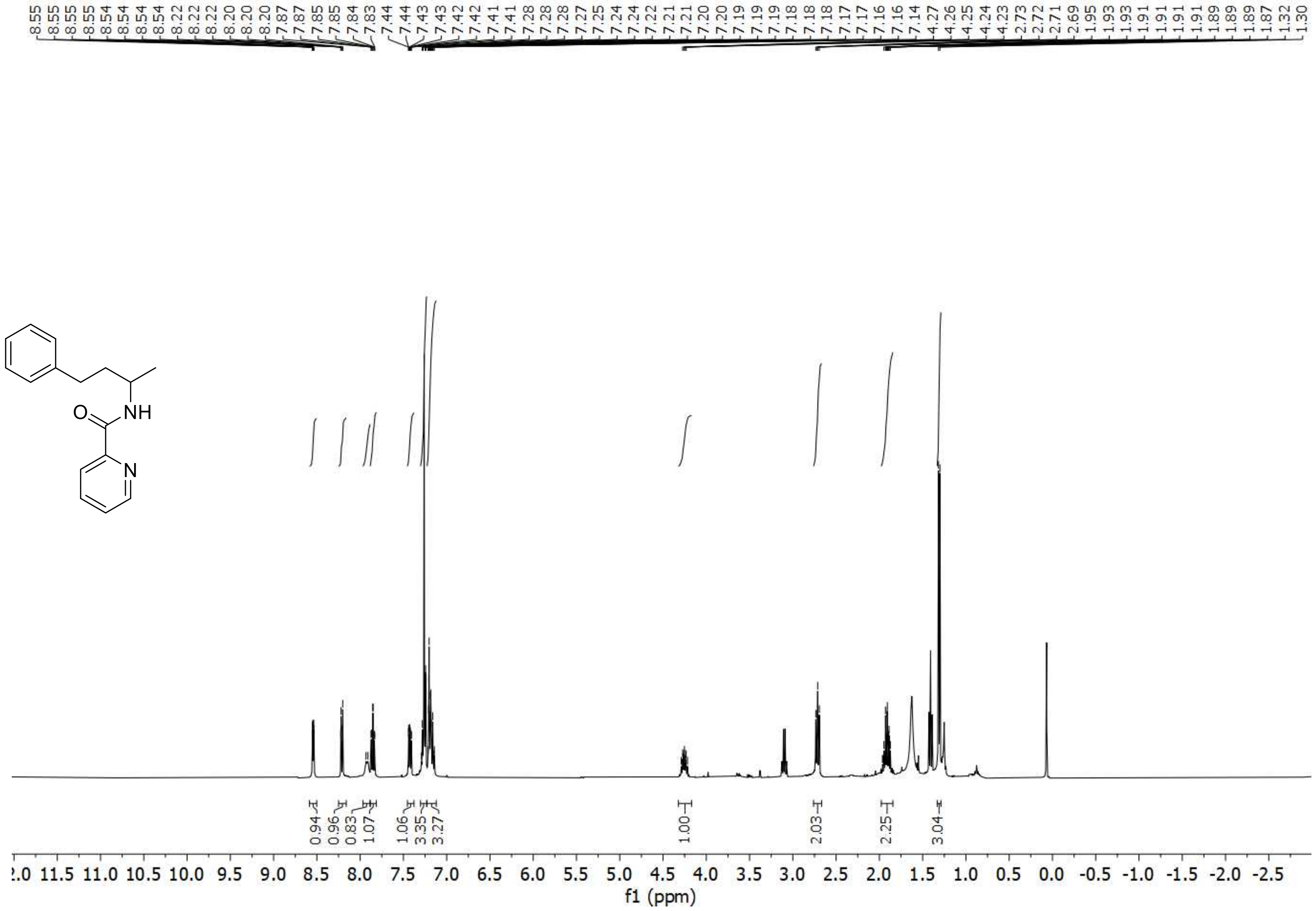

Supplement: Supplementary file 1 [file molecules-26-07399-s001.zip › molecules-1442201-supplementary.pdf]
